# Probing the Hydrophobic Part of Analogues of the Incadronate-Evidence of Their Interaction with Immunological System of Sheep

**DOI:** 10.3390/ph19020256

**Published:** 2026-02-01

**Authors:** Ewa Chmielewska, Joanna Wietrzyk, Jan Kuryszko, Zdzisław Kiełbowicz, Paweł Kafarski

**Affiliations:** 1 Department of Bioorganic Chemistry, Faculty of Chemistry, Wrocław University of Science and Technology, Wybrzeże Wyspiańskiego 27, 50-370 Wrocław, Poland; 2 Laboratory of Experimental Anticancer Therapy, Department of Experimental Oncology, Ludwik Hirszfeld Institute of Immunology and Experimental Therapy Polish Academy of Sciences, Rudolfa Weigla 12, 53-114 Wrocław, Poland; joanna.wietrzyk@hirszfeld.pl; 3 The Faculty of Veterinary Medicine, Wrocław University of Environmental and Life Sciences, Norwida 31, 50-375 Wrocław, Poland; jan.kuryszko@upwr.edu.pl (J.K.); zdzislaw.kielbowicz@upwr.edu.pl (Z.K.); 4 Department of Chemistry, Faculty of Agriculture and Forestry, University of Warmia and Mazury, Plac Łódzki 4, 10-721 Olsztyn, Poland; pawel.kafarski@uwm.edu.pl

**Keywords:** aminomethylenebisphosphonates, synthesis of aminobisphosphonic acids, osteoporosis, sheep model, ovariectomy, biological activity of amino-*gem*-bisphosphonates, preclinical studies, bone remodeling, sheep

## Abstract

**Background**: Thirty-five analogues of a promising antiosteoporotic drug, incadronate, have been synthesized and evaluated for their antiproliferative activity against mouse macrophage-like J774E cells. These cells originated from identical precursors as osteoclasts and served to select the most active compounds. Two of them, *n*-heptyl- and *n*-octyl-aminomethylenebispohosphonic acids, were then used for the medication of sheep with induced osteoporosis. They demonstrated mild antiosteoporotic activity that was documented using bone histopathology. Additionally, a decrease in the immunological response to *Corynebacterium pseudotuberculosis* was observed as a major side effect accompanied by this medication. **Methods**: Aminomethylenebisphosphonates may be obtained in the three-component reaction of aliphatic amine, triethyl orthoformate and diethyl phosphite with the further acid hydrolysis of crude esters. The obtained *N*-substituted alkiloaminomethylenebisphosphonic acids are presented alongside with their in vitro activity toward mouse macrophages J774E. **Results**: The new families of aminomethylenebisphosphonic compounds synthesized by our approach may be of practical importance due to the significant cytotoxic activity in the cell cultures investigated. Two of them were chosen for further evaluation in the treatment of induced osteoporosis in sheep. **Conclusions**: In vivo studies confirmed the mild therapeutic effects of compounds **4** (*N*-(*n*-heptylamino)methylenebisphosphonic acid and **5** (*N*-(*n*-octylamino)methylenebisphosphonic acid; however, they are not suitable analogues of incadronate for consideration as potential drugs.

## 1. Introduction

Osteoporosis is a chronic, progressive, multifactorial skeletal disorder characterized by decreased bone mass and deteriorated bone microarchitecture. These changes result in an increased risk of fracture. Osteoporosis is a common disease that affects 1 in 3 women and 1 in 12 men. Currently, there are an estimated 200 million women and 14 million men with osteoporosis or low bone mass worldwide [1]. The lifetime risk of fracture related to osteoporosis is nearly 40% at the age of 50. Osteoporosis and the resulting fractures represent a significant public health burden [2,3,4,5,6]. Healthy skeletal bone is continually remodeled (broken and subsequently replaced). Physiological bone remodeling is a highly coordinated process that is responsible for bone resorption and formation. This process is necessary to repair damaged bone and maintain mineral homeostasis. Approximately 5% of the bone surface is remodeled at any one time. This process is controlled by osteoblasts and osteoclasts. Osteoblasts generate new bone tissue, while osteoclasts break down bone tissue [7,8,9,10]. Bone remodeling is dependent on various hormones, including parathyroid hormone, calcitriol, calcitonin, estrogen, testosterone, growth hormone, insulin-like growth factor, thyroid hormone, and cortisol [11,12,13]. Dietary calcium and vitamin D are also important for bone health. Osteoporosis-related bone loss occurs when bone resorption prevails under bone formation.

Bisphosphonates are the most common drugs for the treatment of osteoporosis. They slow bone loss by decreasing bone resorption [14,15] and are administered both orally and intravenously. They are divided into two classes, the low potency non-nitrogen containing bisphosphonates and the potent nitrogen-containing bisphosphonates. The pharmacology of potent nitrogen-containing bisphosphonates is complex [16]. Their primary mechanism relays on the inhibition of the osteoclastic resorption of bone by both the promotion of the formation of the adenosine triphosphate analogue that induces apoptosis and the inhibition of farnesyl diphosphonate synthase. This inhibition results in the deregulation of intracellular transport, cytoskeletal organization and cell proliferation and results in the inhibition of osteoclast function [17,18].

Bisphosphonates bind to bone and are internalized by osteoclasts to inhibit resorption. They are incorporated into newly formed bone and can persist for several years through multiple cycles of bone resorption and deposition. Bisphosphonates have a half-life of more than 10 years. Treatment over 10 years would result in a cumulative effect. Extended treatment may be detrimental to bone health by preventing the cyclic changes required to maintain normal bone architecture [19,20,21]. Patients are continuously exposed to the pharmacological effects of the drug long after stopping the medication. Osteonecrosis of the jaw is a rare but recognized side effect resulting from long-term bisphosphonate use [22,23,24].

**Scheme 1:**
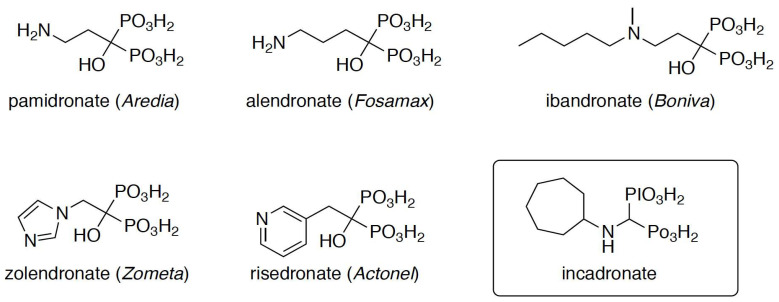
Structures of the bisphosphonates used in the treatment of osteoporosis.

The binding affinity of any bisphosphonate to hydroxyapatite will predict the likelihood of its attachment to bone and the duration of the attachment. Previous research revealed a complex network of interactions between these compounds and hydroxyapatite. These interactions involve phosphonic acid moieties, hydroxyl groups, and nitrogen moieties with a dependence on their position in an alkyl fragment or heterocyclic ring [17]. Hydroxyl groups are present in most commercialized drugs (Scheme 1) and enhance drug binding to bone. The removal of this group may result in weaker binding and a shorter persistence of the drug in bone [25]. Incadronate (Bisphonal), also known as cimadronate (Scheme 1), is an example of this type of drug that was applied for the treatment of malignancy-associated hypercalcemia in Japan [26,27,28].

In this paper, a series of *N*-alkyl- and *N*-cycloalkylaminomethylenebisphosphonates, which are considered as analogues of incadronate, have been synthesized and examined for their antiproliferative activity against a mouse macrophage J774E derived cell sub-line [29]. The most active compounds were used to heal fractures in sheep with steroid-induced osteoporosis [30]. These animals were chosen because of their similarity of sheep bones to human ones in regard to their architecture, anisotropy, bone and joint organization and mechanisms of bone regeneration [31,32]. The metabolic rate of sheep (based on oxygen consumption per gramme of body weight) is closer to that of man than a rat or dog model [33].

## 2. Results

### 2.1. Chemistry

**Scheme 2:**
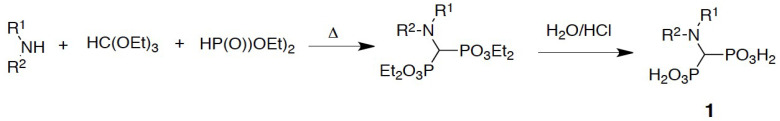
Three-component reaction applied to the synthesis of aminomethylenebisphosphonates.

The aminomethylenebisphosphonates were prepared by the classic three-component reaction between a primary or secondary amine, triethyl orthoformate, and diethyl phosphite, which was followed by acid hydrolysis of the obtained esters (Scheme 2) [34].

The structures of the final *N*-substituted aminomethylenebisphosphonic acids are presented with their in vitro activity toward mouse macrophage J774E in Table 1. This general procedure also resulted in a high yield of the model compound incadronate.

### 2.2. Influence of Mouse Macrophage J774E on Proliferation

The antiproliferative activity on in vitro cell cultures was examined to detect the antiosteoporotic activity of bisphosphonates. Mouse macrophage-like J774E cells, originating from the identical precursors as osteoclasts, were used. The choice of these line was prompted by the fact that murine macrophage cell line, J744E, is standardly used to initially screen the immunomodulatory activity of some natural compounds [35] and is standardly used as an osteoclast surrogate in the preliminary screening for antiosteoporotic compounds [29]. Cells, frozen in liquid nitrogen, are available from the bank of cell lines of the Polish Academy of Sciences in Wrocław. These cells were sensitive to aminomethylenebisphosphonates because of the induction of apoptosis [36,37]. (Table 1). This sensitivity allowed us to determine the effect of these compounds on J774E growth and to select the most active compounds for in vivo studies. The results are presented as IC_50_ values (the concentration of the tested agent that inhibits the proliferation of the cancer cell population by 50%).

The J774E culture was sensitive to the action of the studied aminomethylenebisphosphonates (Table 1). The most straightforward structure–activity relationship was obtained for linear alkanes with a bisphosphonate nitrogen atom substitution. These surfactant-like compounds (**4**–**11**) are strong inhibitors of J774E proliferation (an order of magnitude more potent than incadronate). The activity is independent of the hydrophobicity of the bisphosphonate. This finding is supported by the weaker activity of compounds **12**–**16**. These compounds are substituted at nitrogen by branched alkyl chains of a similar number of carbon atoms. The homologues of incadronate, compounds **17**, **18** and **20**, are equipped with the parent compound. The cyclopropyl derivative **17** is more than twice as active as the *n*-propyl bearing compound **2**.

Generally, the antiproliferative activity of analogues of incadronate is less dependent on the hydrophobicity of the *N* substituent than on its shape, although these differences are non-indicative.

### 2.3. In Vivo Evaluation of Compounds **4** and **5**

Based on the literature data, sheep have proven invaluable in osteoporosis research [38,39]. The mechanical properties of the macro- and microarchitecture of bone and the similar reaction to ovariectomy in sheep and humans are of high similarity, making ovariectomized sheep a suitable in vivo model. Moreover, osteoporosis induction in sheep is a simple and safe surgical approach, and sheep also have easy husbandry needs and a compliant nature.

The human and sheep skeleton are similar in regard to estrogen deficiency and specific therapeutic agents. The advantages related to sheep use are many: the skeleton size and mechanical features of are similar to humans: older sheep show Haversian bone remodeling, they are genetically more human-like than rodents, and sheep show sex hormone profiles comparable to women. Merino sheep have an almost continuous the estrous cycle, they are relatively easy to handle and maintain, and they present fewer ethical concerns than other non-human primates [40,41,42,43,44].

**Figure 1:**
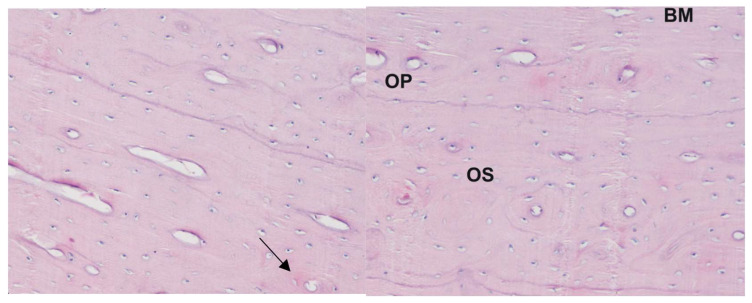
Histological picture of the corpus of the humerus bone from sheep with induced osteoporosis: staining with the Delafield method; magnification 400×. Numerous small osteons are visible containing 2–3 lamellae (arrow). The central canal is relatively large in respect to the osteon size; BM—interstitial lamellae, OP—primary osteon, OS—osteoresorption zone.

Osteoporosis was induced in female Merino sheep by a separately described regimen [45] (ovariectomy followed by controlled methylprednisolone treatment) [31,46,47,48,49]. The level of bone loss was determined by bone mineral density using quantitative computed tomography (QCT) (64-row DISCOVERY 690 CT scanner made by GE^®^, Waukesha, WI, USA) and X-ray microtomography (SkySkan 1172, 100kV, Bruker^®^, Kontich, Belgium). Forty days after the last steroid administration, a bone biopsy was performed. Bone biopsies were taken using diamond-tipped trephine needles that were 9 mm in diameter. For the procedure, the animals were premedicated with xylazine at a dose of 0.25 mg/kg body weight, into a vein. Bone biopsies were placed in physiological fluid in tubes and transported for micro-CT examination. An ultrastructural and histological analysis of bone slides revealed that the described method of induction of osteoporosis in sheep was successful (see Figure 1 for representative examples).

Compounds **4** and **5** were tested on female sheep with induced osteoporosis. They were chosen taking into consideration their high activity in vitro (higher than incadronate) and aqueous solubility suitable for drug formulation. Additionally, compound **4** is a formal isomer of incadronate.

**Figure 2:**
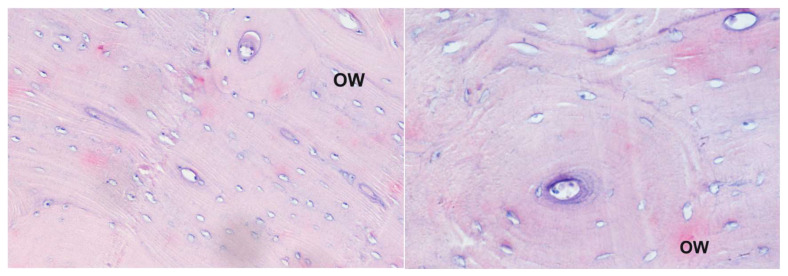
Histological picture of different samples of the corpus of the humerus bone from animals receiving compound **4**: staining with the Delafield method; magnification 200× (**left panel**) and 400× (**right panel**). Large secondary osteons indicate progress in bone tissue healing; OW—secondary osteon.

**Figure 3:**
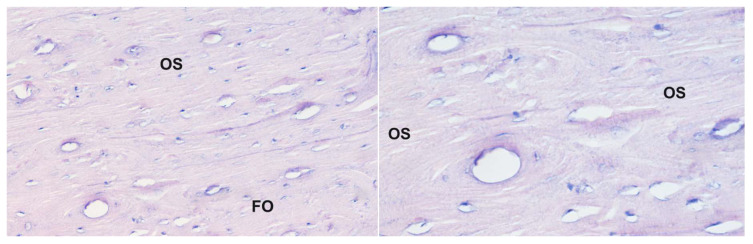
Histological picture of the same sample of the corpus of the humerus bone from sheep receiving bisphosphonate **5**: staining with the Delafield method; magnification 200× (**left panel**) and 400× (**right panel**). A large increase in the development multilamellar osteons with a relatively large canal size is observed; OS—osteoresorption zone, FO—forming osteon.

Bisphosphonate administration began after three months after ovariectomy. An aqueous solution of sodium salt of bisphosphonate (35 mg/sheep) was administered weekly, using a probe directly into the rumen. The first three doses were 150 mg per sheep, while the next six were 300 mg per sheep. In total, nine doses were administered to each sheep. The animals were then sacrificed (30 days after bisphosphonates therapy), and the bone samples from the mandible, humerus, radius, ulna, femur, tibia and pelvis were collected for histology. The activity experiments of compounds **4** and **5** were performed independently at time intervals. Mild antiosteoporotic activity of compounds **4** and **5** was observed from the induction of repair processes, but the speed of recovery was not satisfactory as indicated by preliminary histopathological examination (Figure 2 and Figure 3) as well as histomorphometry analysis. Anyway, despite the short period of healing, some positive effect were observed; however, it might be not assumed that repair processes dominate when using these two bisphosphonates. This seems to disqualify them as drug candidates.

Furthermore, sheep medication with compounds **4** and **5** was accompanied by severe side effects. Animals lost their fleece, and some weight loss was observed during the initial treatment period despite the fact that no significant deviations from the biochemical norms and hormonal markers were observed on blood tests.

Sheep treated with compound **4** suffered caseous lymphadenitis in the late phase of the medication, resulting in outcomes ranging from skin injury to animal death. This result may be attributed to the decreased immunological response to *Corynebacterium pseudotuberculosis*, which is the causal agent of this infectious disease [50,51]. Although it is well known that bisphosphonates interfere with immunological system [52], this is a surprising finding since data from the literature indicate that it is a common osteoporosis treatment that rather boosts immunologic response [53].

## 3. Discussion

A series of simple analogues of incadronate have been obtained and screened for their antiproliferative activity toward macrophage-like J774E cells. This in vitro antiproliferative test relied on the induction of cell apoptosis. Most of the analogues appeared to be active in this test. Unexpectedly, it was found that the hydrophobicity of the *N*-substituents does not influence their activity. Although in vitro studies did not provide a clear indication for selection of the most active compounds, two of them were chosen for further evaluation in the treatment of induced osteoporosis in sheep.

In vitro studies confirmed not satisfactory, mild therapeutic effects of compounds **4** and **5**, and thus they are not suitable for consideration as potential drugs. Therefore, the search for novel antiosteoporotic bisphosphonates within this group of compounds should consider the introduction of functional groups, improving their solubility and/or modifying their binding to bone hydroxyapatite. Furthermore, the observed side effects and induction of caseous lymphadenitis by the compound suggest that the search for novel drugs in this class of compounds should be treated with some reservation.

## 4. Materials and Methods

### 4.1. General Information

All solvents and reagents that were purchased from commercial suppliers were of analytical grade and used without further purification. Unless otherwise specified, the solvents were removed with a rotary evaporator. The ^1^H-, ^31^P-, and ^13^C-NMR spectroscopic experiments were performed on a Bruker Avance II Ultrashield Plus (Bruker, Rheinstetten, Germany) operating at 600.58 MHz (^1^H), 243.12 MHz (^31^P{^1^H}), and 151.016 MHz (^13^C) and a JEOL JNM-ECZ 400S Research FT NMR Spectrometer (JEOL Ltd., Tokyo, Japan) operating at 399.78 MHz (^1^H), 161.83 MHz (^31^P{^1^H}), and 100.53 (^13^C). Measurements were made in D_2_O, and solutions of D_2_O + NaOD at 300K, and all the solvents were supplied by Merck Life Science (Darmstadt, Germany). The chemical shifts are reported in ppm relative to TMS and 85% H_3_PO_4_, used as external standards, and the coupling constants are reported in Hz. The melting points were determined on an SRS Melting Point Apparatus OptiMelt MPA 100 (Stanford Research Systems, Sunnyvale, CA, USA) and are reported uncorrected. The purity of all test compounds was higher than 95% by ^1^H NMR and LC-MS. The mass spectra were recorded at the Faculty of Chemistry, Wroclaw University of Science and Technology, using a Waters Aquity UPLC and Waters Xevo G3 QTof mass spectrometer (method of electrospray ionization, ESI) (Waters, Milford, MA, USA).

### 4.2. Chemistry—Synthetic Procedure

A mixture of amine (0.03 mol), triethyl phosphite (0.062 mol) and triethyl orthoformate (0.031 mol) was heated (120 °C) for 8–14 h with constant stirring a Radleys Carousel 6 Plus parallel synthesis reaction station (Saffron Walden, Essex, United Kingdom). The volatile components of the reaction mixture were evaporated, and the resulting crude product as oil was hydrolyzed with 6 N hydrochloric acid (20 mL) by refluxing for 12 h. The crude bisphosphonic acids were obtained by the evaporation of solvents (oils) and further purified by crystallization from hot water or a hot water–ethanol (80/20) mixture. In some cases, during hot crystallization, activated carbon was used to decolorize the solutions, which were filtered off under reduced pressure. Crystal growth was observed after the solution was cool. In the case of acids **6**–**11**, upon evaporation of the aqueous hydrochloric acid solution, the product was observed as solid. In this case, the resulting crude bisphosphonic acid was not crystallized because of its complete insolubility in water or alcohol. These products were washed with water.

Aminomethylenebisphosphonic acid (**1**) was obtained from methylbenzylamine as a white solid (85% yield from reaction with *p*-aminobenzylamine); m.p. 258–260 °C; ^31^P NMR (243.12 MHz, D_2_O + NaOD, ppm): δ = 18.21; ^1^H NMR (600.58 MHz, D_2_O + NaOD, ppm) δ: 2.45 (1H, t, *J* = 17.4 Hz, CHP); ^13^C NMR (151.02 MHz, D_2_O + NaOD, ppm) δ: 50.80 (t, *J* = 127.9 Hz, CP_2_); HRMS (TOF MS ESI^−^): [M − H]^−^ calcd for CH_7_NO_6_P_2_: 189.9670, found: 189.9671.

*N*-(n-Propylamino)methylenebisphosphonic acid (**2**) was obtained with a 43% yield; mp. 225–227 °C; ^31^P NMR (242.97 MHz, D_2_O + NaOD, ppm): δ = 18.08; ^1^H NMR (600.58 MHz, D_2_O + NaOD, ppm) δ: 0.52 (t, *J* = 7.42 Hz, 2H); 1.08 (dd, *J*_1_ = 7.41 Hz, *J*_2_ = 14.79, 1H), 2.18 (t, *J* = 17.08 Hz, 1H, CHP), 2.35 (t, *J* = 7.38 Hz, 1H); ^13^C NMR (151.02 MHz, D_2_O + NaOD, ppm) δ: 11.23, 22.53, 52.81, 59.33 (t, *J* = 129.27 Hz, CP_2_); HRMS (TOF MS ESI^−^): [M − H]^−^ calcd for C_4_H_13_NO_6_P_2_: 232.0140, found: 232.0142.

*N*-(n-Pentylamino)methylenebisphosphonic acid (**3**) was obtained with a 61% yield; mp. 200 °C; ^31^P NMR (242.93 MHz, D_2_O + NaOD) δ: 17.63 ppm; ^1^H NMR (600.58 MHz, D_2_O + NaOD, ppm) δ: (t, *J* = 7.38 Hz, 3H), 0.67–0.71 (m, 3H), 1.08–1.18 (m, 2H),1.34–1.44 (m, 1H), 2.39 (t, *J* = 17.56 Hz, 1H, CHP), 2.62 (t, *J* = 5.64 Hz, 2H); ^13^C NMR (150.91 MHz, D_2_O + NaOD) δ: 22.14, 25.70, 38.57, 49.53, 49.58, 59.80 (t, *J* = 128.87 Hz, CP_2_); HRMS (TOF MS ESI^−^): [M − H]^−^ calcd for C_6_H_17_NO_6_P_2_: 260.0453, found: 232.0452.

*N*-(n-Heptylamino)methylenebisphosphonic acid (**4**) was obtained with a 72% yield; mp. 212–213 °C; ^31^P NMR (242.93 MHz, D_2_O + NaOD) δ: 14.91 ppm; ^1^H NMR (600.58 MHz, D_2_O + NaOD, ppm) δ: 0.76 (t, *J* = 6.89 Hz, 3H), 1.15–1.20 (m, 4H); 1.21–1.27 (m, 4H), 1.43 (dt, *J*_1_ = 7.25 Hz, *J*_2_ = 14.17, 2H), 2.60 (t, *J* = 17.30 Hz, 1H, CHP), 2.84 (t, *J* = 7.53 Hz, 2H); ^13^C NMR (150.91 MHz, D_2_O + NaOD) δ: 13.43, 21.98, 26.39, 28.46, 28.70, 31.01, 50.99, 59.46 (t, *J* = 125.32 HZ, CP_2_); HRMS (TOF MS ESI^−^): [M − H]^−^ calcd for C_8_H_21_NO_6_P_2_: 288.0766, found 288.0765.

*N*-(n-Octylamino)methylenebisphosphonic acid (**5**) was obtained with a 67% yield; mp. 202 °C; ^31^P NMR (242.93 MHz, D_2_O + NaOD) δ: 18.07 ppm; ^1^H NMR (600.58 MHz, D_2_O + NaOD, ppm) δ: 0.49 (t, *J* = 6.79 Hz, 3H), 0.85–0.96 (m, 10H), 1.04–1.1.10 (m, 2H), 2.18 (t, *J* = 17.05 Hz, 1H, CHP), 2.38 (7, *J* = 7.48 Hz, 2H); ^13^C NMR (150.91 MHz, D_2_O + NaOD) δ: 13.43, 21.91, 26.65, 28.34, 28.79, 29.59, 31.02, 51.32, 59.65 (t, *J* = 132.10 Hz, CP_2_); HRMS (TOF MS ESI^−^): [M − H]^−^ calcd for C_9_H_23_NO_6_P_2_: 302.0922, found 302.0927.

*N*-(n-Nonylamino)methylenebisphosphonic acid (**6**) was obtained with a 85% yield; mp. 279–280 °C; ^31^P NMR (242.93 MHz, solid) δ: 13.40 ppm; ^13^C NMR (150.91 MHz, solid) δ: 20.01, 29.13, 32.97, 35.45, 36.14, 36.64, 37.01, 39.33, 56.77, 60.73 (t, *J* = 120.03 Hz, CP_2_); HRMS (TOF MS ESI^−^): [M − H]^−^ calcd for C_10_H_25_NO_6_P_2_: 316.1079, found 316.1063.

*N*-(n-Decylamino)methylenebisphosphonic acid (**7**) was obtained with a 90% yield; mp. 200 °C; ^31^P NMR (242.93 MHz, solid) δ: 12.44 ppm; ^13^C NMR (150.91 MHz, solid) δ: 19.83, 29.96, 30.65, 32.39 (d, *J* = 55.29 Hz), 35.91, 36.80, 38.03, 38.68, 39.31, 53.48, 59.86 (d, *J* = 104.73 Hz, **C**P), 60.95 (d, *J* = 104.73 Hz, CP_2_).

*N*-(n-Undecylamino)methylenebisphosphonic acid (**8**) was obtained with a 86% yield; mp. 205 °C; ^31^P NMR (242.93 MHz, solid) δ: 9.29 ppm; ^13^C NMR (150.91 MHz, solid) δ: 17.08, 26.94, 27.68, 29.12, 29.90, 32.38, 34.30, 35.19, 37.24, 54.13, 578.49 (t, *J* = 122.22 Hz, CP_2_).

*N*-(n-Dodecylamino)methylenebisphosphonic acid (**9**) was obtained as a white solid with a 89% yield; mp. > 300 °C; ^31^P NMR (242.93, solid) δ = 10.15; ^13^C NMR (150.91 MHz, solid) δ: 19.73, 29.90, 32.30, 33.47, 34.74, 36.53, 37.50, 37.79, 38.59, 39.55, 40.35, 57.19, 60.66 (t, *J* = 106.40 Hz, CP_2_); HRMS (TOF MS ESI^−^): [M − H]^−^ calcd for C_13_H_31_NO_6_P_2_: 358.1549, found 358.1408.

*N*-(n-Tridecylamino)methylenebisphosphonic acid (**10**) was obtained with a 52% yield; mp. > 300 °C; ^31^P NMR (242.93 MHz, solid) δ: 5.69 ppm; ^13^C NMR (150.91 MHz, solid) δ: 20.09, 30.15, 30.97, 32.23, 33.00, 33.56, 35.43, 37.41, 38.16, 38.59, 39.12, 40.22, 40.42, 57.25, 61.05 (dd, *J* = 112.49 Hz, CP_2_).

*N*-(n-Tetradecylamino)methylenebisphosphonic acid (**11**) was obtained with a 49% yield; mp. > 300 °C; ^31^P NMR (242.93 MHz, solid) δ: 18.99 ppm; ^13^C NMR (150.91 MHz, solid) δ: 19.01, 27.22, 31.99, 33.74, 36.31, 36.72, 37.25, 56.65, 60.64 (t, *J* = 111.40 Hz, CP_2_).

*N*-(Isopropylamino)methylenebisphosphonic acid (**12**) was obtained with a 49% yield; mp. 225–227 °C; ^31^P NMR (243.12 MHz D_2_O + NaOD) δ: 17.99 ppm; ^1^H NMR (600.58 MHz, D_2_O + NaOD, ppm) δ: 0.77 (d, *J* = 6.29 Hz, 6H); 2.54 (t, *J* = 17.56 Hz, 1H, CHP), 2.93–2.99 (m, 1H); ^13^C NMR (150.91 MHz,) δ: 21.78, 47.16, 55.26 (t, *J* = 129.80 Hz, CP_2_); HRMS (TOF MS ESI^−^): [M − H]^−^ Calcd for C_4_H_13_NO_6_P_2_: 232.0140, found: 232.0143.

*N*-(Isobutylamino)methylenebisphosphonic acid (**13**) was obtained with a 58% yield; mp. 215–216 °C; ^31^P NMR{^1^H} (243.12 MHz D_2_O + NaOD) δ: 7.92 and 8.02 ppm; ^1^H NMR (600.58 MHz, D_2_O + NaOD, ppm) δ: 0.93 (d, *J* = 6.72 Hz, 6H); 1.97 (dt, *J*_1_ = 6.86 Hz, *J*_2_ = 13.73 Hz, 1H), 3.12 (d, *J* = 7.18 Hz, 1H), 3.26 (t, *J* = 17.22 Hz, 1H, CHP); ^13^C NMR (150.91 MHz, D_2_O + NaOD) δ: 19.39, 20.17, 27.53, 58.90 (t, *J* = 246.01 Hz, CP_2_); HRMS (TOF MS ESI^−^): [M − H]^−^ calcd for C_5_H_15_NO_6_P_2_: 246.0296, found 246.0289.

*N*-(Isopentylamino)methylenebisphosphonic acid (**14**) was obtained as a white solid with a 46% yield; mp. 185–187 °C; ^31^P NMR (243.12 MHz D_2_O + NaOD) δ: 18.18 ppm; ^1^H NMR (600.58 MHz, D_2_O + NaOD, ppm) δ: 0.42 (d, *J* = 6.61 Hz, 6H); 0.87 (dd, *J*_1_ *=* 7.21 Hz, *J*_2_ *=* 14.85 Hz, 2H), 1.13 (dt, *J*_1_ *=* 6.64, *J*_2_ *=* 13.30 Hz, 1H), 2.08 (t, *J* = 16.86 Hz, 1H, CHP), 2.31 (t, *J* = 7.60 Hz, 2H); ^13^C NMR (150.91 MHz, D_2_O + NaOD) δ: 22.21, 25.45, 38.61, 49.20, 59.48 (t, *J* = 129.14 Hz, CP_2_); HRMS (TOF MS ESI^−^): [M − H]^−^ calcd for C_6_H_17_NO_6_P_2_: 260.0453, found 260.0465.

*N*-(Isohexylamino)methylenebisphosphonic acid (**15**) was obtained with a 41% yield; mp. 193–195 °C; ^31^P NMR (243.12 MHz D_2_O + NaOD) δ: 17.89 ppm; ^1^H NMR (600.58 MHz, D_2_O + NaOD, ppm) δ: 0.96 (d, *J* = 10.35 Hz, 3H), 1.08 (d, *J* = 10.91 Hz, 3H), 1.35–1.46 (m, 1H), 1.47–1.61 (m, 2H), 1.78–1.94 (m, 2H), 2.77 (t, *J* = 17.26 Hz, 1H, CHP), 2.87–3.16 (m, 2H); HRMS (TOF MS ESI^−^): [M − H]^−^ calcd for C_7_H_19_NO_6_P_2_: 274.0609, found: 274. 0620 and [M − CH_3_]^−^ 260.0448.

*N*-2-(6-Methylhexylamino)methylenebisphosphonic acid (**16**) was obtained with a 34% yield; mp. 244–24 °C; ^31^P NMR{^1^H} (243.12 MHz D_2_O + NaOD) δ: 17.74 and 17.87 ppm; ^1^H NMR (600.58 MHz, D_2_O + NaOD, ppm) δ: 0.65 (dd, *J*_1_ = 2.26 Hz, *J*_2_ = 6.63, 6H), 0.82 (d, *J* = 6.32 Hz, 3H), 0.91–1.01 (m, 3H), 1.02–1.15 (m, 2H), 1.29–1.41 (m, 2H), 2.62 (t, *J* = 17.60 Hz, 1H, CHP), 2.87–2.92 (m, 1H); ^13^C NMR (150.91 MHz, D_2_O + NaOD) δ: 18.41, 21.92, 22.12, 23.17, 27.20, 36.28, 38.78, 52.01, 55.21 (t, *J* = 125.83 Hz, CP_2_); HRMS (TOF MS ESI^−^): calcd for C_9_H_23_NO_6_P_2_: 302.0922, found: 302.0297 [M − H]^−^; calcd: 605.1922, found: 605.1945 [2M − H]^−^; calcd: 908.2922, found: 908.2953 [3M − H]^−^.

*N*-(2,5-Dimethylhexylamino)methylenebisphosphonic acid (**17**) was obtained with a 31% yield; mp. 231–234 °C; ^31^P NMR{^1^H} (243.12 MHz D_2_O + NaOD) δ: 18.03 and 18.15 ppm ^1^H NMR (600.58 MHz, D_2_O + NaOD, ppm) δ: 0.59 (d, *J* = 6.59 Hz, 6H); 0.76 (d, *J* = 6.24 Hz, 3H), 0.87–0.93 (m, 3H), 0.97–1.06 (m, 2H), 1.23–1.32 (m, 2H), 2.56 (t, *J* = 17.57 Hz, 1H, CHP), 2.78–2.86 (m, 1H); ^13^C NMR (150.91 MHz, D_2_O + NaOD) δ: 18.53, 21.97, 22.16, 23.18, 27.22, 36.36, 51.96, 55.26 (t, *J* = 128.98 Hz, CP_2_); HRMS (TOF MS ESI^−^): [M − H]^−^ calcd for C_9_H_23_NO_6_P_2_: 302.0922, found 302.0927.

*N*-(Cyclopropylo)methylenebisphosphonic acid (**18**) was obtained with a 56% yield; mp. 224–226 °C; ^31^P NMR (243.12 MHz D_2_O + NaOD) δ: 8.07 ppm ^1^H NMR (600.58 MHz, D_2_O + NaOD, ppm) with amine δ: 1.44–1.56 (m, 1H), 1.63–1.79 (m, 2H), 2.97 (t, *J* = 18.72 Hz, 1H, CHP), 3.48 (t, *J* = 5.13 Hz, 2H); ^13^C NMR (150.91 MHz, D_2_O + NaOD) δ: 21.26, 24.90, 53.66, 67.03 (t, *J* = 114.06 Hz, CP_2_); HRMS (TOF MS ESI^−^): [M − H]^−^ calcd for C_4_H_11_NO_6_P_2_: 229.9983, found 229.8729.

*N*-(Cyclopentylo)methylenebisphosphonic acid (**19**) was obtained with a 66% yield; mp. 210–211 °C; ^31^P NMR (243.12 MHz D_2_O + NaOD) δ: 17.70 ppm; ^1^H NMR (600.58 MHz, D_2_O + NaOD, ppm) δ: 0.67 (t, *J* = 7.36 Hz, 3H), 1.06–1.13 (m, 2H), 1.21 (dt, *J* = 7.39, *J* = 14.98, 2H), 2.35 (t, *J* = 17.34 Hz, 1H, CHP), 2.55 (t, *J* = 7.43 Hz, 2H); ^13^C NMR (150.91 MHz, D_2_O + NaOD) δ: 13.54, 19.97, 31.73, 50.89, 50.92, 59.70 (t, *J* = 129.41 Hz, CP_2_);HRMS (TOF MS ESI^−^): [M − H]^−^ calcd for C_6_H_15_NO_6_P_2_: 258.0296, found 258.0286.

*N*-(Cyclohexylamino)methylenebisphosphonic acid (**20**) was obtained with a 42% yield; mp. 214–215 °C; ^31^P NMR (243.12 MHz D_2_O + NaOD) δ: 14.76 ppm; ^1^H NMR (600.58 MHz, D_2_O + NaOD, ppm) δ: 0.97–1.09 (m, 3H), 1.12–1.21 (m, 2H), 1.49 (d, *J* = 12.64 Hz, 1H), 1.62 (d, *J* = 13.30 Hz, 2H),1.93 (d, *J* = 9.98 Hz, 2H), 2.87 (d, *J* = 17.23 Hz, 2H, CHP), 3.01–3.05 (m, 1H); ^13^C NMR (150.91 MHz, D_2_O + NaOD) δ: 13.54, 19.97, 31.73, 50.92, 59.70 (t, *J* = 129.41 Hz, CP_2_); HRMS (TOF MS ESI^−^): [M − H]^−^ calcd for C_7_H_17_NO_6_P_2_: 272.0453, found 272.0620.

*N*-(Cycloheptylamino)methylenebisphosphonic acid (incadronate) (**21**) was obtained with a 63% yield; mp. 225 °C; ^31^P NMR (243.12 MHz D_2_O + NaOD) δ: 8.07 ppm; ^1^H NMR (600.58 MHz, D_2_O + NaOD, ppm) δ: 1.46–1.63 (m, 6H), 1.66–1.81 (m, 4H), 2.11–2.19 (m, 2H), 3.59 (t, *J* = 18.23 Hz, 1H, CHP), 3.67–3.75 (m, 1H), ^13^C NMR (150.91 MHz, D_2_O + NaOD) δ: 24.49, 25.42, 31.46, 54.71 (t, *J* = 124.27 Hz, CP_2_), 56.15; HRMS (TOF MS ESI^−^): [M − H]^−^ calcd for C_8_H_19_NO_6_P_2_: 286.0609, found 286.0597.

*N*-(Cyclooctylamino)methylenebisphosphonic acid (**22**) was obtained with a 54% yield; mp. 247–248 °C; ^31^P NMR (243.12 MHz D_2_O + NaOD) δ: 18.45 ppm; ^1^H NMR (600.58 MHz, D_2_O + NaOD, ppm) δ: 1.02–1.10 (m, 7H), 1.20–1.26 (m, 3H), 1.28–1.31 (m, 2H), 1.46–1.50 (m, 2H), 2.42 (t, *J* = 17.40 Hz, 1H, CHP), 2.79 (dt, *J*_1_ = 3.64 Hz, *J*_2_ = 17.40 Hz, 1H); ^13^C NMR (150.91 MHz, D_2_O + NaOD) δ: 23.57, 25.19, 36.97, 31.01, 55.10 (t, *J* = 129.38 Hz, **C**P_2_), 56.03; HRMS (TOF MS ESI^−^): [M − H]^−^ calcd for C_9_H_21_NO_6_P_2_: 300.0766, found 300.0768.

*N,N*-(Di-n-propylamino)methylenebisphosphonic acid (**23**) was obtained with a 35% yield; mp. 206–208 °C; ^31^P NMR (243.12 MHz D_2_O + NaOD) δ: 17.31 ppm; ^1^H NMR (600.58 MHz, D_2_O + NaOD, ppm): δ: 0.71 (t, *J* = 7.37 Hz, 6H), 1.25–1.34 (m, 4H), 2.43 (t, *J* = 17.57 Hz, 1H, CHP), 2.47–2.62 (m, 4H); ^13^C NMR (150.91 MHz, D_2_O + NaOD) δ: 11.06, 11.10, 22.40, 22.48, 52.85, 52.91, 59.43 (t, *J* = 114.06Hz, CP_2_); HRMS (TOF MS ESI^−^): [M − H]^−^ calcd for C_7_H_19_NO_6_P_2_ 274.0610, found 274.0618.

*N,N*-(Di-n-cyclohexylamino)methylenebisphosphonic acid (**24**) was obtained with a 46% yield; mp. 209–211 °C; ^31^P NMR (242.93 MHz, D_2_O + NaOD) δ: 17.18 ppm; ^1^H NMR (600.58 MHz, D_2_O + NaOD, ppm) δ: 0.97–1.04 (m, 6H), 1.20 (q, *J* = 12.50 Hz, 4H), 1.37 (q, *J* = 11.19 Hz, 4H), 1.45 (d, *J* = 12.82 Hz, 2H), 1.63 (d, *J* = 12.99 Hz, 4H), 3.32 (t, *J* = 20.36 Hz, 1H, CHP), 3.47 (q, *J* = 7.05 Hz, 2H); ^13^C NMR (150.91 MHz, D_2_O + NaOD) δ: 24.46, 25.37, 31.23 (d, *J* = 35.59 Hz), 54, 67 (t, *J* = 123.97 Hz, CP_2_), 56.18; HRMS (TOF MS ESI^−^): [M − H]^−^ calcd for C_13_H_27_NO_6_P_2_: 387.1239, found 387.1232.

*N*-Ethyl-*N*-(cyclohexylamino)methylenebisphosphonic acid (**25**) was obtained with a 40% yield; mp. 199–201 °C; ^31^P NMR (242.93 MHz, D_2_O + NaOD) δ: 9.89 ppm; ^1^H NMR (600.58 MHz, D_2_O + NaOD, ppm) δ: 0.86–0.92 (m, 1H); 0.93–0.97 (m, 2H), 0.97 (t, *J* = 7.24 Hz, 3H), 1.05 (dt, *J*_1_ = 9.95, *J*_2_- = 12.00 Hz, 2H), 1.27 (d, *J* = 12.93 Hz, 1H), 1.47 (d, *J* = 13.19 Hz, 2H), 1.89 (d, *J* = 10.75 Hz, 2H), 3.05 (t, *J* = 19.70 Hz, 1H, CHP), 3.19 (q, *J* = 7.21 Hz, 2H), 3.30 (dt, *J*_1_ = 3.08 Hz, *J*_2_ = 11.33 Hz, 1H), ^13^C NMR (150.91 MHz, D_2_O + NaOD) δ: 13.88, 24.64, 24.71, 29.53, 46.28, 61.33 (t, *J* = 115.08 HZ, CP_2_)_,_ 64.54; HRMS (TOF MS ESI^−^): [M − H]^−^ calcd for C_9_H_21_NO_6_P_2_: 300.0766, found 300.0768.

*N*-(Pyrrolidylamino)methylenebisphosphonic acid (**26**) was obtained with a 21% yield; mp. 149–151 °C; ^31^P NMR (242.93 MHz, D_2_O + NaOD) δ: 11.12 ppm; ^1^H NMR (600.58 MHz, D_2_O + NaOD, ppm) δ: 1.74–1.84 (m, 4H), 3.13 (t, *J* = 17.42 Hz, 1H, CHP), 3.30–3.42 (m, 4H); ^13^C NMR (150.91 MHz, D_2_O + NaOD) δ: 22.96, 52.32, 62.02 (t, *J* = 169.03 HZ, CP_2_); HRMS (TOF MS ESI^−^): [M − H]^−^ calcd for C_5_H_13_NO_6_P_2_: 244.0140, found 244.0138.

*N*-(Piperidylamino)methylenebisphosphonic acid (**27**) was obtained with a 61% yield; mp. 241–244 °C; ^31^P NMR (242.93 MHz, D_2_O + NaOD) δ: 8.07 ppm; ^1^H NMR (600.58 MHz, D_2_O + NaOD, ppm) δ: 1.44–1.56 (m, 2H); 1.63–1.80 (m, 4H), 2.97 (t, *J* = 18.72 Hz, 1H, CHP), 3.48 (t, *J* = 5.12 Hz, 4H); ^13^C NMR (150.91 MHz, D_2_O + NaOD) δ: 21.26, 24.90, 53.66, 67.03 (t, *J* = 141.06 HZ, **C**P_2_); HRMS (TOF MS ESI^−^): [M − H]^−^ calcd for C_6_H_15_NO_6_P_2_: 258.0296, 516.0592 [2M − H]^−,^ found 258.0286, 515.9357 [2M − H]^−^.

*N*-(Azepanylamino)methylenebisphosphonic acid (**28**) was obtained with an 85% yield; mp. 235–236 °C; ^31^P NMR (243.12 MHz D_2_O + NaOD) δ: 8.65 ppm; ^1^H NMR (600.58 MHz, D_2_O + NaOD, ppm) δ: 1.47–1.55 (m, 4H), 1.67–1.75 (m, 4H), 2.98 (t, *J* = 18.64 Hz, 1H, CHP), 3.46 (t, *J* = 5.34, 4H); HRMS (TOF MS ESI^−^): [M − H]^−^ calcd for C_7_H_17_NO_6_P_2_: 272.0453, found: 272.0463.

*N-(Azocinylamino)methylenebisphosphonic acid* (**29**) was obtained with a 44% yield; mp. 230–231 °C; ^31^P NMR (243.12 MHz D_2_O + NaOD) δ: 8.03 ppm; ^1^H NMR (600.58 MHz, D_2_O + NaOD, ppm) δ: 0.49–1.50 (m, 2H), 1.54–1.62 (m, 4H), 1.71–1.80 (m, 4H), 3.04 (t, *J* = 18.68 Hz, 1H, CHP), 3.49 (t, *J* = 5.76, 4H); HRMS (TOF MS ESI^−^): [M − H]^−^ calcd for C_8_H_19_NO_6_P_2_: 286.0610, found: 286.0642.

*N*-(4-Methypiperidylamino)methylenebisphosphonic acid (**30**) was obtained with a 69% yield; mp. 225–228 °C; ^31^P NMR (243.12 MHz D_2_O + NaOD) δ: 17.05 ppm; ^1^H NMR (600.58 MHz, D_2_O + NaOD, ppm) δ: 0.40 (d, *J* = 6.04 Hz, 6H), 1.29–1.38 (m, 3H), 0.58–0.65 (m, 2H), 0.83–0.94 (m, 2H), 1.11 (d, *J* = 11.80 Hz, 2H), 2.34 (t, *J* = 21.44 Hz, 1H, CHP), 2.53 (d, *J* = 7.30 Hz 4H); ^13^C NMR (150.91 MHz, D_2_O + NaOD) δ: 21.46, 29.21, 34.23, 51.13, 66.68 (t, *J* = 121.03 Hz, CP_2_); HRMS (TOF MS ESI^−^): [M − H]^−^ calcd for C_6_H_15_NO_6_P_2_: 258.0296, found 258.0286.

*N*-(2,6-Dimethylpiperidylamino)methylenebisphosphonic acid (**31**) was obtained with a 49% yield; mp. 225–228 °C; ^31^P NMR (243.12 MHz D_2_O + NaOD) δ: 7.93 ppm; ^1^H NMR (600.58 MHz, D_2_O + NaOD, ppm) δ: 1.27 (d, *J* = 6.04 Hz, 6H), 1.29–1.38 (m, 3H), 1.41–1.49 (m, 1H), 1.75 (d, *J* = 13.49 Hz, 2H), 3.52 (t, *J* = 23.52 Hz, 1H, CHP), 3.57–3.61 (m, 2H); ^13^C NMR (150.91 MHz, D_2_O + NaOD) δ: 20.37, 21.01, 33.18, 59.90 (d, *J* = 4.20 H), 61.77 (t, *J* = 113.43 Hz, CP_2_); HRMS (TOF MS ESI^−^): [M − H]^−^ calcd for C_8_H_19_NO_6_P_2_: 286.0610, found 286.0619.

*N*-(3,5-Dimethylpiperidylamino)methylenebisphosphonic acid (**32**) was obtained with a 57% yield; mp. 229–230 °C; ^31^P NMR (243.12 MHz D_2_O + NaOD) δ: 17.14 ppm; ^1^H NMR (600.58 MHz, D_2_O + NaOD, ppm) δ: 0.67 (d, *J* = 6.17 Hz, 6H), 2.28 (t, *J* = 21.52 Hz, 1H, CHP), 2.33 (t, *J* = 11.05 Hz, 2H), 2.51 (d, *J* = 11.305 Hz, 2H) 3.19–3.30 (m, 2H); ^13^C NMR (150.91 MHz, D_2_O + NaOD) δ: 18.19, 56.70, 65.84 (t, *J* = 122.22 Hz, CP_2_), 72.86; HRMS (TOF MS ESI^−^): [M − H]^−^ calcd for C_8_H_19_NO_6_P_2_: 286.0610, found 286.0619.

*N*-(2-Ethylpiperidylamino)methylenebisphosphonic acid (**33**) was obtained with a 42% yield; mp. 205–207 °C; ^31^P NMR (243.12 MHz D_2_O + NaOD) δ: 10.41 ppm; ^1^H NMR (600.58 MHz, D_2_O + NaOD, ppm) δ: 0.68 (t, *J* = 6.63 Hz, 3H), 1.20–1.29 (m, 2H), 1.33–1.50 (m, 2H), 1.52–1.62 (m, 2H), 1.69 (dd, *J*_1_ = 11.10 Hz, 2H), 3.17–3.29 (m, 1H), 3.30 (t *J* = 19.56 Hz, 1H, CHP), 3.48–3.60 (m, 2H); ^13^C NMR (150.91 MHz, D_2_O + NaOD) δ: 7.46, 22.32, 23.54, 24.78, 29.24, 58.84 (t, *J* = 114.66 Hz, CP_2_), 62.99; HRMS (TOF MS ESI^−^): [M − H]^−^ calcd for C_8_H_19_NO_6_P_2_: 286.0609, found 286.0619.

*N*-(Morpholino)methylenebisphosphonic acid (**34**) was obtained with a 64% yield; mp. 248 °C; ^31^P NMR (243.12 MHz D_2_O + NaOD) δ: 16.16 ppm; ^1^H NMR (600.58 MHz, D_2_O + NaOD, ppm) δ: 2.71 (t, *J* = 22.09 Hz, 1H, CHP), 3.00 (t, *J* = 4.20 Hz, 2H), 3.61 (t, *J* = 4.50 Hz, 2H); ^13^C NMR (150.91 MHz, D_2_O + NaOD) δ: 51.12, 66.67 (t, *J* = 123.30 Hz, CP_2_), 67.58; HRMS (TOF MS ESI^−^): [M − H]^−^ calcd for C_5_H_13_NO_7_P_2_: 260.0089, found 260.0081.

*Cis*– 2,6-dimethyl-*N*-(morpholino)methylenebisphosphonic acid (**35**) was obtained with a 59% yield; mp. 222 °C; ^31^P NMR (243.12 MHz D_2_O + NaOD) δ: 17.14 ppm; ^1^H NMR (600.58 MHz, D_2_O + NaOD, ppm) δ: 0.67 (d, *J* = 6.17 Hz, 2H), 2.30 (t, *J* = 21.52 Hz, 1H, CHP), 2.33 (t, *J* = 11.05 Hz, 2H) 3.19–3.30 (m, 2H); ^13^C NMR (150.91 MHz, D_2_O + NaOD) δ: 18.19, 56.70, 65.84 (t, *J* = 122.22 Hz, CP_2_), 72.86; HRMS (TOF MS ESI^−^): [M − H]^−^ calcd for C_7_H_17_NO_7_P_2_: 288.0402, found 288.0405.

### 4.3. Antiproliferative Activity

#### 4.3.1. Cell Culture

Mycoplasma-free J744E cell lines were purchased from the European Collection of Authenticated Cell Cultures (ECACC, Salisbury, England) and maintained at the Institute of Immunology and Experimental Therapy (IIET, Wroclaw, Poland). Cells were thawed in a water bath at 37 °C with gentle stirring and placed in the culture of an Eagle medium (Life Technologies, Warszawa, Poland). Then, they were passaged twice a week in RPMI—1640 growth medium: 10% (*v*/*v*) FBS, 100 μg/mL streptomycin, 100 U/mL penicillin and 2 mM L-glutamine) in Petri dishes monitoring their growth under a microscope. The cells were separated from the bottom and collected from the culture vessel. Cell densities were counted in a Bürker chamber using a drop of cell suspension 1:1 in trypan blue. All cell lines were cultured during all experiments in a humid atmosphere at 37 °C and 5% CO_2_ and passaged twice a week using EDTA–trypsin solution (pH 8; IIET, Wroclaw, Poland) as a detachment agent.

#### 4.3.2. SRB Antiproliferative Assay

Twenty-four hours before addition of the tested compounds, the cells were seeded in 96-well plates (Sarstedt AG & Co. KG, Nümbrecht, Germany) in an appropriate culture medium. After 24 h, 100 µL of the tested compound in culture medium (10^−3^, 10^−4^, 10^−5^, 10^−6^ M) was added. The plates were incubated at 37 °C under a wet atmosphere saturated with 5% CO_2_. The in vitro cytotoxic effects of all bisphosphonates were examined using a colorimetric test (MTT assay) after 72 h exposure of the cultured cells to their varying concentrations [54]. Then, 20 µL aliquot of MTT solution (MTT: 3-(4,5-dimethylthiazol-2-yl)-2,5-diphenyl tetrazolium bromide) was added to each well and incubated for 4 h. After incubation, 80 µL of the lysing mixture was added to each well (lysing mixture: 225 mL of dimethylformamide, 67.5 g of sodium dodecyl sulfate and 275 mL of distilled water). The optical densities of the samples were determined after 24 h using a Multiskan RC photometer (Labsystems, Helsinki, Finland) at 570 nm.

Cells were treated with each compound using at least four concentrations in the range of 1000 µM–1 µM for 72 h. Then, 0.2 M NaOH, used as a stock solution solvent, was tested for antiproliferative activity, and it did not affect the cell proliferation at 1 mM, which was the highest concentration used in the compound solutions.

Each concentration of compounds was tested in triplicate in a single experiment, and each experiment was repeated at least three times independently. The results are presented as the mean IC_50_ ± standard deviation (SD) calculated using the Prolab-3 system based on Cheburator 0.4 software [55].

### 4.4. Studies on Healing Effect on Sheep

Each bisphosphonate was tested on a group of seven sheep, aged 5–6 years, weighing 45–60 kg. To monitor the changes that occur in the animals participating in the experiment, a positive control group (PP) and a negative control group (PN) were created. The negative control group consisted of one sheep that did not undergo ovariectomy and was not subjected to steroid therapy. This was a healthy sheep, in which it was possible to observe the correct histological image of the bone tissue. The positive control was one sheep that underwent ovariectomy and steroid therapy and was not treated with new bisphosphonic acid. This group aimed to enable the observation of the development process of osteoporosis similar to postmenopausal osteoporosis. The research group consisted of five bisphosphonate-treated sheep after the induction of osteoporosis and steroid therapy. The animals were kept in groups of two in small boxes (10 m^2^). Animals from both control groups were kept under the same conditions as sheep from the test groups and were given the same food. This housing restricted their physical activity and access to sunlight. The sheep were fed twice a day with hay and had unrestricted access to water. Two weeks after the ovariectomy (surgical removal of both ovaries), a low-calcium diet and the methylprednisolone administration were started at a dose of 250 mg/sheep. Before administering the drug, radiological examinations of the tibia and humerus of each sheep were performed in two projections, lateral and sagittal, in order to visualize the structure of the bone. Subsequent doses of methylprednisolone were administered every 20 days at a reduced dose of 150 mg/sheep. In total, the sheep received the steroid drug four times. All animals were weighed every 10 days, and blood samples were collected for biochemical, morphological, hormonal, and bone turnover markers every 10 days. In addition, X-ray examination were performed in a standing position to monitor the changes in bone microstructure. Three months after induction of the osteoporosis, the administration of the tested bisphosphonate began. The aqueous solution of bisphosphonate (35 mg/sheep) was administered to the animals weekly using a probe directly into the rumen for a period of 9 weeks. The first three doses were 150 mg per sheep, while the next six were 300 mg per sheep. Each sheep received nine doses of bisphosphonate. During bisphosphonate therapy, radiological and hematological tests (biochemistry and biochemical profiles) were performed every 10 days. Euthanasia was performed on the 30th day after the end of bisphosphonate therapy with an intravenous high dose (60 mg/kg body weight) of pentobarbital (Morbital^®^, BioWet, Puławy, Poland).

The animals were sacrificed, and the bone samples from the mandible, humerus, radius, ulna, femur, tibia, and pelvic bone were collected for histology.

### 4.5. Histological Studies

For histological examination, fragments of the femur, humerus, lumbar vertebra, and mandible were collected from each sheep. Bone samples were fixed in 4% formalin solution and decalcified by successive bathing in 10% sodium versenate, distilled water, a mixture of formic and citric acids, and distilled water. Then, they were dehydrated and stored in paraffin. The bone segments were sectioned with a microtone to 6–7 μm slides for histomorphometry analysis. The samples were fixed for 3 h in a 3.5% solution of glutaralaldehyde in phosphate buffer (0.1 M, pH between 7.2 and 7.4), washed thoroughly with buffer and then fixed with a solution containing 1.0% osmium tetroxide in buffer. After drying (ethanol–acetone), the samples were submerged in Epon 812.

Bone slides were stained using four methods (Delafield method with hematoxylin and eosin (HiE), Van Gieson procedure, Goldner–Masson procedure and von Koss procedure and analyzed using transmission microscopy (Tesla BS-500, Brno, Czech Republic). HiE staining is a survey method that allows observation of the overall morphology of bone tissue. This method allowed for the observation of differences between healthy and osteoporotic bone tissue as well as between the two study groups. The use of van Gieson staining in morphometry enabled the counting of primary and secondary osteons as well as the measurement of the thickness of the systemic and intersystemic lamellae of bone tissue. The purpose of this method was to perform measurements that allowed for a statistical assessment of changes that occurred in all study groups. Goldner–Masson and von Koss staining was used to assess the degree of bone mineralization. Due to the decalcification process that precedes staining, the Goldner–Masson and von Koss methods should be considered qualitative, not quantitative. Since all sections were treated identically, it was assumed that the amount of calcium released during the decalcification baths was the same.

After preliminary histological analysis, morphometry of the structures that form bone tissue was performed. The parameters for primary and secondary osteons considered included the number of primary and secondary osteons in the field of view at 200x magnification and the thickness of the systemic and intersystemic laminae. Statistically significant differences were observed at the 5% level between the treated animals and each control group indicating progress in curation. The data obtained were statistically analyzed using the Statistica 9.1 package from StatSoft Inc (Tulsa, OK, USA). They were subjected to an analysis of variance (ANOVA). If the analysis of variance allowed the rejection of the hypothesis of equal means in all groups, further analysis (post hoc) was performed using the Tukey test for groups of different sizes to determine which groups had statistically significant differences at the 5% level.

## 5. Patents

International patents application: WO/2015/159153 A1; 22 October 2015, Novel bisphosphonates and their use. Polish patent application: PL407922 (A1); 16 April 2014, Nowe bisfosfoniany i ich zastosowanie.

## Figures and Tables

**Table 1:** Structures of the obtained bisphosphonates and their activity against mouse macrophages J774E (concentration resulting in a 50% inhibition of proliferation).

Structure			
Compound	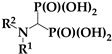		IC_50_ * ± SD [µM]
	R^1^	R^2^	
**1**	H	H	318 ± 26
**2**	*n*-propyl	H	750 ± 13
**3**	*n*-pentyl	H	400 ± 27
**4**	*n*-heptyl	H	20 ± 42
**5**	*n*-octyl	H	13 ± 41
**6**	*n*-nonyl	H	11 ± 84
**7**	*n*-decyl	H	30 ± 32
**8**	*n*-undecyl	H	32 ± 40
**9**	*n*-dodecyl	H	32 ± 24
**10**	*n*-tridecyl	H	32 ± 19
**11**	*n*-tetradecyl	H	18 ± 20
**12**	*i*-propyl	H	310 ± 42
**13**	*i*-butyl	H	312 ± 38
**14**	*i*-pentyl	H	308 ± 44
**15**	*i*-hexyl	H	391 ± 49
**16**	*i*-oct-1-yl	H	360 ± 43
**17**	2,5-dimethylhexyl	H	IN **
**18**	cyclopropyl	H	280 ± 7
**19**	cyclopentyl	H	331 ± 12
**20**	cyclohexyl	H	420 ± 40
**21** (Incadronate)	cycloheptyl	H	254 ± 93
**22**	cyclooktyl	H	240 ± 20
**23**	*n*-propyl	*n*-propyl	IN **
**24**	*n*-cyclohexyl	*n*-cyclohexyl	IN **
**25**	cyclohexyl	ethyl	IN **
**26**	(CH_2_)_4_		300 ± 75
**27**	(CH_2_)_5_		284 ± 67
**28**	(CH_2_)_6_		201 ± 15
**29**	(CH_2_)_7_		345 ± 28
**30**	(CH_2_)_2_CH(CH_3)_(CH_2_)_2_		201 ± 15
**31**	CH_3_CH(CH_2_)_3_CHCH_3_		IN **
**32**	CH_2_CH(CH)_3_CH_2_CH(CH)_3_CH_2_		IN **
**33**	CH_2_(CH_2_)_3_CHCH_2_CH_3_		IN **
**34**	(CH_2_)_2_O(CH_2_)_2_		275 ± 35
**35**	CH_2_CH(CH_3_)OCH(CH_3_)(CH_2_)_2_		3195 ± 28

* IC_50_ values were determined at concentrations in the range of 1–1000 μg/mL. Values are mean ± standard deviation (SD) from at least three experiments performed in triplicates. ** IN—inactive.

## Data Availability

The original contributions presented in this study are included in the article/Supplementary Materials. Further inquiries can be directed to the corresponding author.

## Supplementary Materials

The following supporting information can be downloaded at https://www.mdpi.com/article/10.3390/ph19020256/s1, containing copies of ^1^H-NMR, ^31^P-NMR, ^13^C-NMR, HRMS spectra of all synthesized compounds.
