# Supplementary material for: Probing the Hydrophobic Part of Analogues of the Incadronate-Evidence of Their Interaction with Immunological System of Sheep
Source: Pharmaceuticals (Basel). 2026 Feb 1;19(2):256. doi: 10.3390/ph19020256 (PMC12943640; doi:10.3390/ph19020256)
Supplement: Supplementary file 1 [file pharmaceuticals-19-00256-s001.zip › pharmaceuticals-4115204-supplementary.pdf]

Supporting Information for

Probing the Hydrophobic Part of Analogues of the Incadronate. Evidence of Their Interaction with Immunological System of Sheep

Ewa Chmielewska[1]*, Joanna Wietrzyk[2], Jan Kuryszko[3†], Zdzisław Kiełbowicz[3†] and Paweł Kafarski[4]

[1] Department of Bioorganic Chemistry, Faculty of Chemistry, Wrocław University of Science and Technology, Wybrzeże Wyspiańskiego 27, 50-370 Wrocław, Poland; ewa.chmielewska@pwr.edu.pl (E.C.)
[2] Laboratory of Experimental Anticancer Therapy, Department Of Experimental Oncology, Ludwik Hirszfeld Institute of Immunology and Experimental Therapy Polish Academy of Sciences, Rudolfa Weigla 12, 53-114 Wrocław, Poland; joanna.wietrzyk@hirszfeld.pl (J.W.)
[3] The Faculty of Veterinary Medicine, Wrocław University of Environmental and Life Sciences, Norwida 31; 50-375 Wrocław, Poland; jan.kuryszko@upwr.edu.pl (J.K.); zdzislaw.kielbowicz@upwr.edu.pl (Z.K.)
[4] Department of Chemistry, Faculty of Agriculture and Forestry, University of Warmia and Mazury, Plac Łódzki 4, 10-721 Olsztyn, Poland; pawel.kafarski@uwm.edu.pl (P.K.)
* Correspondence: ewa.chmielewska@pwr.edu.pl ; Tel.: +48 71 320 2977

Copies of ^{31}P NMR, ^{1}H NMR, ^{13}C NMR, MS spectra for representative compounds

Table of contents — Page

Compound **1** – ^{31}P NMR, ^{1}H NMR, ^{13}C NMR, MS, ……………………………….……………….…...**2**
Compound **2** - ^{31}P NMR, ^{1}H NMR, ^{13}C NMR, MS…………………………………………………..….**4**
Compound **3** - ^{31}P NMR, ^{1}H NMR, ^{13}C NMR, MS………………………………………………….....**6**
Compound **4** - ^{31}P NMR, ^{1}H NMR, ^{13}C NMR, MS……………………………………………………….**8**
Compound **5** - ^{31}P NMR, ^{1}H NMR, ^{13}C NMR, MS……………………………………………………..**10**
Compound **6** - ^{31}P NMR, ^{13}C NMR…………………………………………………………………..… ..**12**
Compound **7** - ^{31}P NMR, ^{13}C NMR……………………………………………………………………...**14**
Compound **8** - ^{31}P NMR, ^{13}C NMR……………………………………………………………………..**16**
Compound **9** - ^{31}P NMR, ^{13}C NMR, MS……………………………………………………………….. **17**
Compound **10** - ^{31}P NMR, ^{13}C NMR…………………………………………………………………….**19**
Compound **11** - ^{31}P NMR, ^{13}C NMR…………………………………………………………………… .**21**
Compound **12** - ^{31}P NMR, ^{1}H NMR, ^{13}C NMR, MS…………………………………………………… **22**
Compound **13** - ^{31}P NMR, ^{1}H NMR, ^{13}C NMR, MS…………………………………………………… **24**
Compound **14** - ^{31}P NMR, ^{1}H NMR, ^{13}C NMR, MS…………………………………………………… **26**
Compound **15** - ^{31}P NMR, ^{1}H NMR, MS…..…………………..……………………………………… **28**
Compound **16** - ^{31}P NMR, ^{1}H NMR, ^{13}C NMR, MS…………………………………………………… **30**
Compound **17** - ^{31}P NMR, ^{1}H NMR, ^{13}C NMR, MS…………………………………………………..**32**
Compound **18** - ^{31}P NMR, ^{1}H NMR, ^{13}C NMR…………………………………………………………… **34**
Compound **19** - ^{31}P NMR, ^{1}H NMR, ^{13}C NMR………………………………………………………….....**36**
Compound **20** - ^{31}P NMR, ^{1}H NMR, ^{13}C NMR, MS…………………………………………………… **37**
Compound **21** - ^{31}P NMR, ^{1}H NMR, ^{13}C NMR, MS…………………………………………………… **39**
Compound **22** - ^{31}P NMR, ^{1}H NMR, ^{13}C NMR, MS…………………………………………………… **41**

Compound **23** - ^{31}P NMR, ^{1}H NMR, ^{13}C NMR……………………………………………………………………......**43**
Compound **24** - ^{31}P NMR, ^{1}H NMR, ^{13}C NMR….. ……………………………………………………….……**45**
Compound **25** - ^{31}P NMR, ^{1}H NMR, ^{13}C NMR, MS……………………………………………………………..**46**
Compound **26** - MS………………………………………………………………………………………….. **48**
Compound **27** - ^{31}P NMR, ^{1}H NMR, ^{13}C NMR, MS…………………………………………………………….… **49**
Compound **28** - ^{31}P NMR, ^{1}H NMR……………………………………………………………………………… **51**
Compound **29** - ^{31}P NMR, ^{1}H NMR……………………………………………………………………………… **52**
Compound **30** - ^{31}P NMR, ^{1}H NMR, ^{13}C NMR, MS……………………………………………………………....**53**
Compound **31** - ^{31}P NMR, ^{1}H NMR, ^{13}C NMR, MS…………………………………………………………….**55**
Compound **32** - ^{31}P NMR, ^{1}H NMR, ^{13}C NMR, MS…………………………………………………………… **57**
Compound **33** - ^{31}P NMR, ^{1}H NMR, ^{13}C NMR, MS……………………………………………………………....**60**
Compound **34** - ^{31}P NMR, ^{1}H NMR, ^{13}C NMR, MS…………………………………………………………….....**62**
Compound **35** - ^{31}P NMR, ^{1}H NMR, ^{13}C NMR, MS……………………………………………………………....**64**

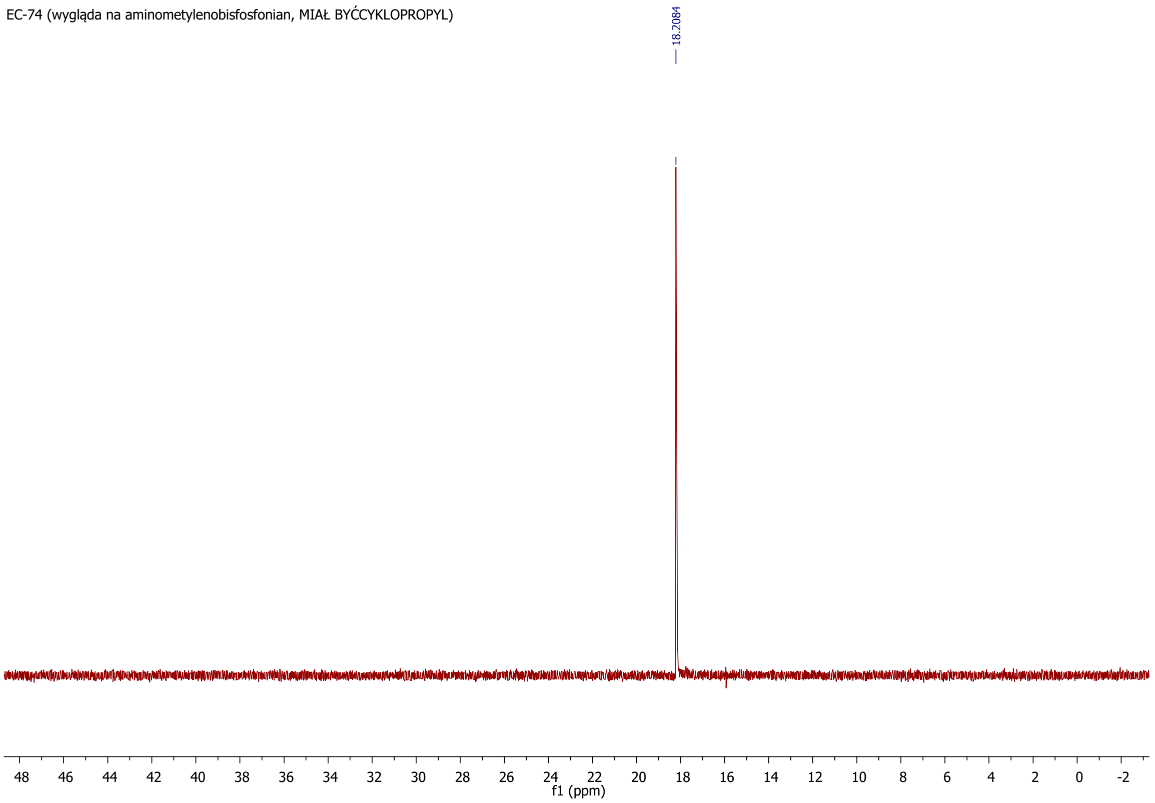

^{31}P NMR spectrum of compound **1**

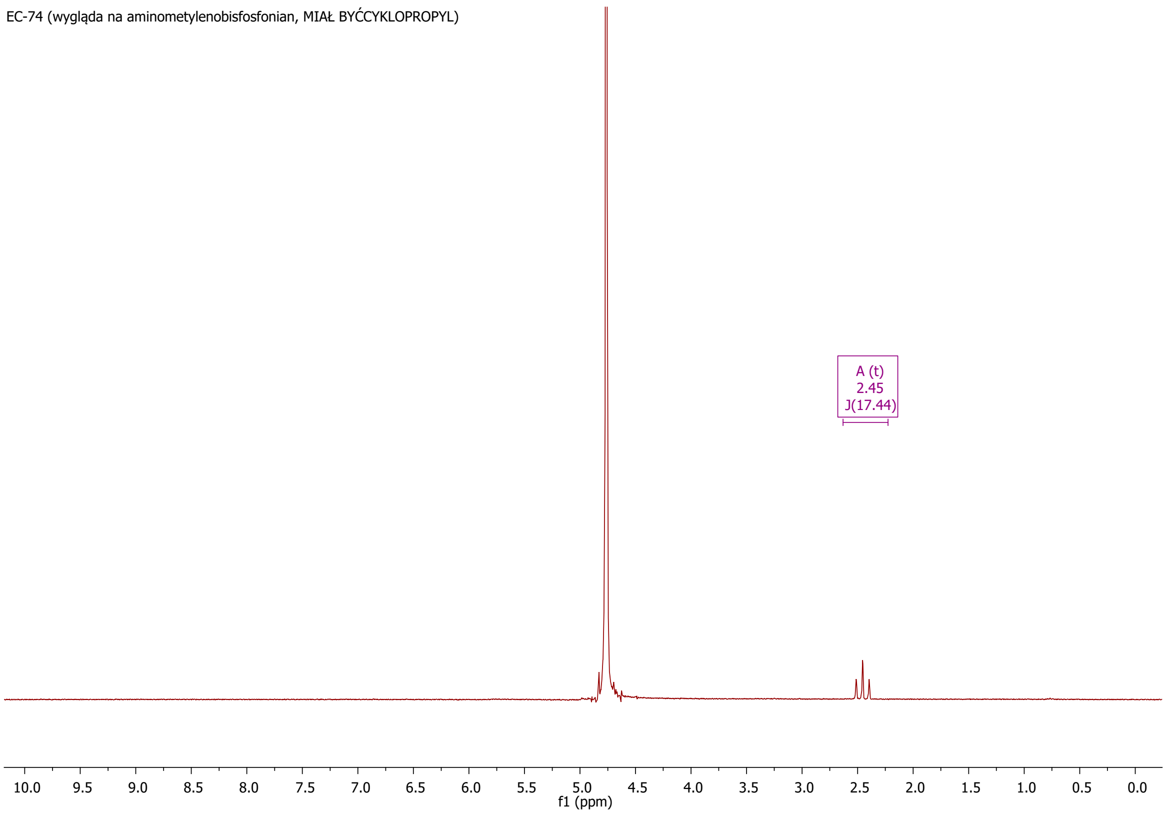

^{1}H NMR spectrum of compound **1**

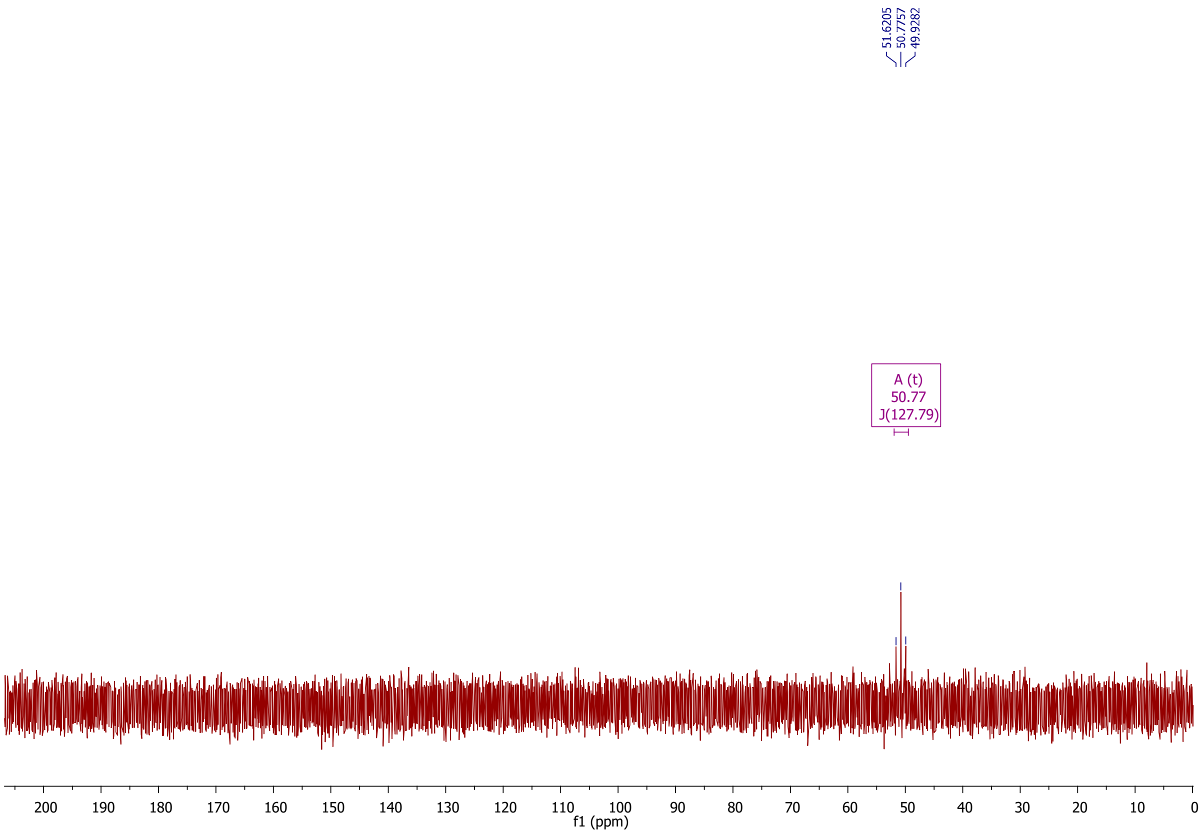

^{13}C NMR spectrum of compound **1**

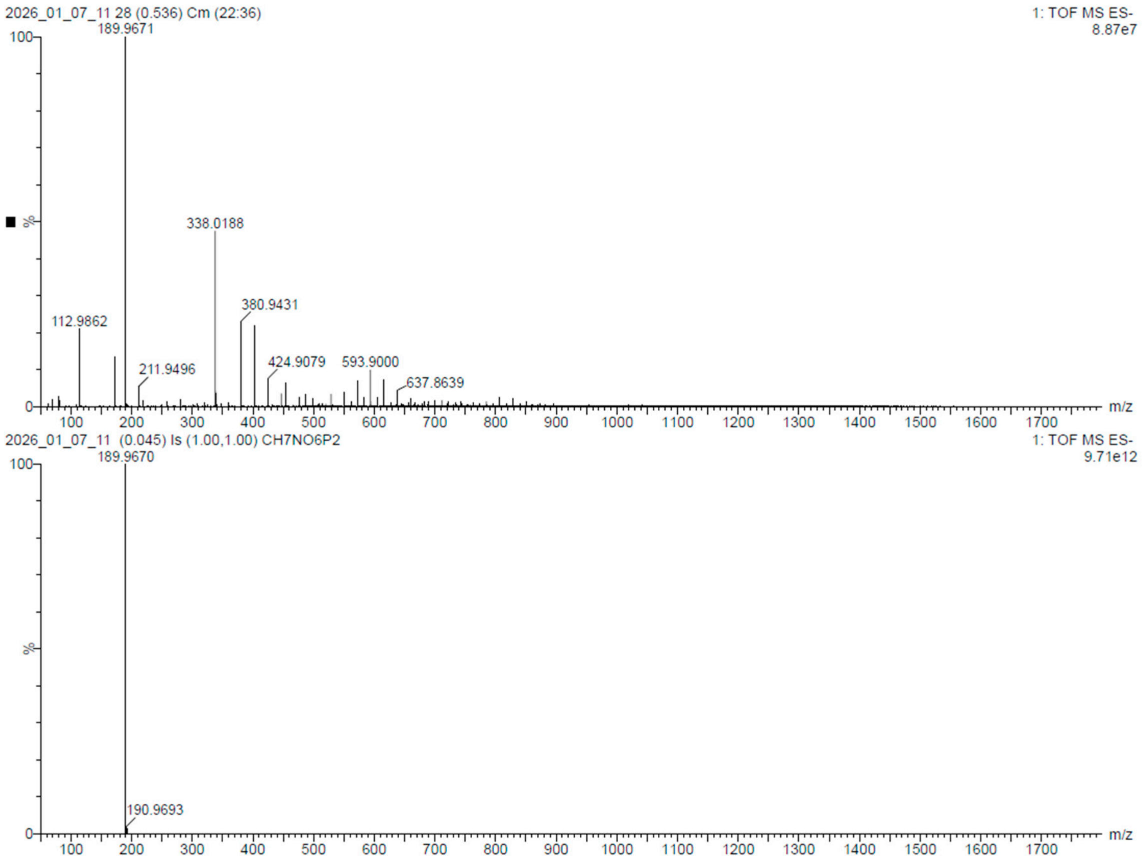

MS spectrum of compound **1**

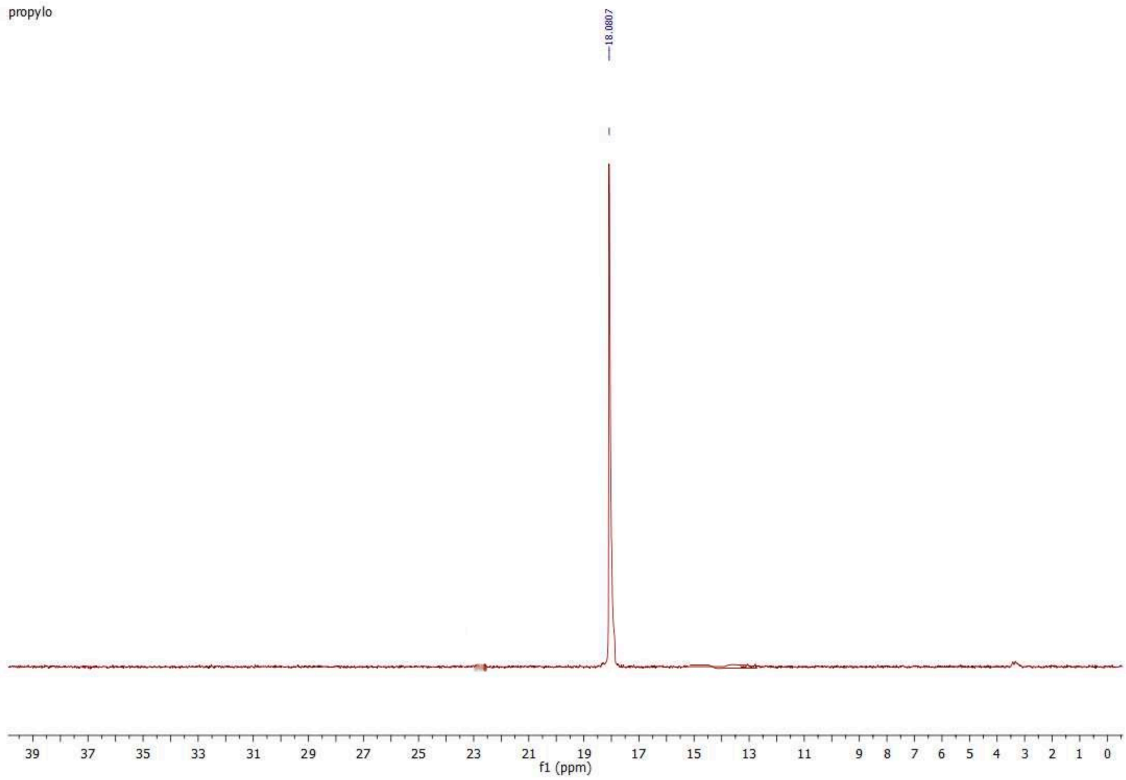

^{31}P NMR spectrum of compound **2**

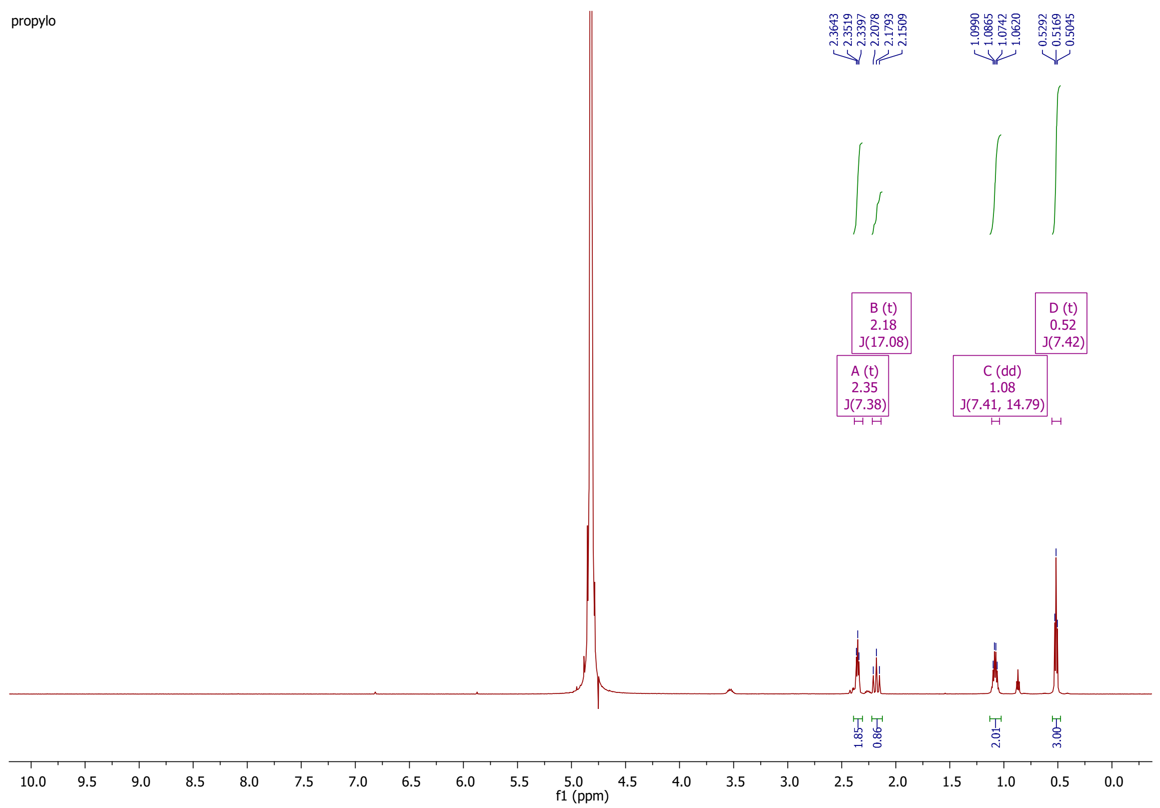

^{1}H NMR spectrum of compound **2**

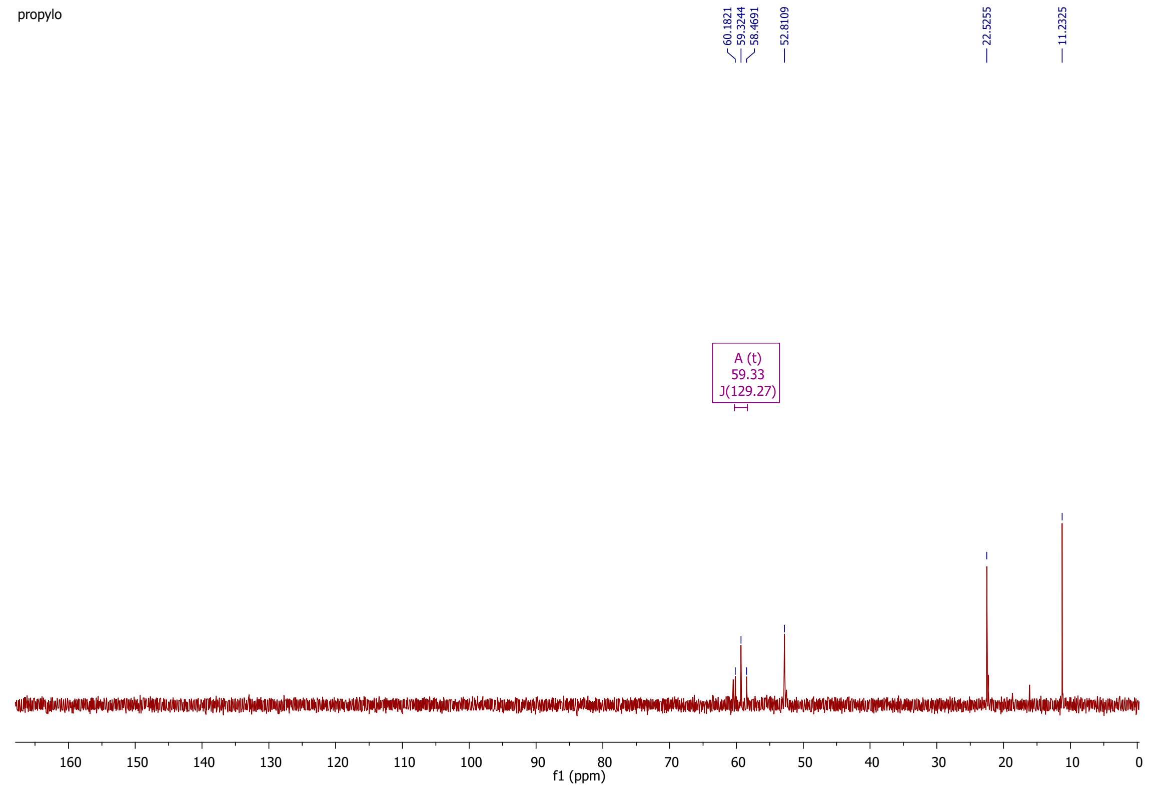

^{13}C NMR spectrum of compound **2**

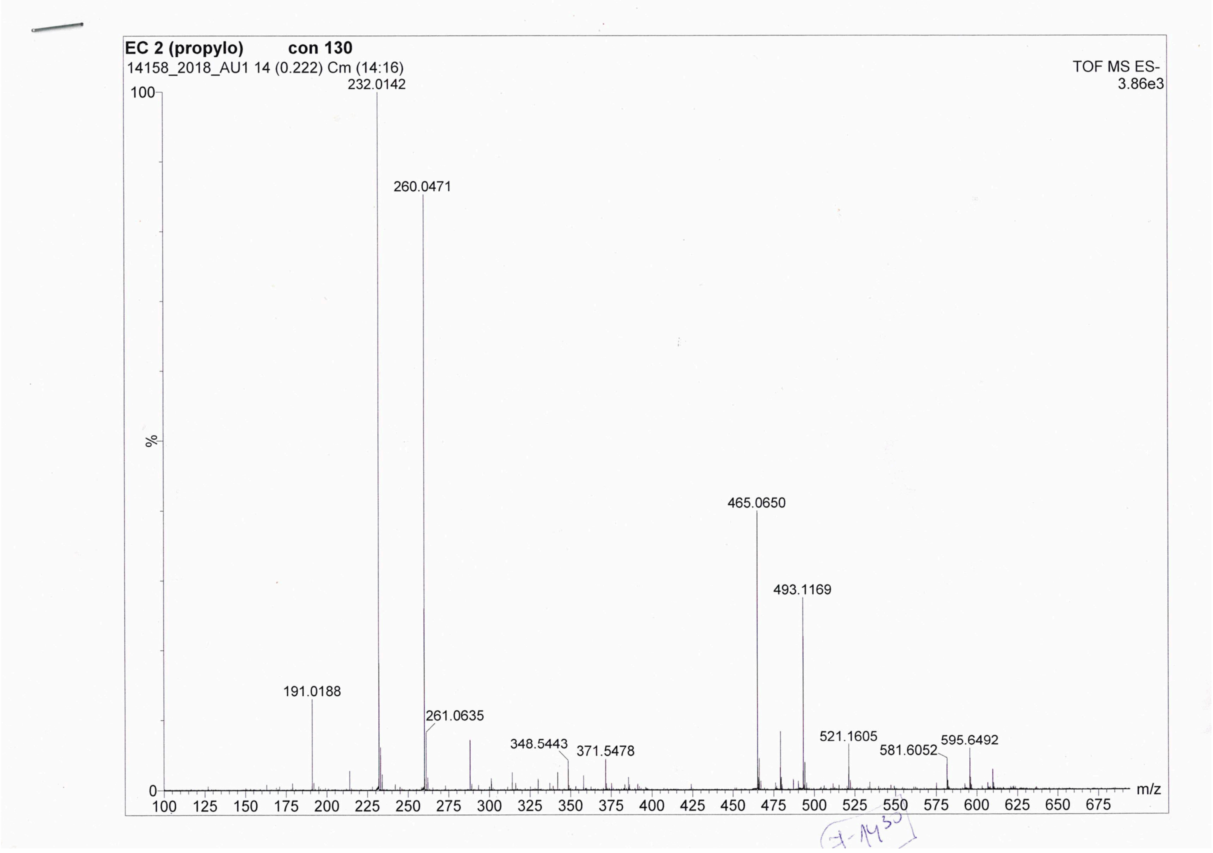

MS spectrum of compound **2**

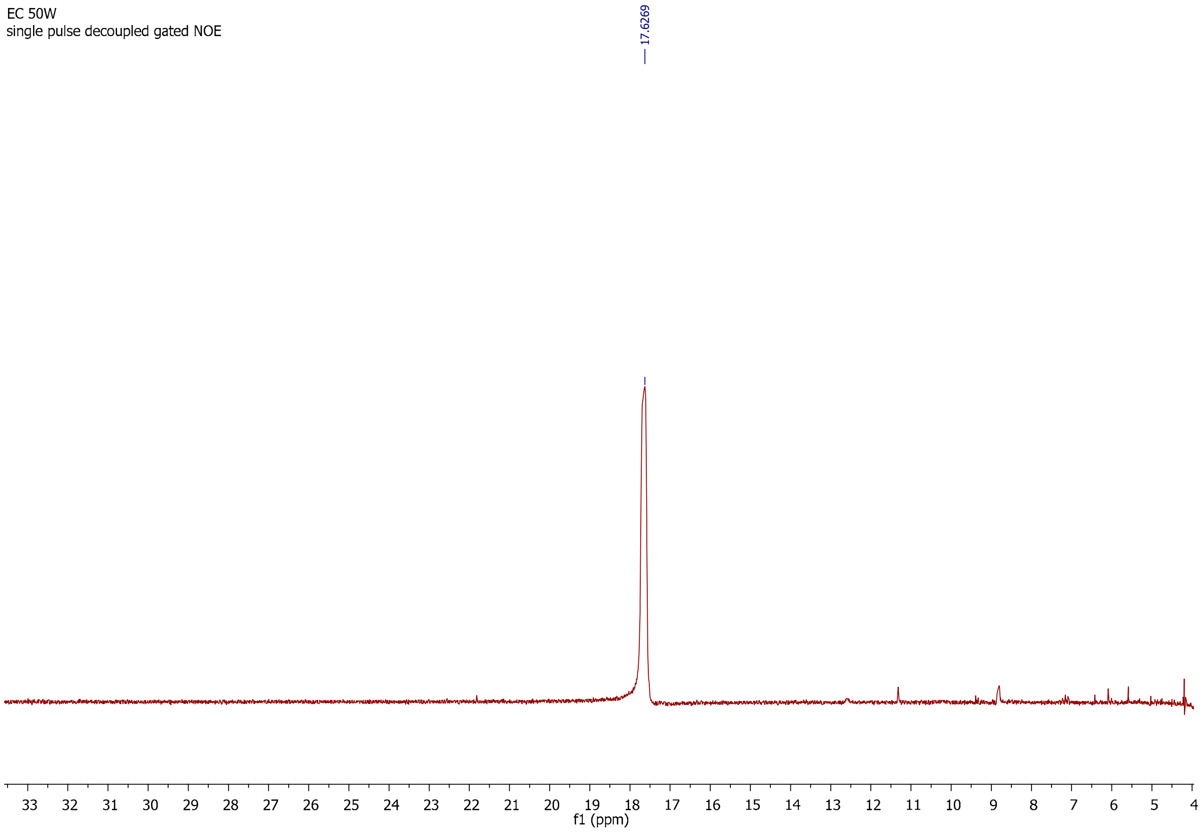

^{31}P NMR spectrum of compound **3**

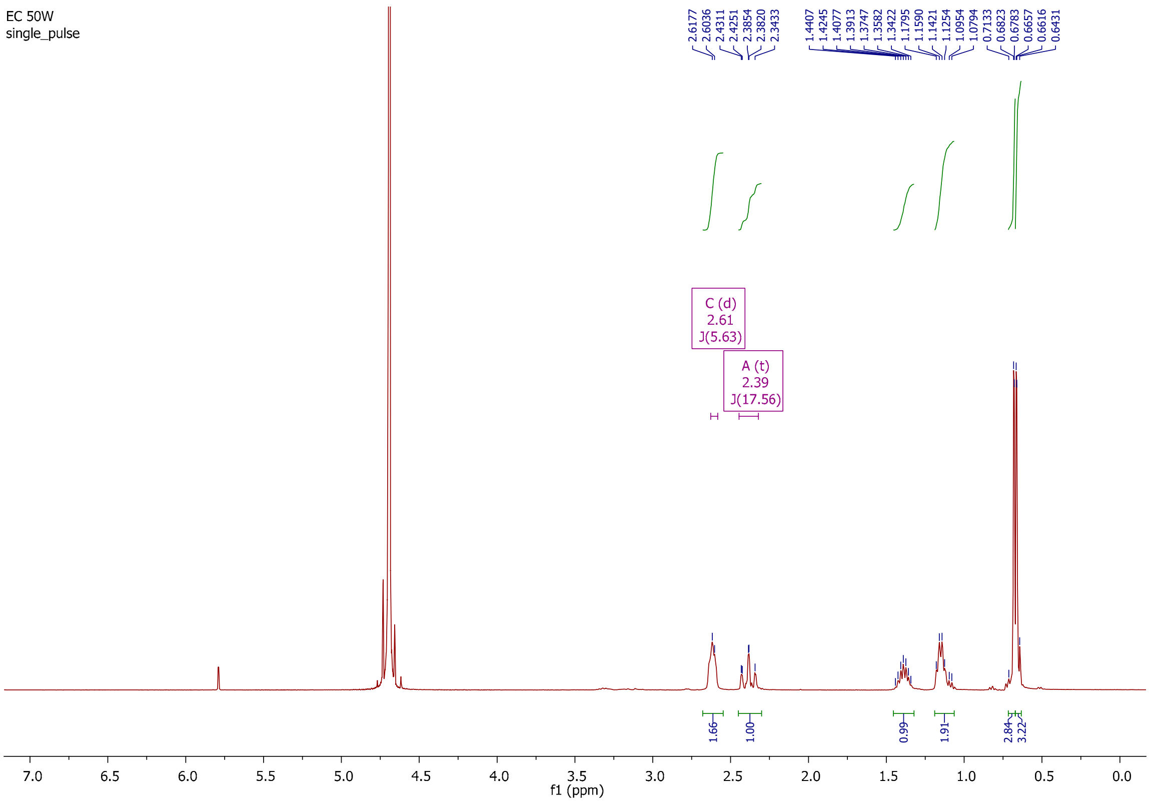

^{1}H NMR spectrum of compound **3**

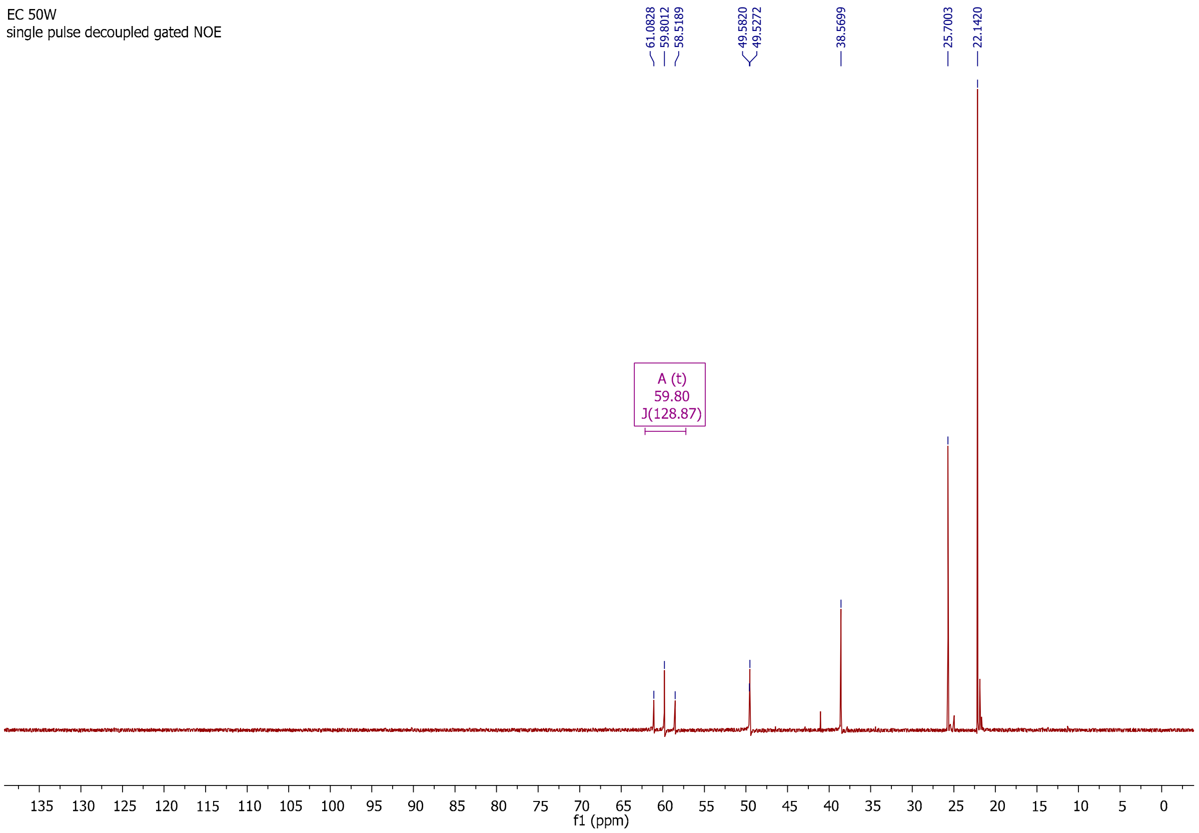

[31]C NMR spectrum of compound **3**

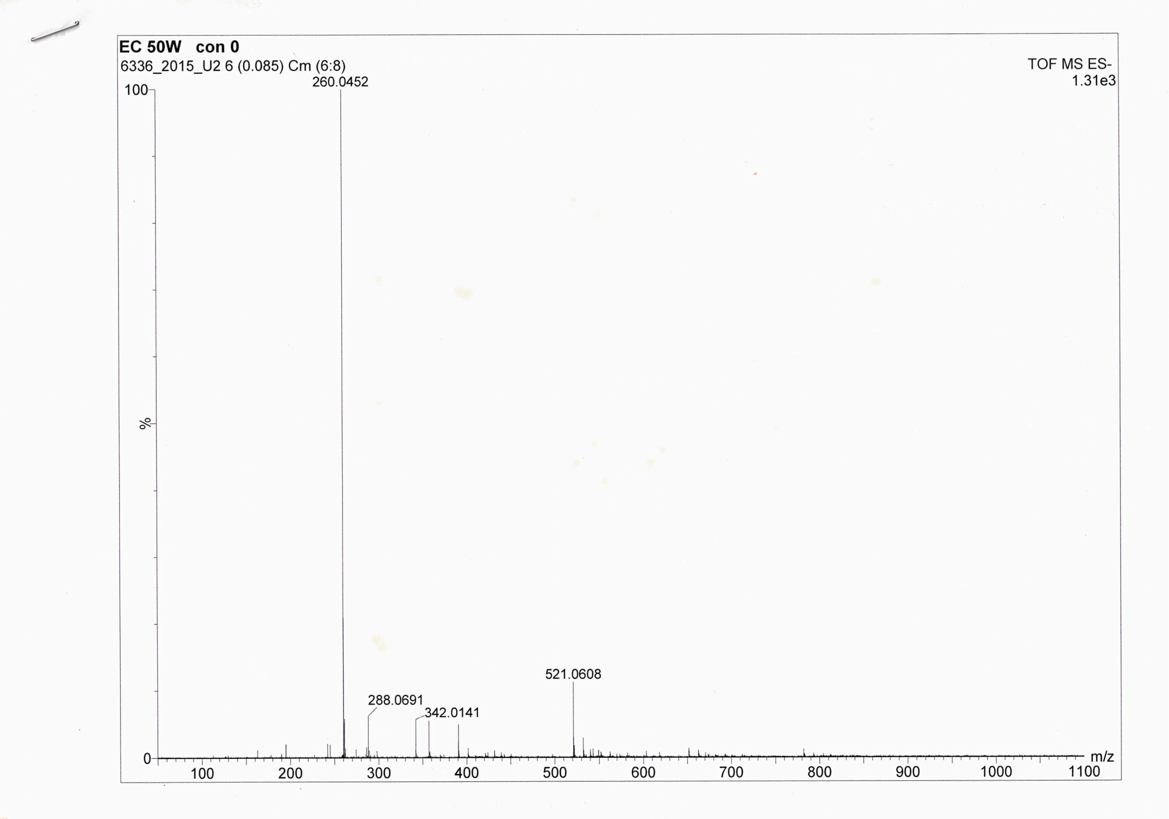

MS spectrum of compound **3**

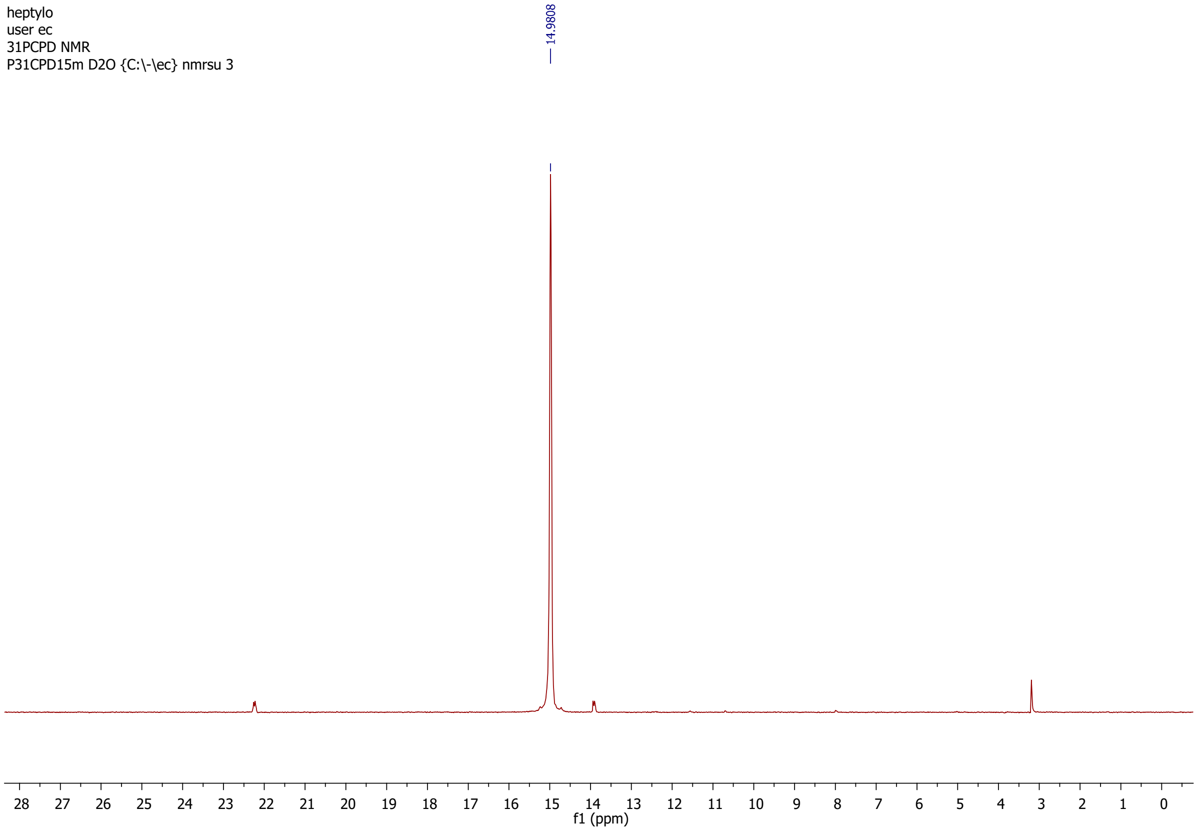

^{31}P NMR spectrum of compound **4**

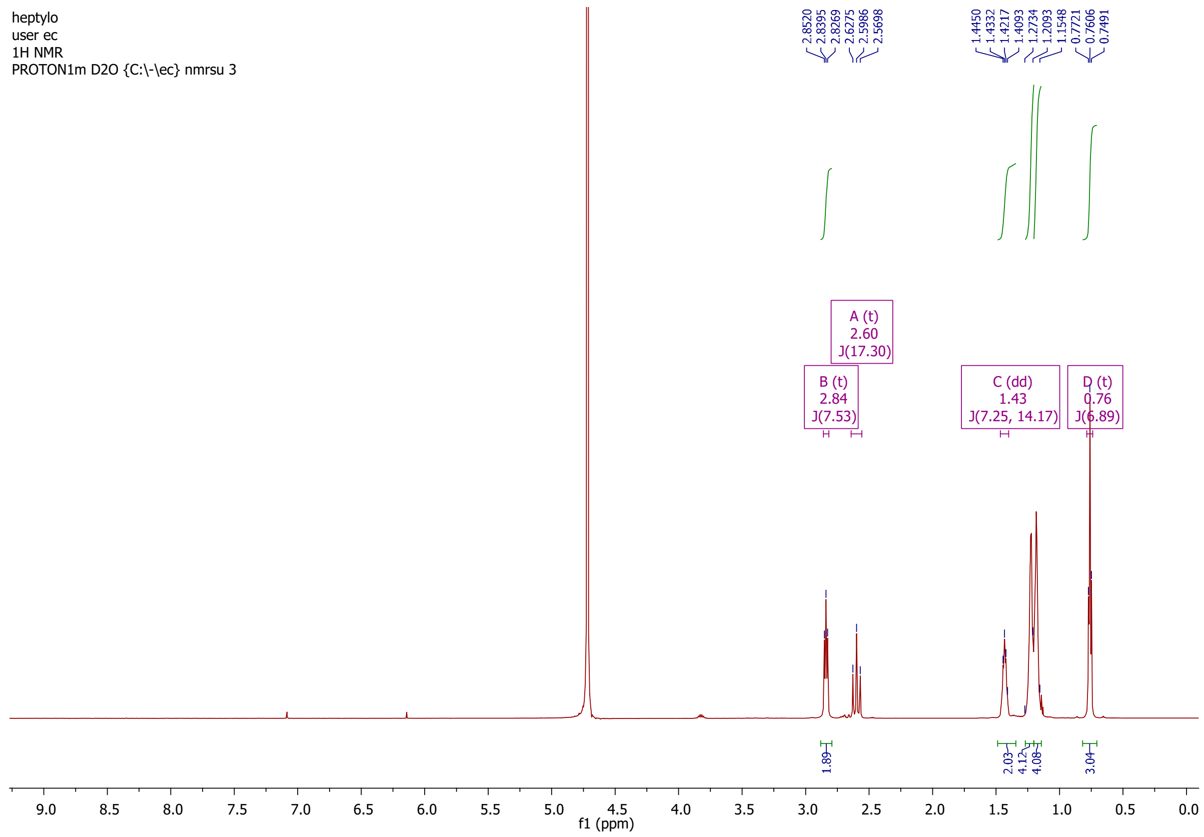

^{1}H NMR spectrum of compound **4**

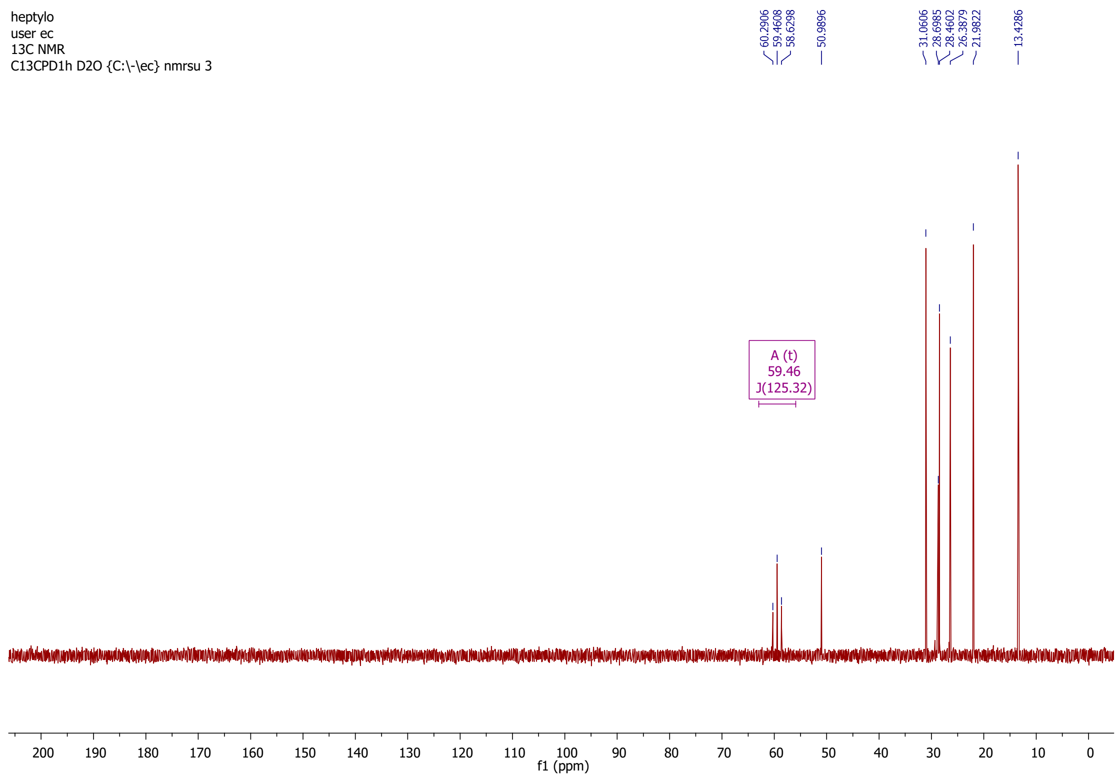

^{31}P NMR spectrum of compound **4**

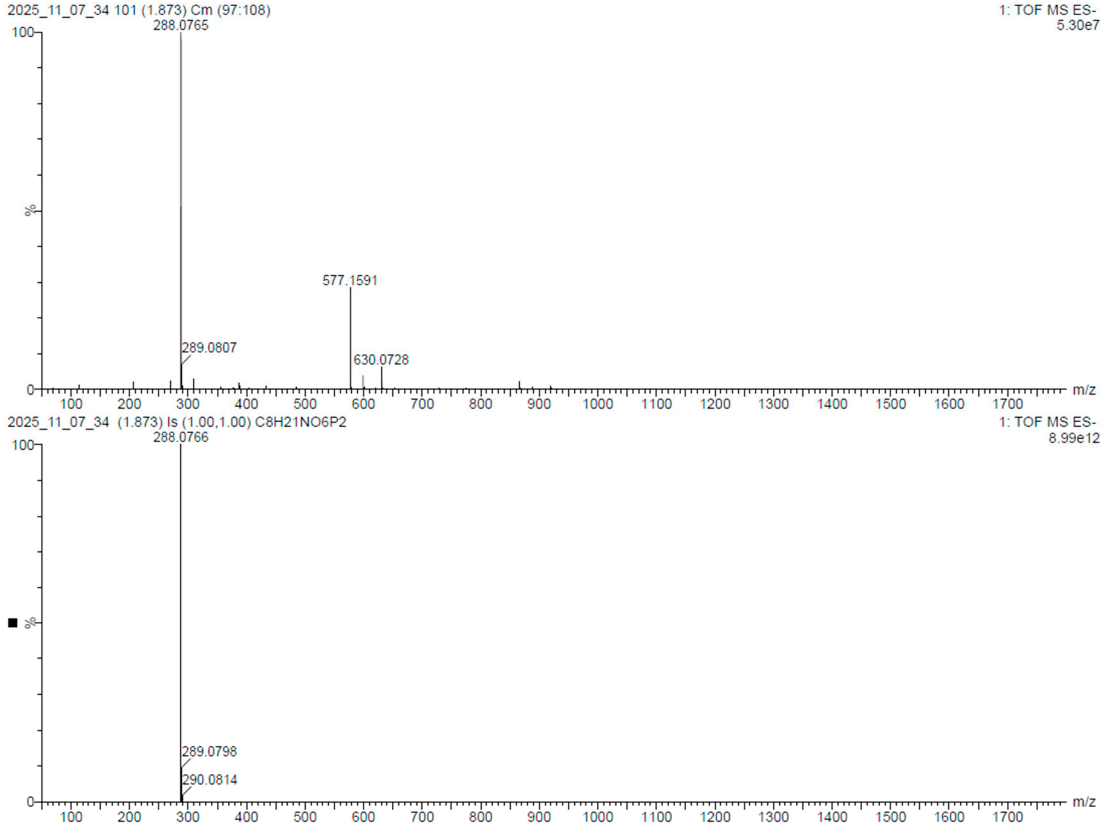

MS spectrum of compound **4**

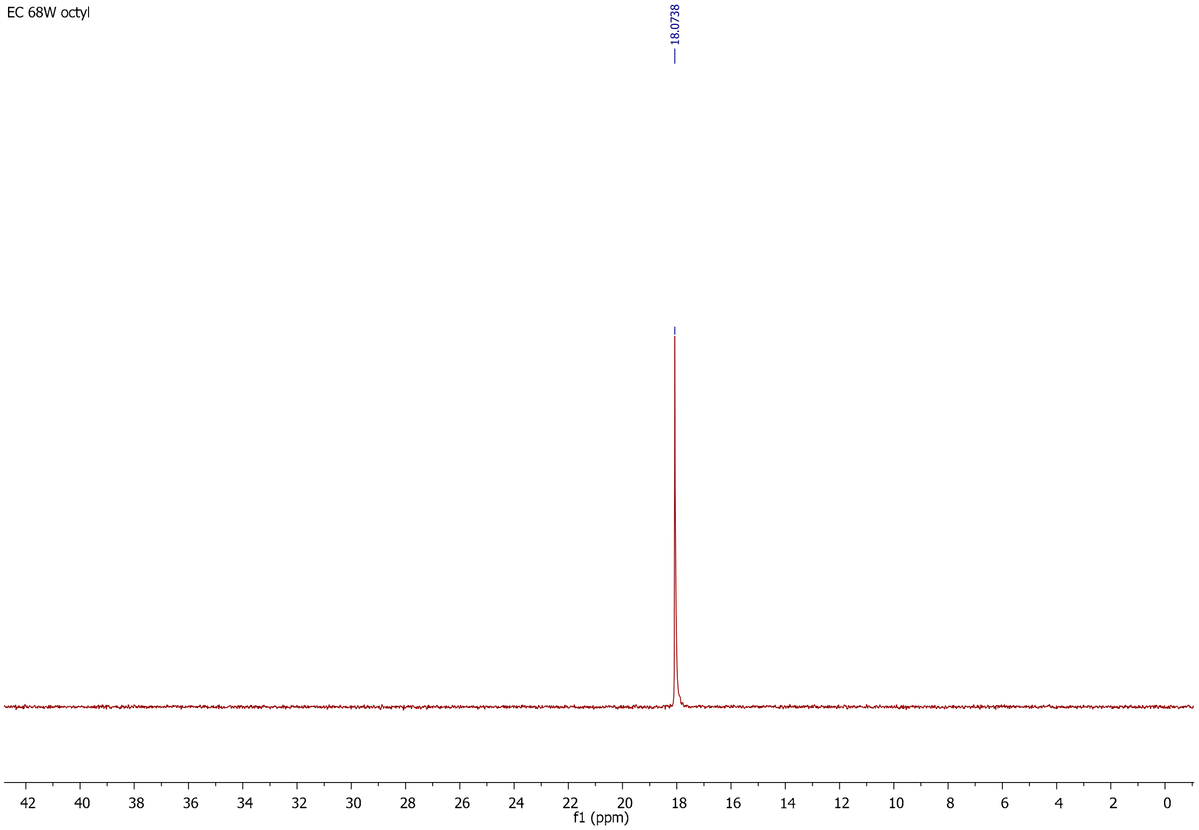

^{31}P NMR spectrum of compound **5**

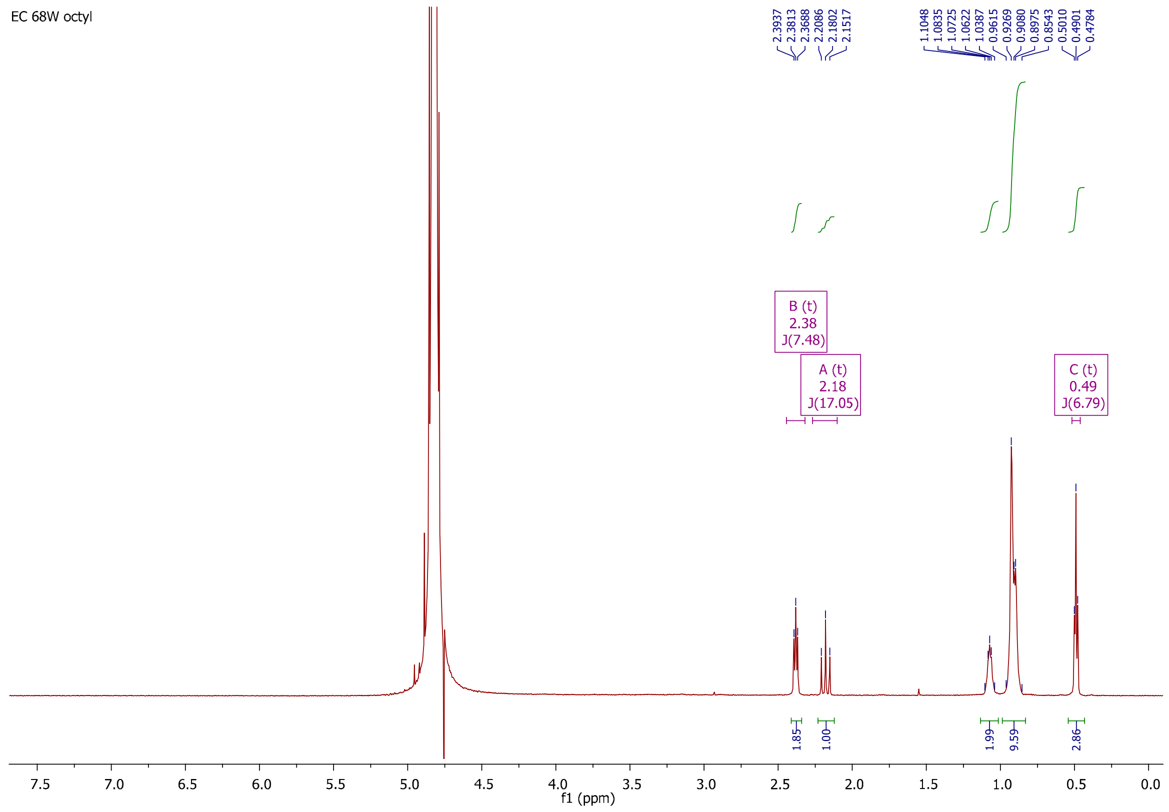

^{1}H NMR spectrum of compound **5**

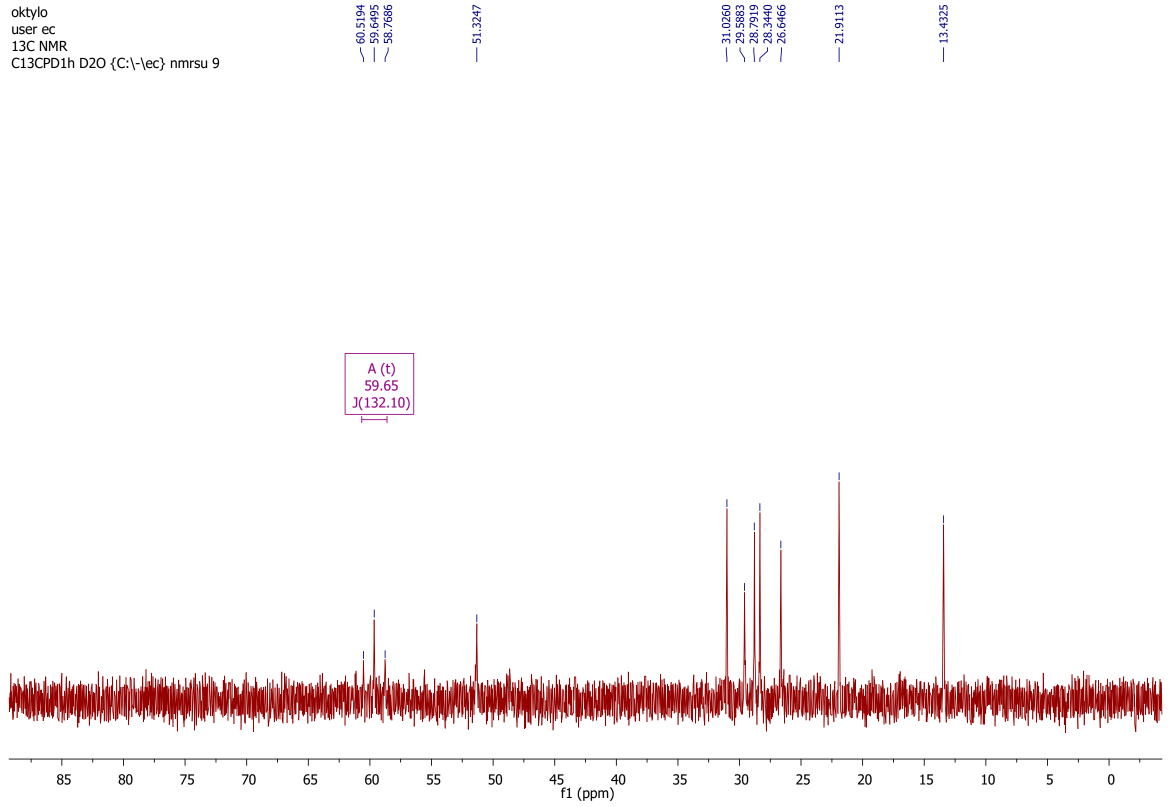

^{13}C NMR spectrum of compound **5**

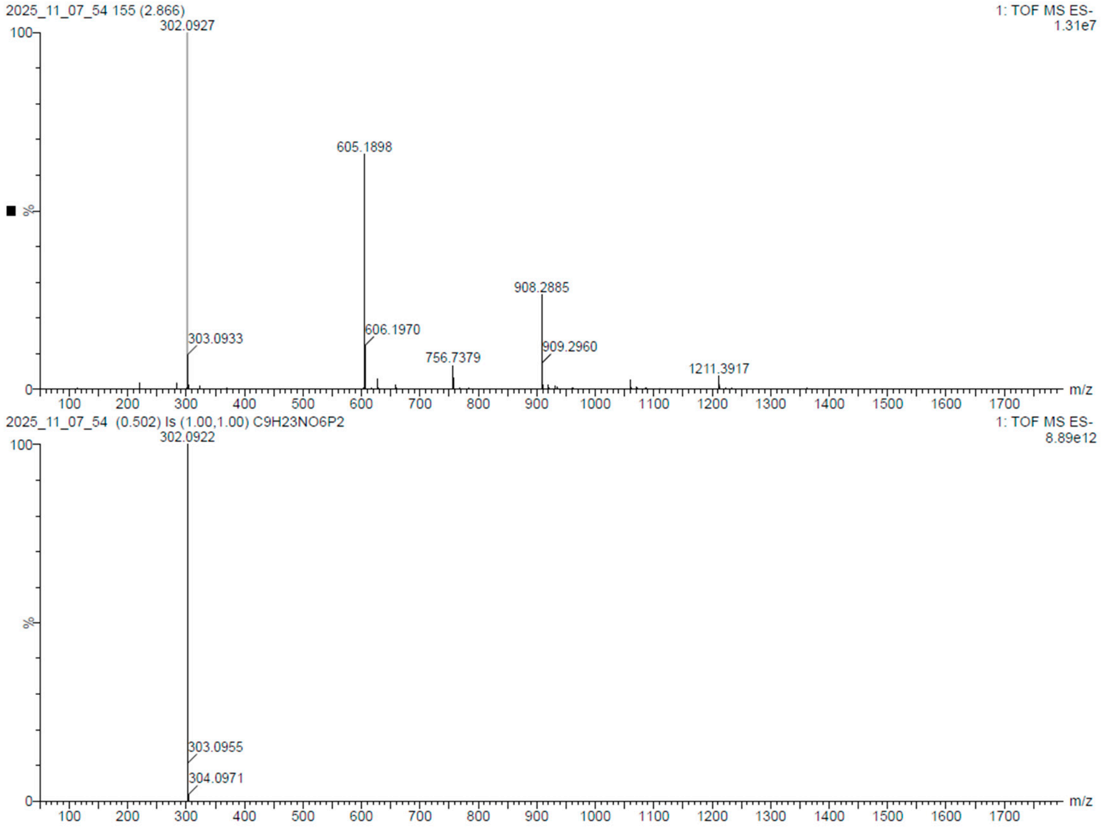

MS spectrum of compound **5**

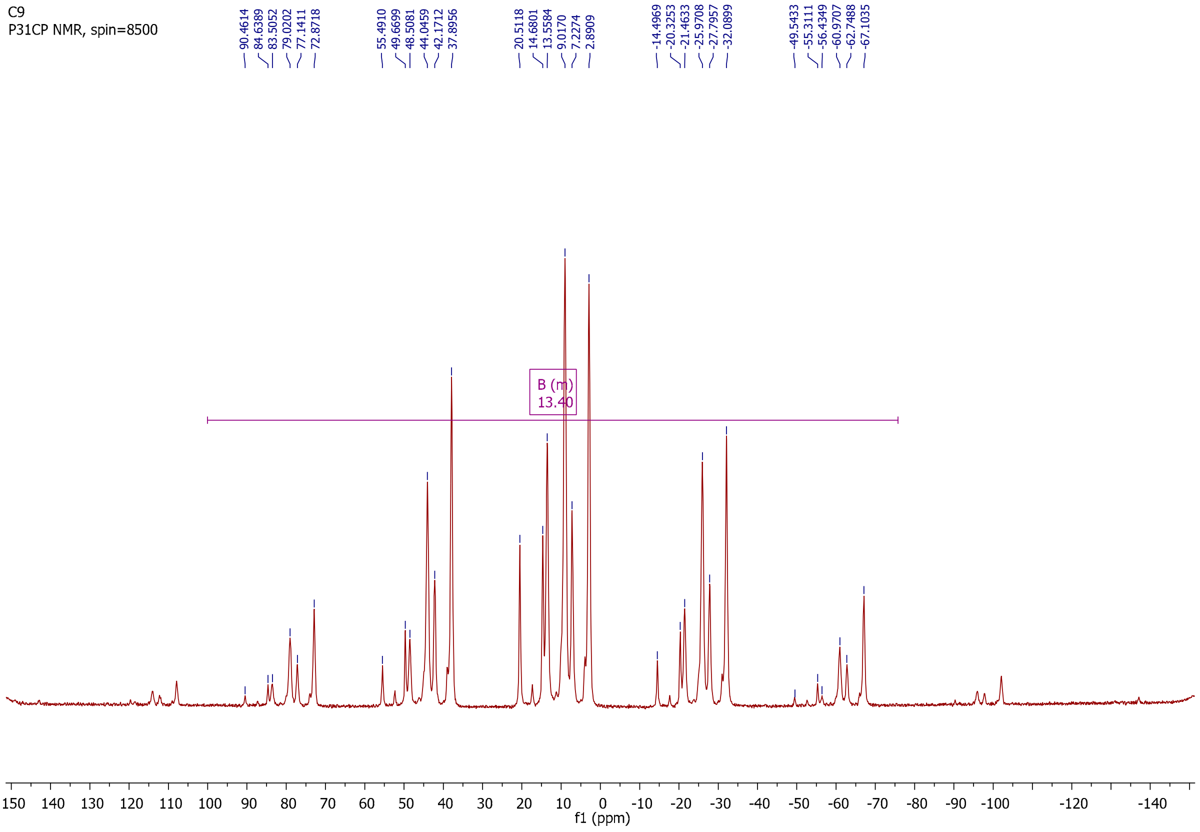

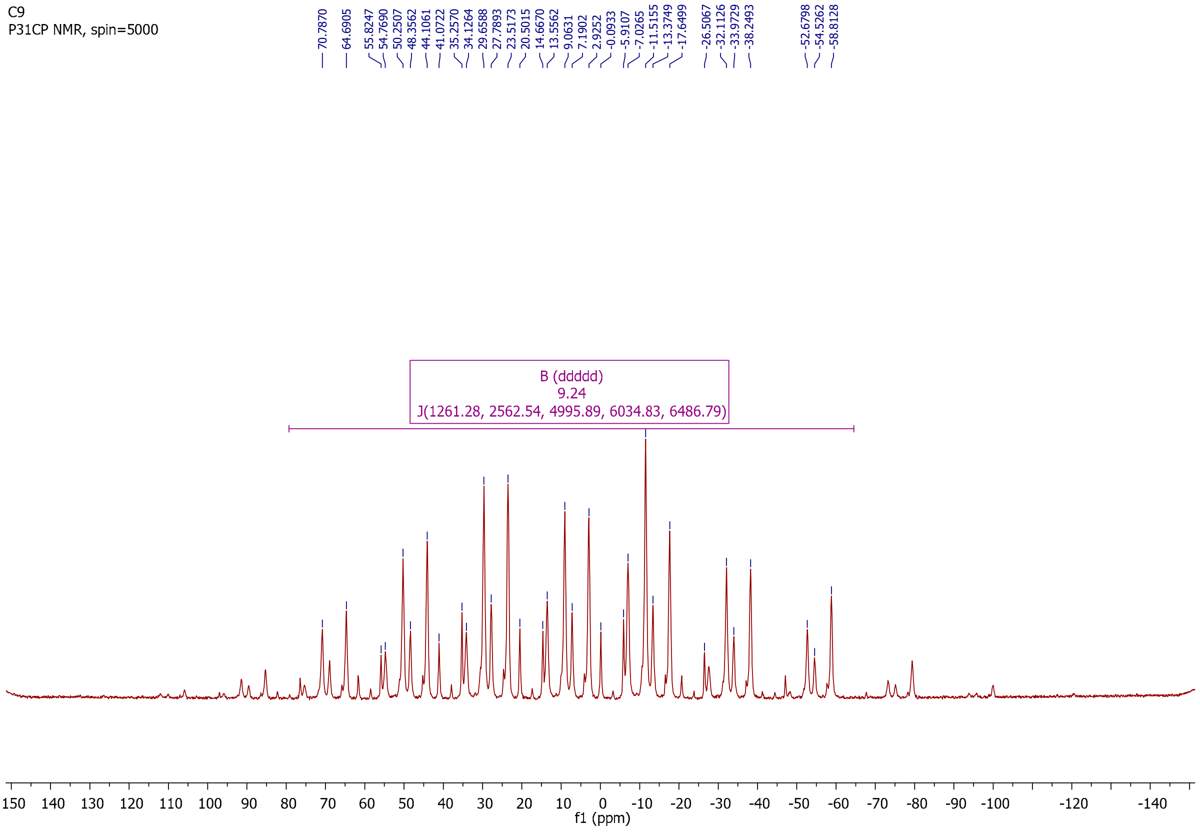

^{31}P NMR spectrum of compound **6**

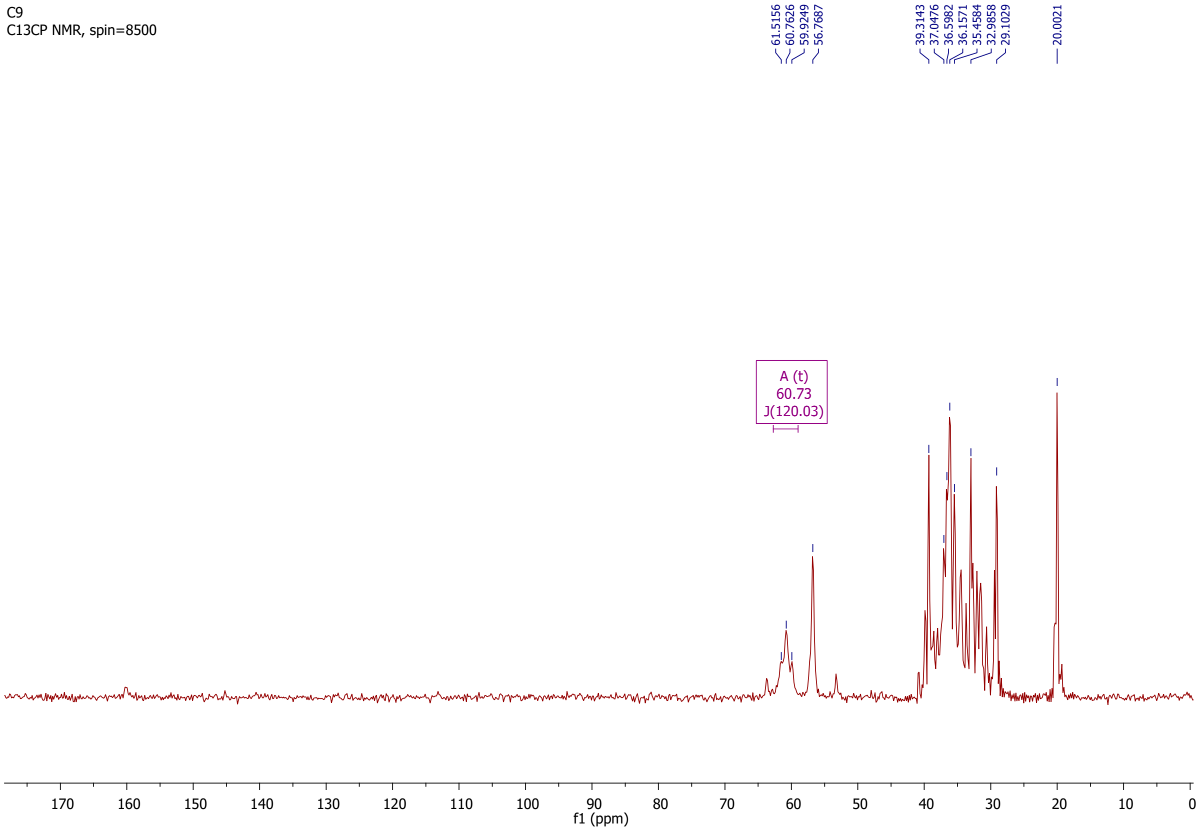

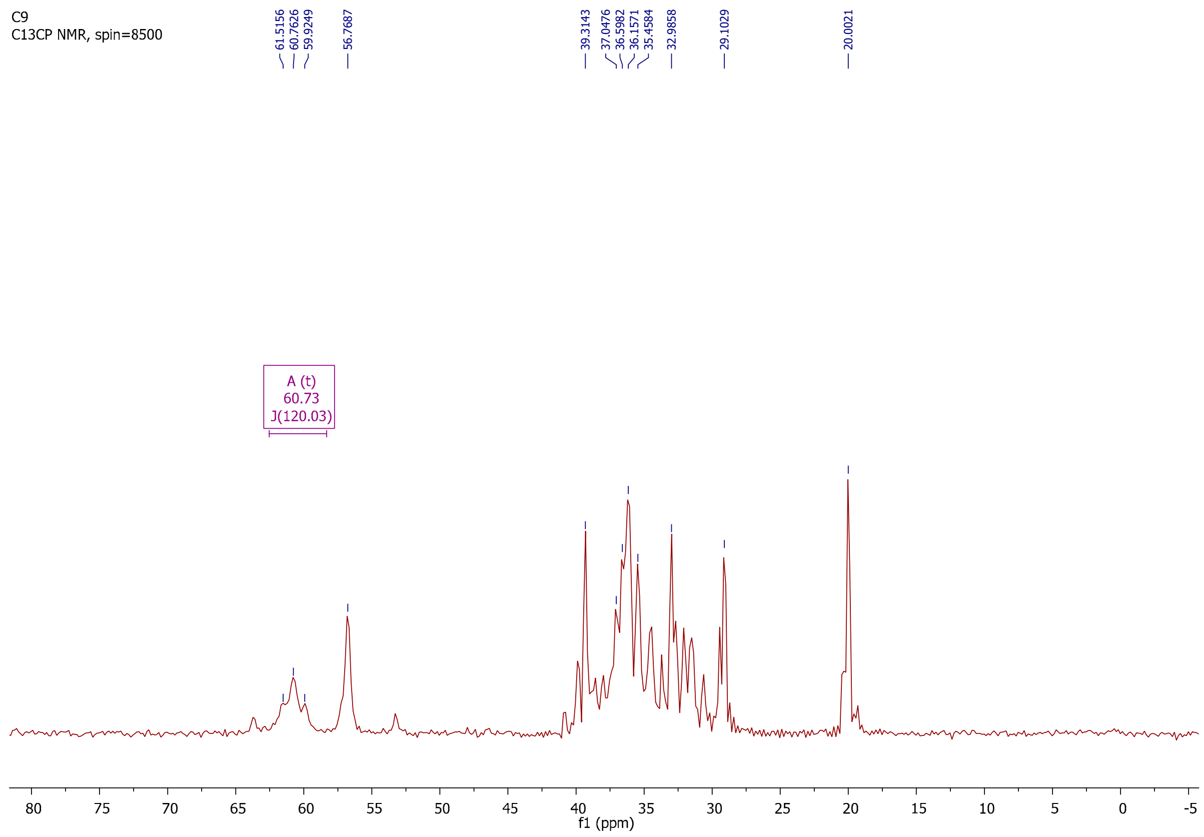

^{13}C NMR spectrum of compound **6**

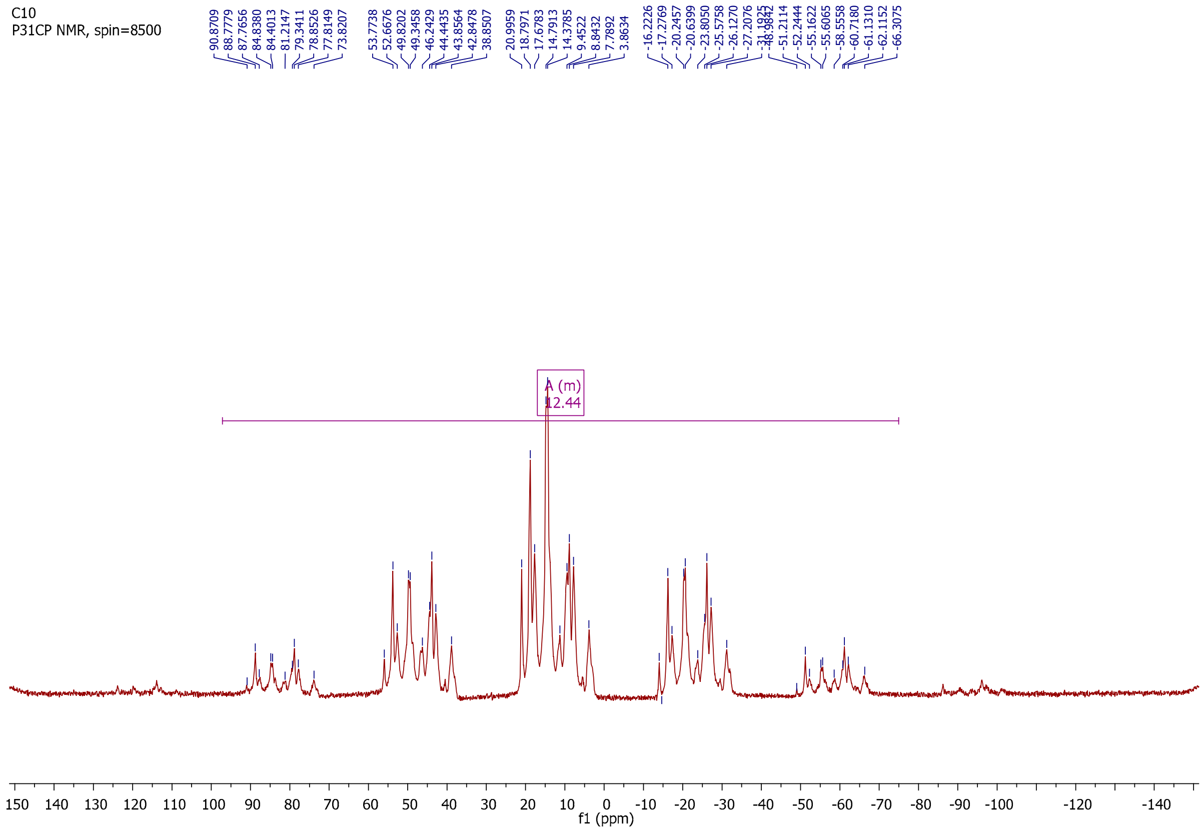

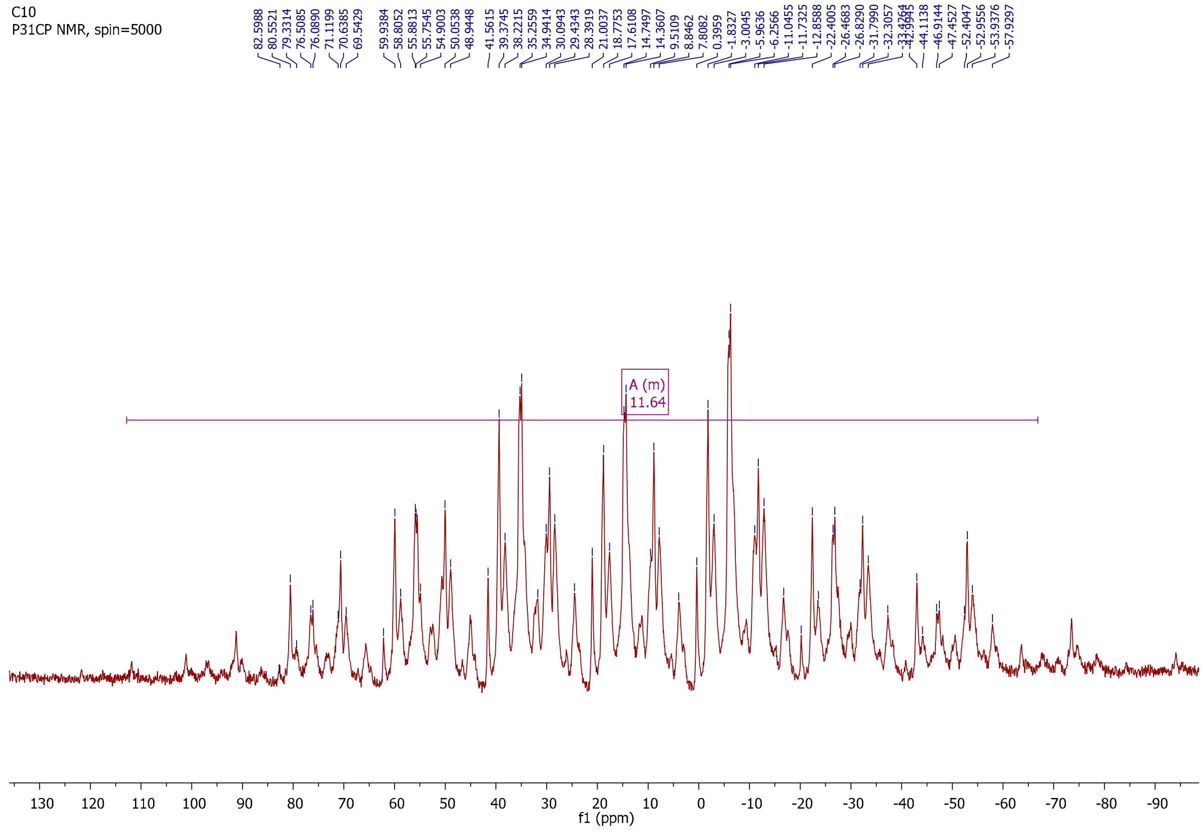

^{31}P NMR spectrum of compound **7**

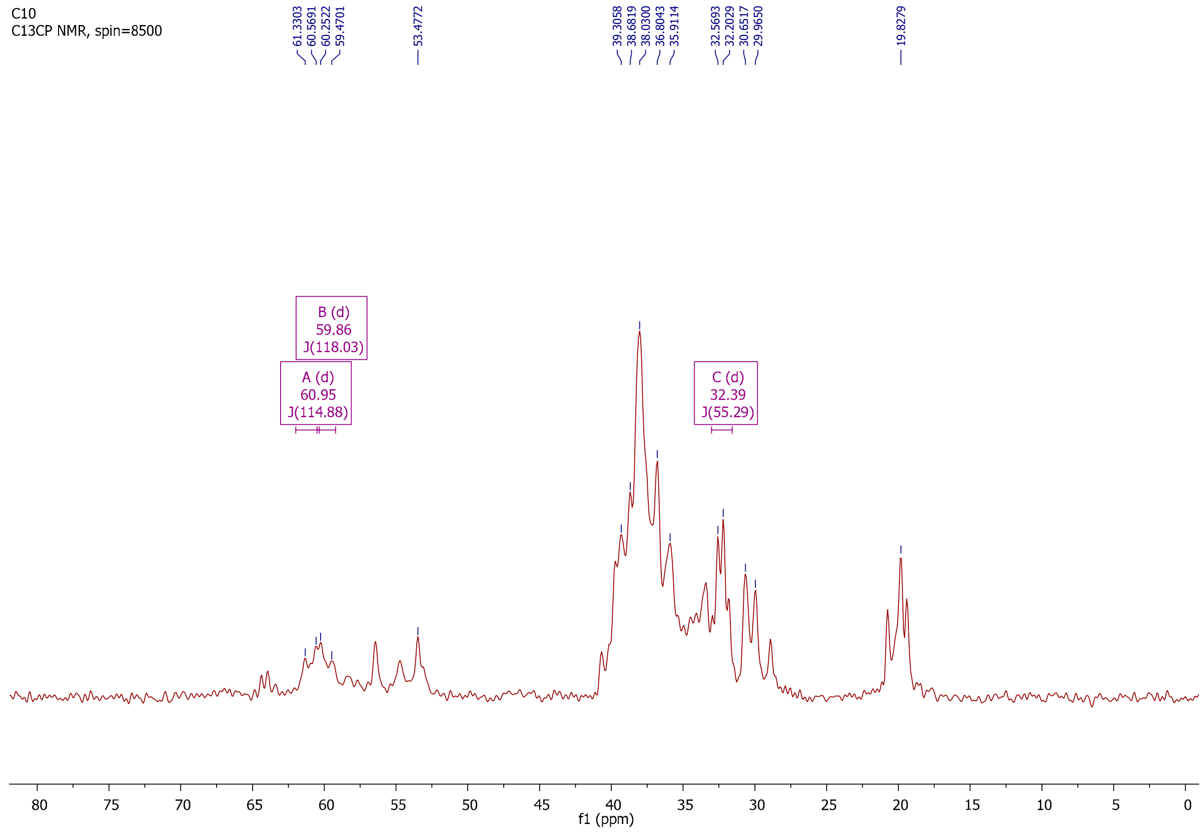

^{31}P NMR spectrum of compound **7**

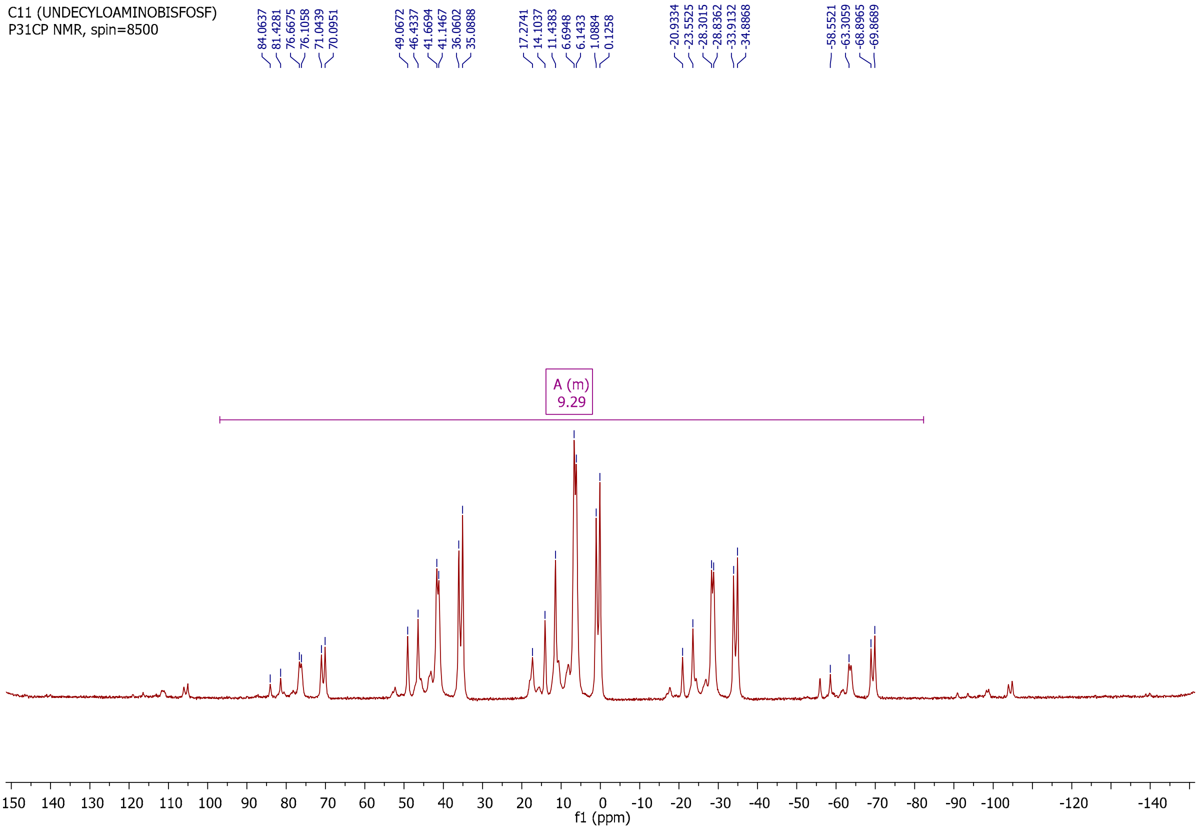

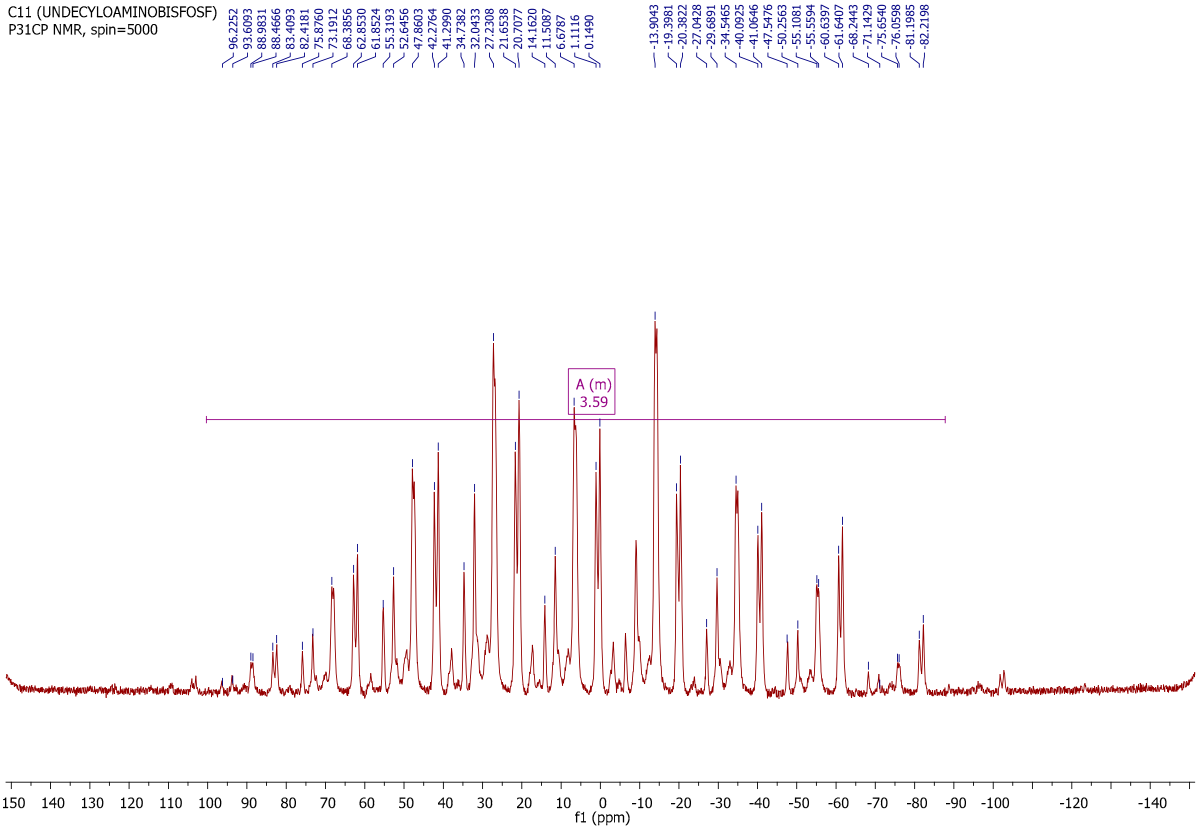

^{31}P NMR spectrum of compound **8**

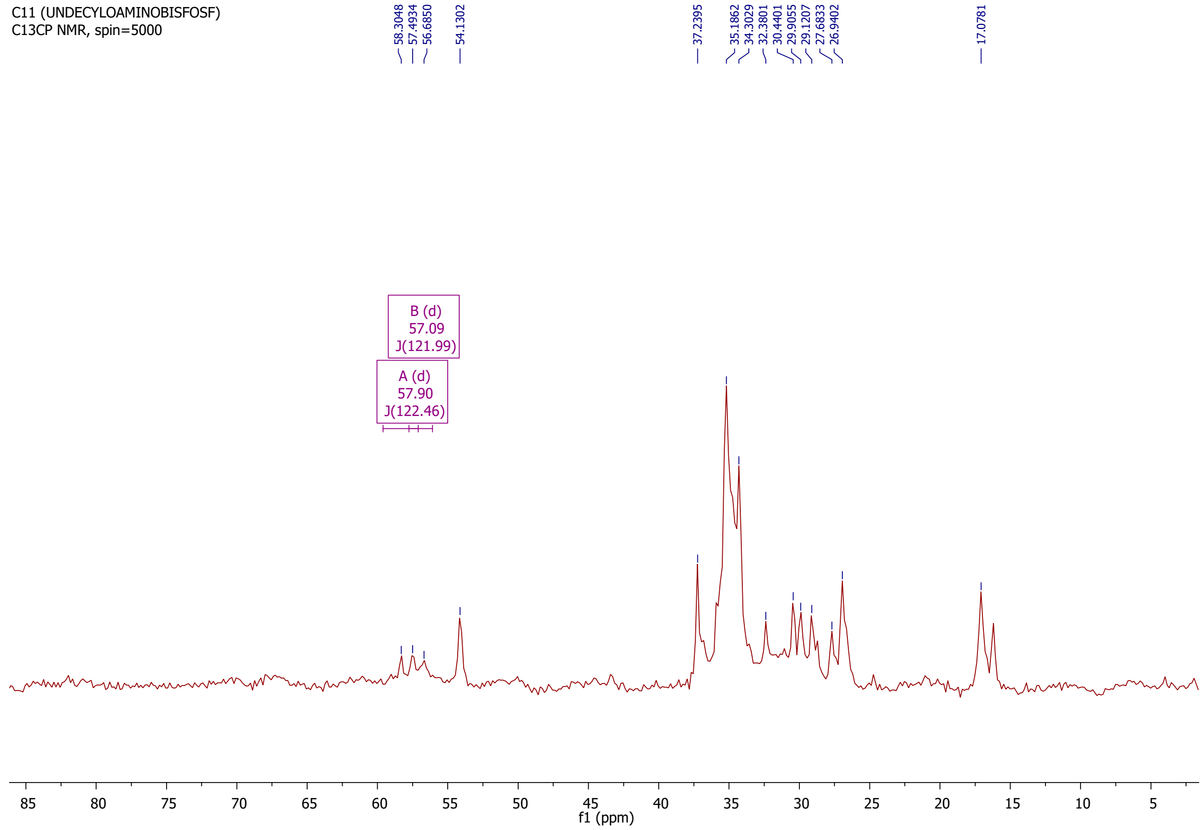

[13] C NMR spectrum of compound **8**

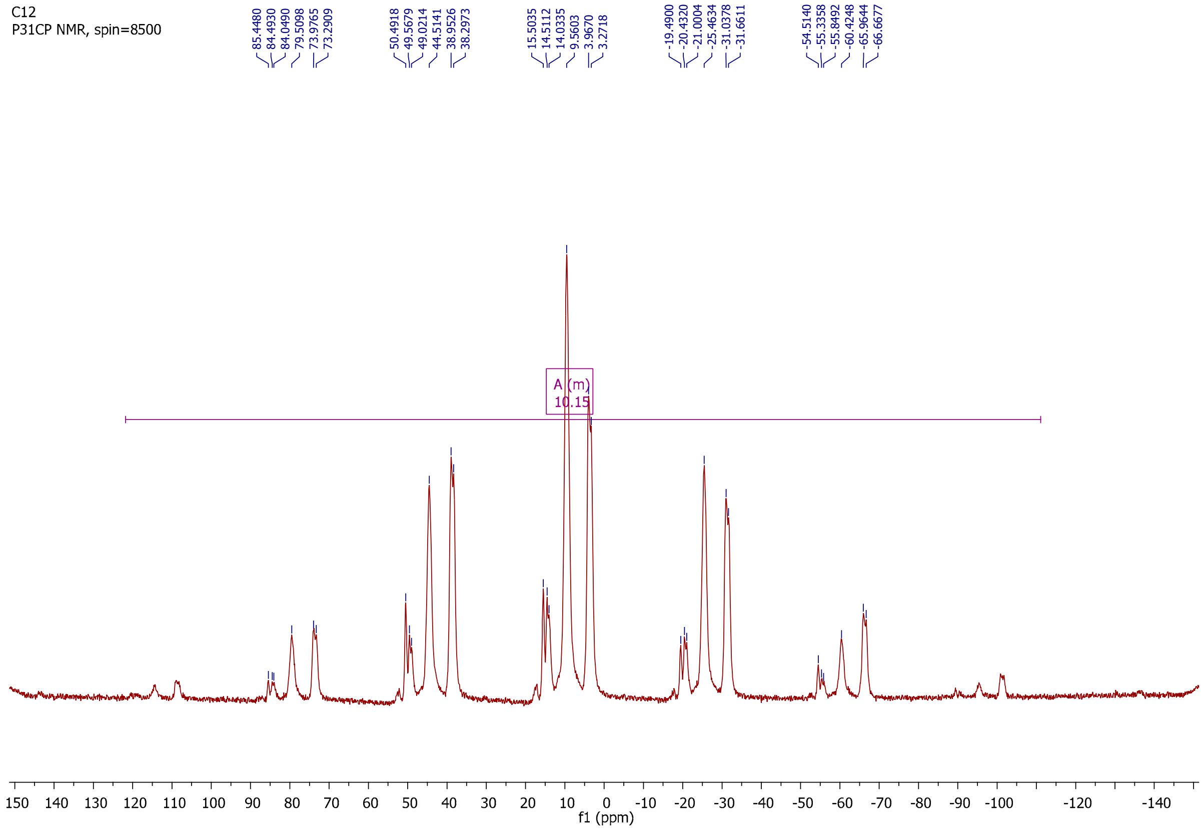

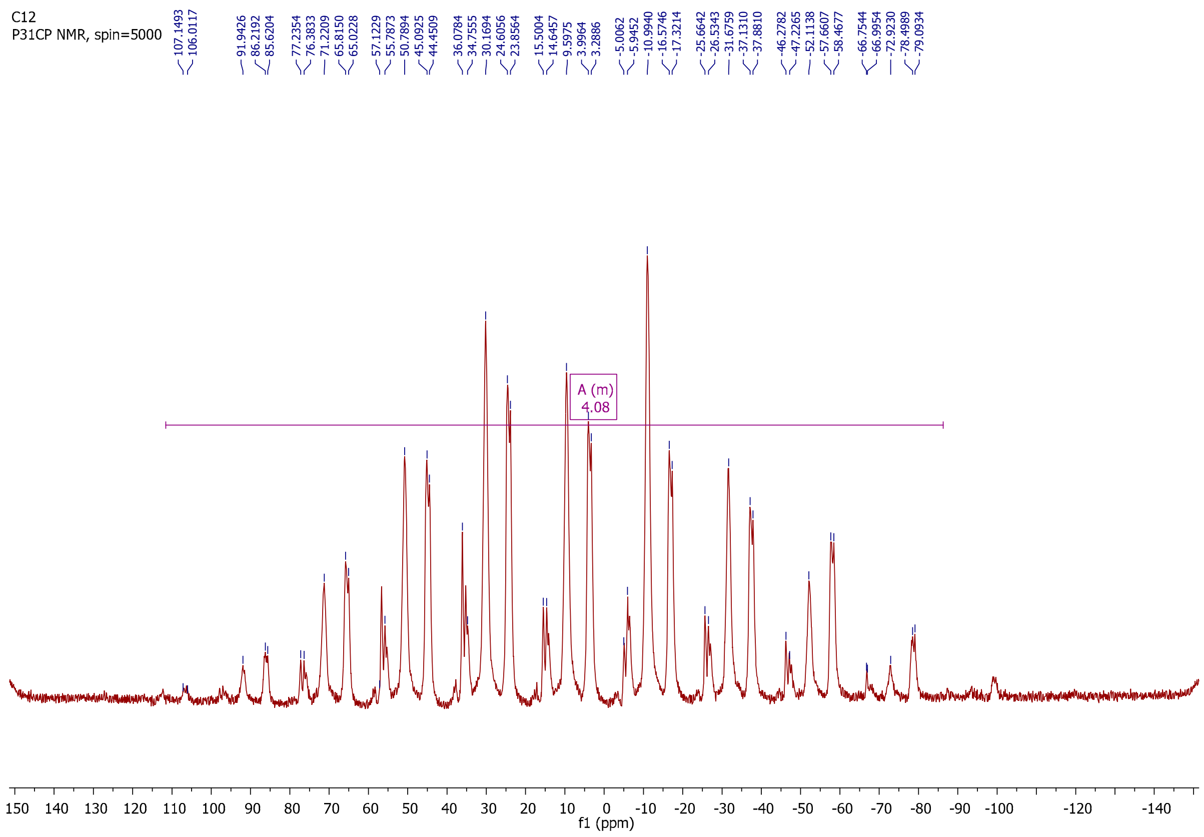

[31] P NMR spectrum of compound **9**

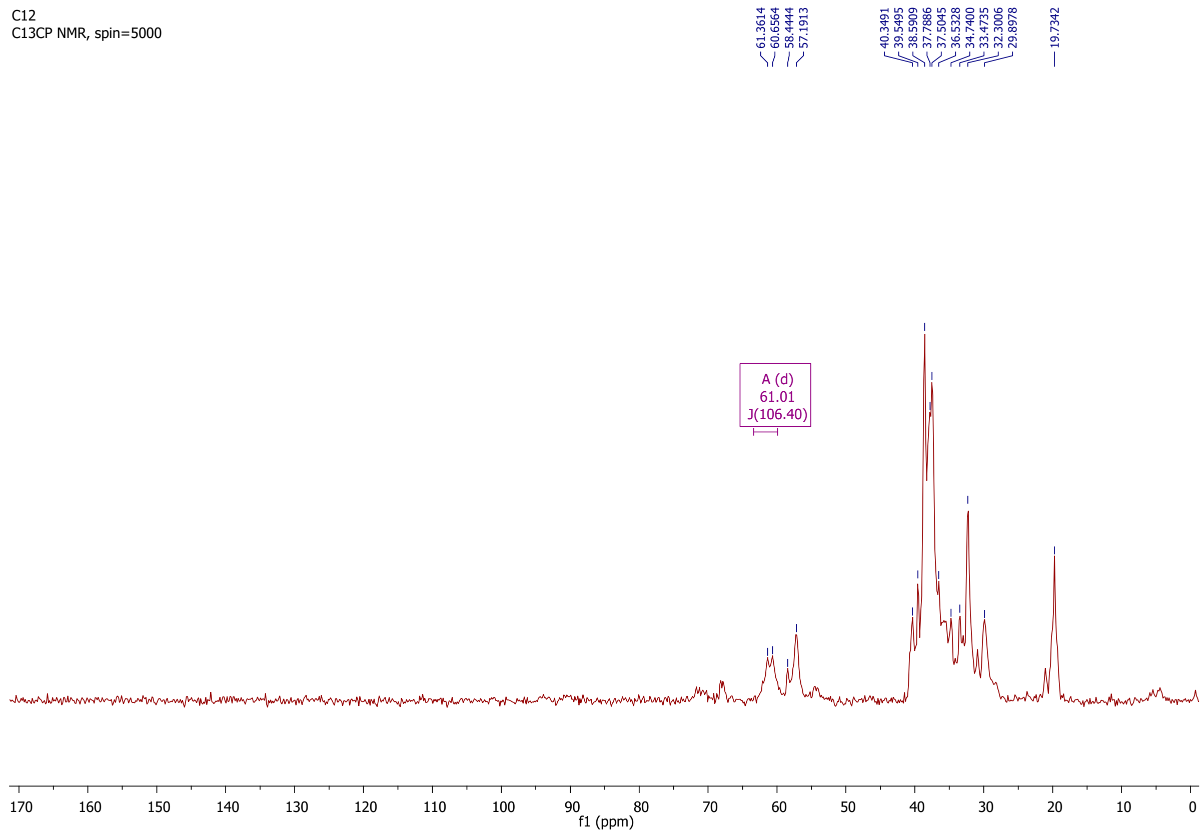

[13] C NMR spectrum of compound **9**

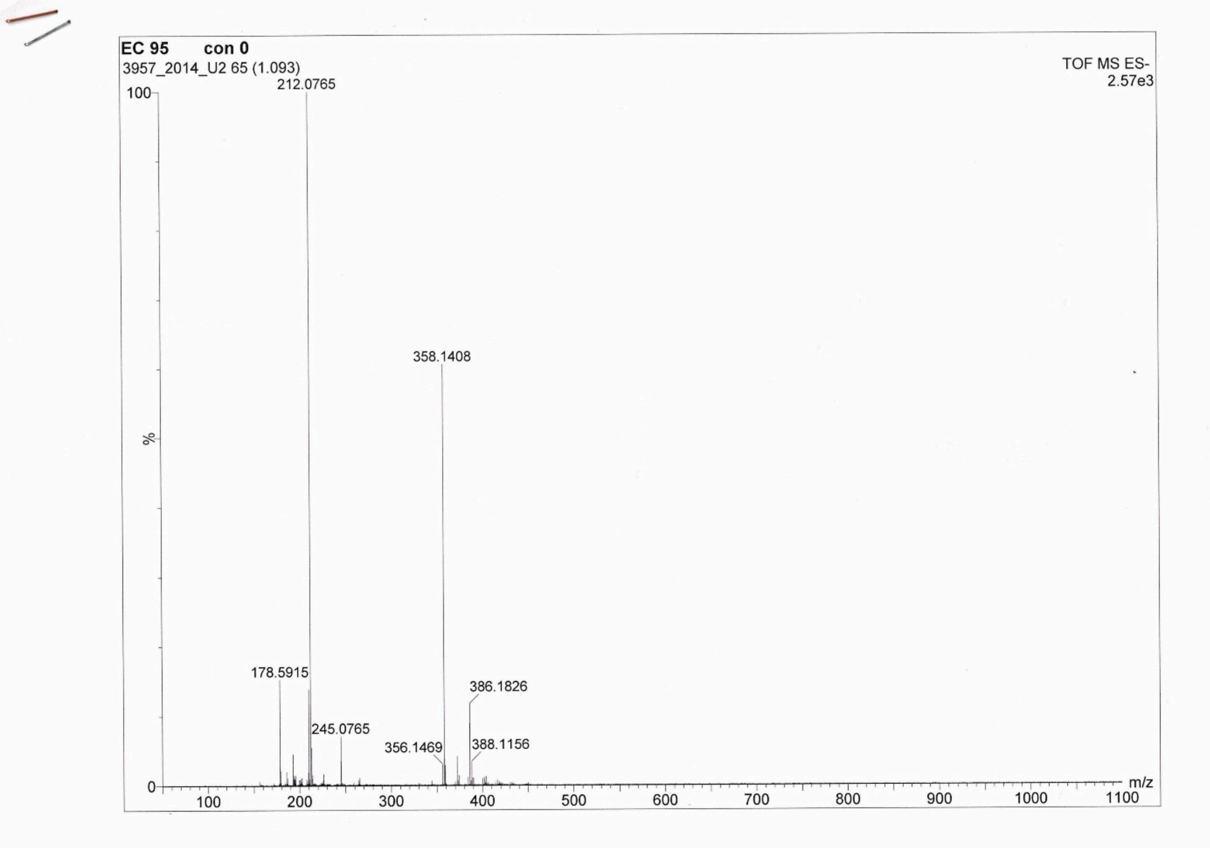

MS spectrum of compound **9**

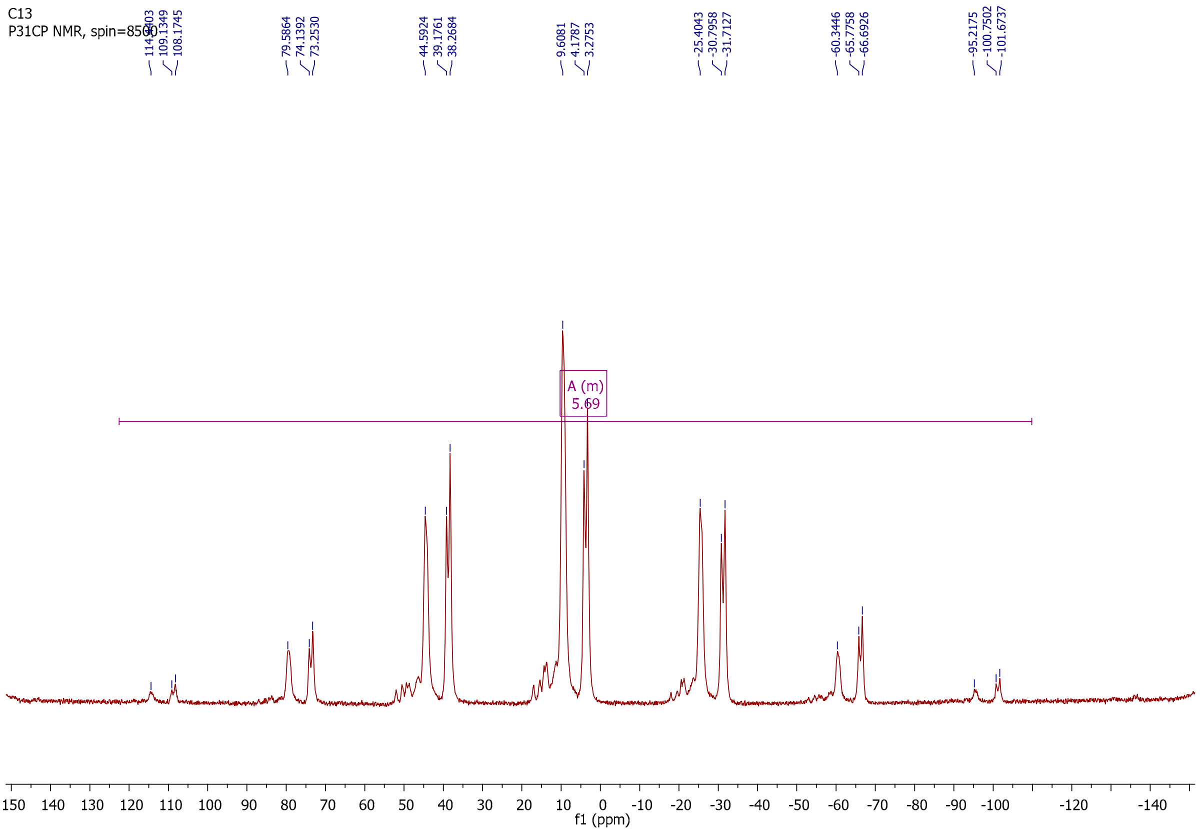

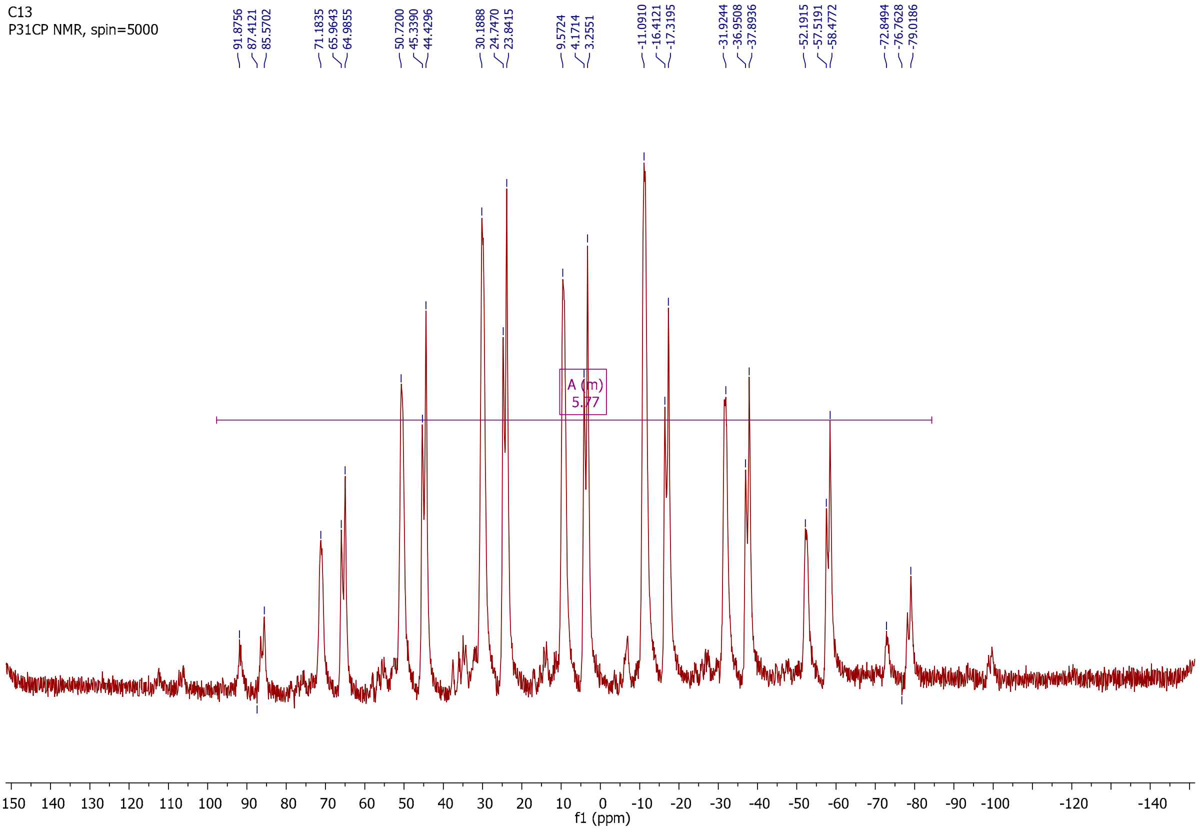

[31] P NMR spectrum of compound **10**

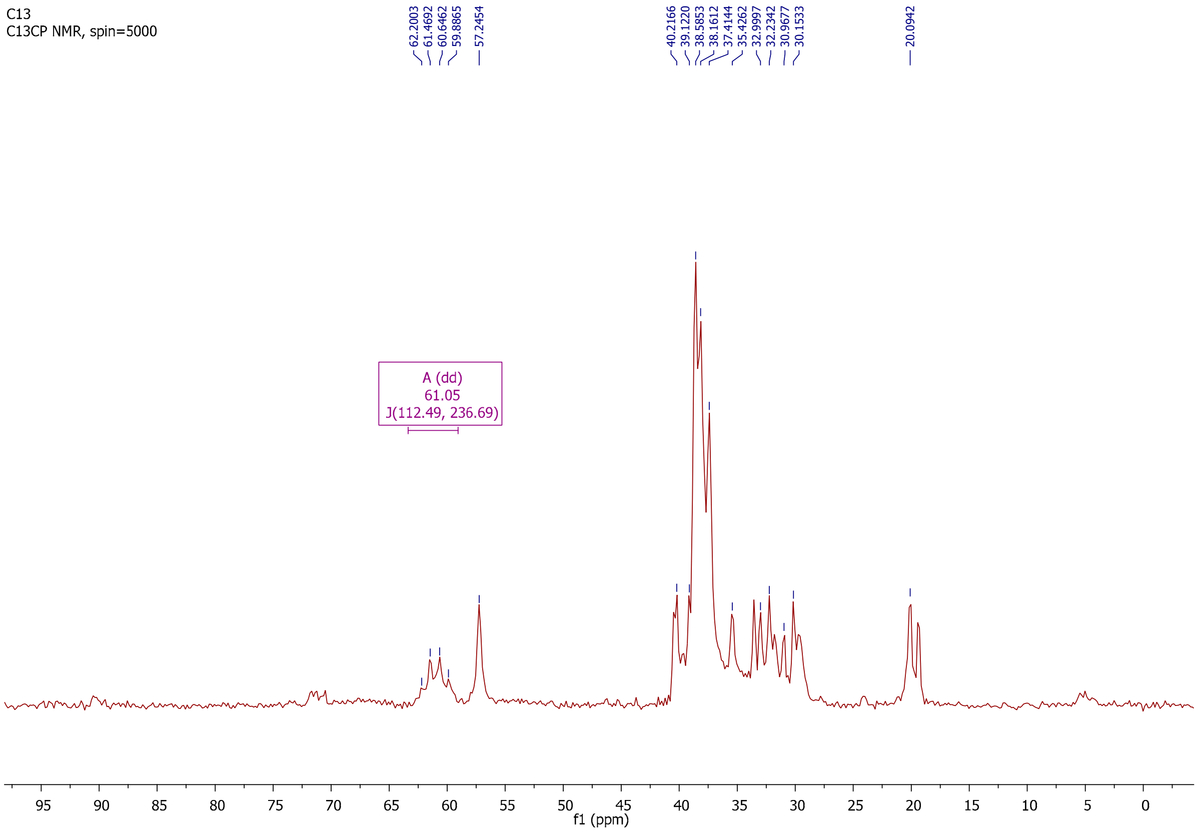

[13] C NMR spectrum of compound **10**

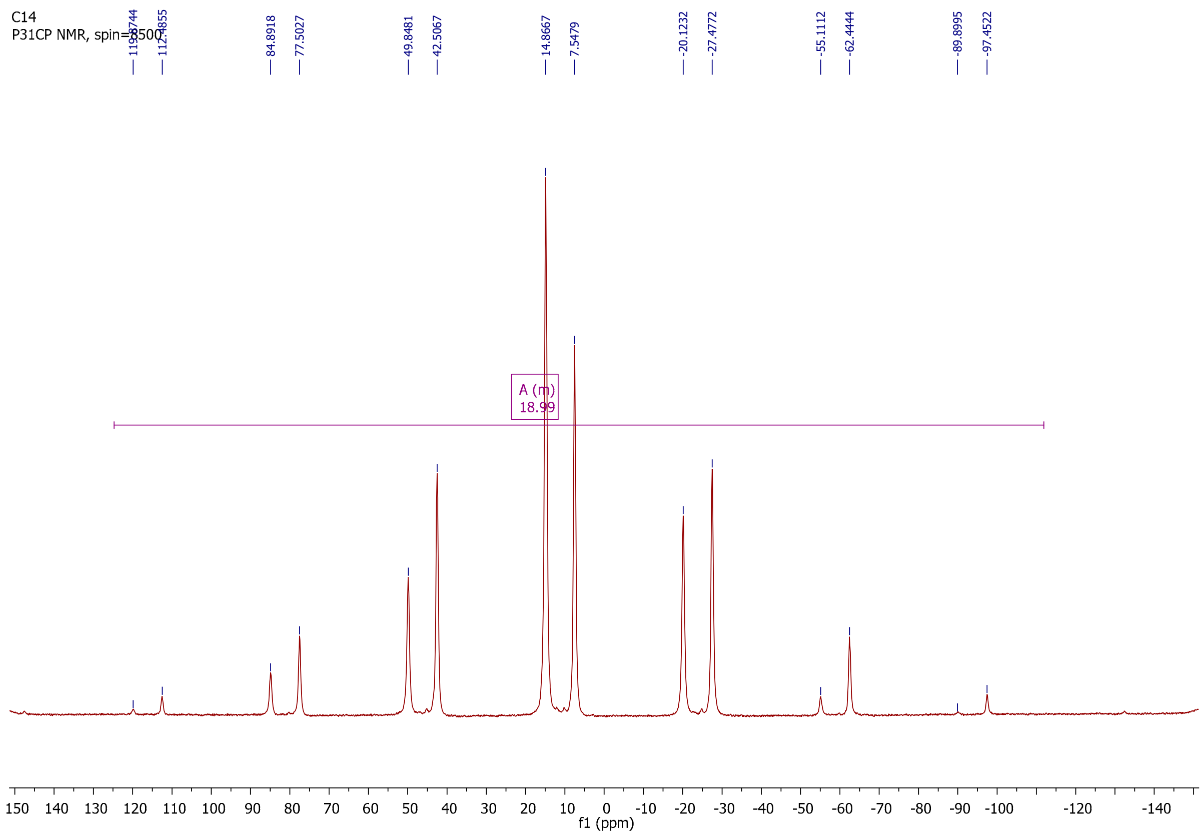

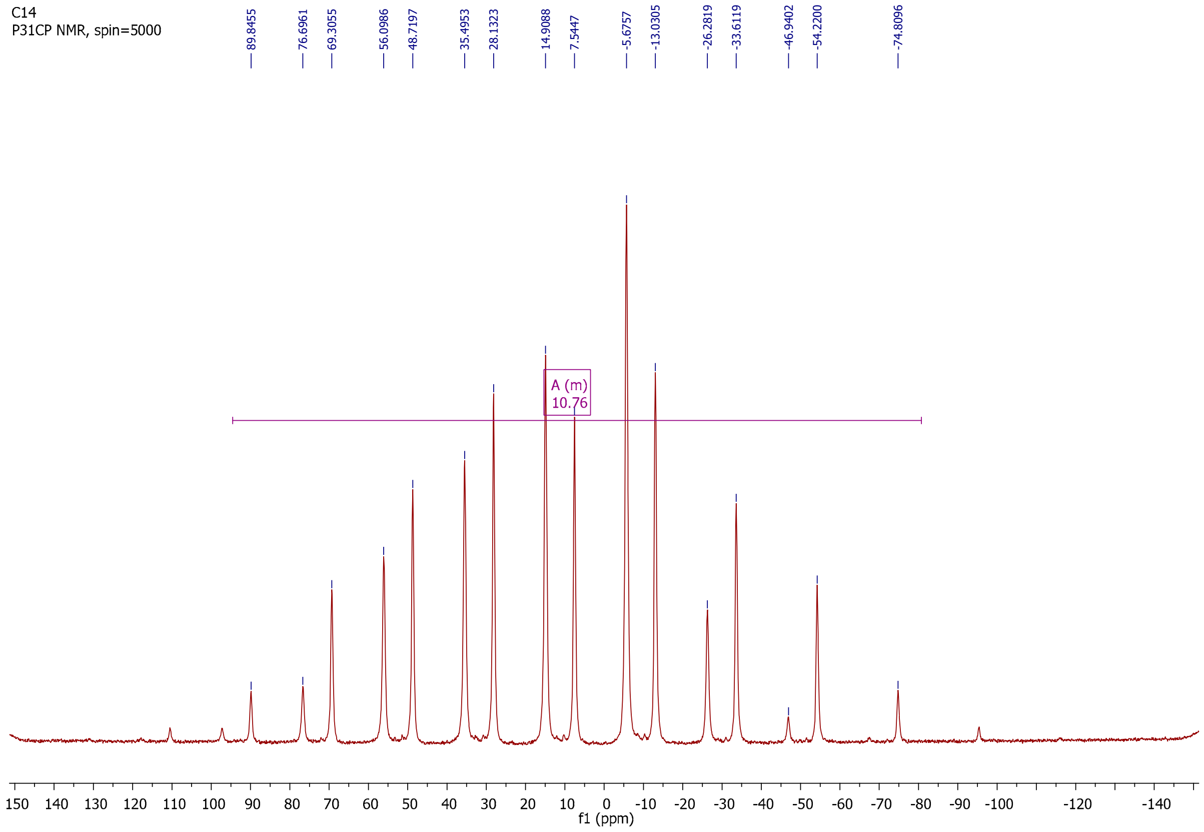

^{31}P NMR spectrum of compound **11**

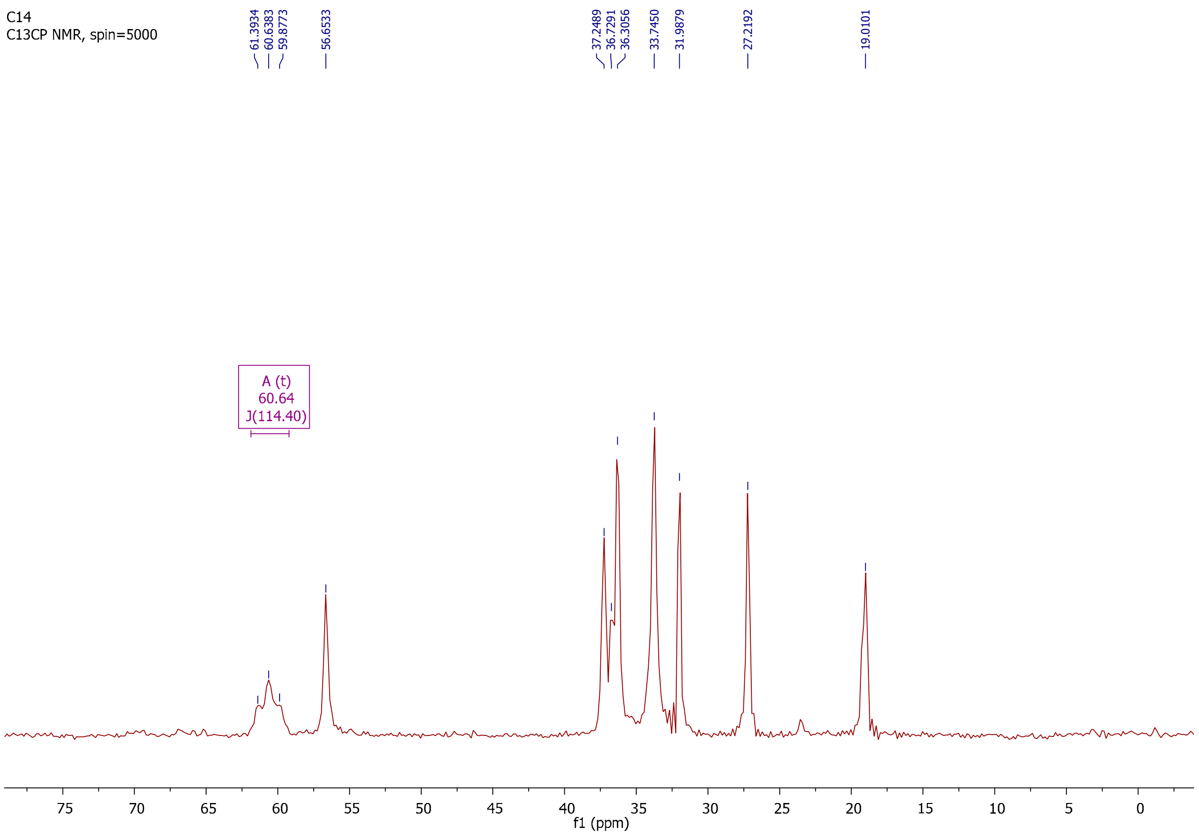

^{13}C NMR spectrum of compound **11**

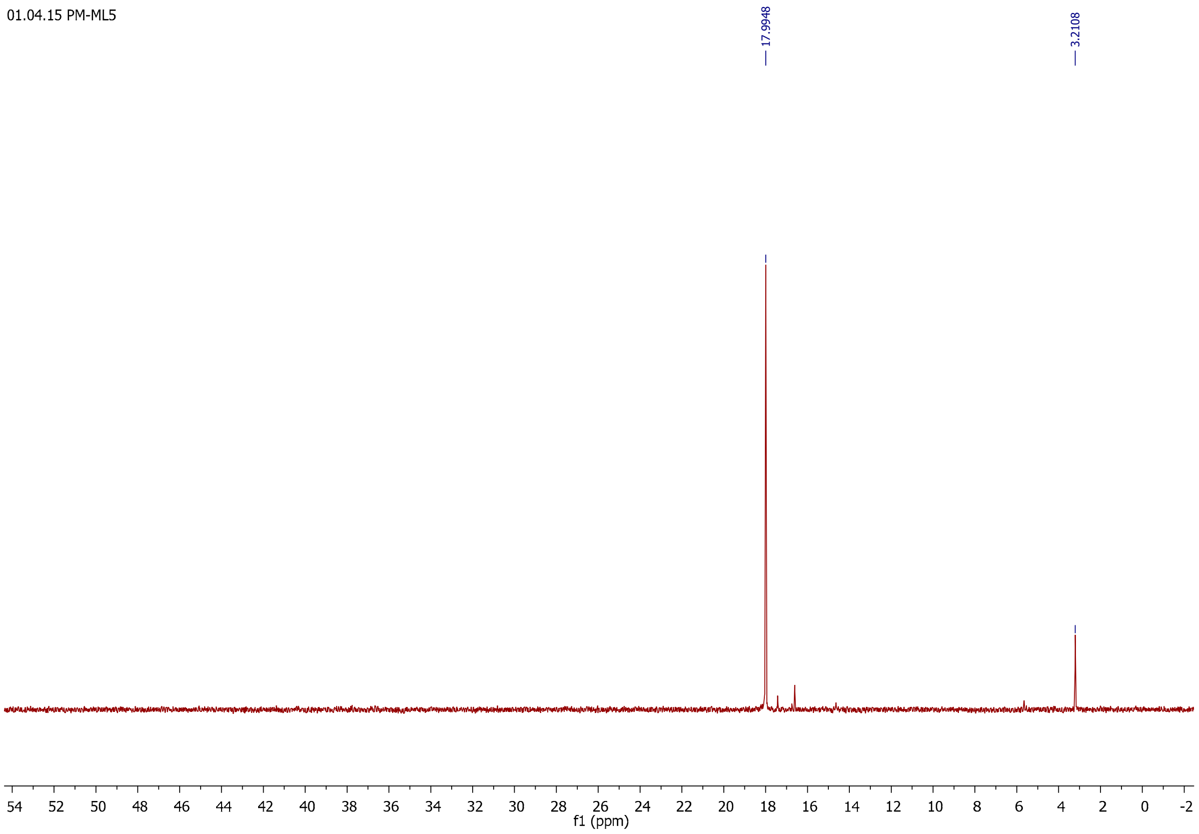

^{31}P NMR spectrum of compound **12**

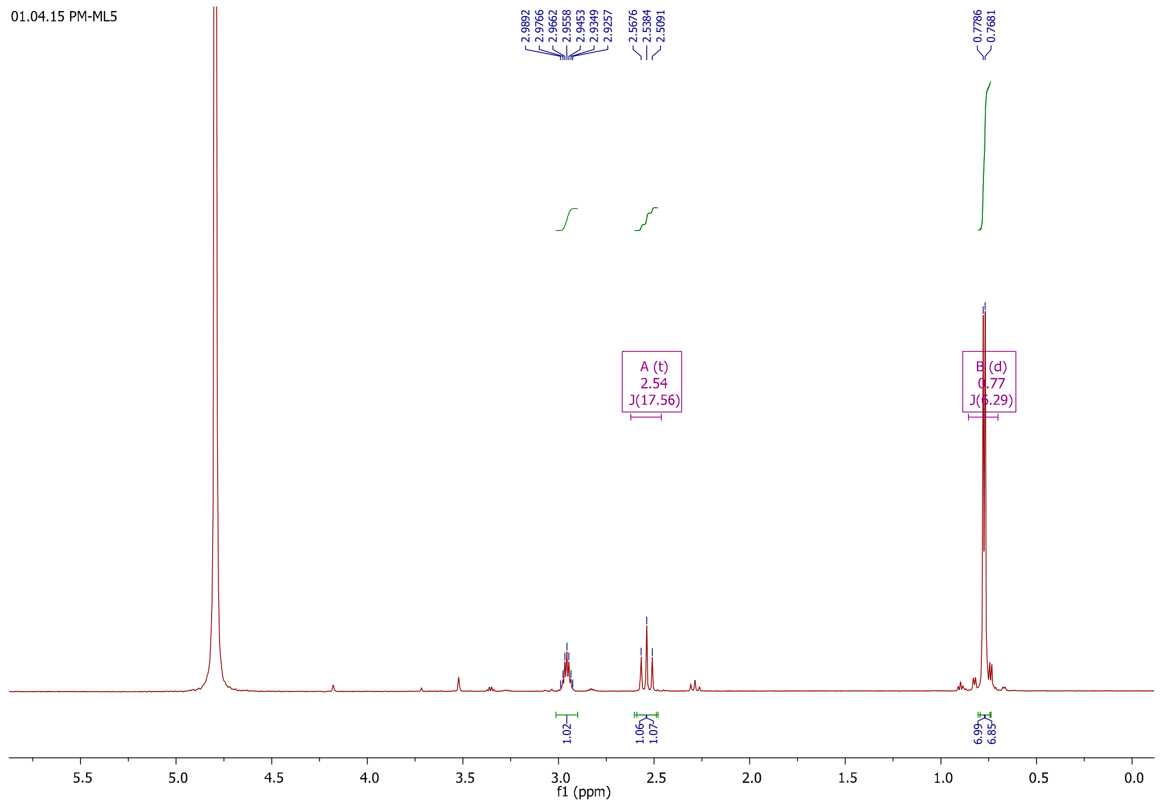

^{1}H NMR spectrum of compound **12**

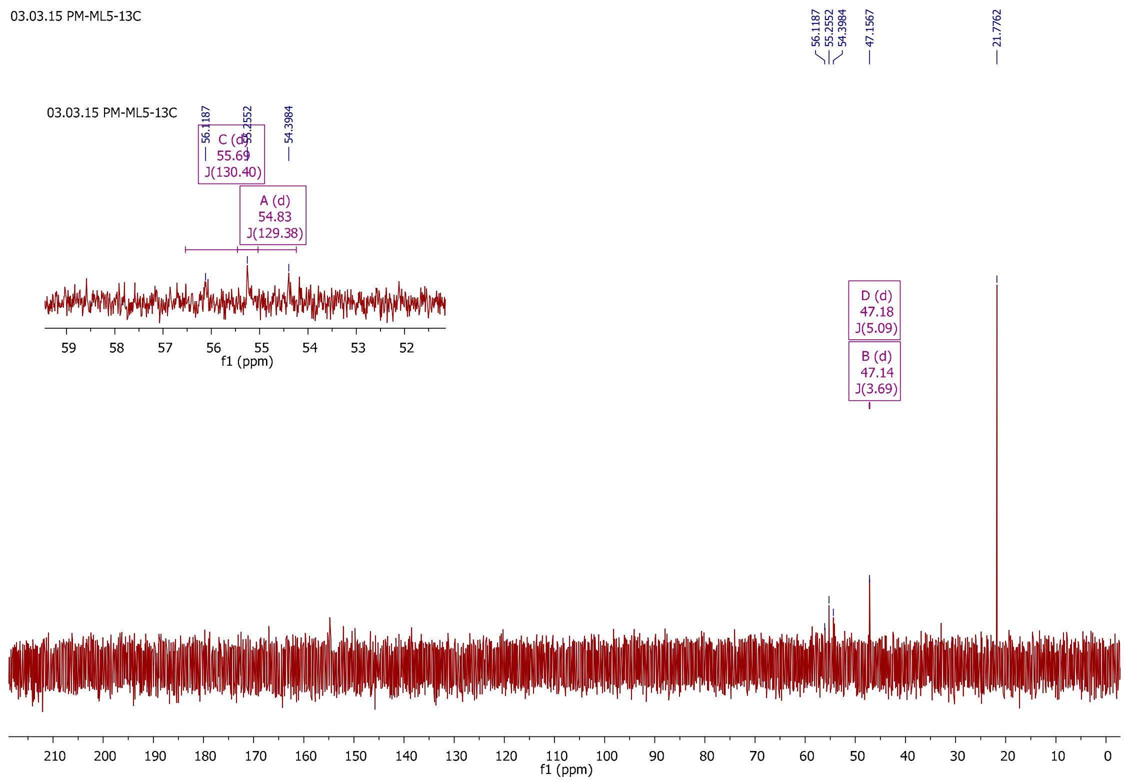

^{13}C NMR spectrum of compound **12**

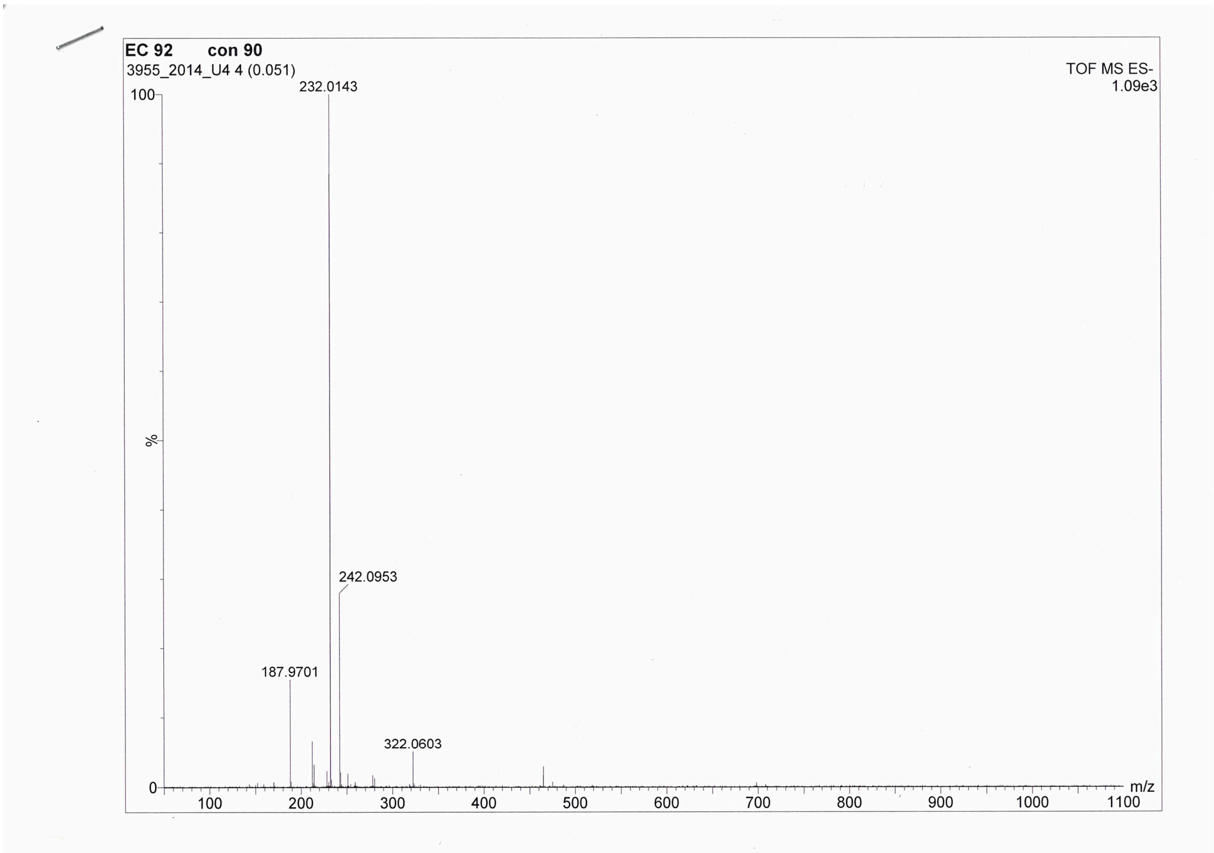

MS spectrum of compound **12**

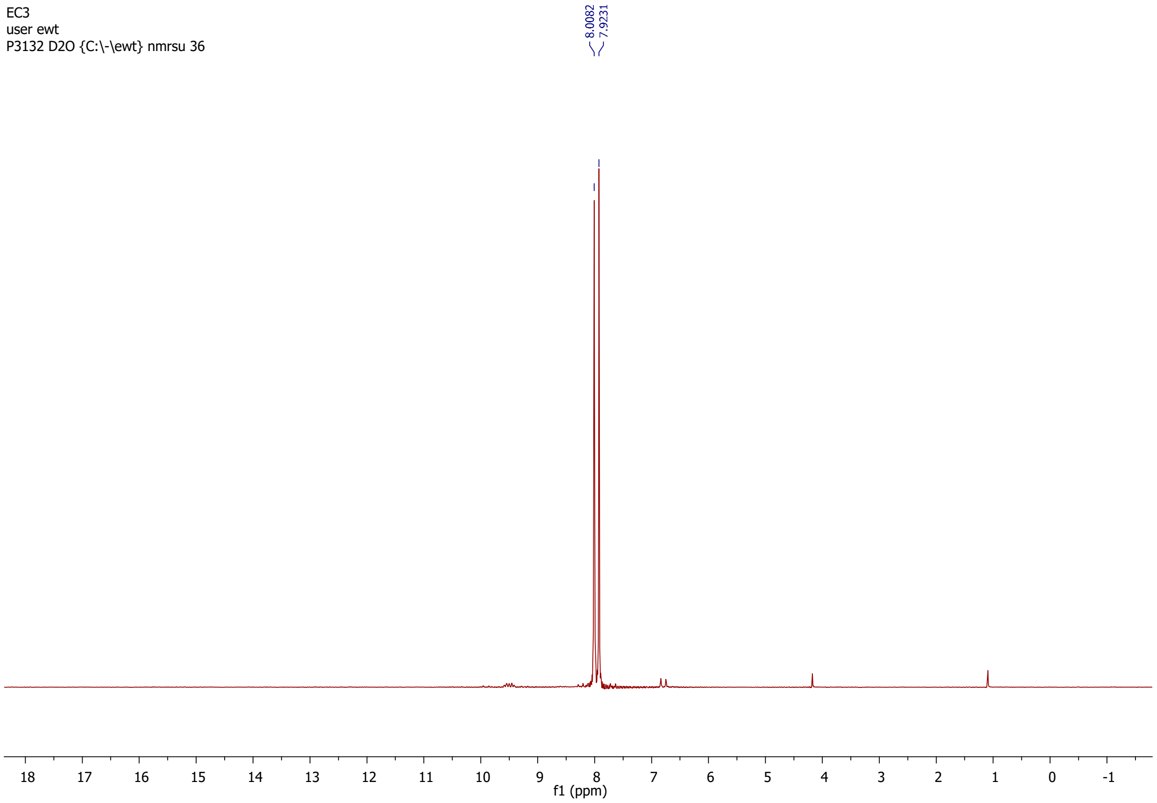

^{31}P NMR spectrum of compound **13**

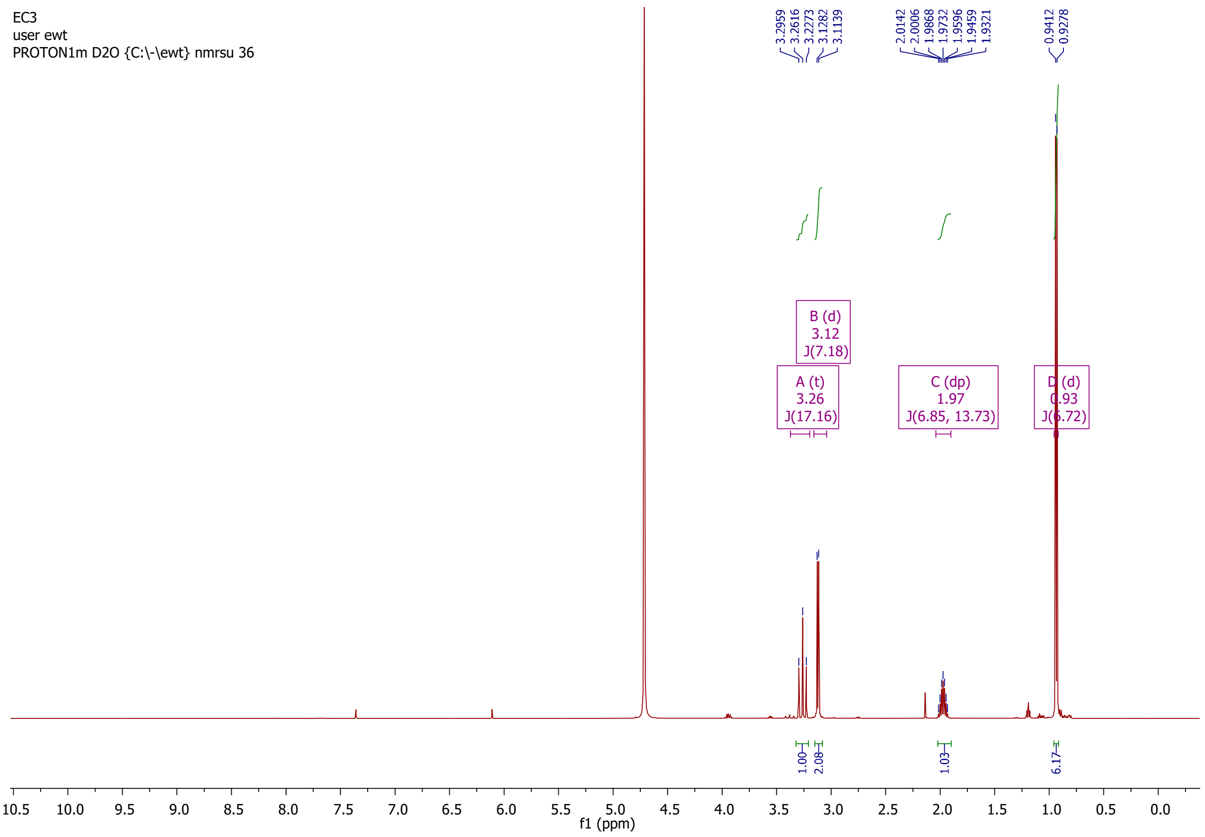

^{1}H NMR spectrum of compound **13**

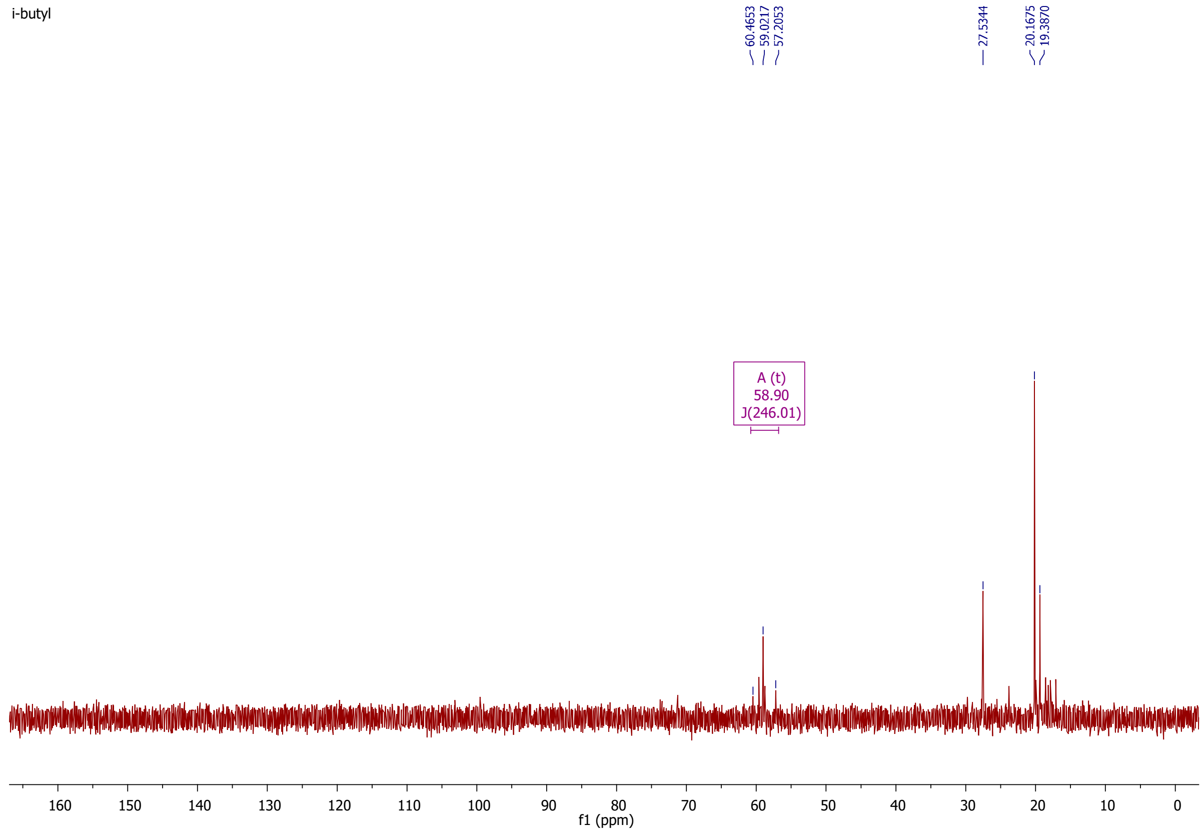

^{13}C NMR spectrum of compound **13**

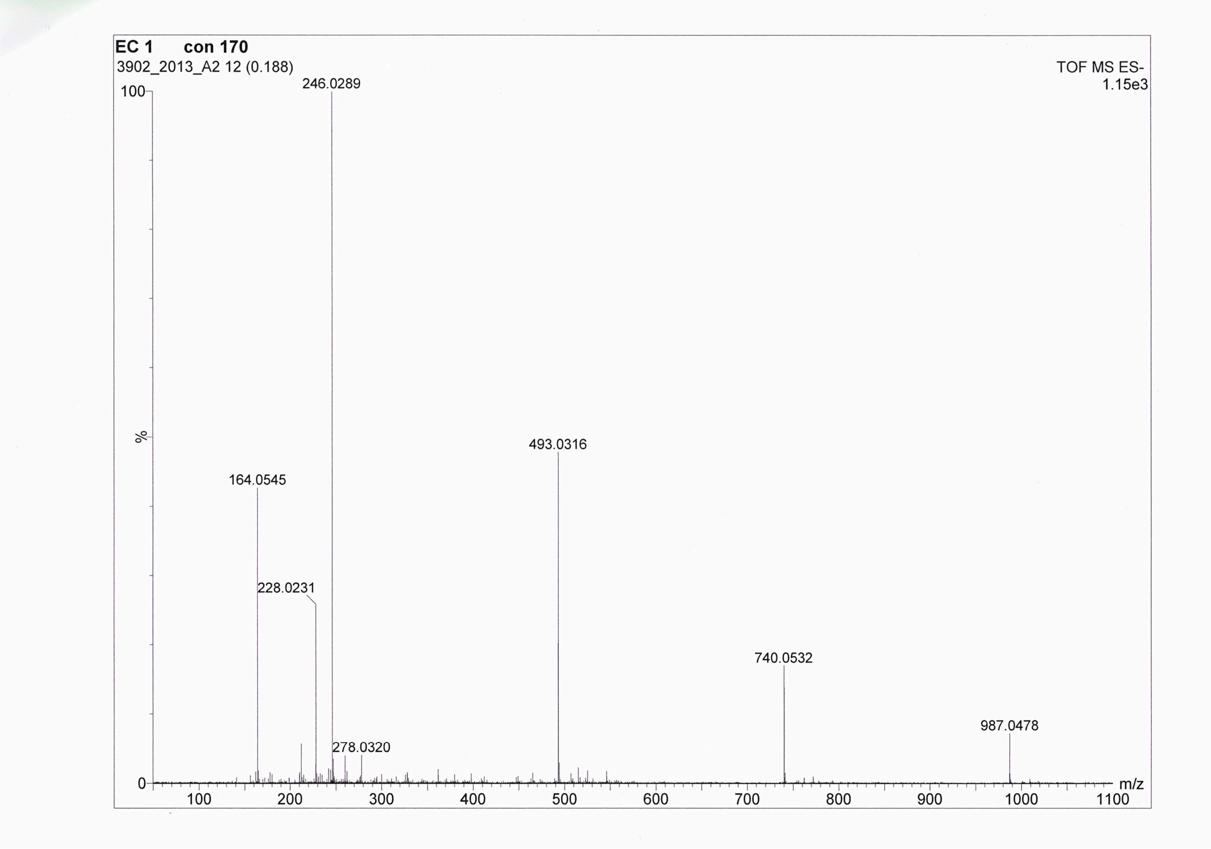

MS spectrum of compound **13**

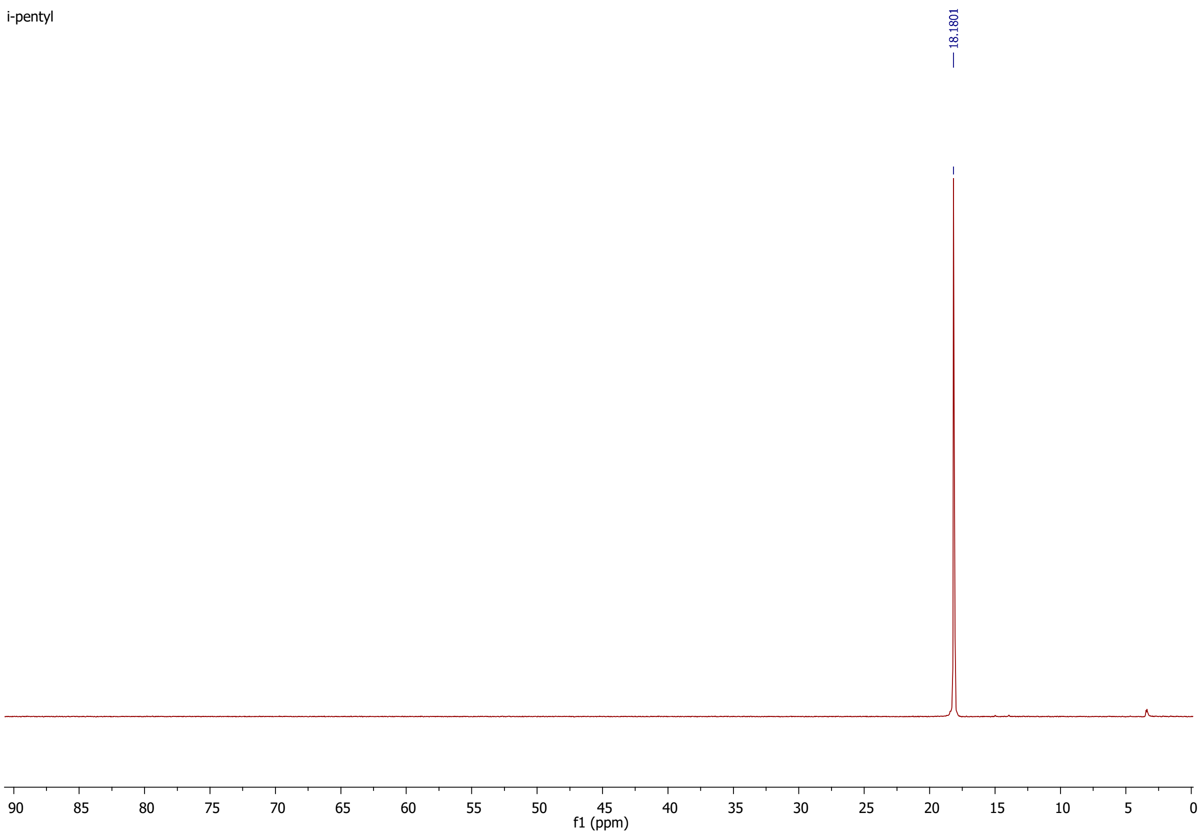

^{31}P NMR spectrum of compound **14**

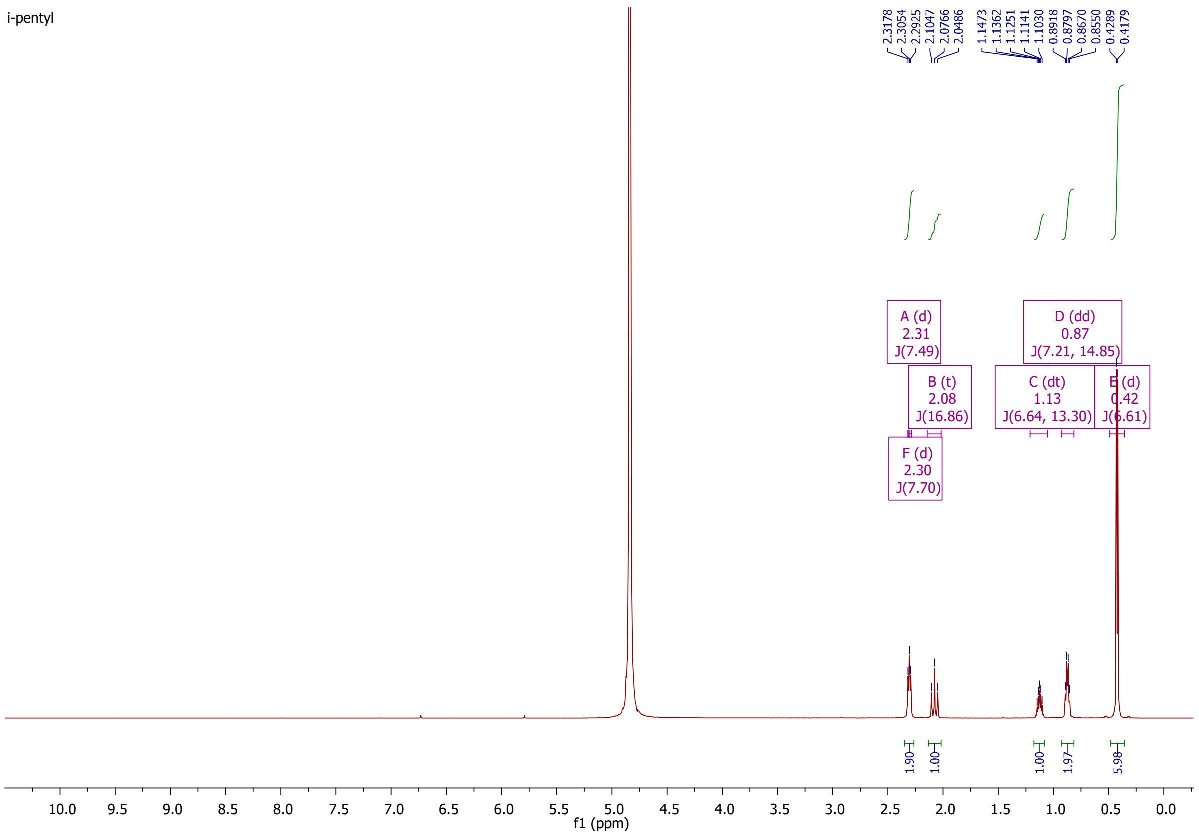

^{1}H NMR spectrum of compound **14**

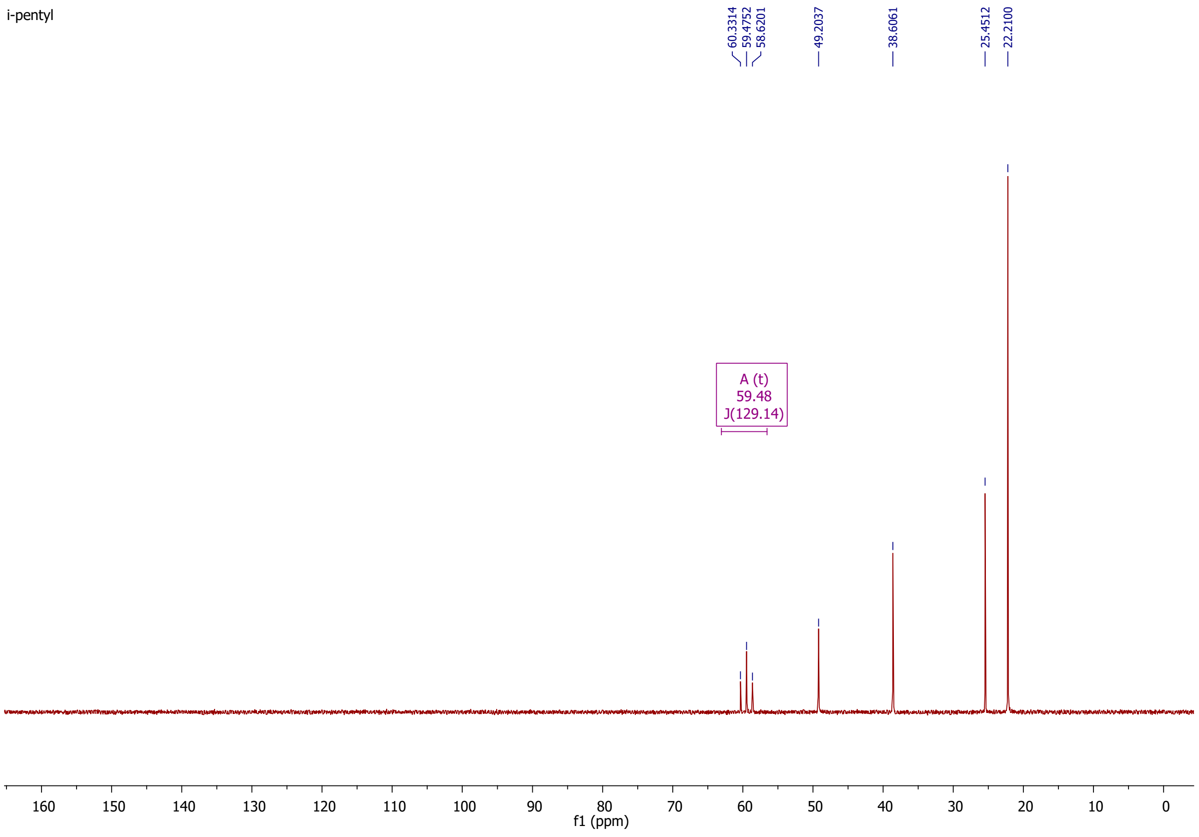

^{13}C NMR spectrum of compound **14**

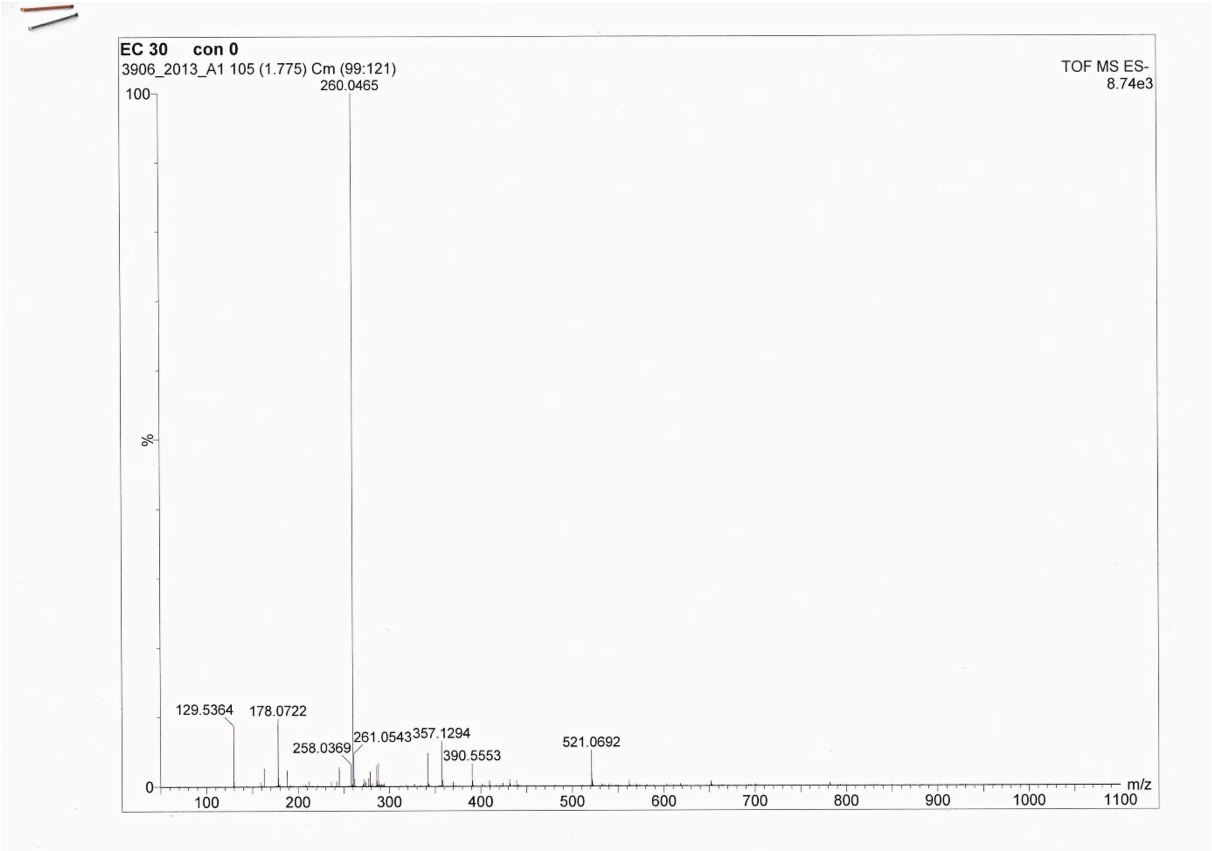

MS spectrum of compound **14**

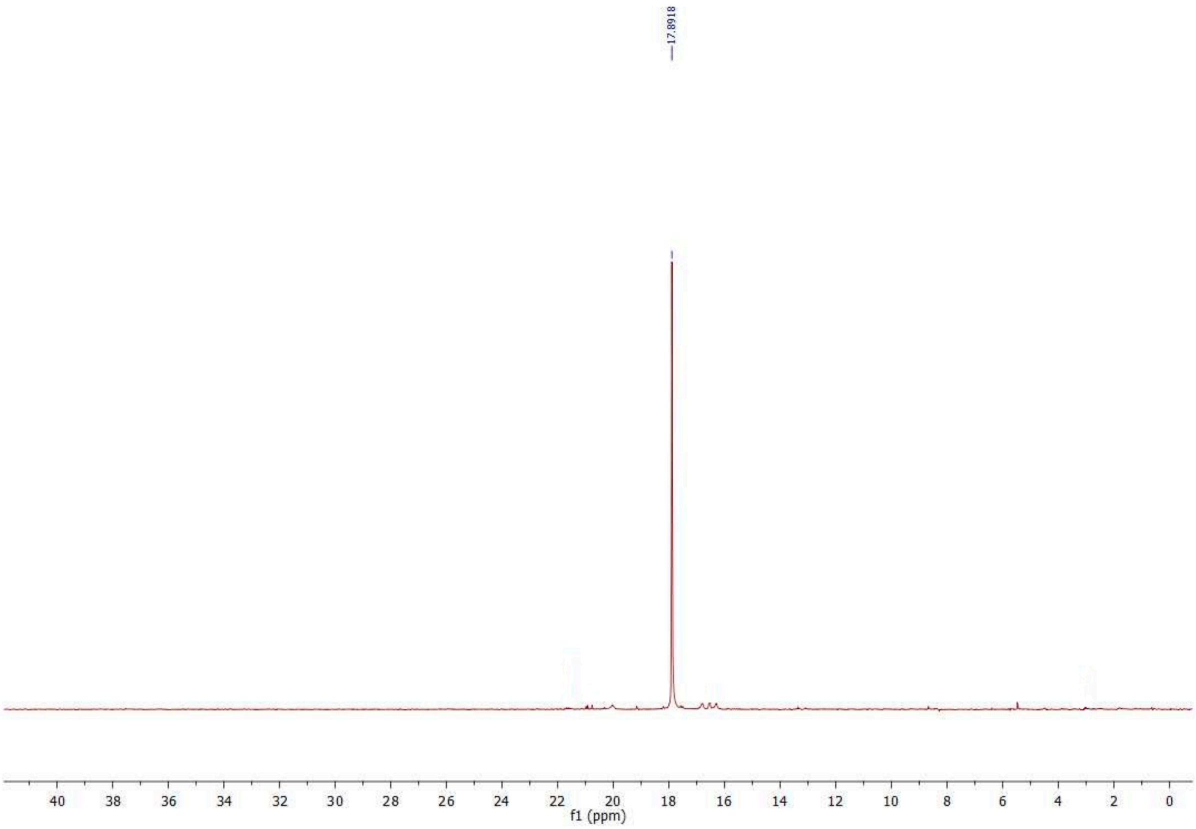

^{31}P NMR spectrum of compound **15**

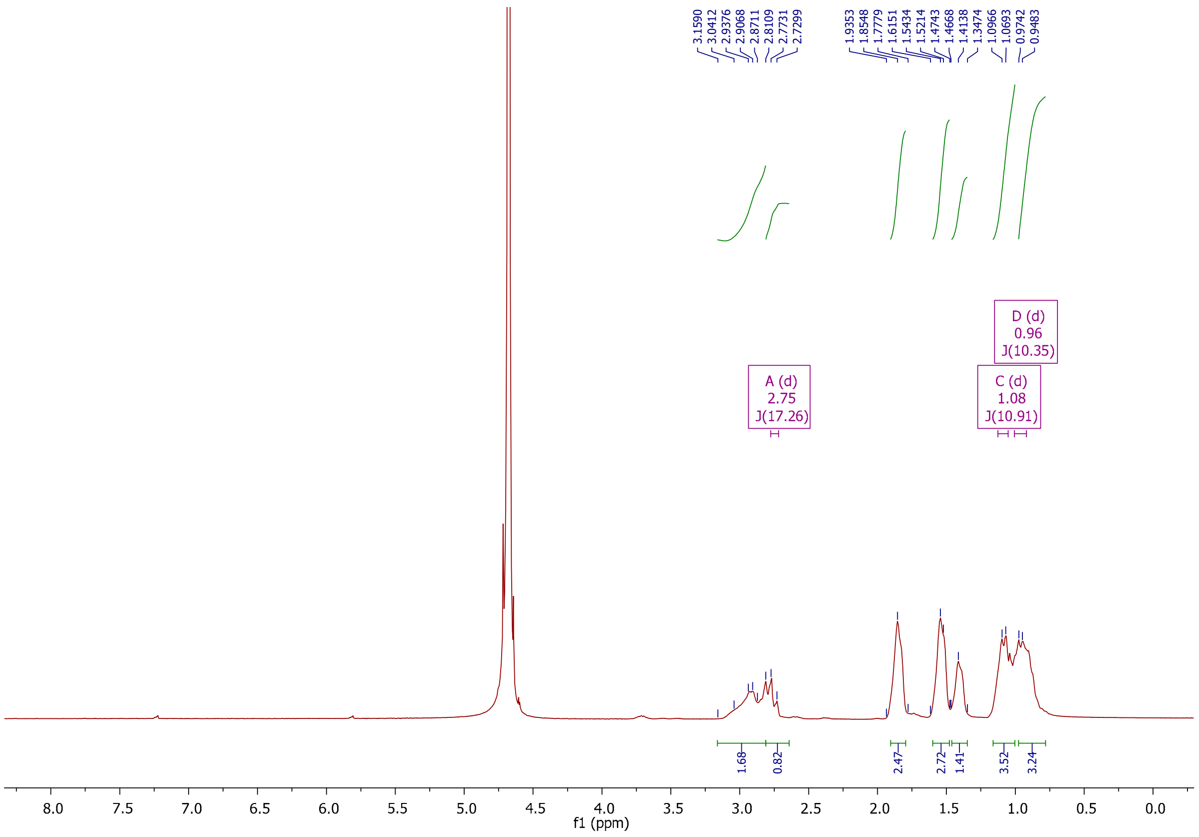

^{1}H NMR spectrum of compound **15**

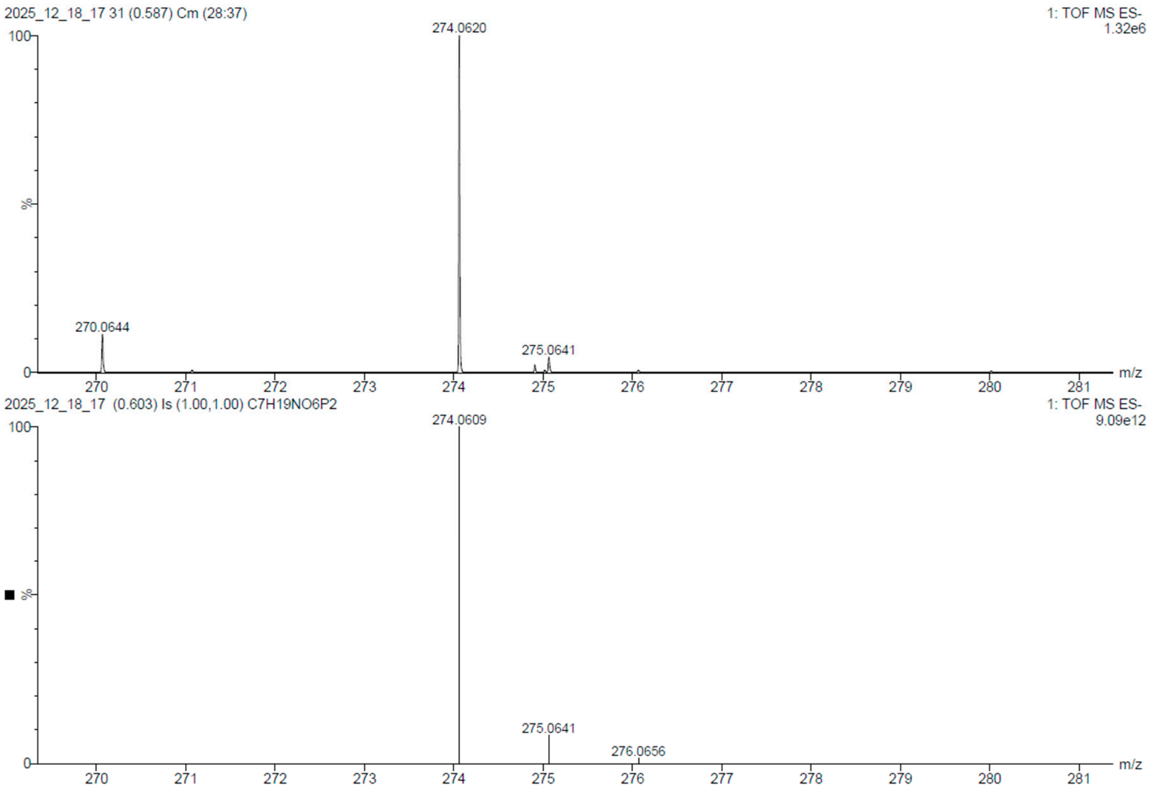

MS spectrum of compound **15**

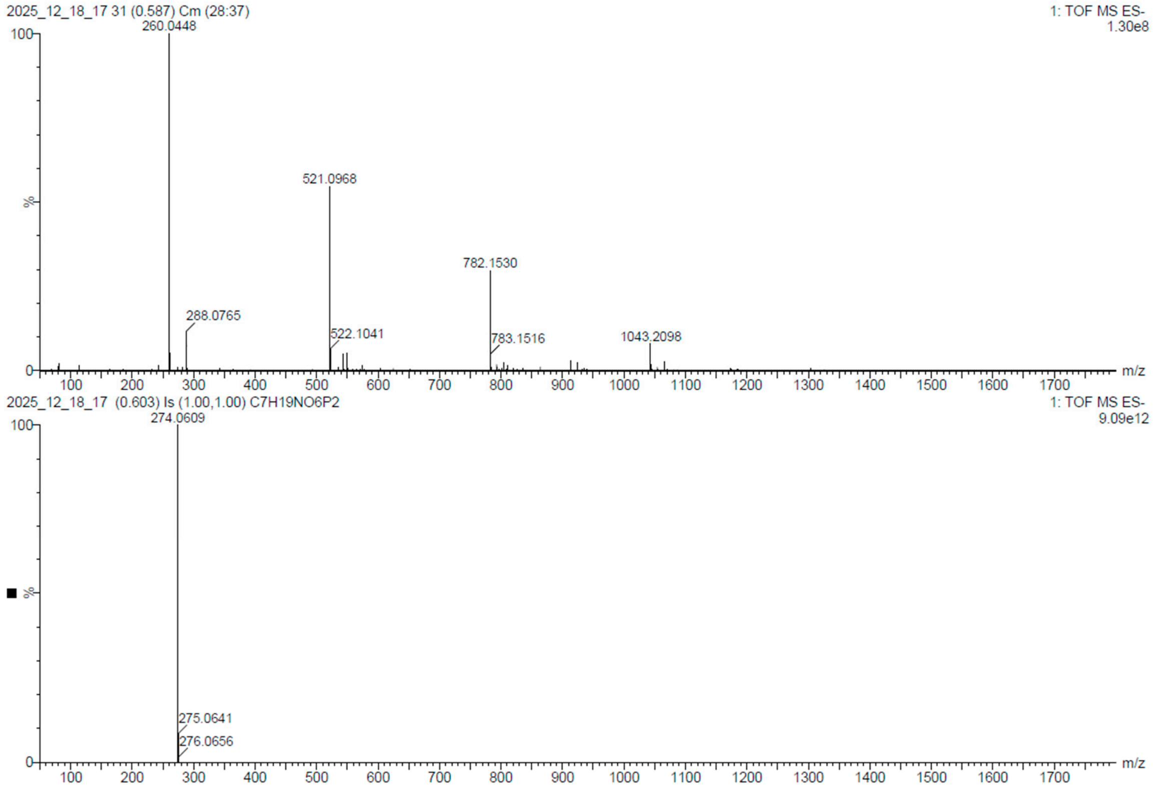

MS spectrum of compound **15**

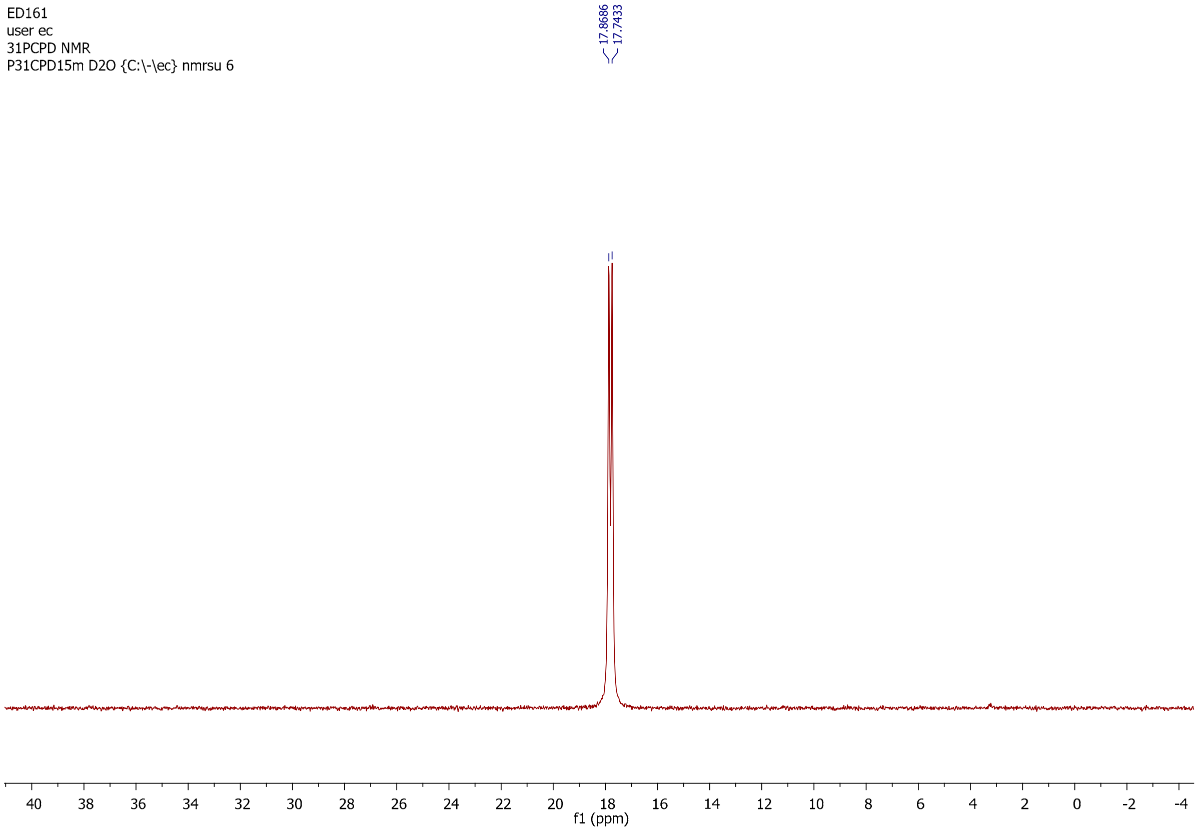

^{31}P NMR spectrum of compound **16**

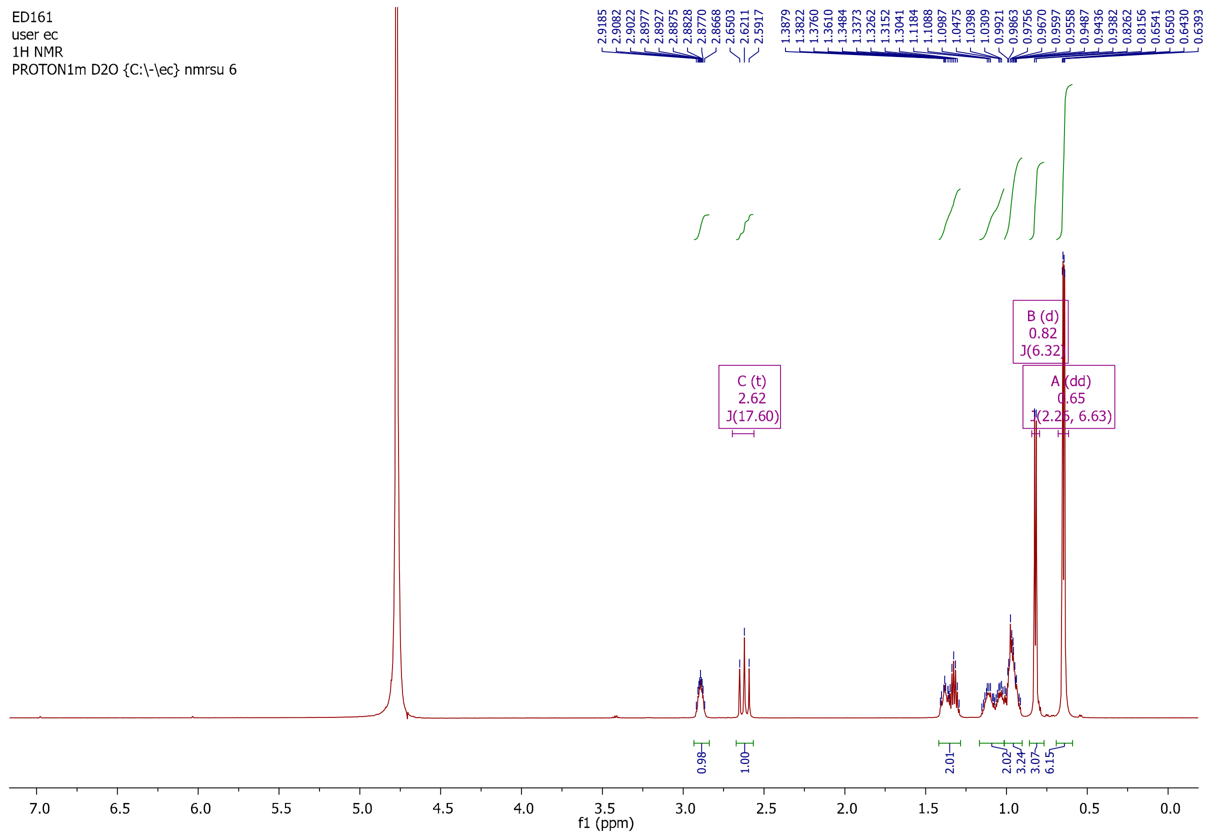

^{1}H NMR spectrum of compound **16**

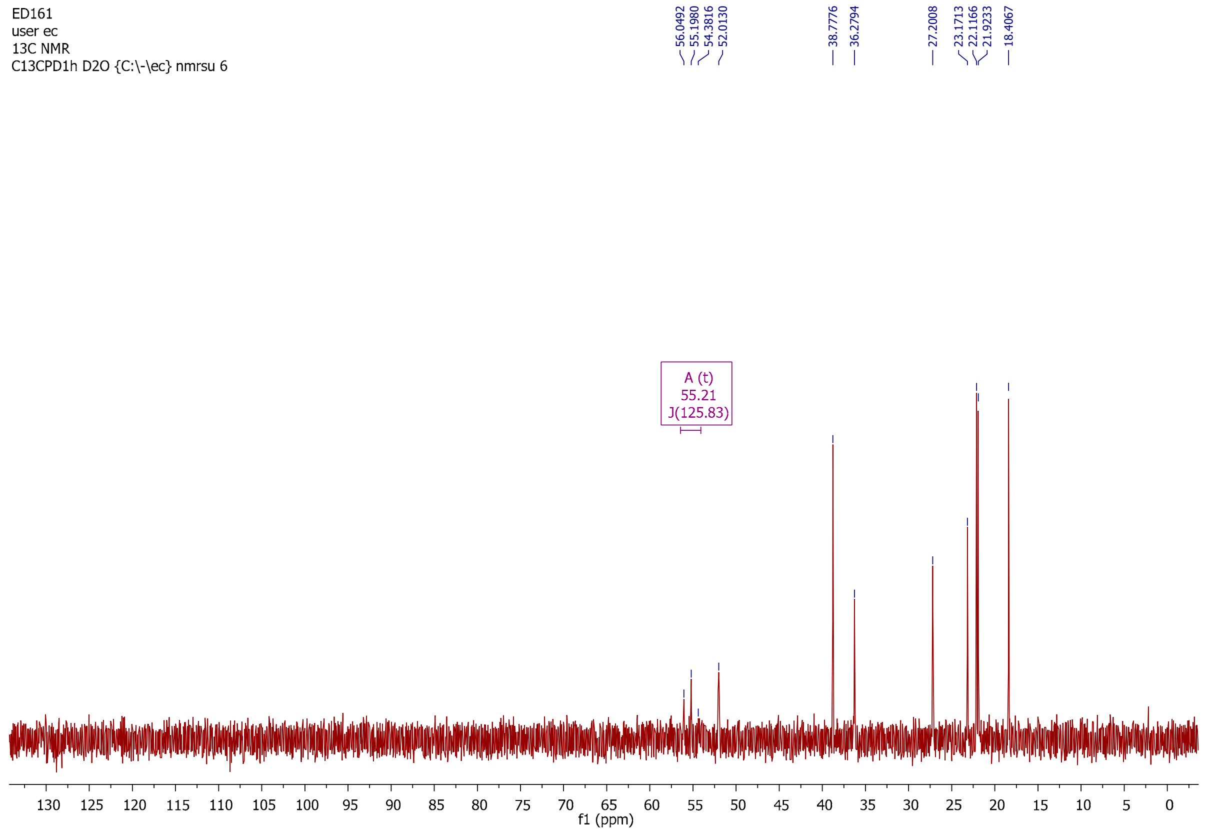

^{13}C NMR spectrum of compound **16**

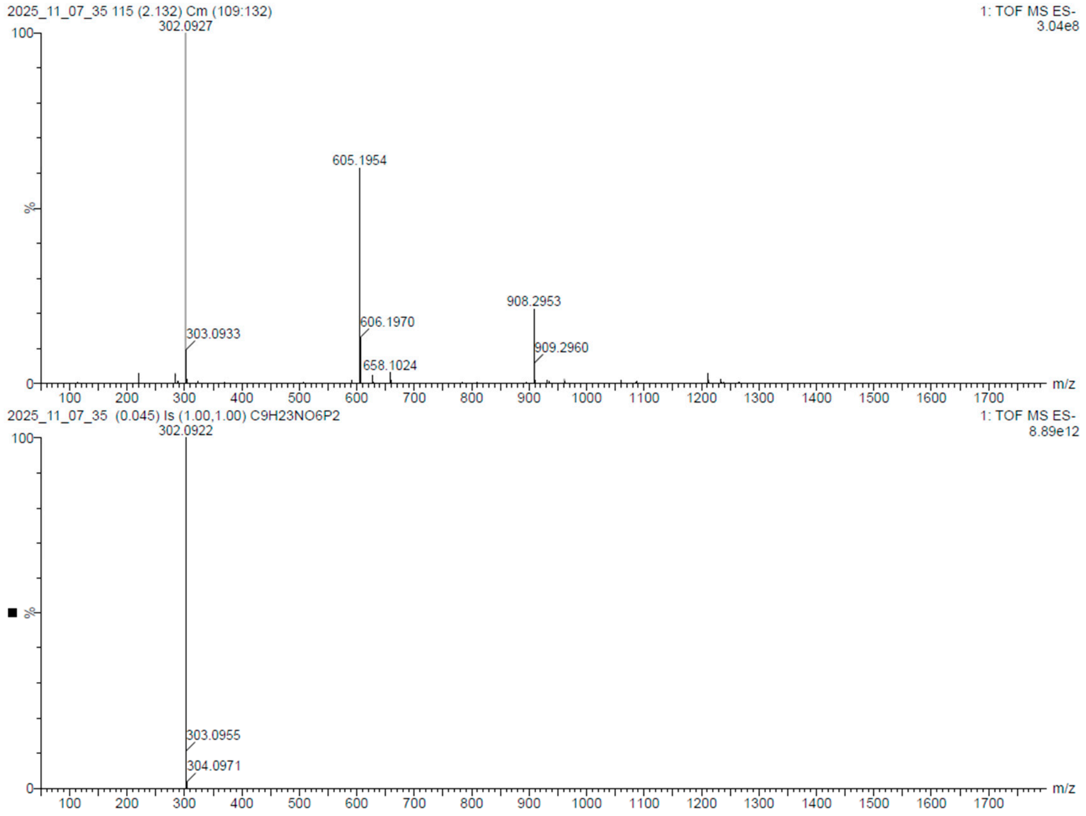

MS spectrum of compound **16**

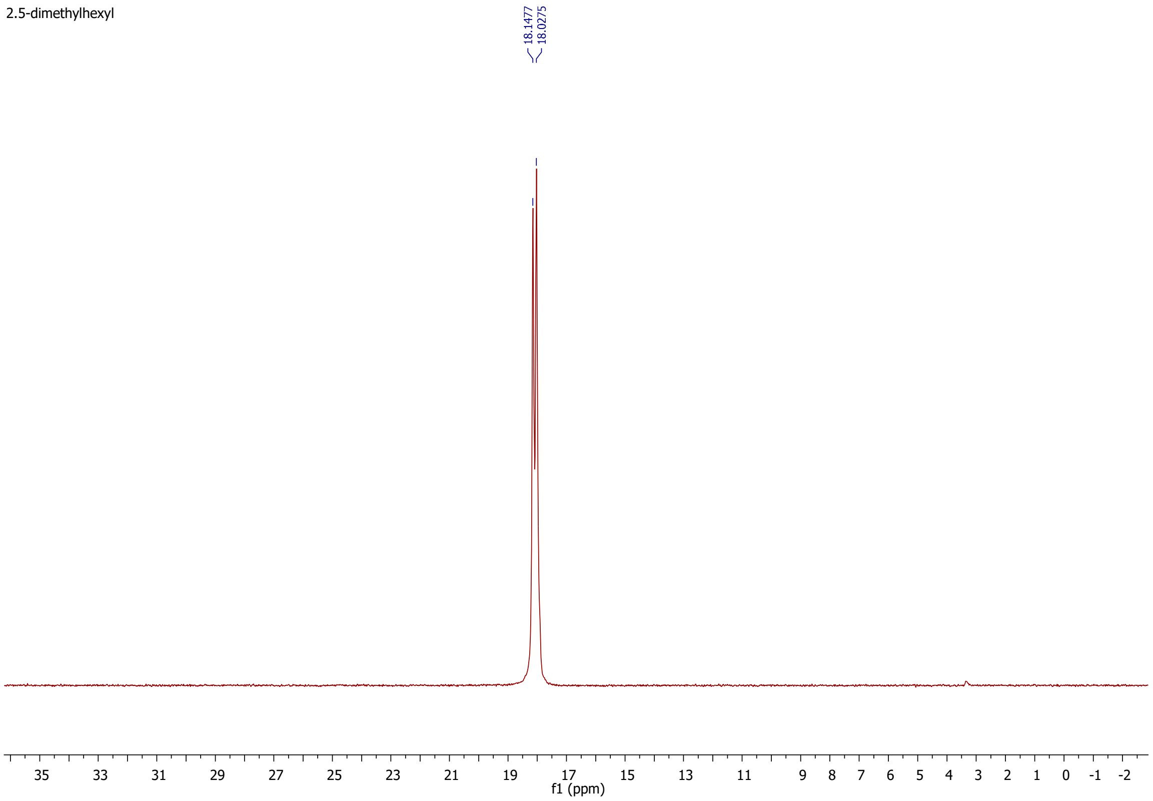

^{31}P NMR spectrum of compound **17**

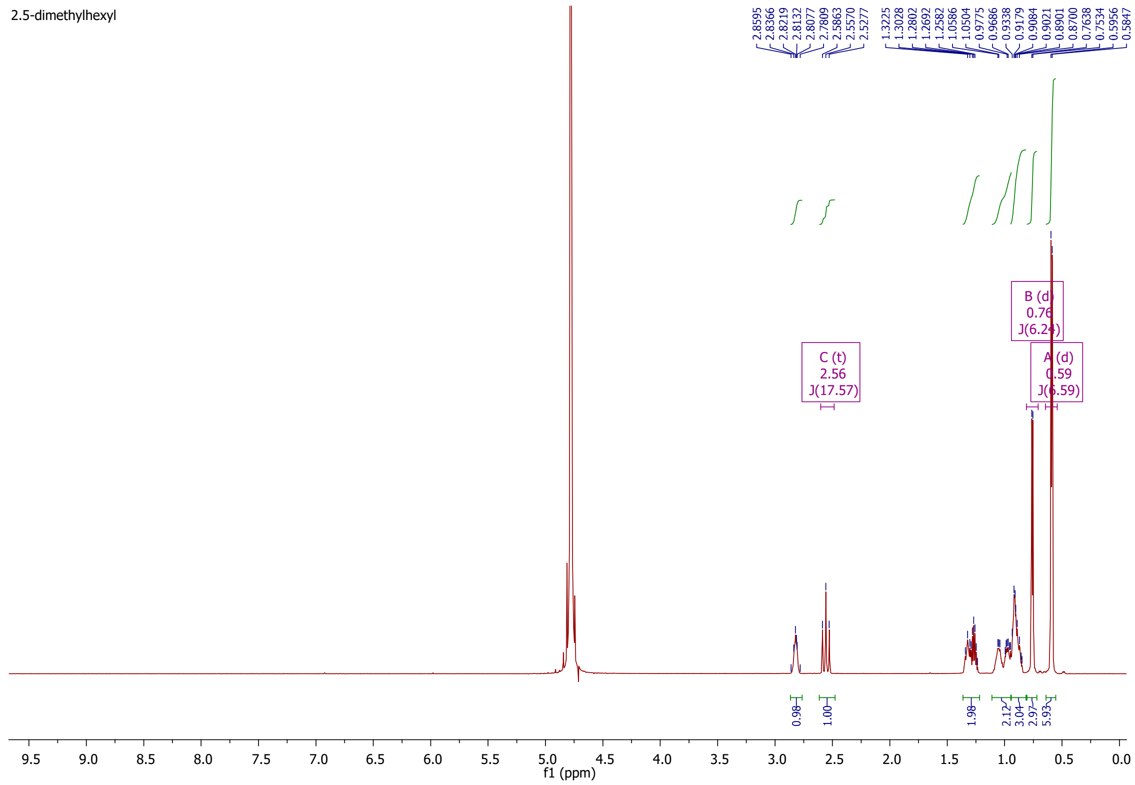

^{1}H NMR spectrum of compound **17**

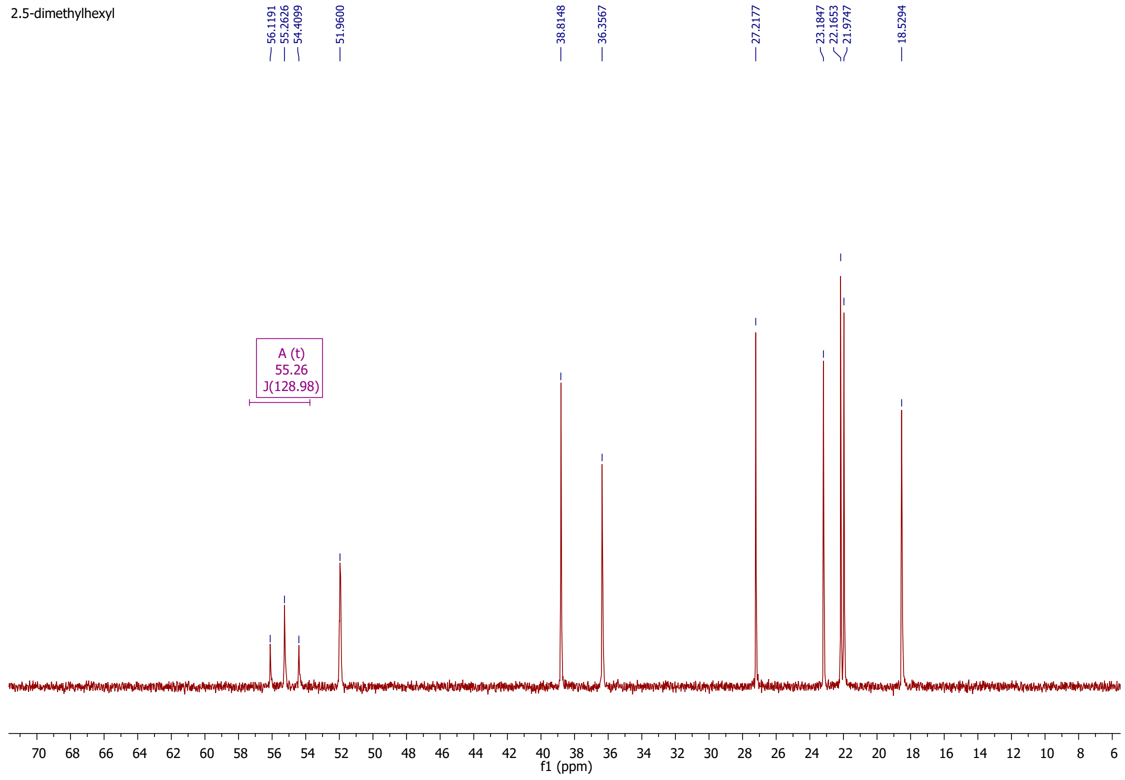

^{13}C NMR spectrum of compound **17**

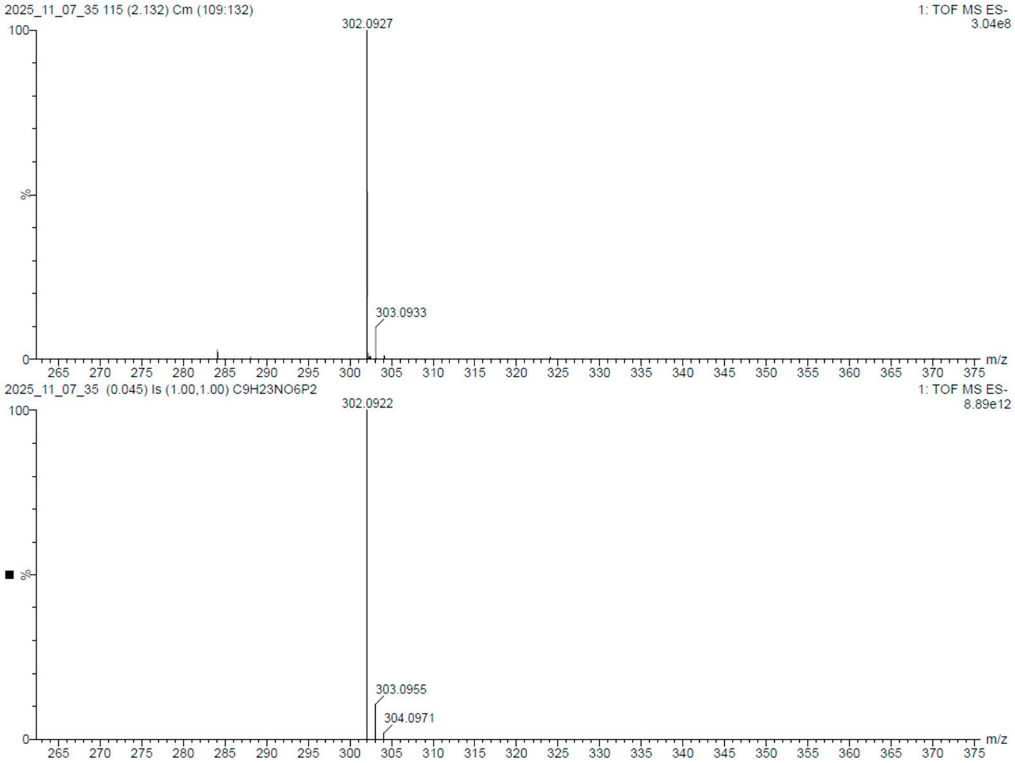

MS spectrum of compound **17**

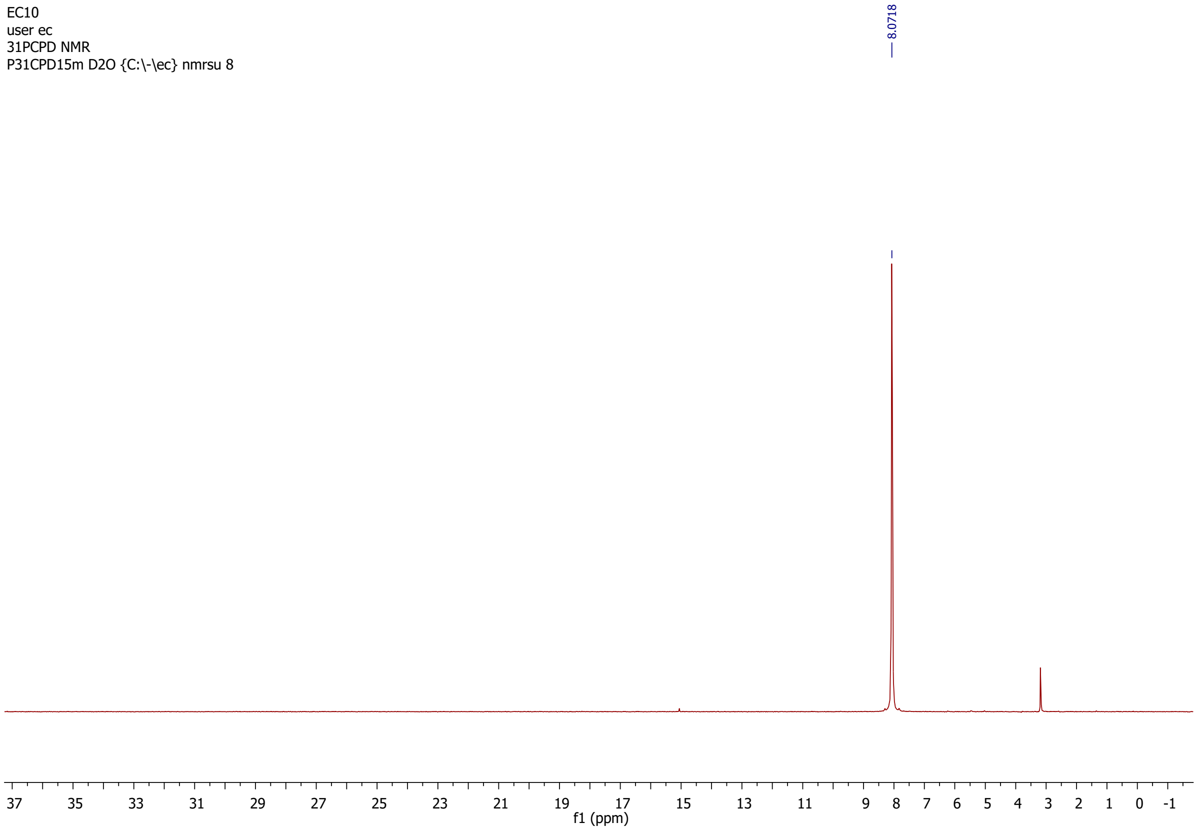

^{31}P NMR spectrum of compound **18**

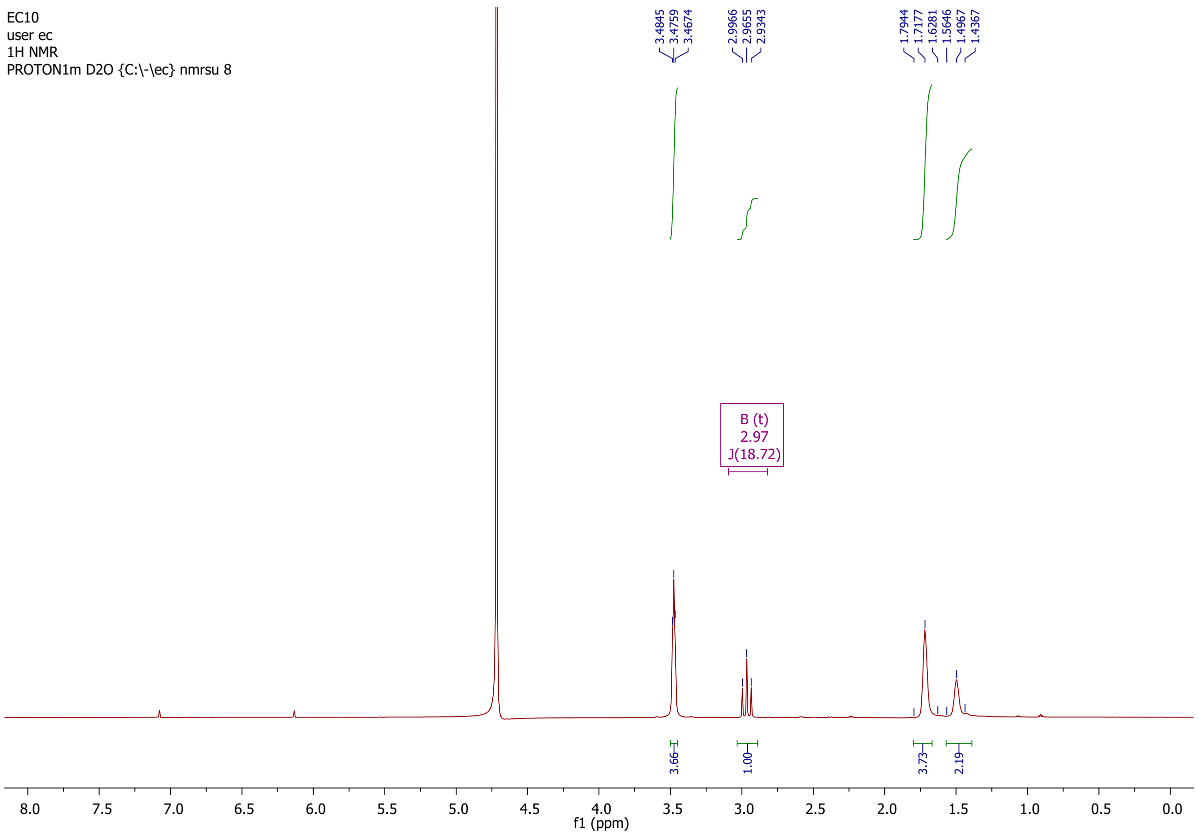

^{1}H NMR spectrum of compound **18**

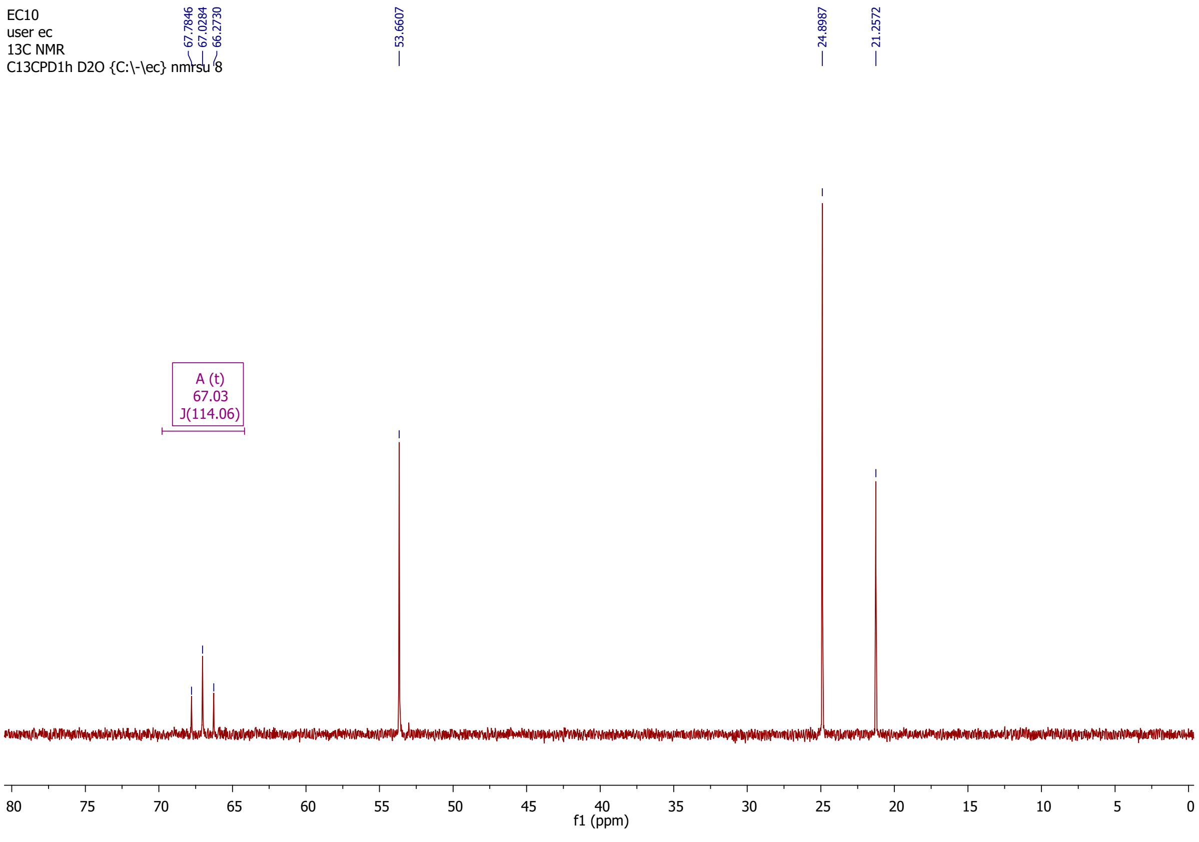

^{1c3}C NMR spectrum of compound **18**

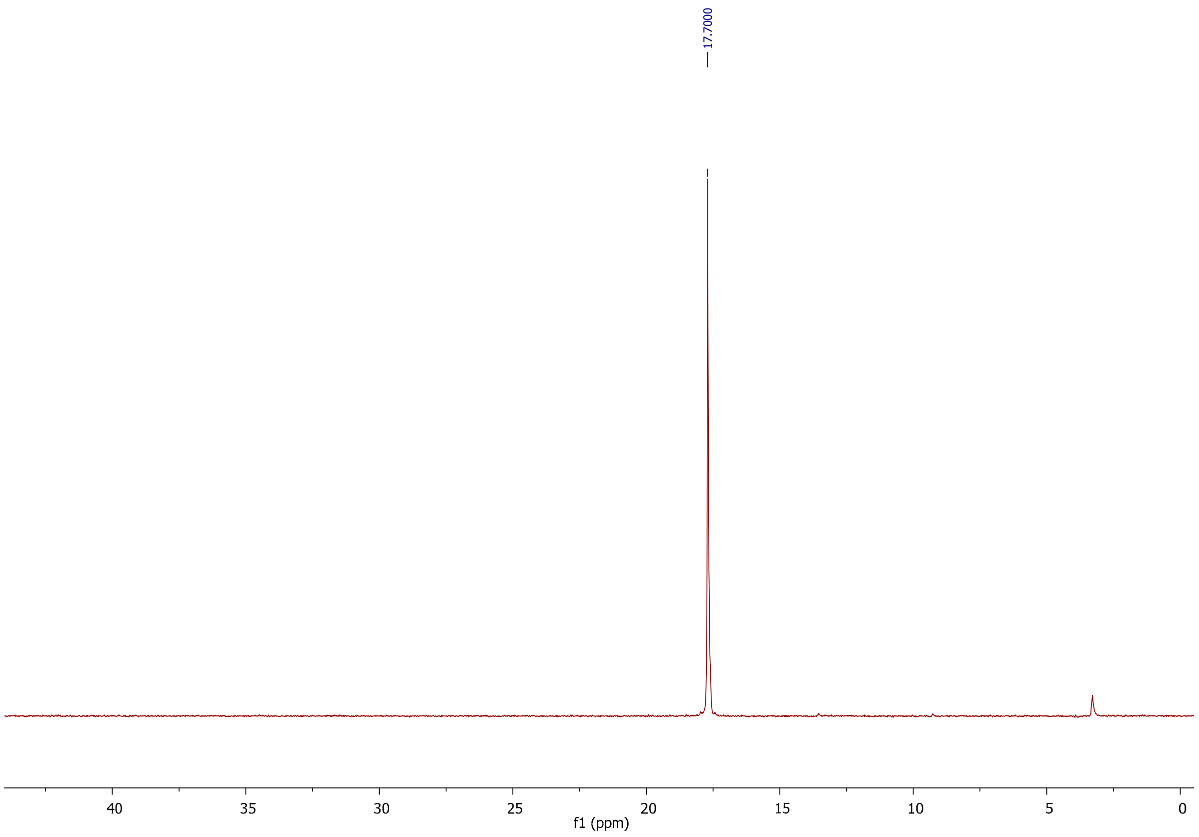

^{31}P NMR spectrum of compound **19**

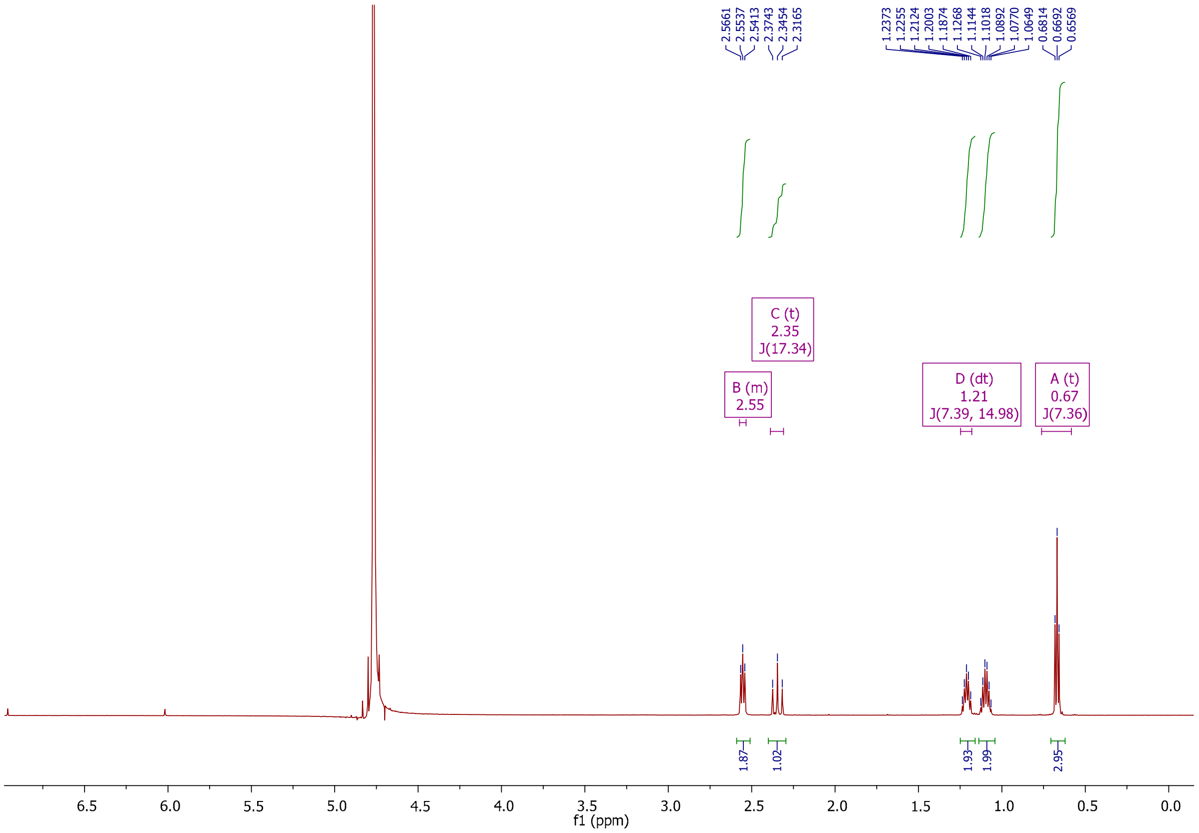

^{1}H NMR spectrum of compound **19**

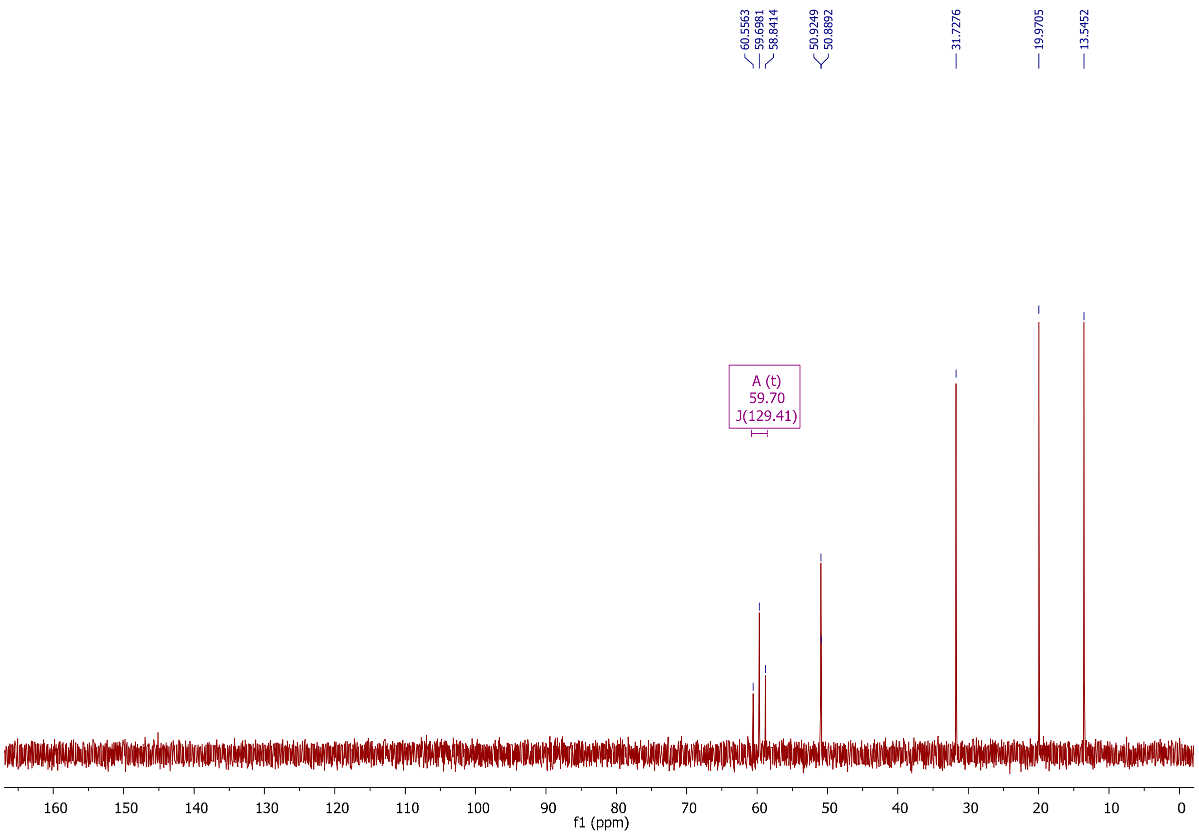

^{13}C NMR spectrum of compound **19**

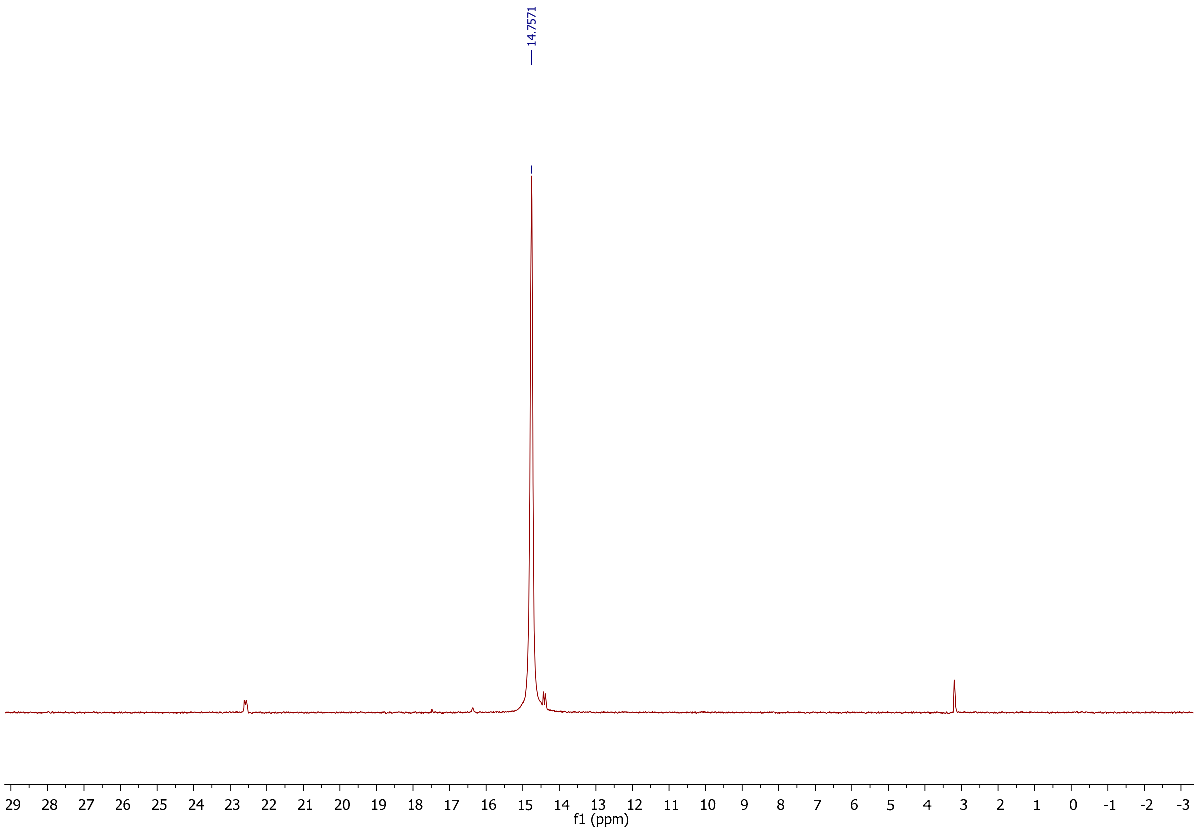

^{31}P NMR spectrum of compound **20**

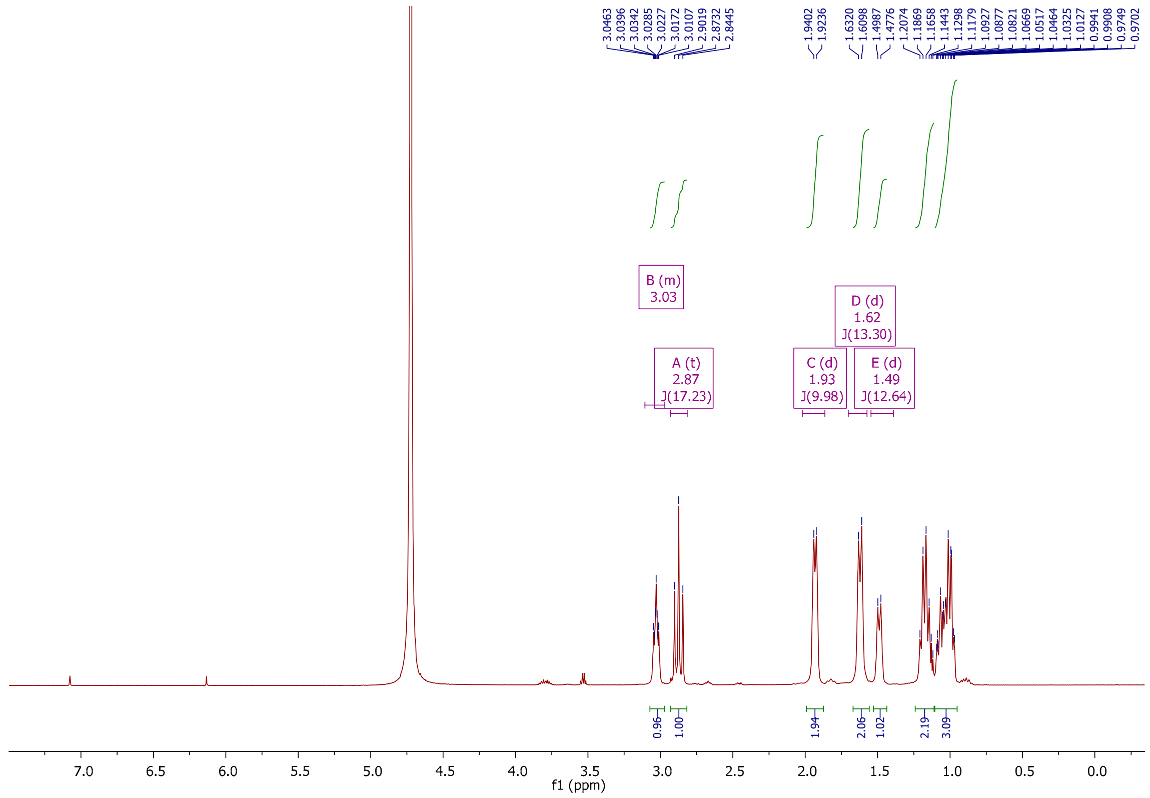

^{1}H NMR spectrum of compound **20**

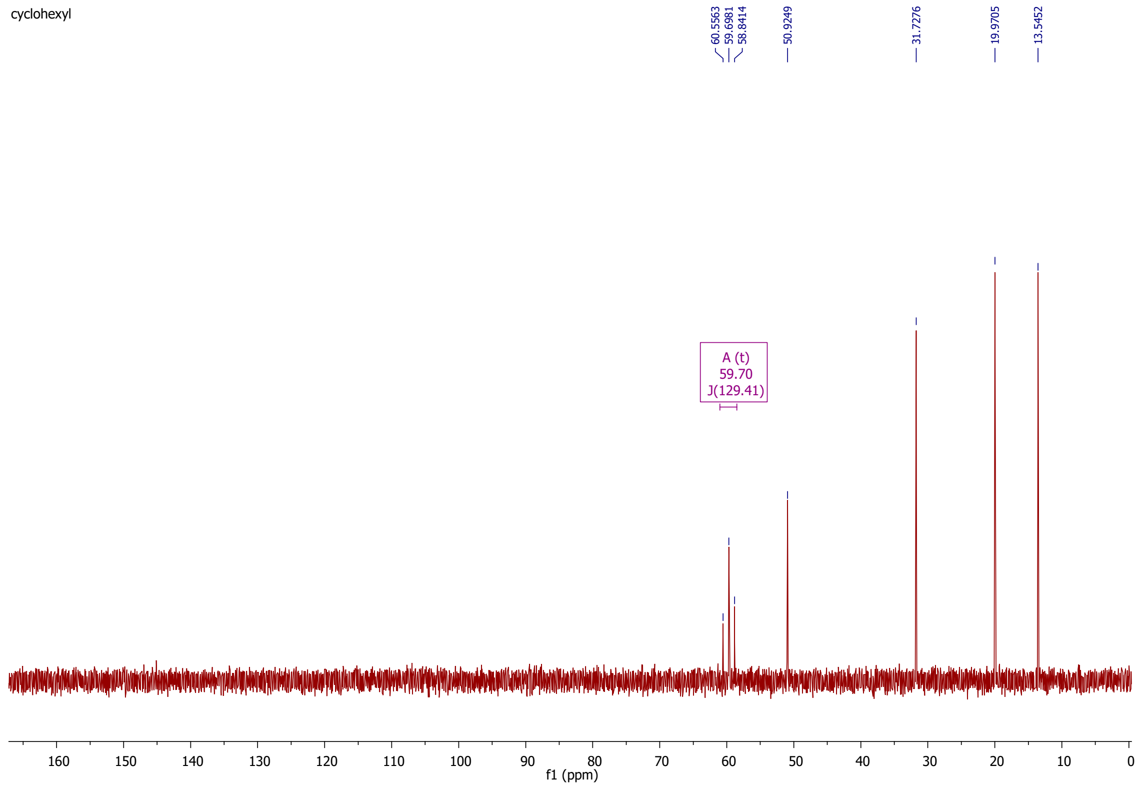

^{13}C NMR spectrum of compound **20**

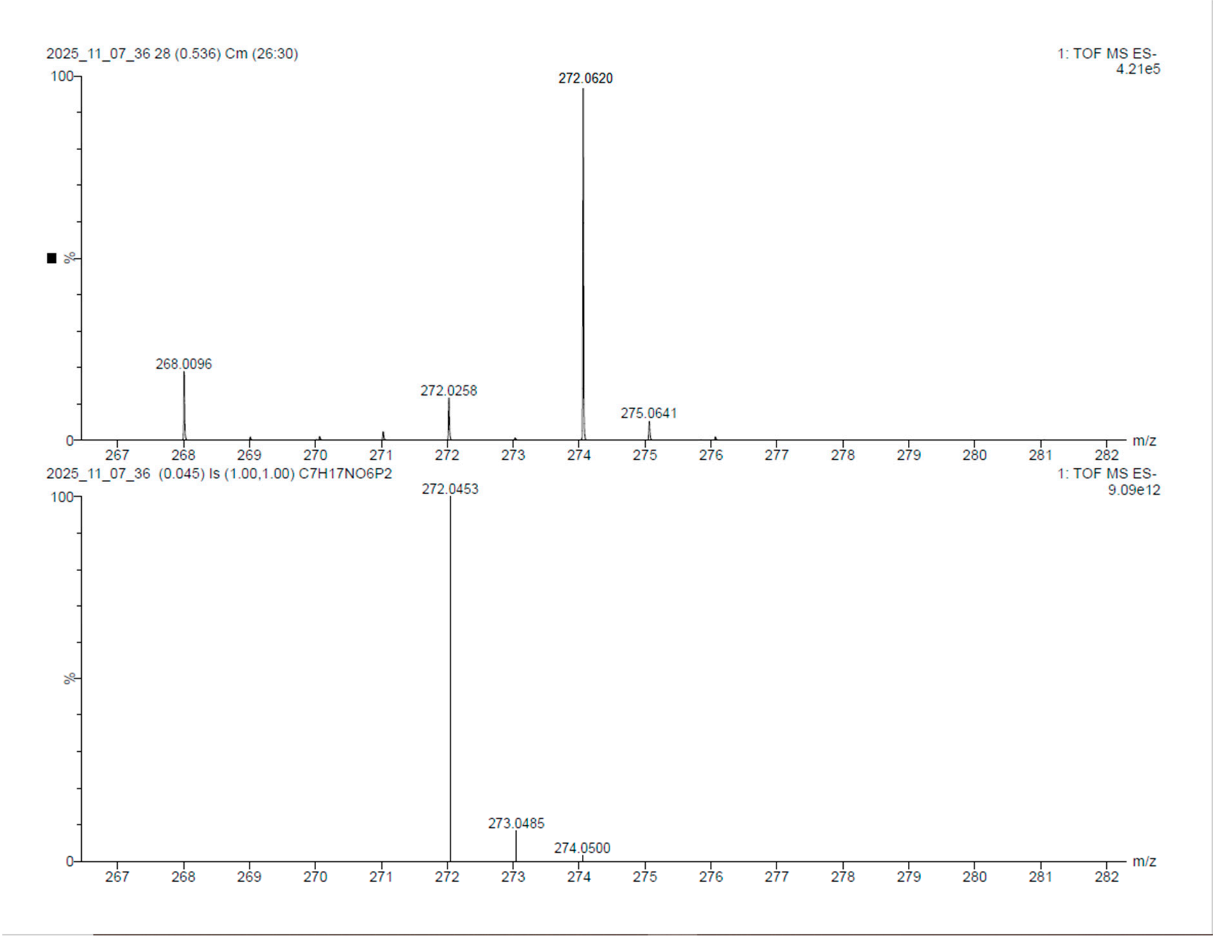

MS spectrum of compound **20**

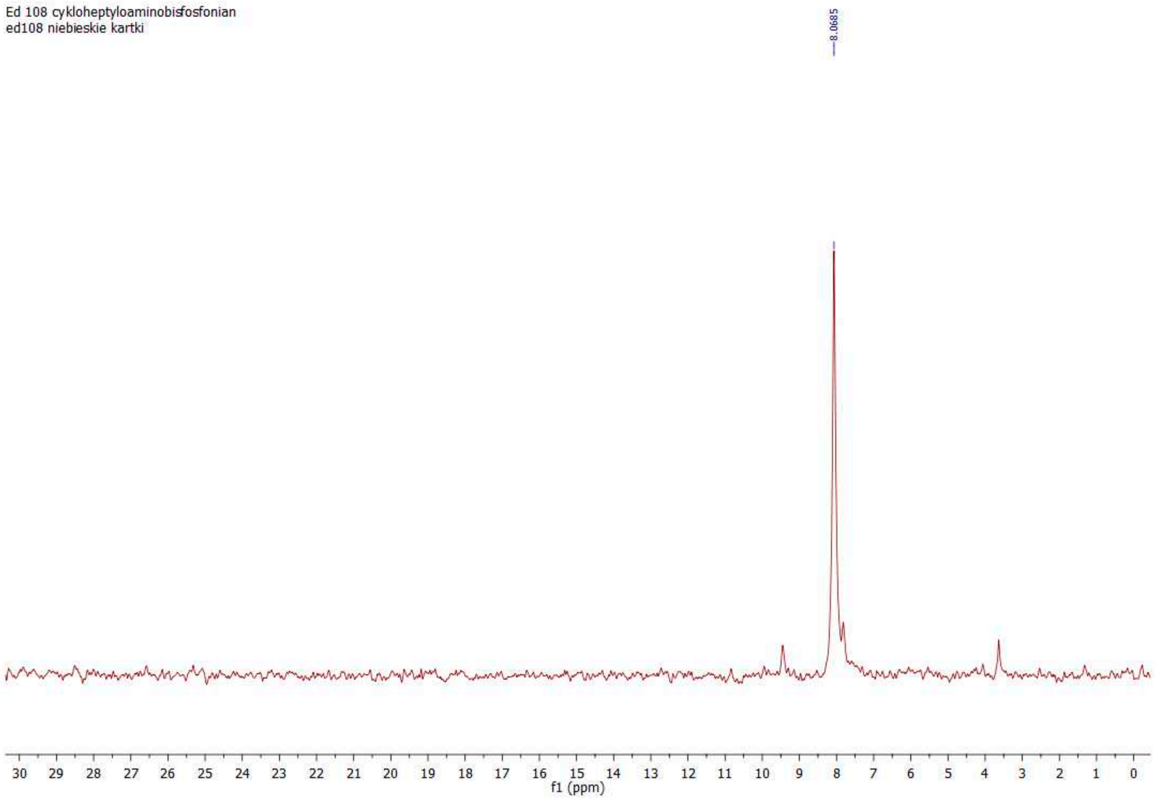

^{31}P NMR spectrum of compound **21**

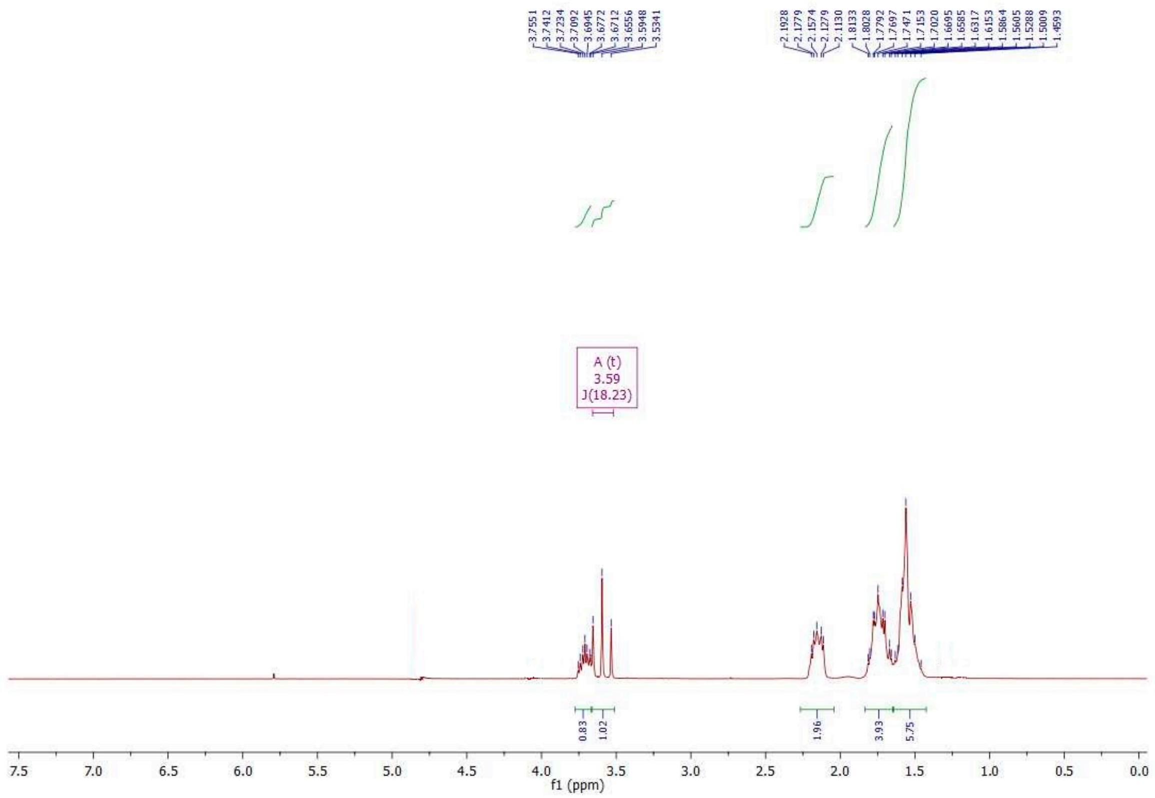

^{1}H NMR spectrum of compound **21**

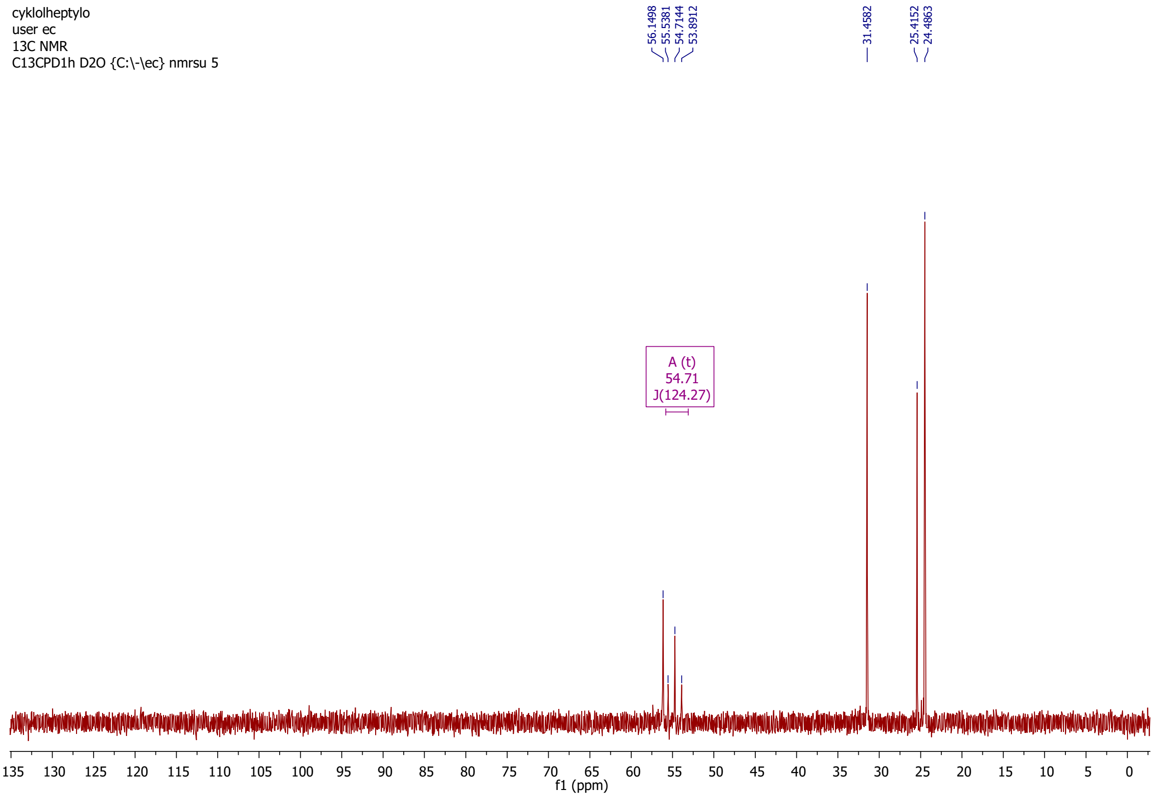

^{13}C NMR spectrum of compound **21**

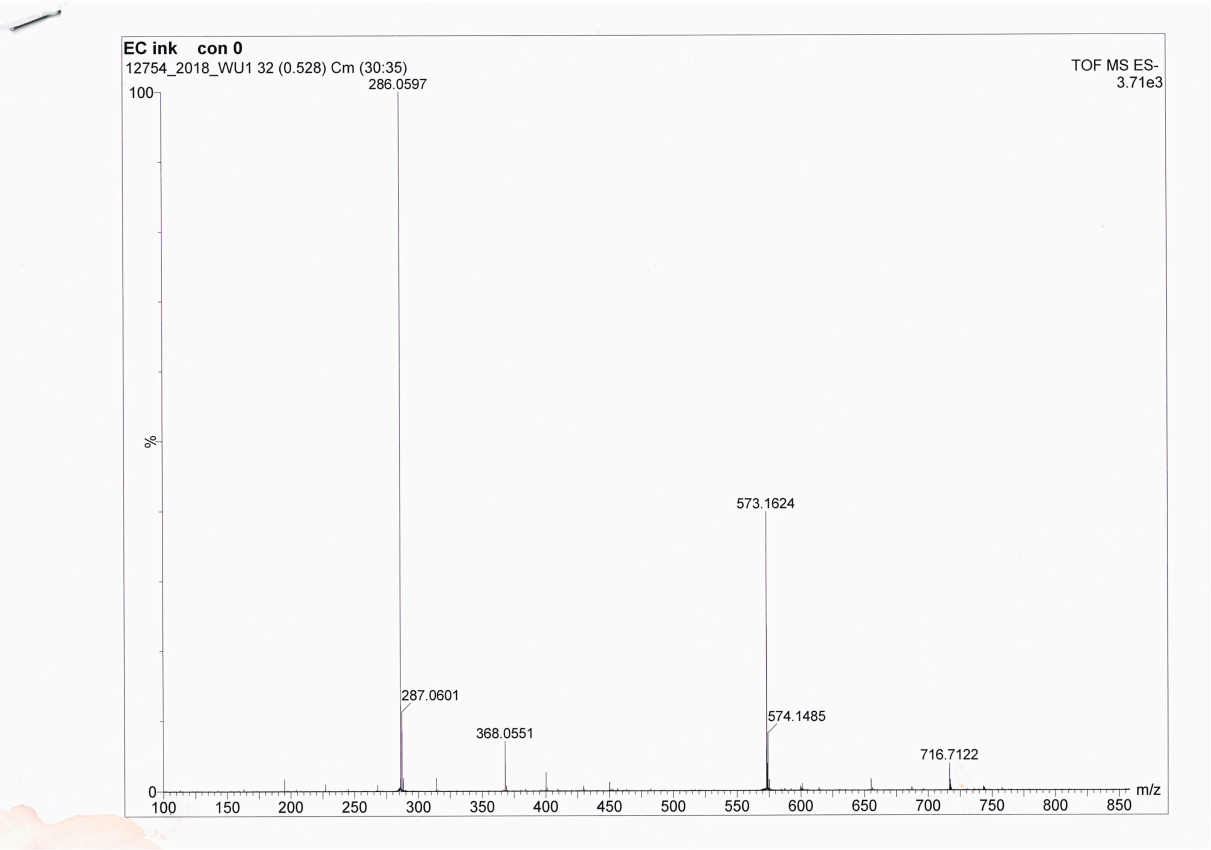

MS spectrum of compound **21**

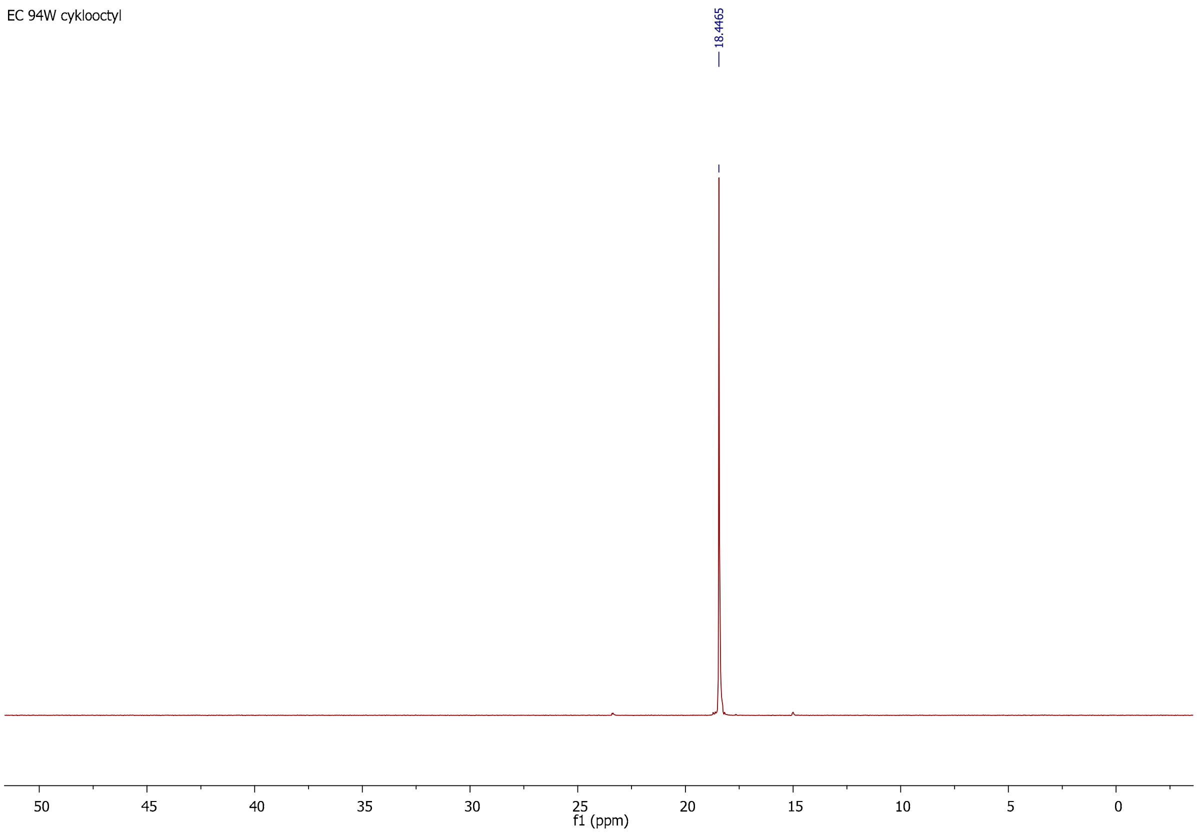

^{31}P NMR spectrum of compound **22**

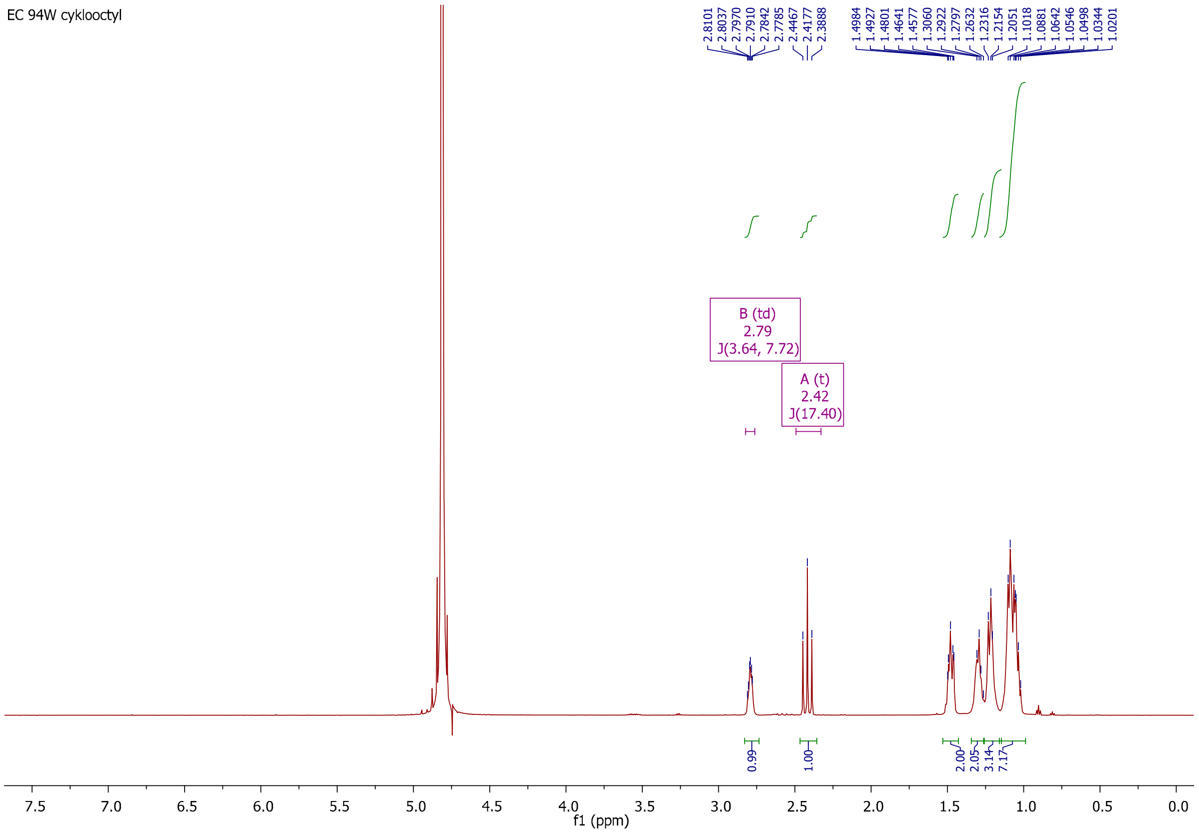

^{1}H NMR spectrum of compound **22**

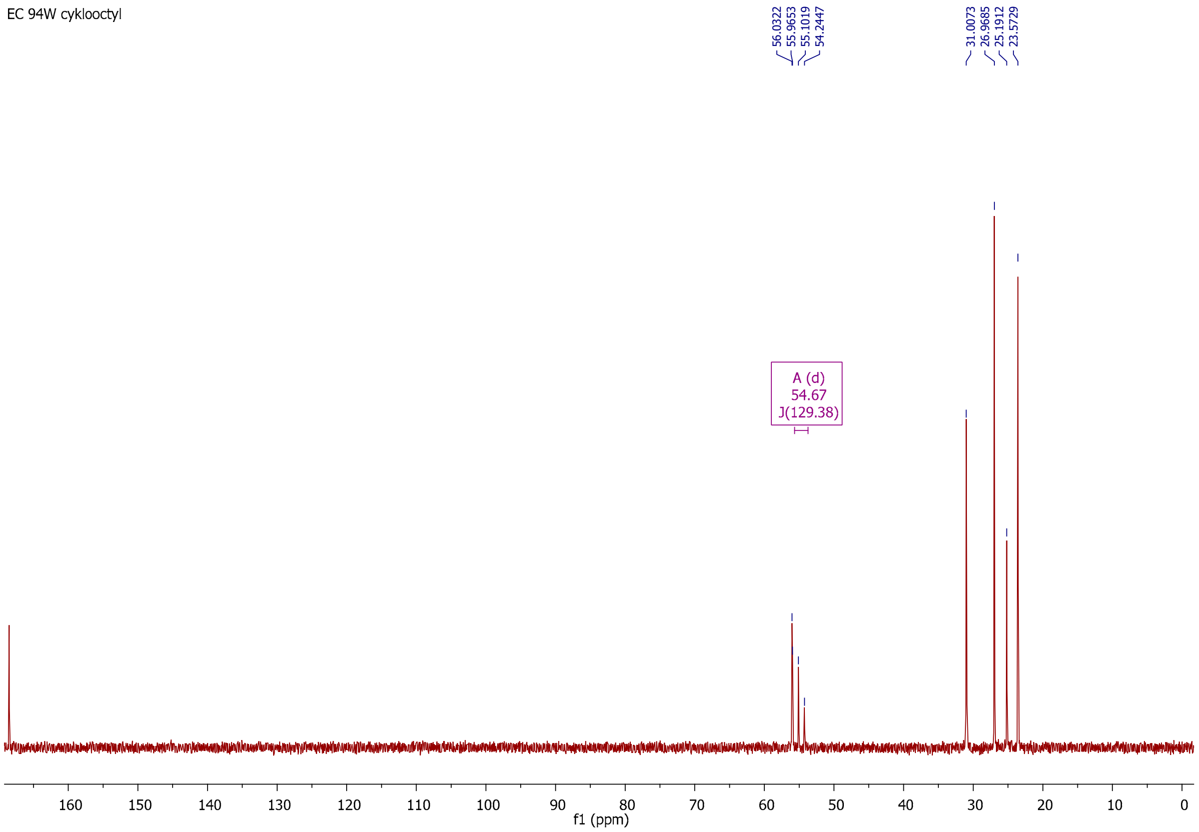

^{13}C NMR spectrum of compound **22**

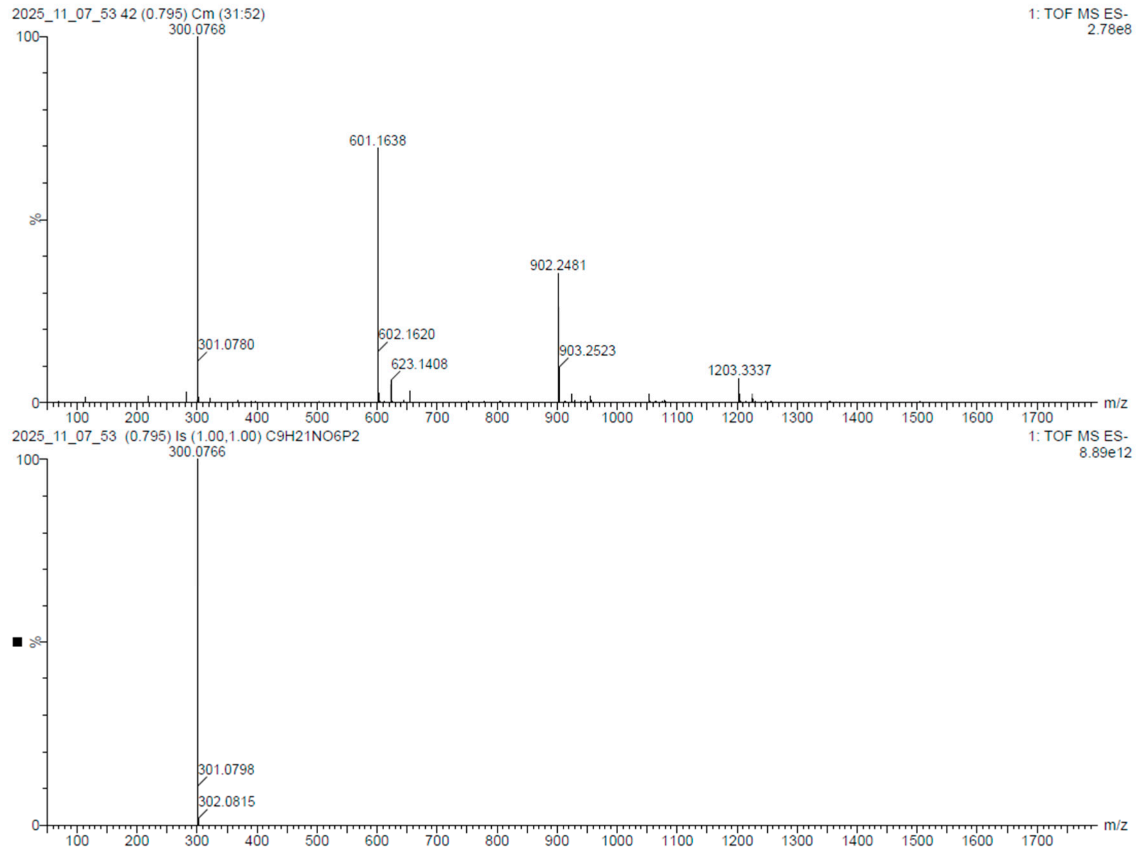

MS spectrum of compound **22**

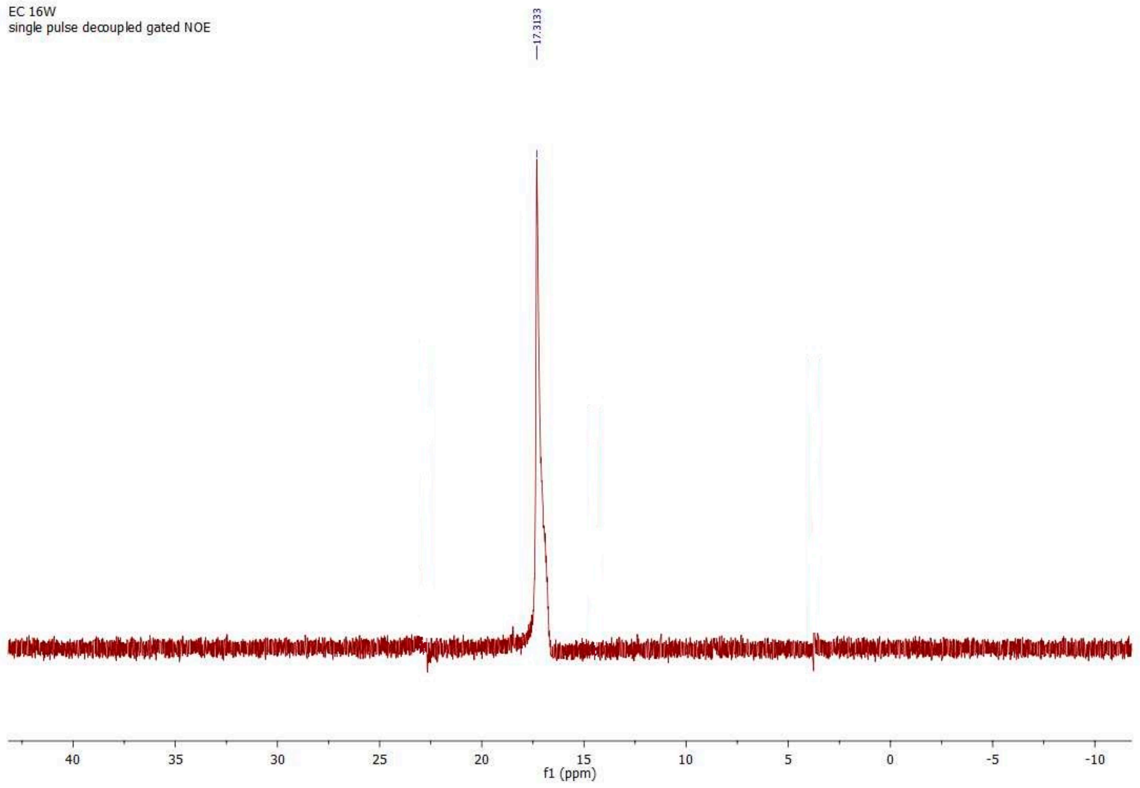

^{31}P NMR spectrum of compound **23**

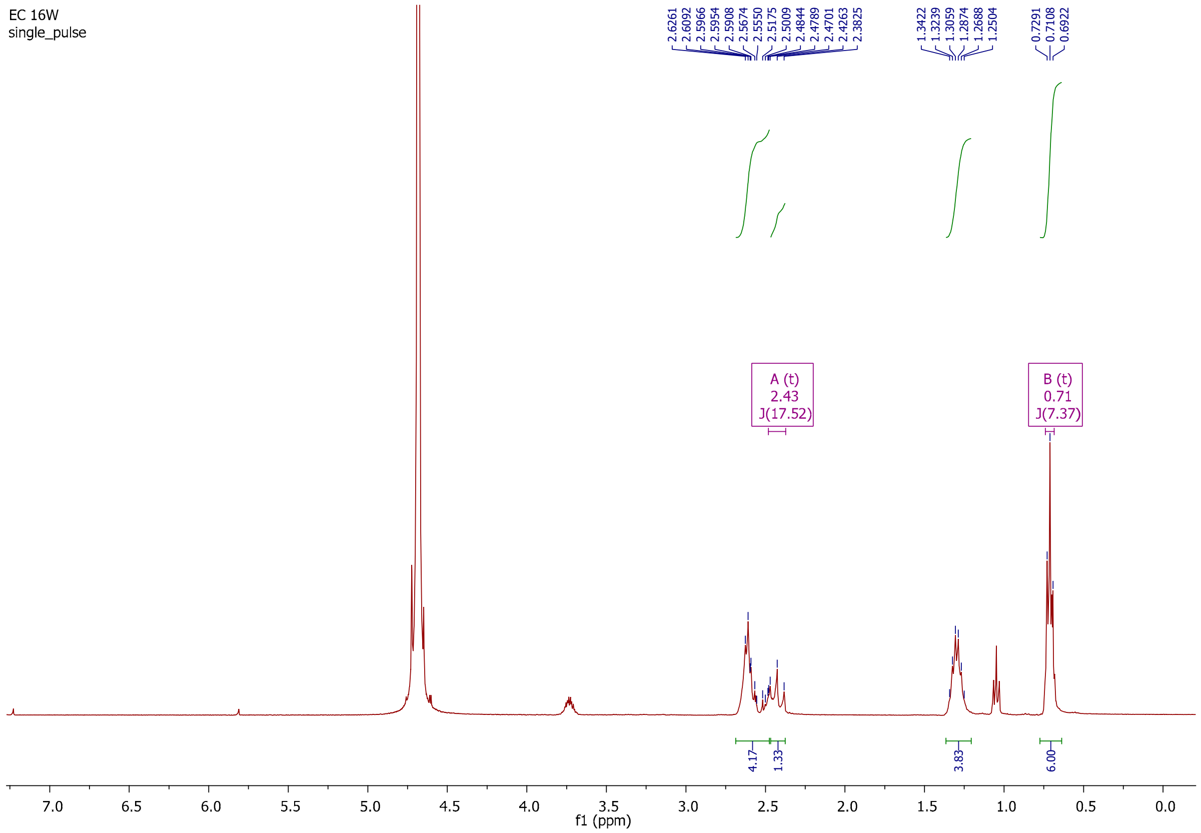

^{1}H NMR spectrum of compound **23**

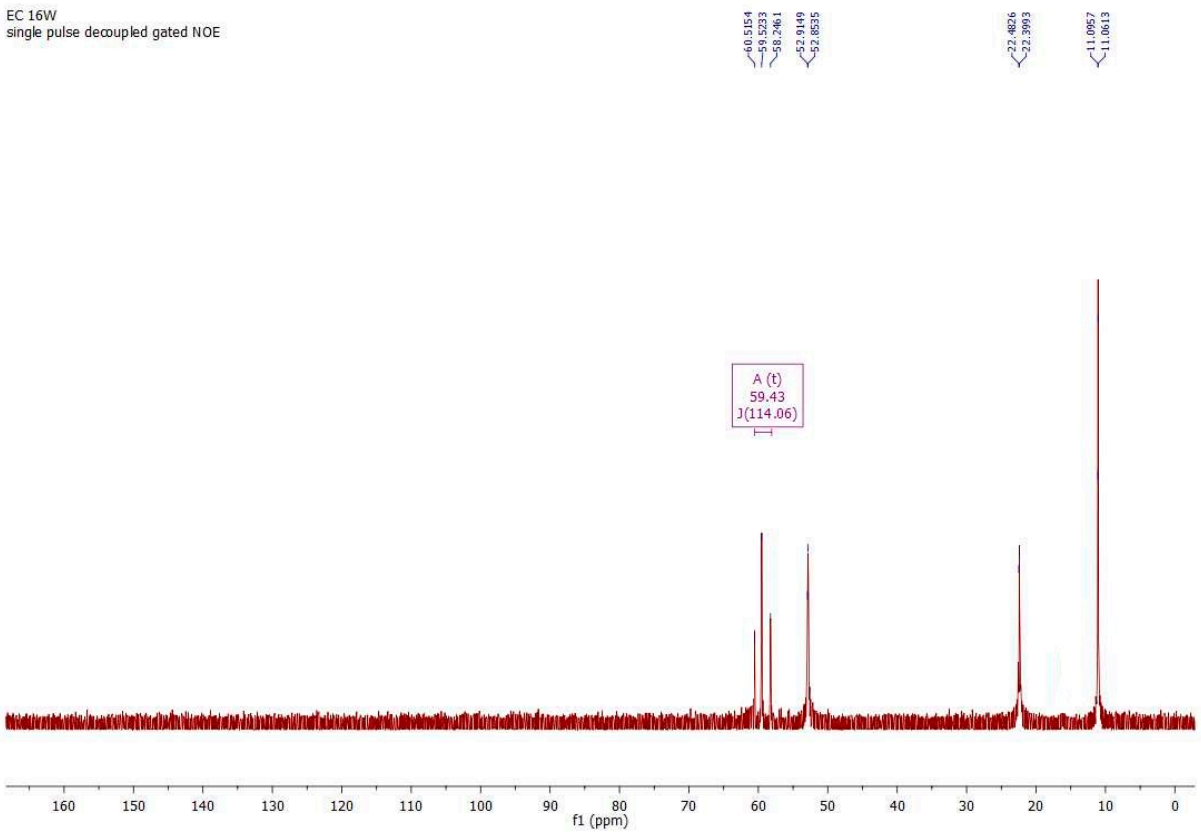

^{31}P NMR spectrum of compound **23**

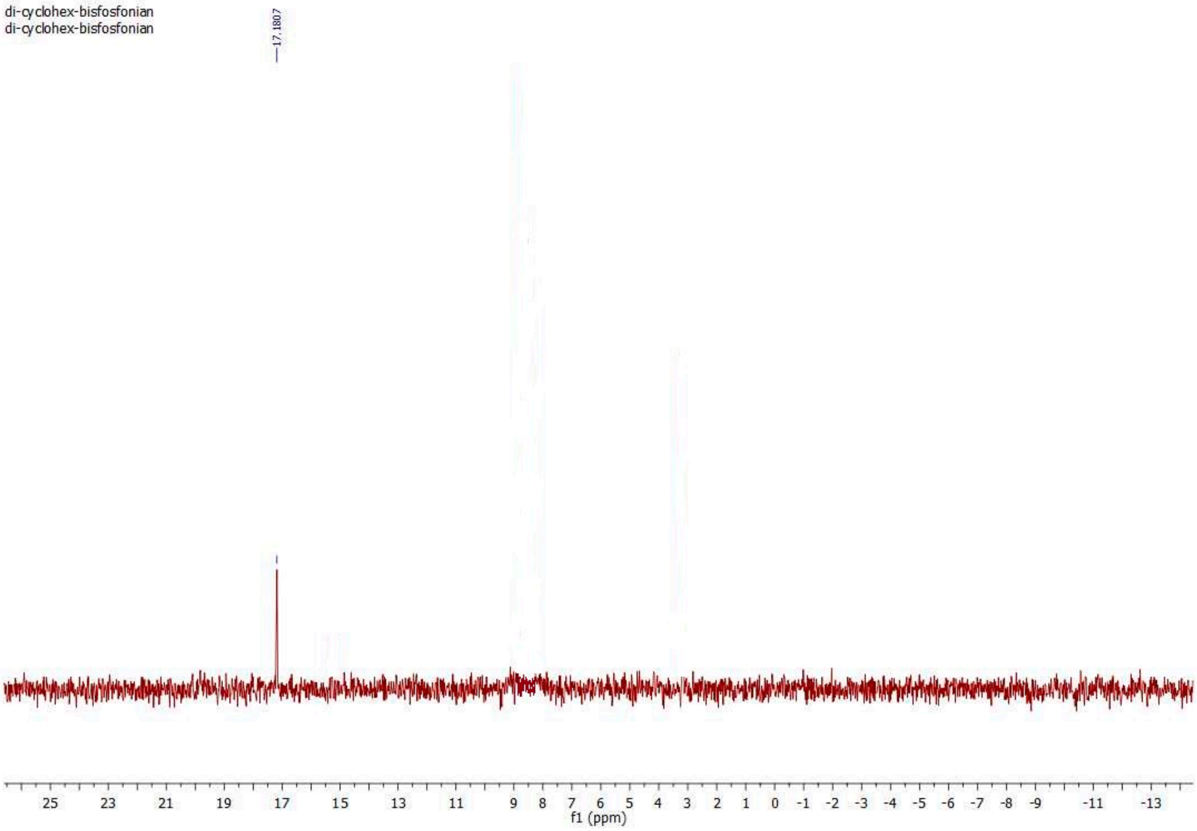

^{31}P NMR spectrum of compound **24**

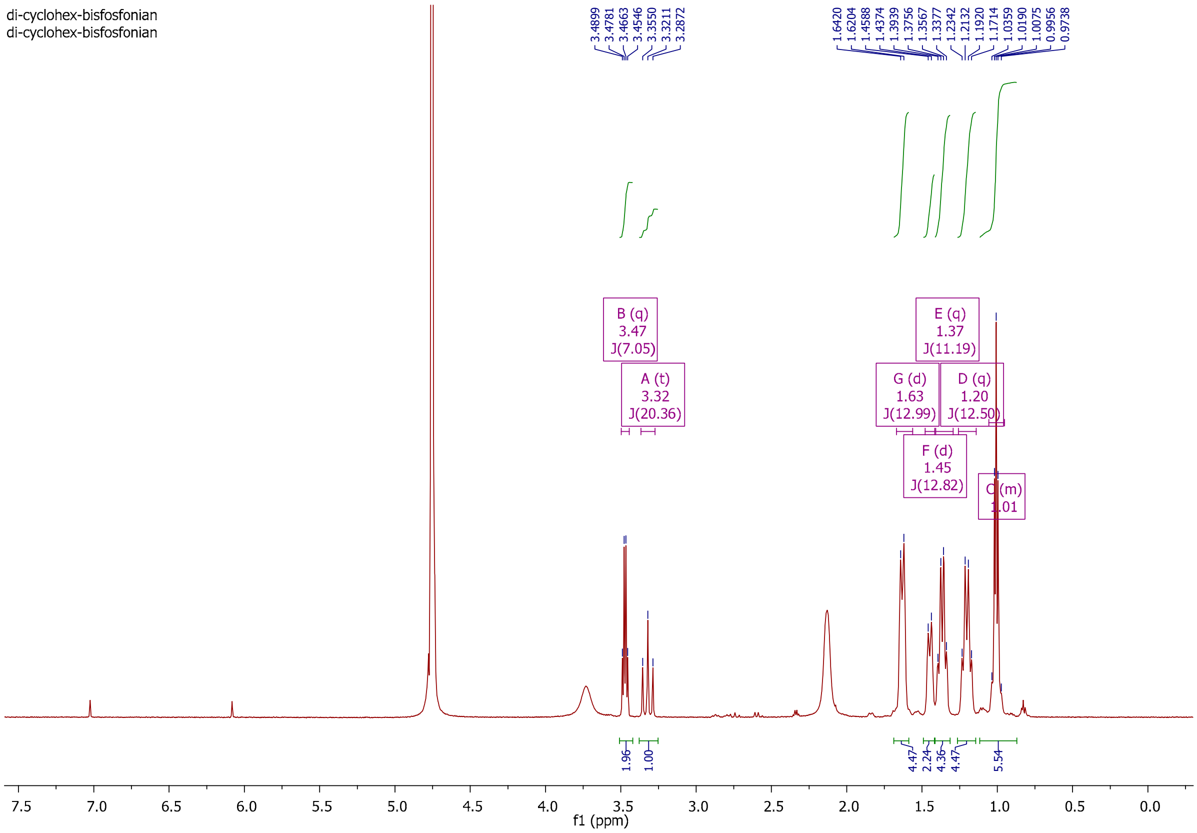

^{1}H NMR spectrum of compound **24**

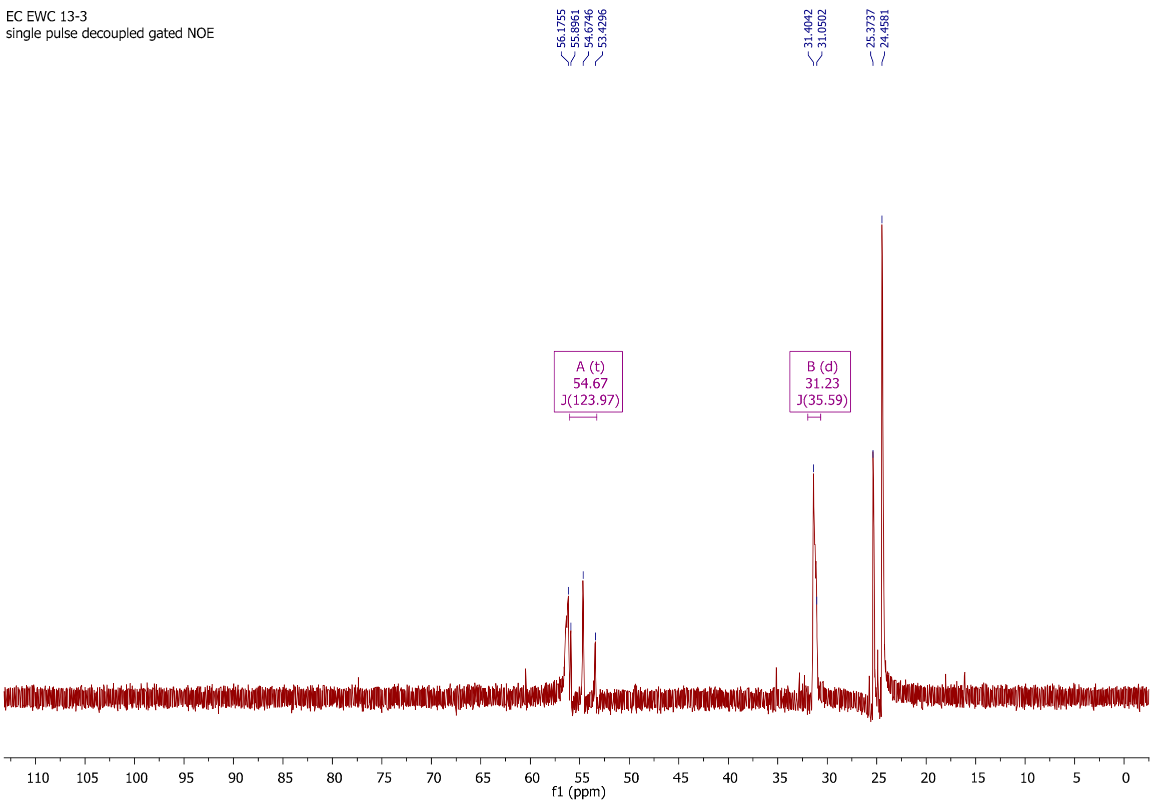

[31]C NMR spectrum of compound **24**

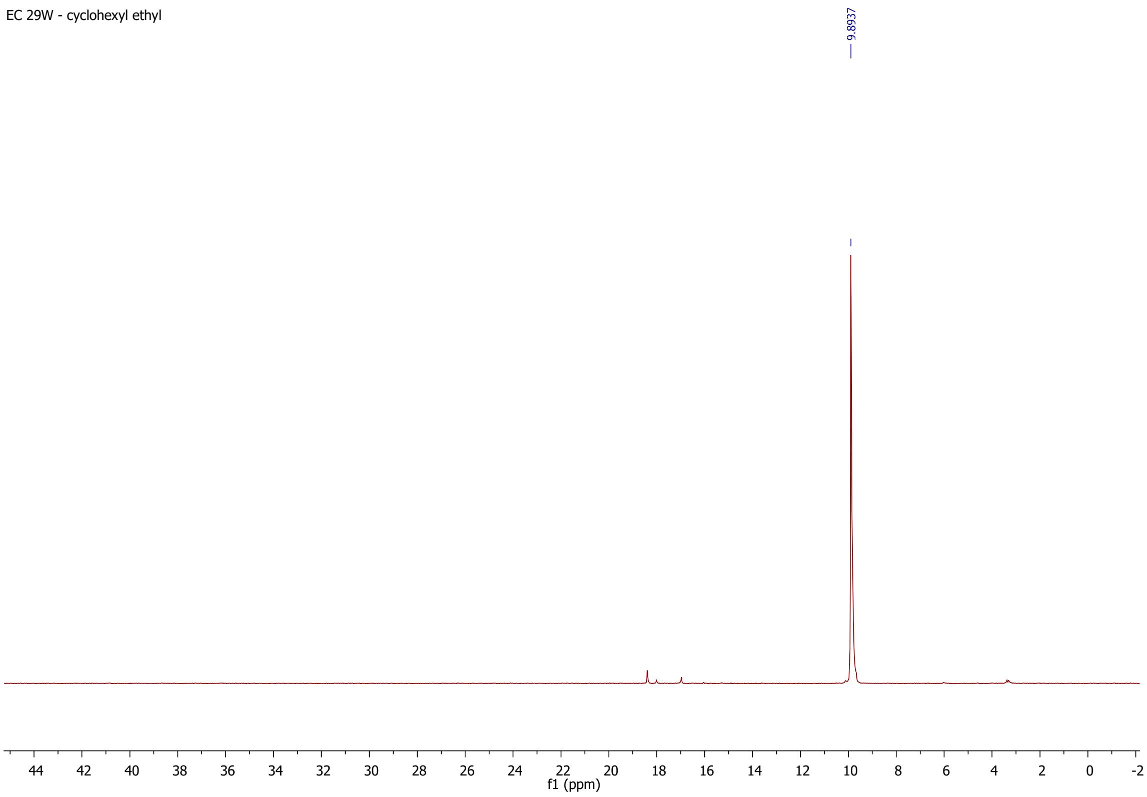

^{31}P NMR spectrum of compound **25**

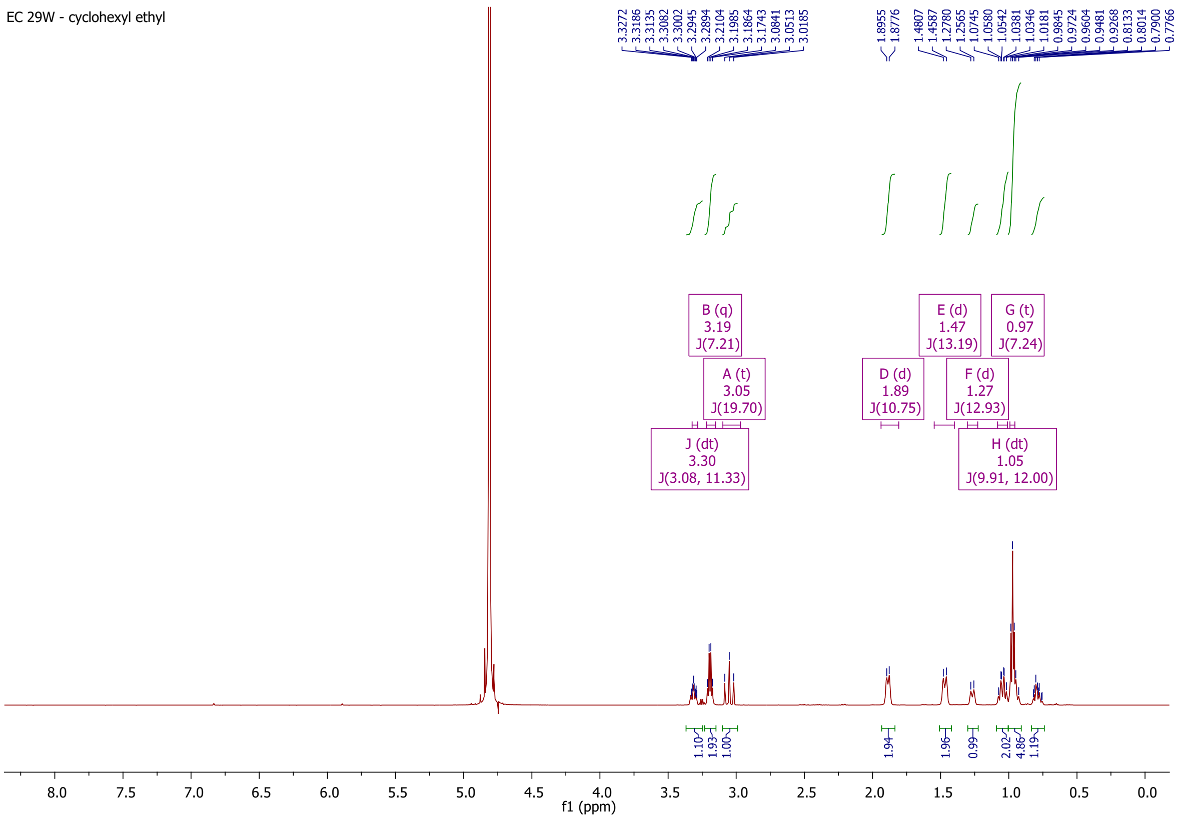

^{1}H NMR spectrum of compound **25**

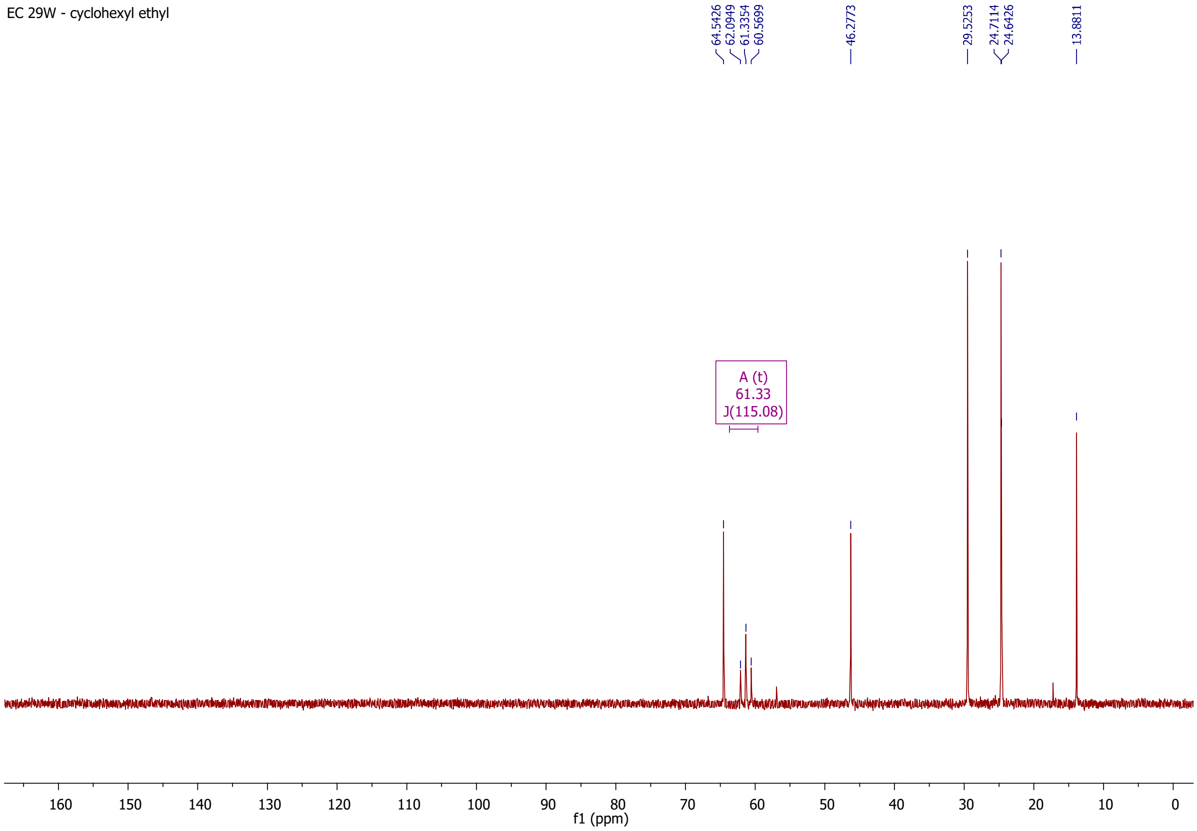

^{13}C NMR spectrum of compound **25**

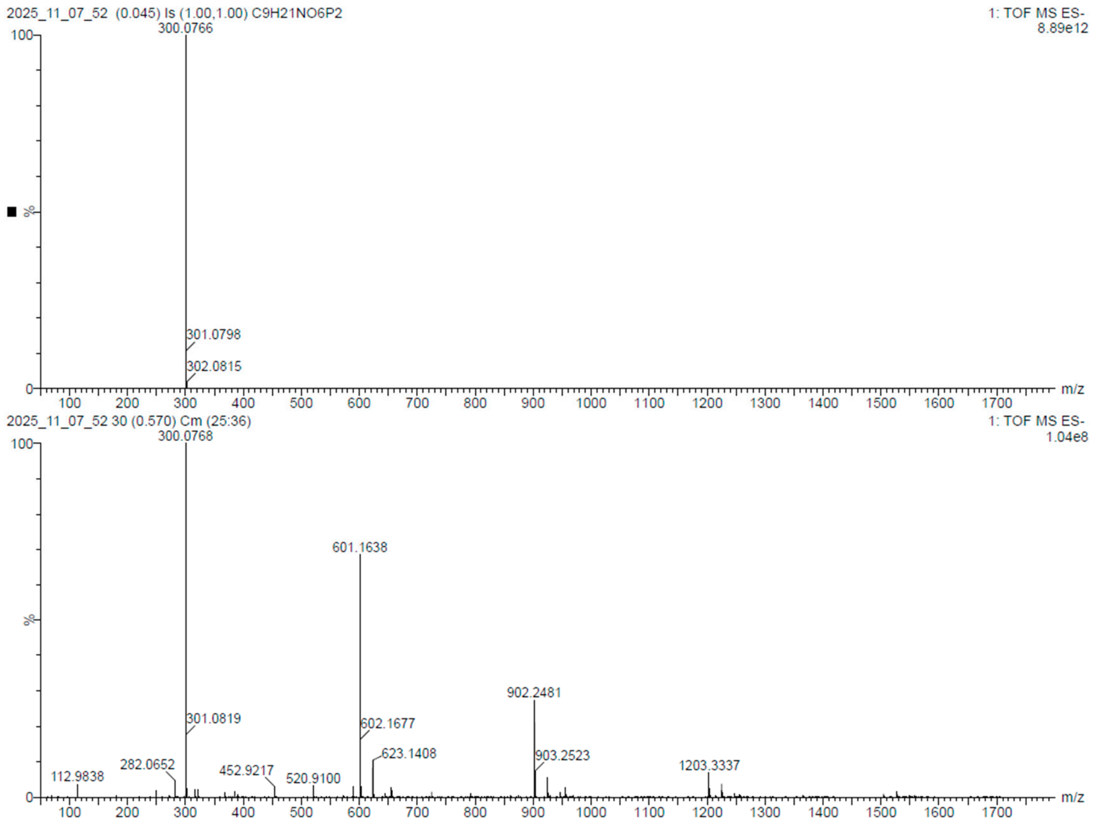

MS spectrum of compound **25**

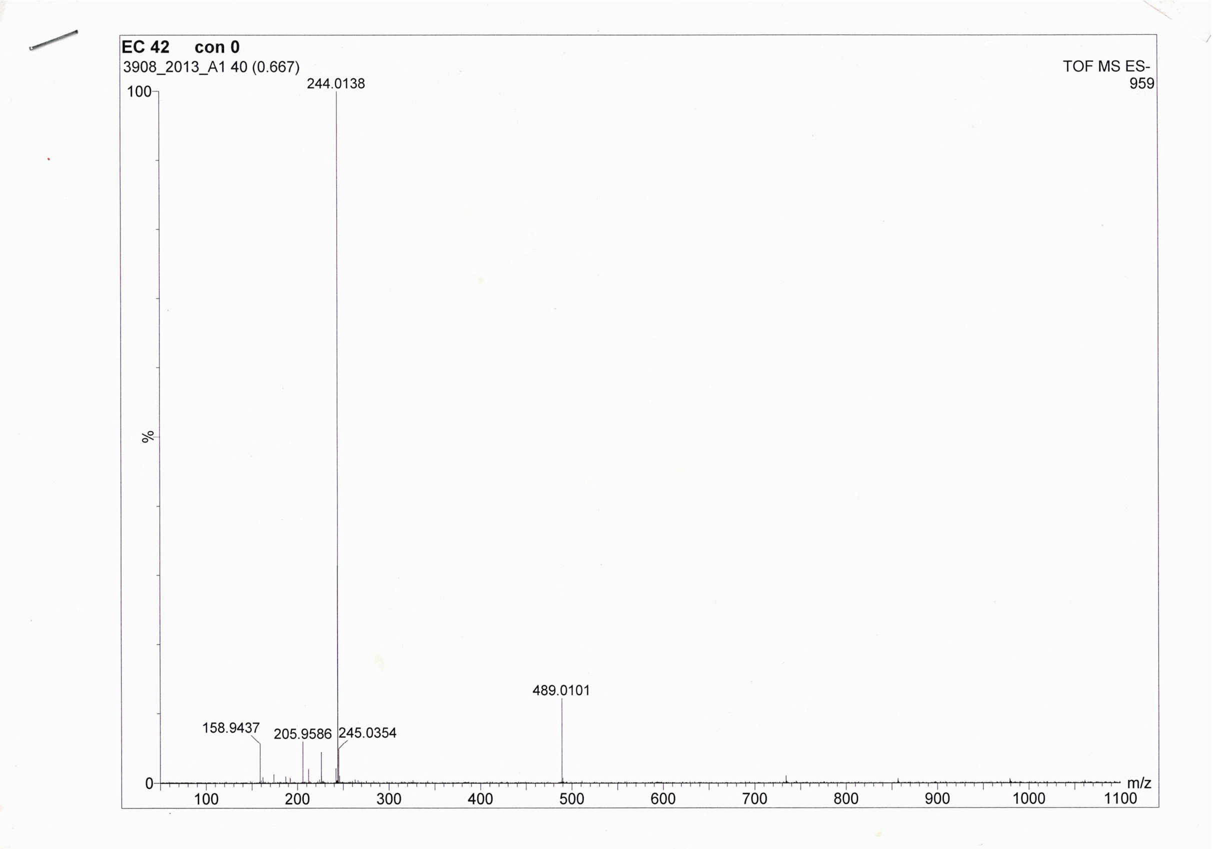

MS spectrum of compound **26**

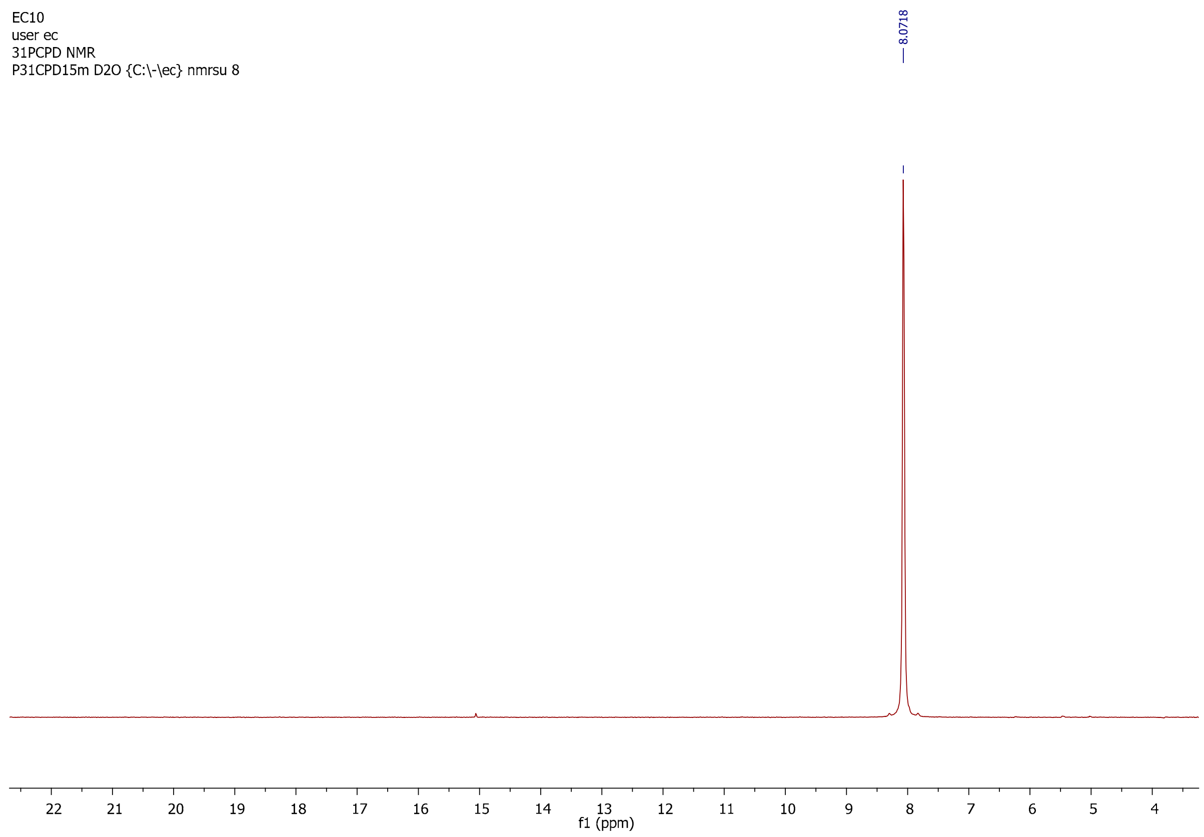

^{31}P NMR spectrum of compound **27**

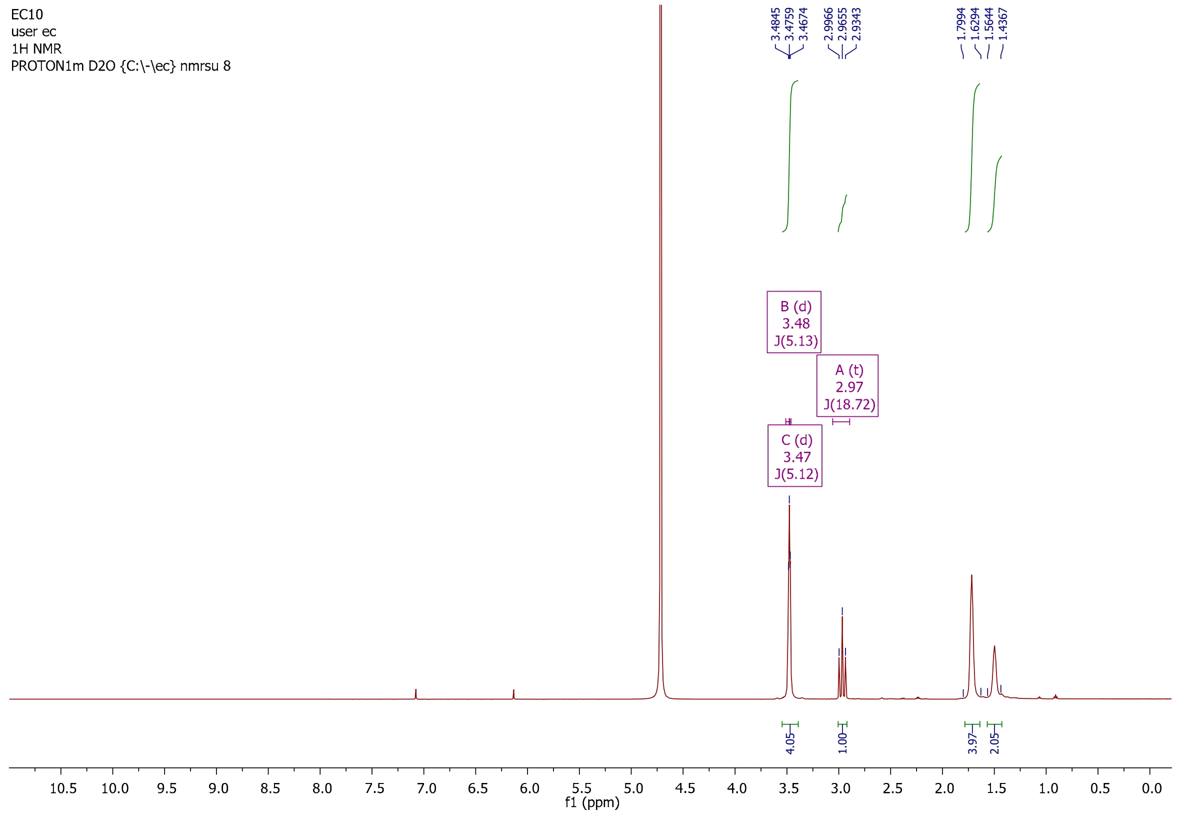

^{1}H NMR spectrum of compound **27**

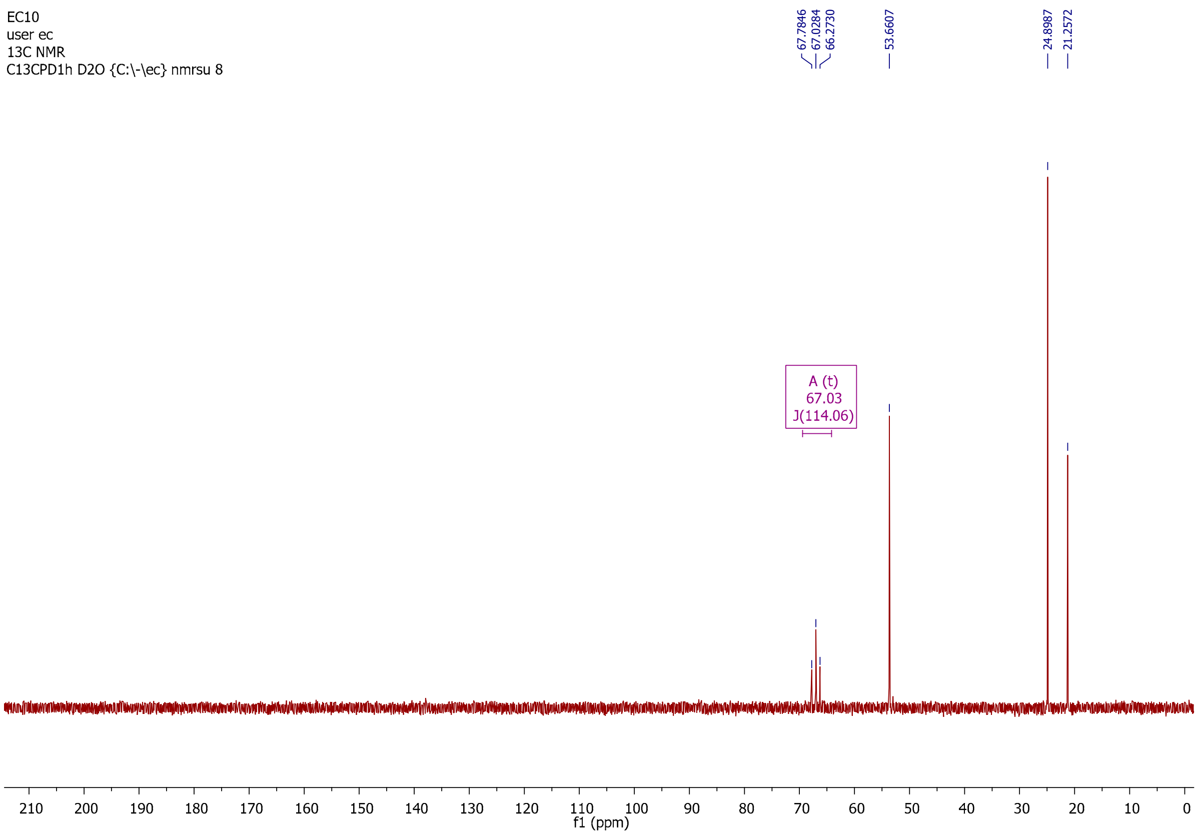

^{13}C NMR spectrum of compound **27**

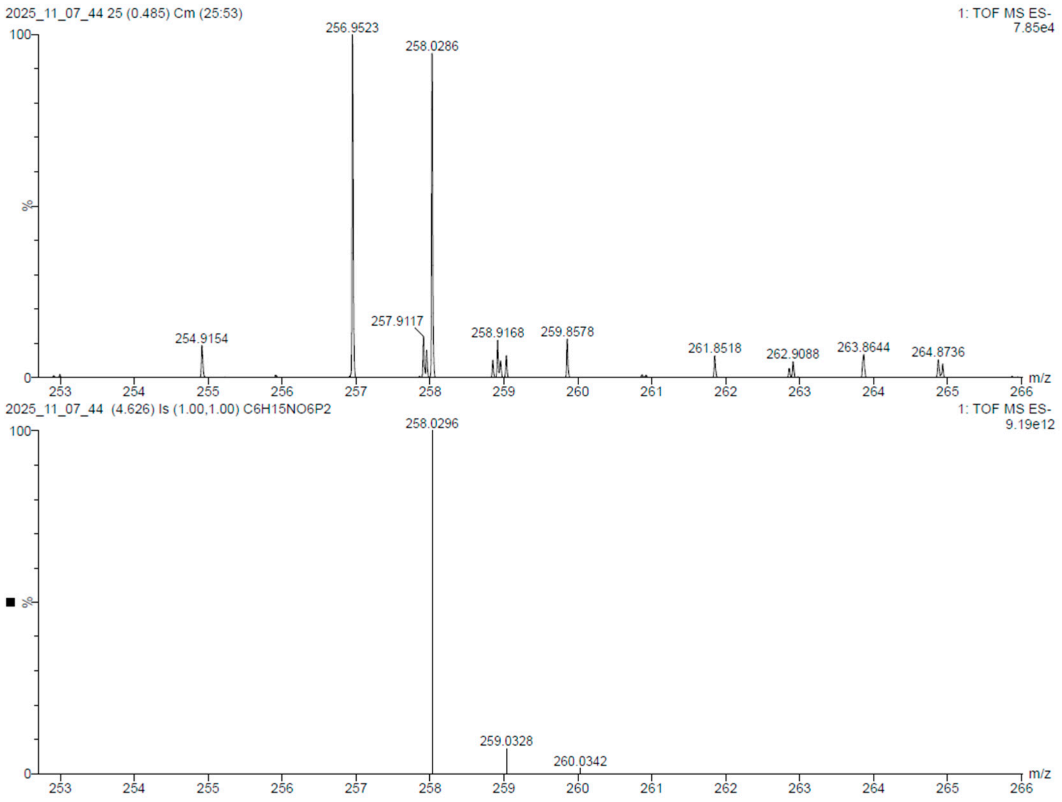

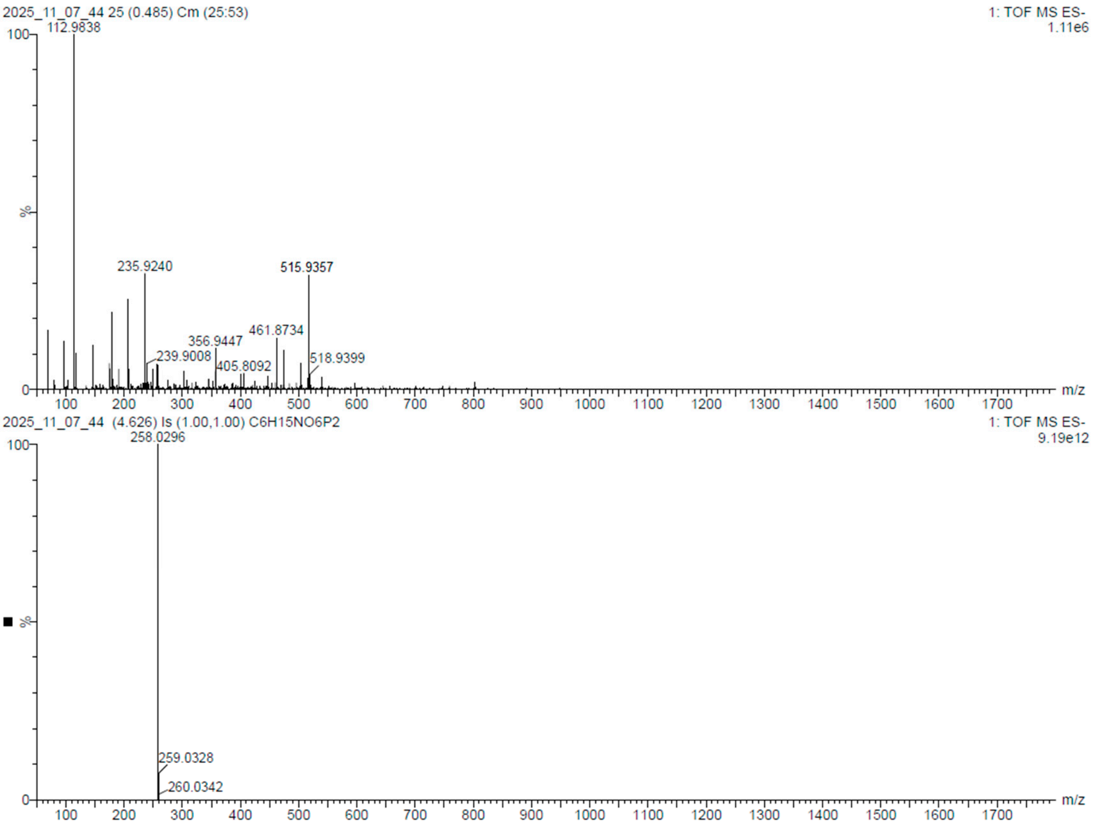

MS spectrum of compound **27**

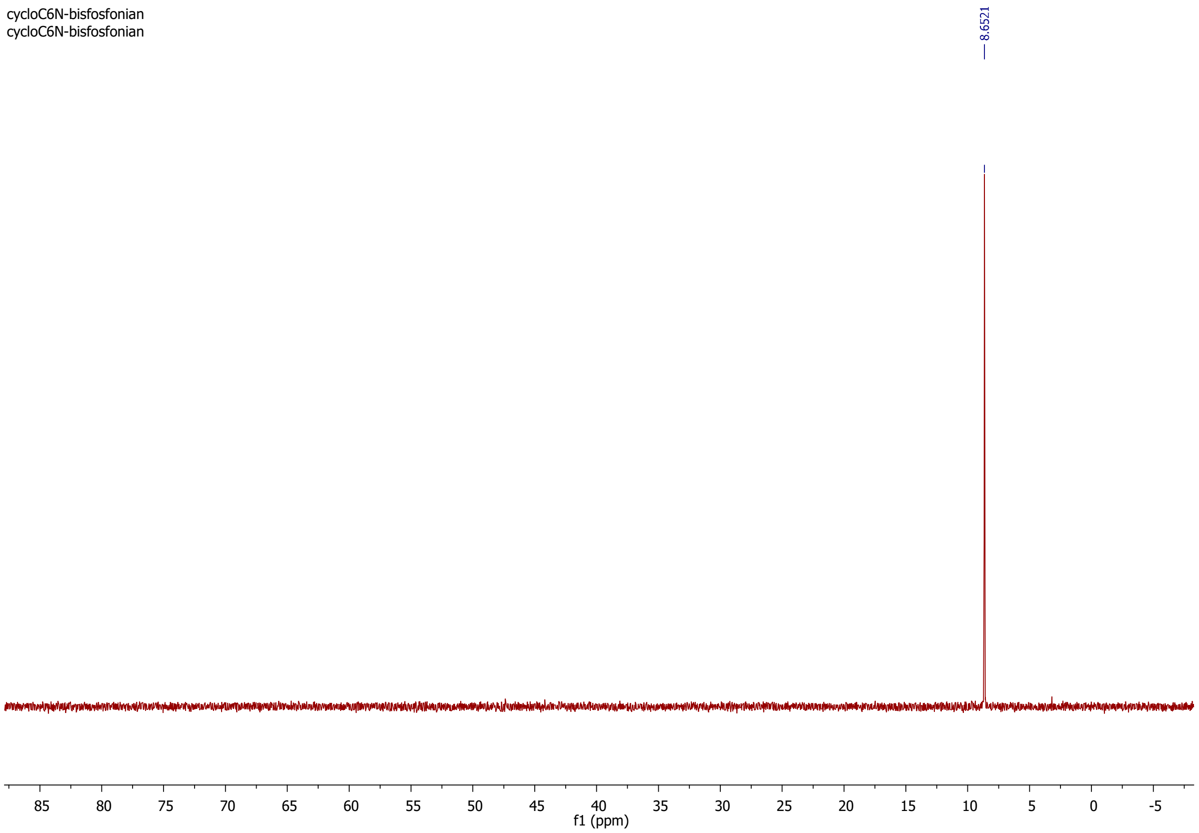

^{31}P NMR spectrum of compound **28**

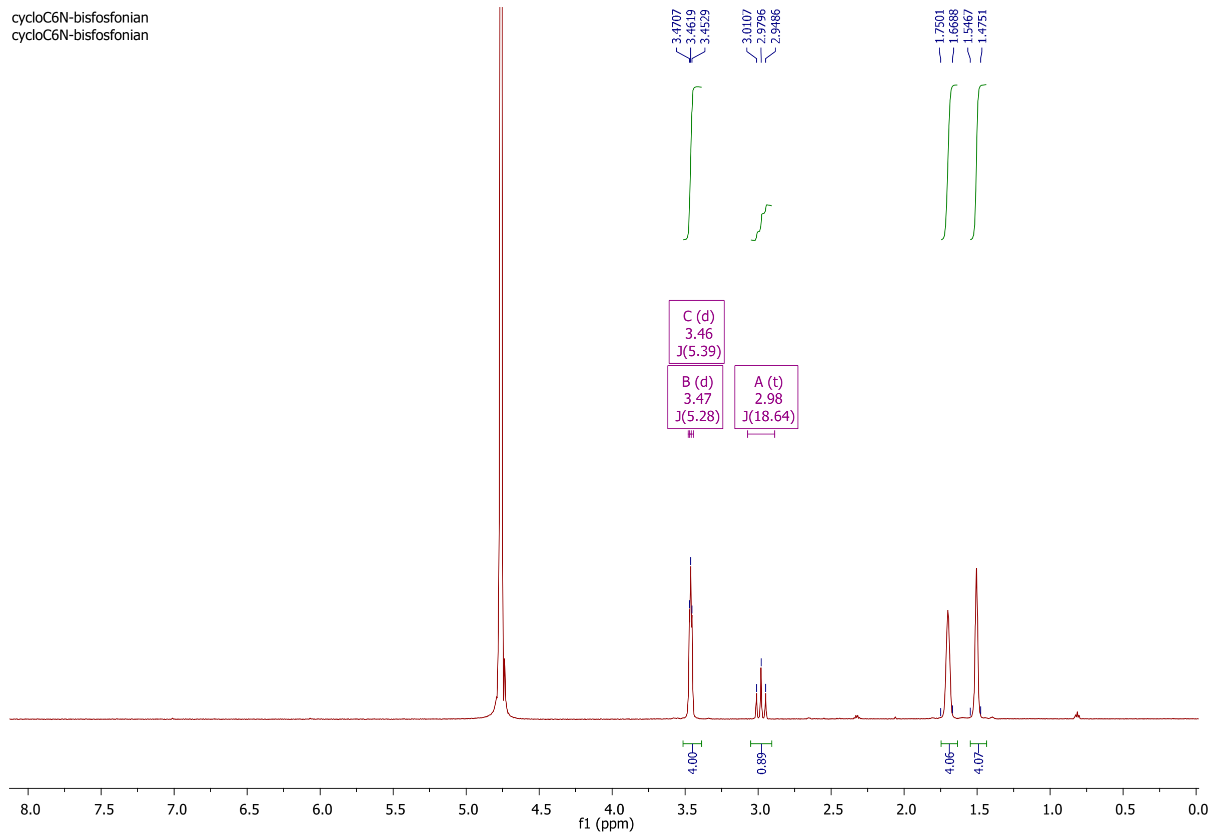

^{1}H NMR spectrum of compound **28**

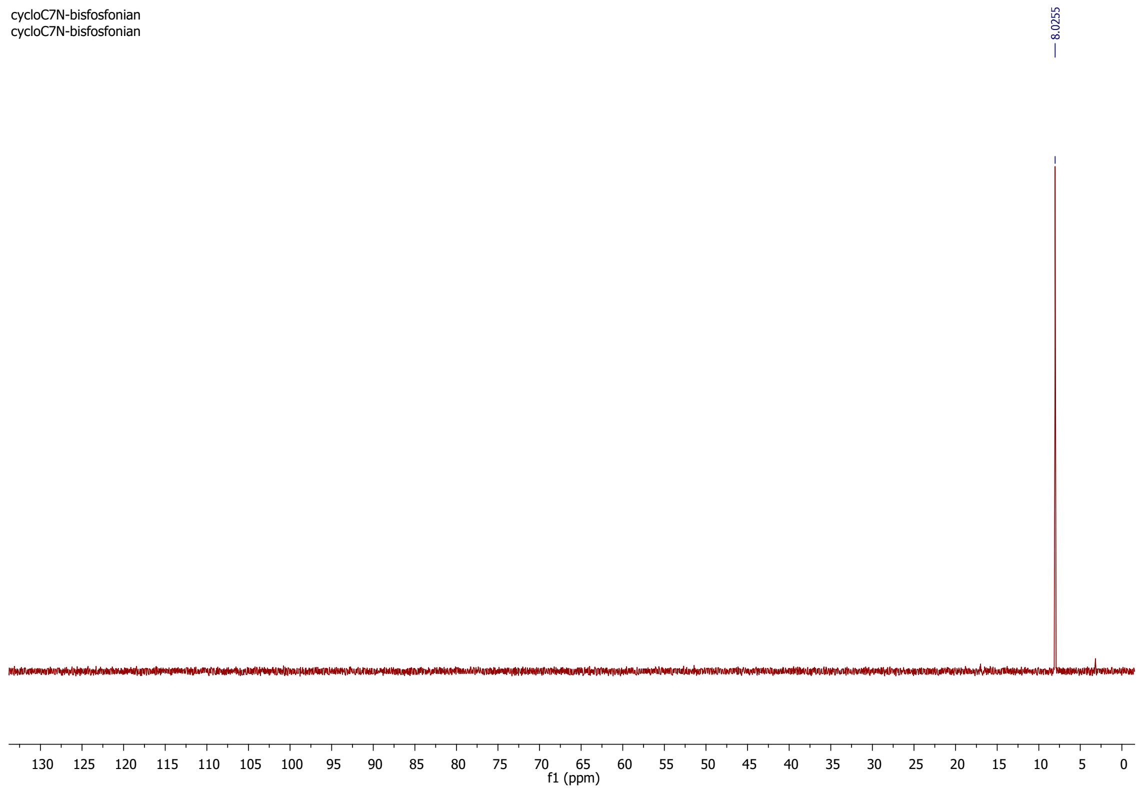

^{31}P NMR spectrum of compound **29**

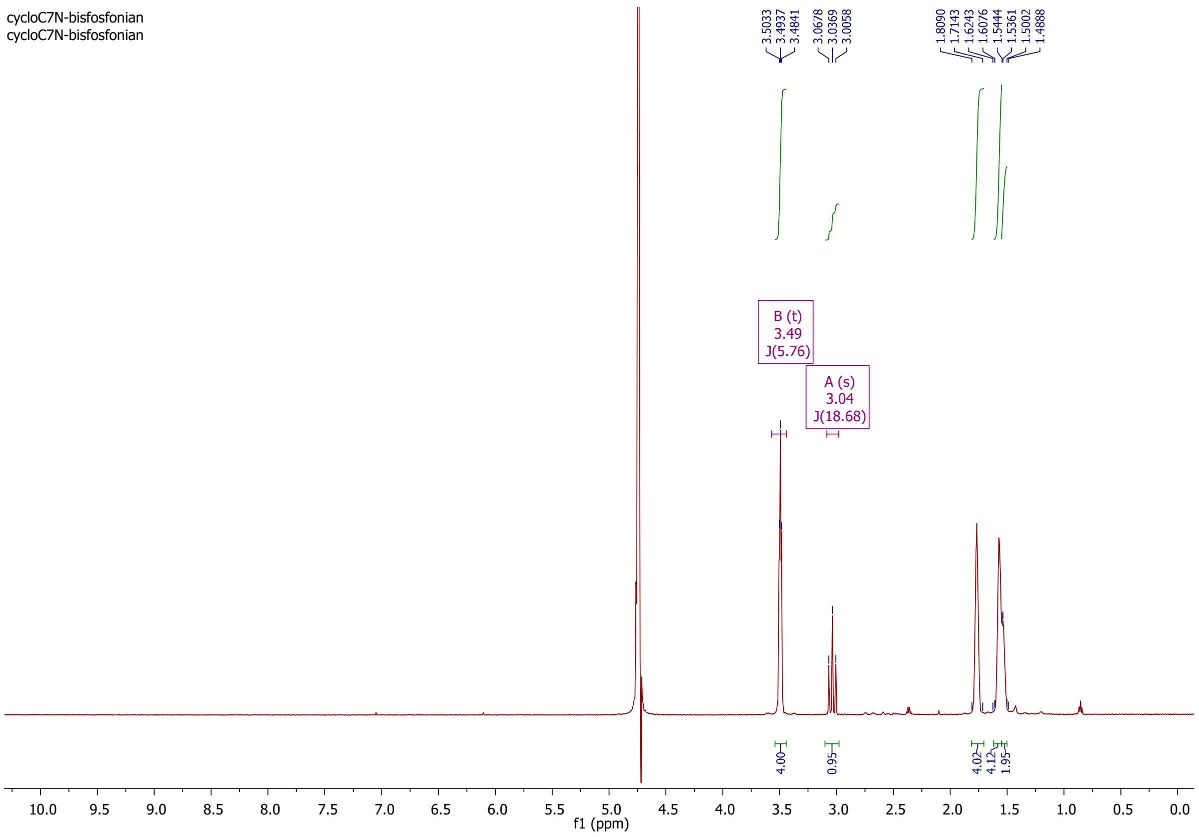

^{1}H NMR spectrum of compound **29**

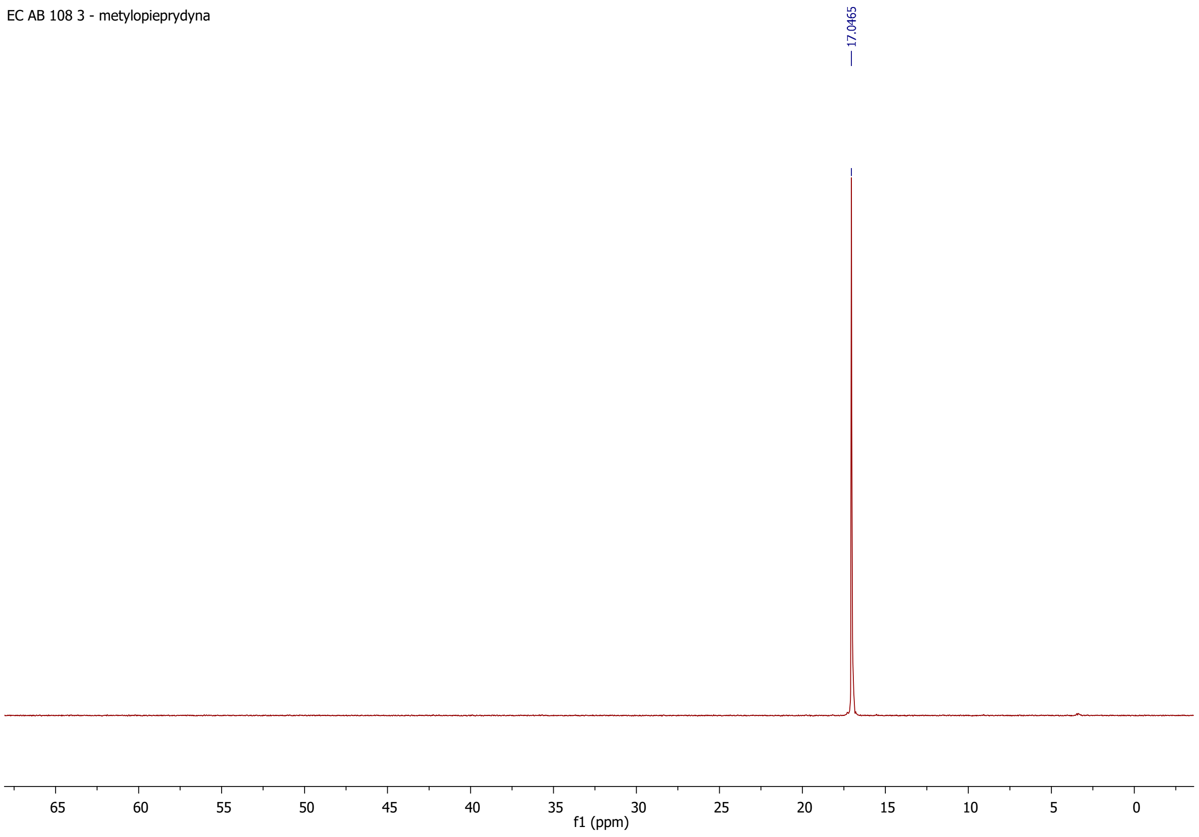

^{31}P NMR spectrum of compound **30**

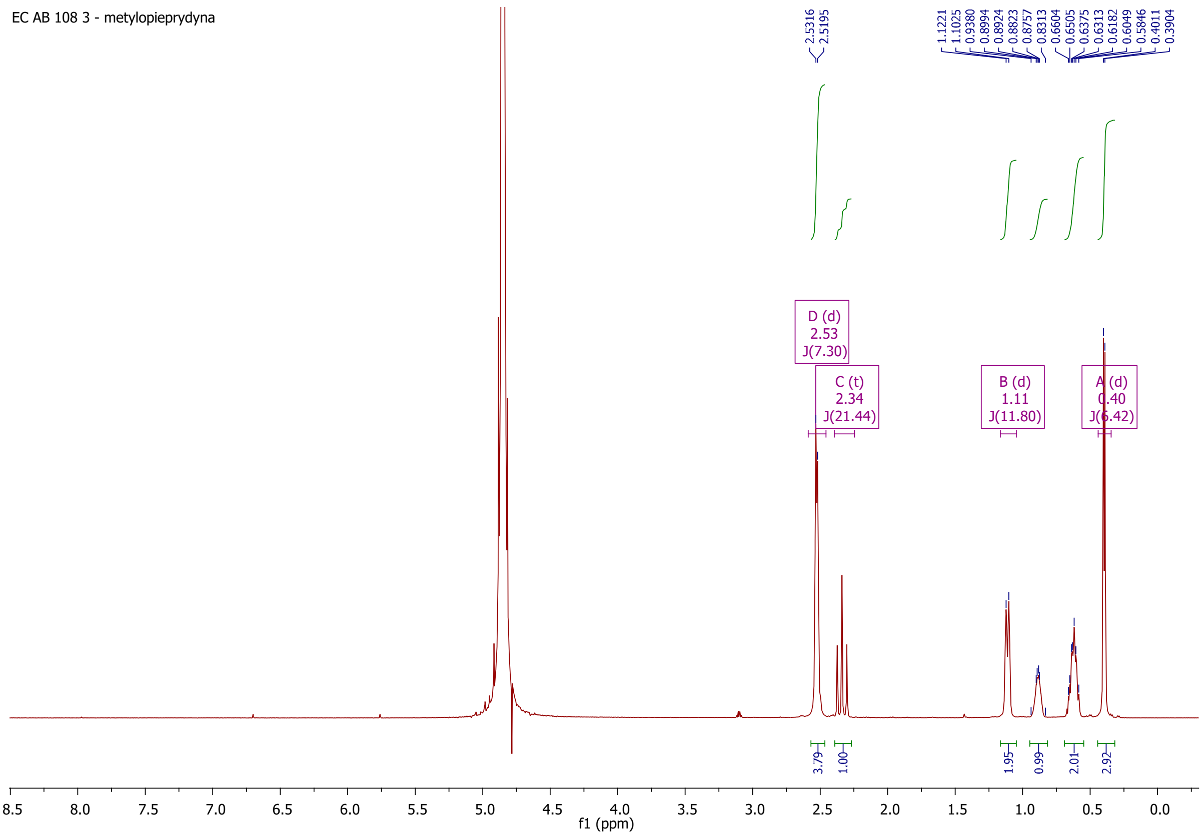

^{1}H NMR spectrum of compound **30**

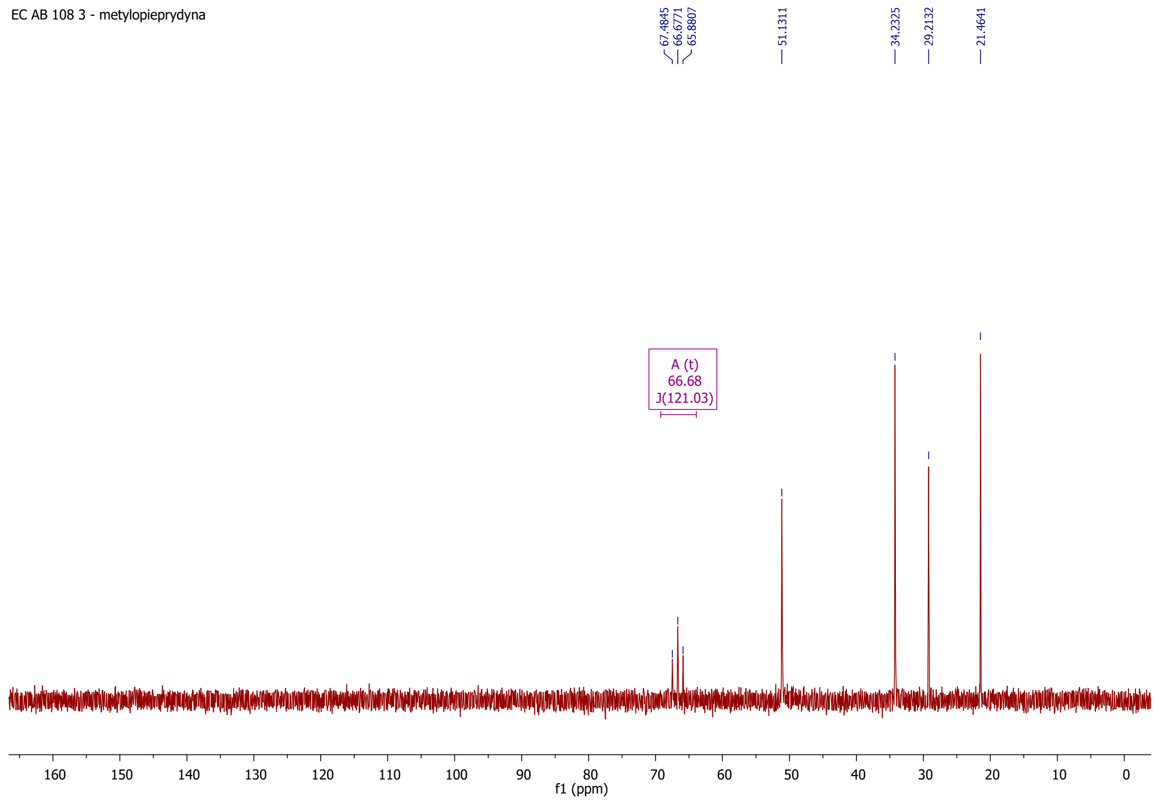

^{13}C NMR spectrum of compound **30**

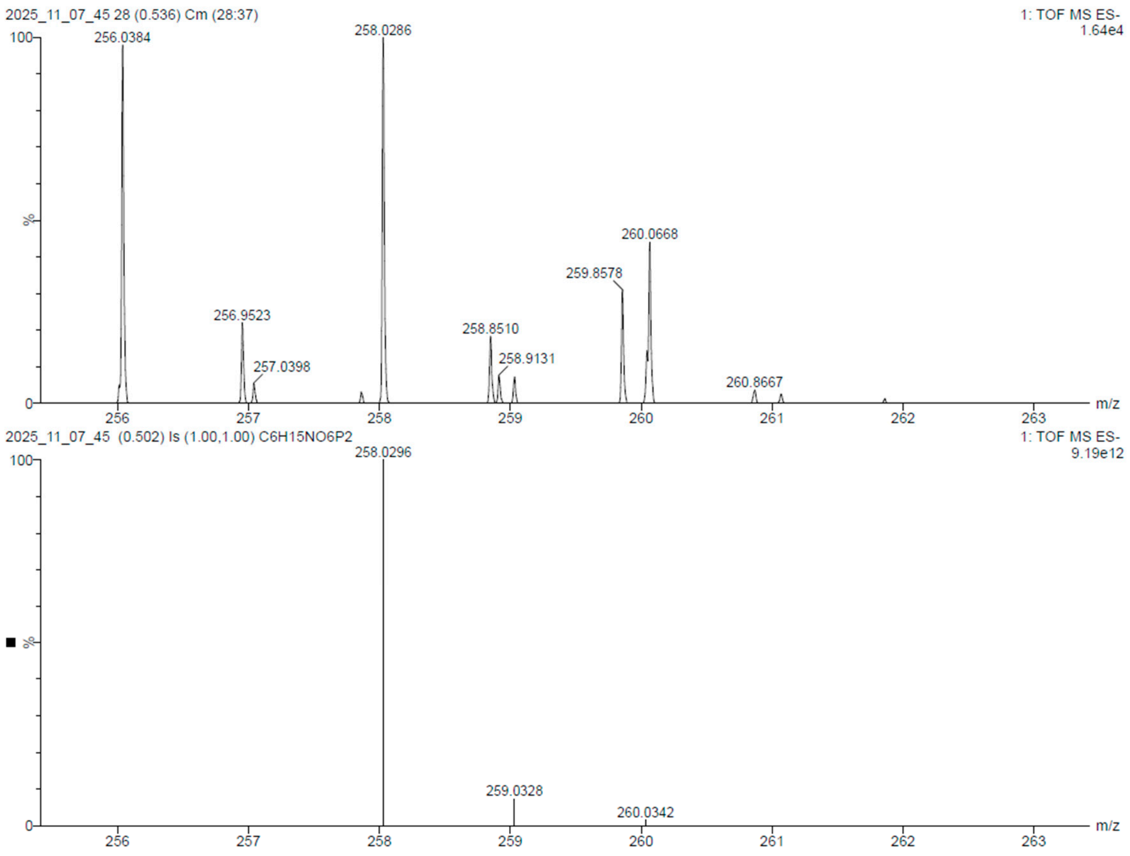

MS spectrum of compound **30**

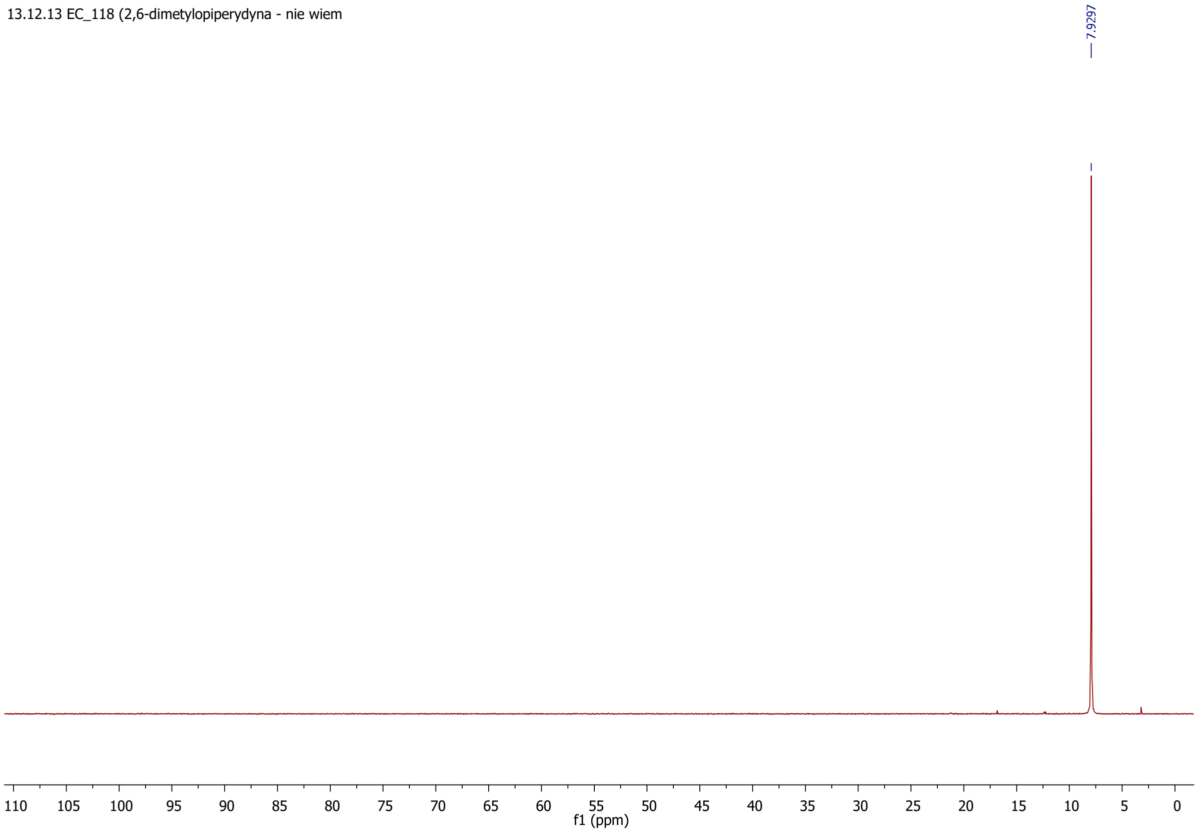

^{31}P NMR spectrum of compound **31**

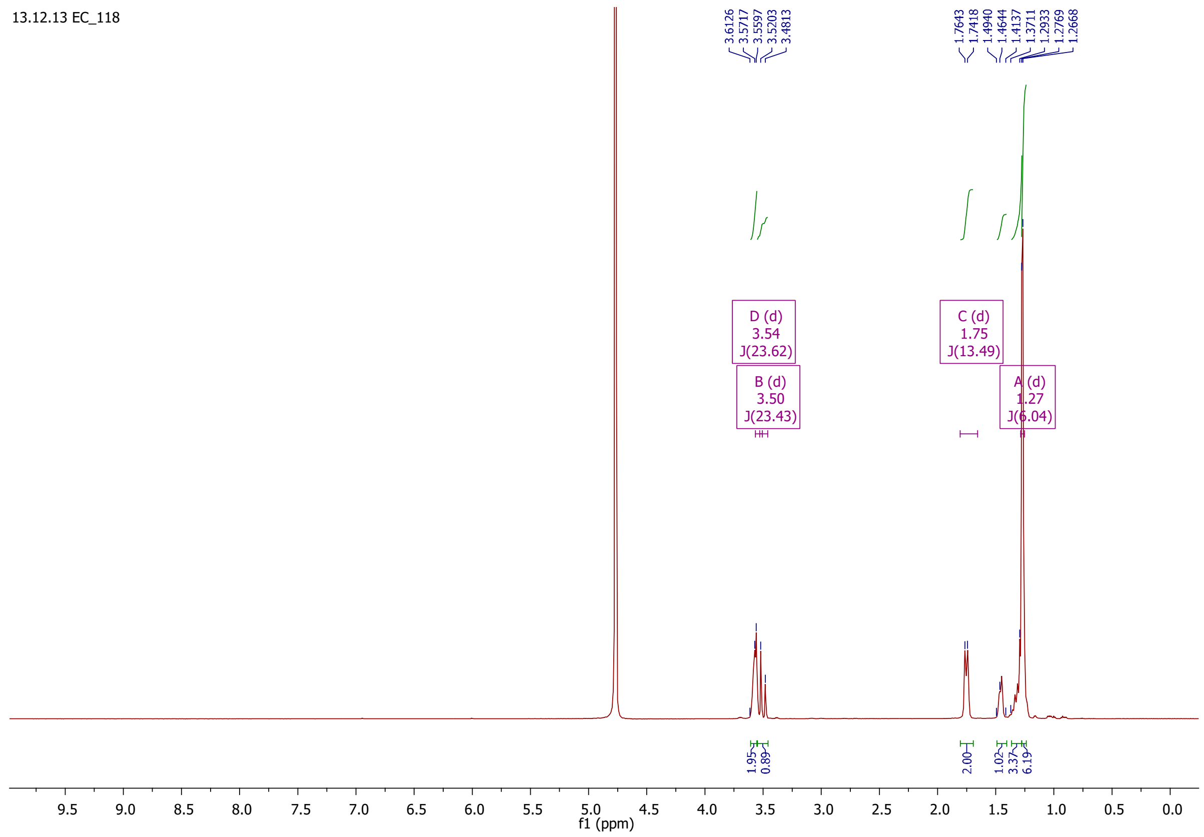

^{31}P NMR spectrum of compound **31**

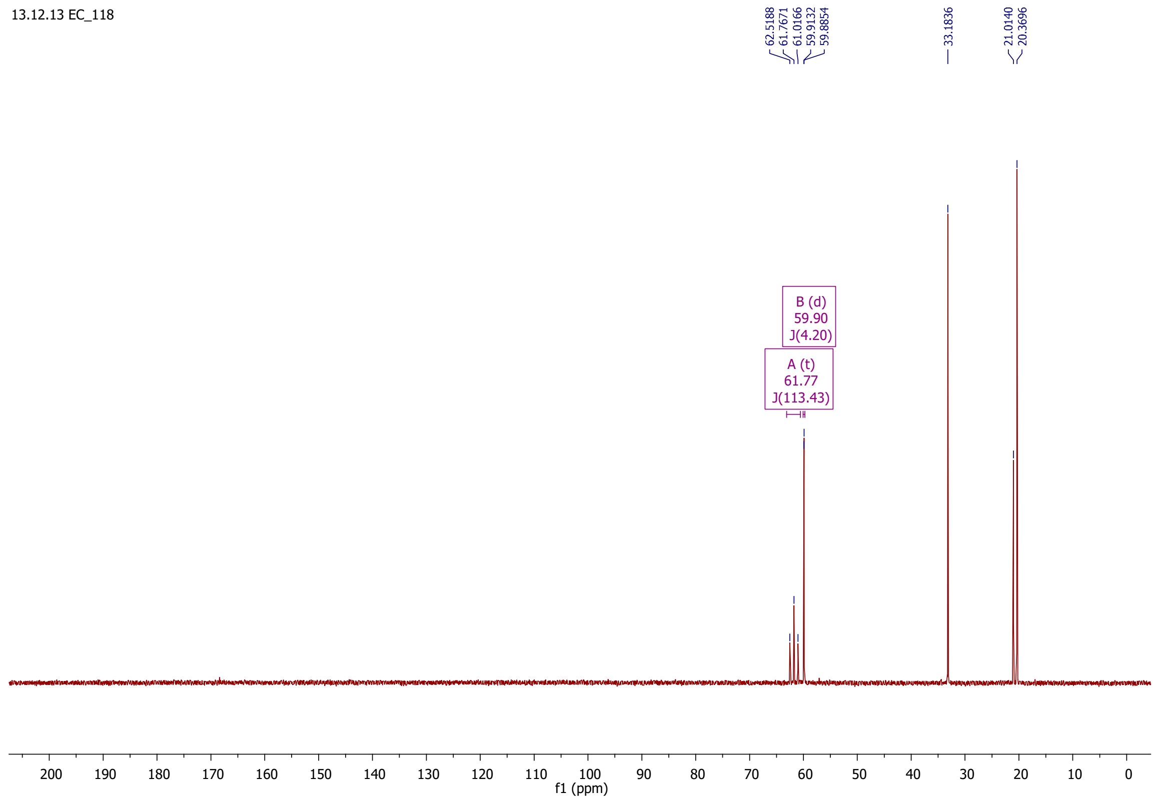

^{1}H NMR spectrum of compound **31**

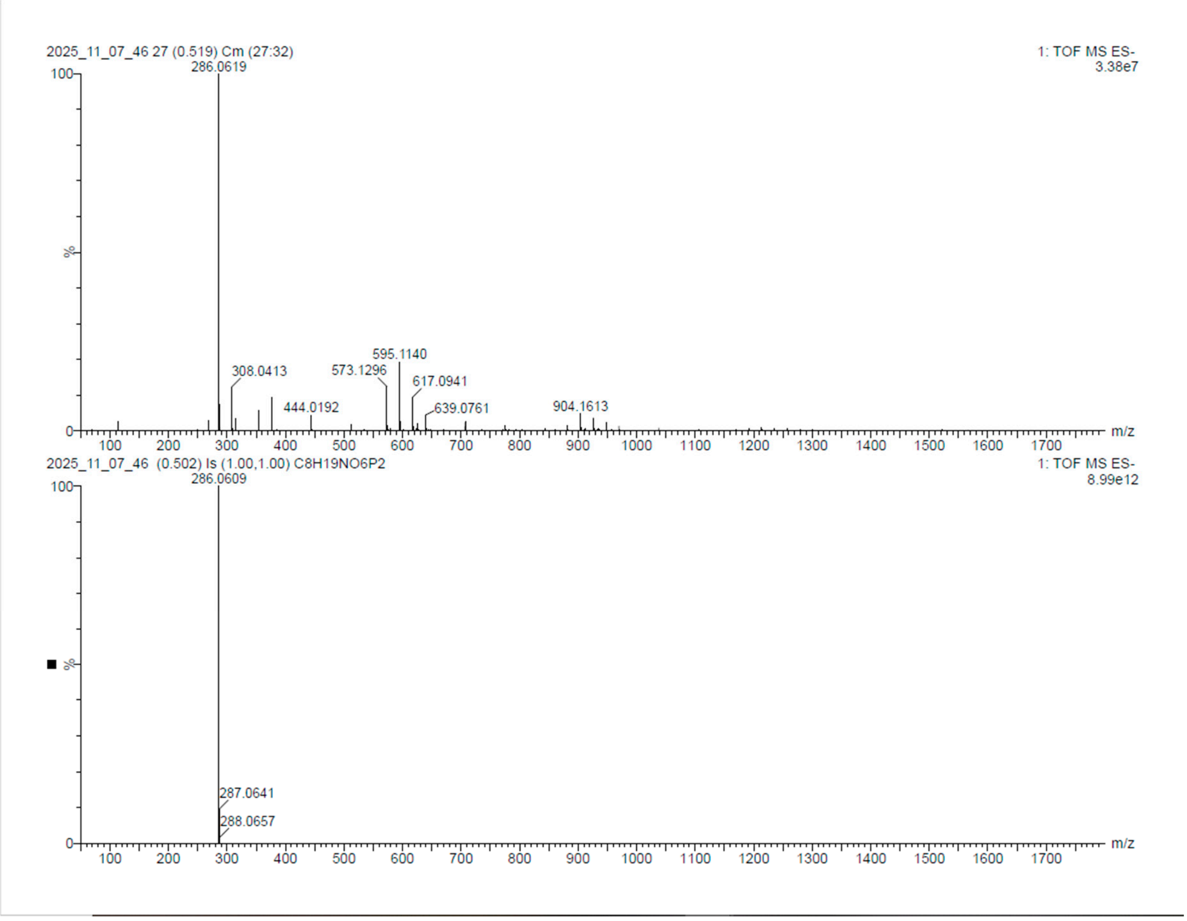

MS spectrum of compound **31**

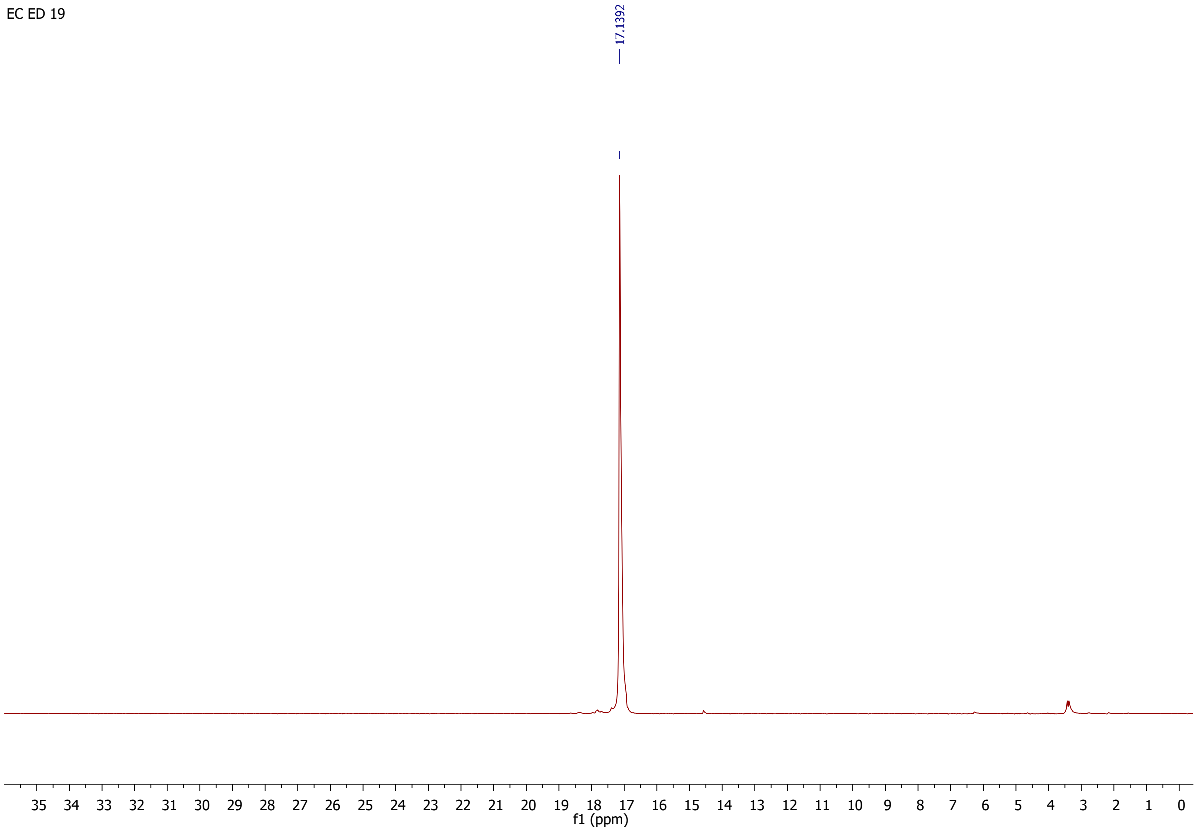

^{31}P NMR spectrum of compound **32**

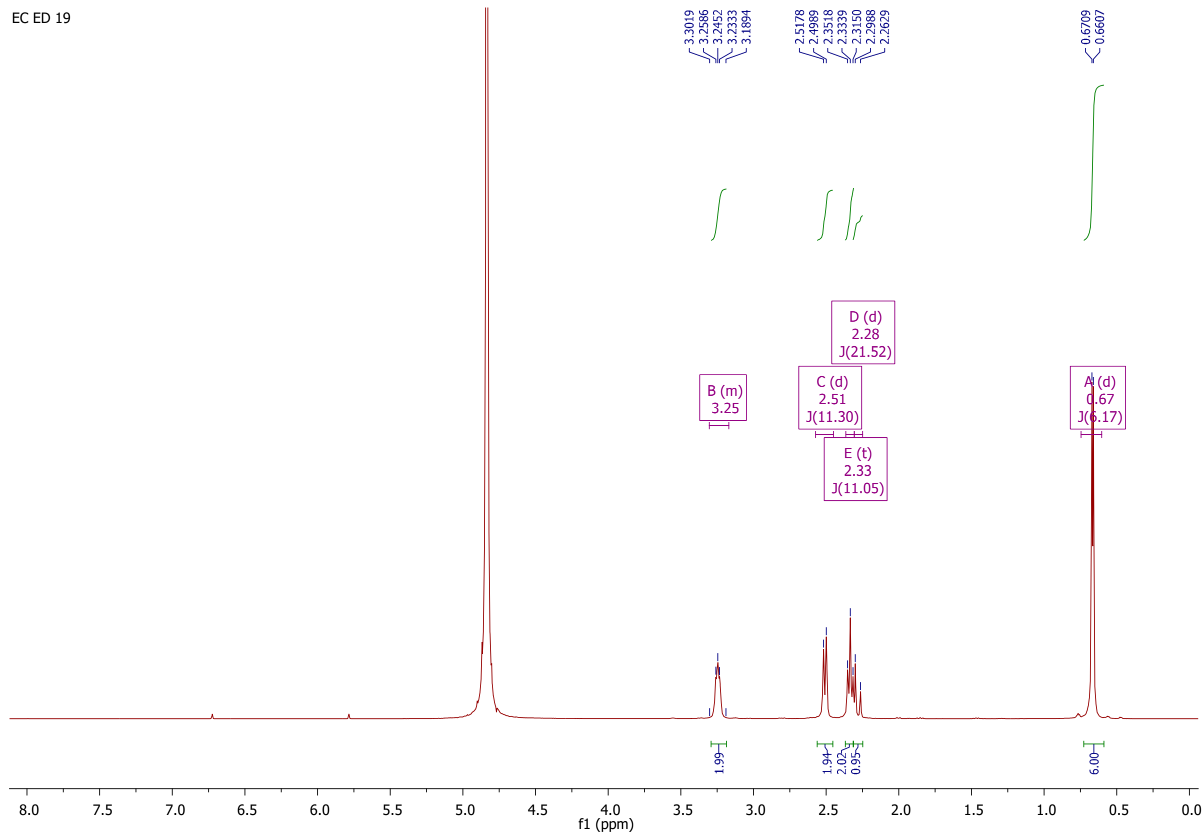

^{1}H NMR spectrum of compound **32**

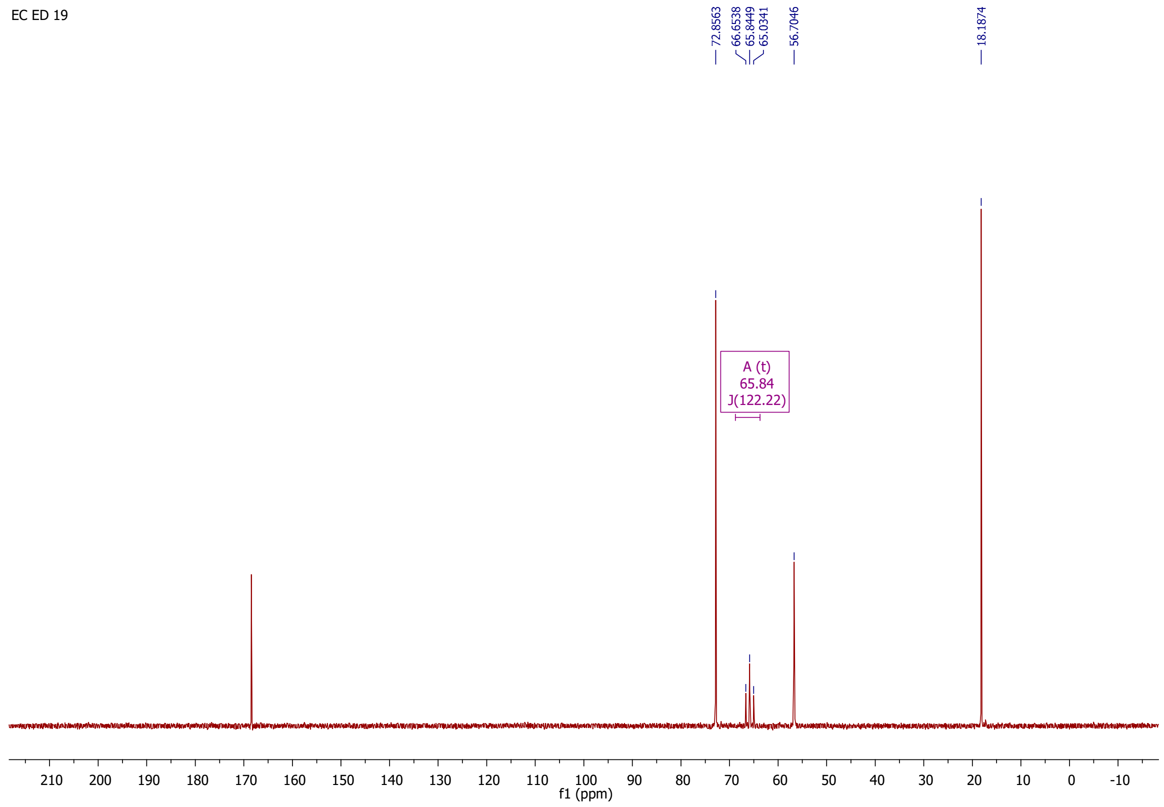

^{13}C NMR spectrum of compound **32**

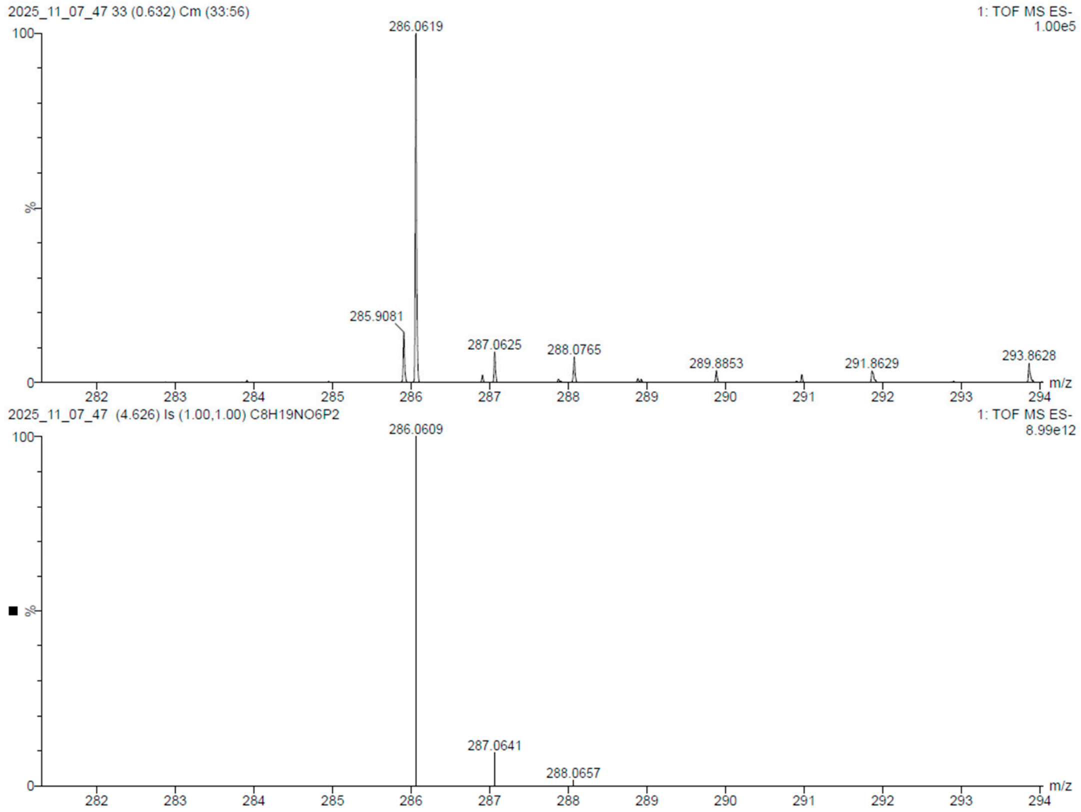

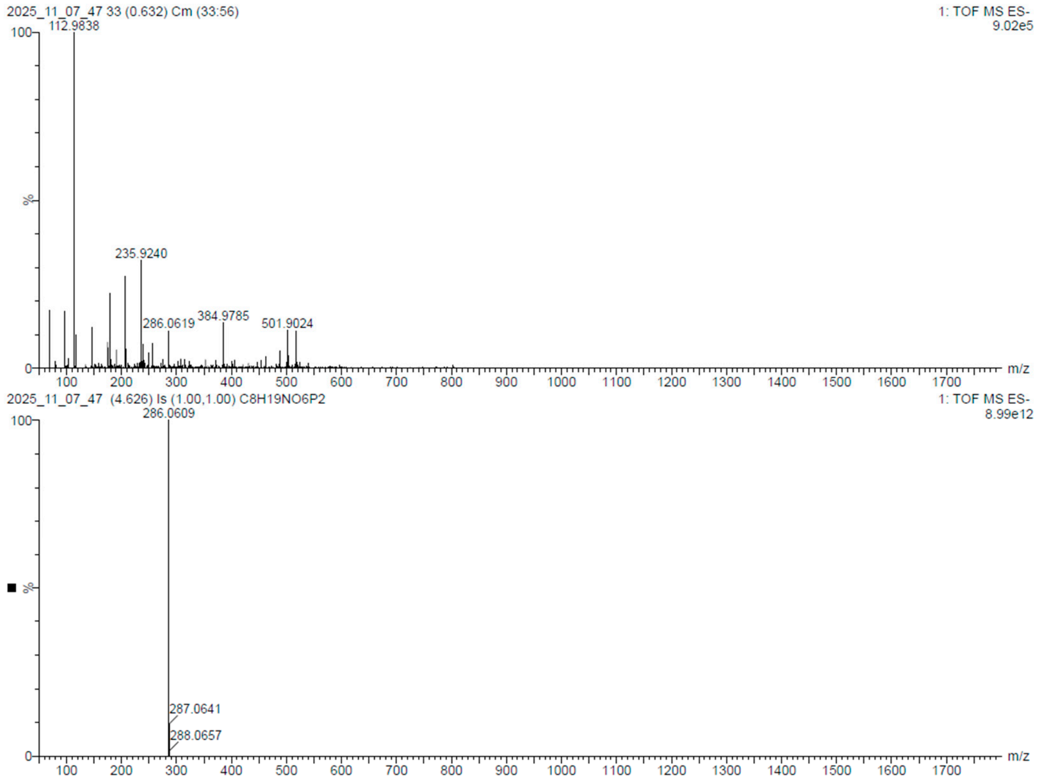

MS spectrum of compound **32**

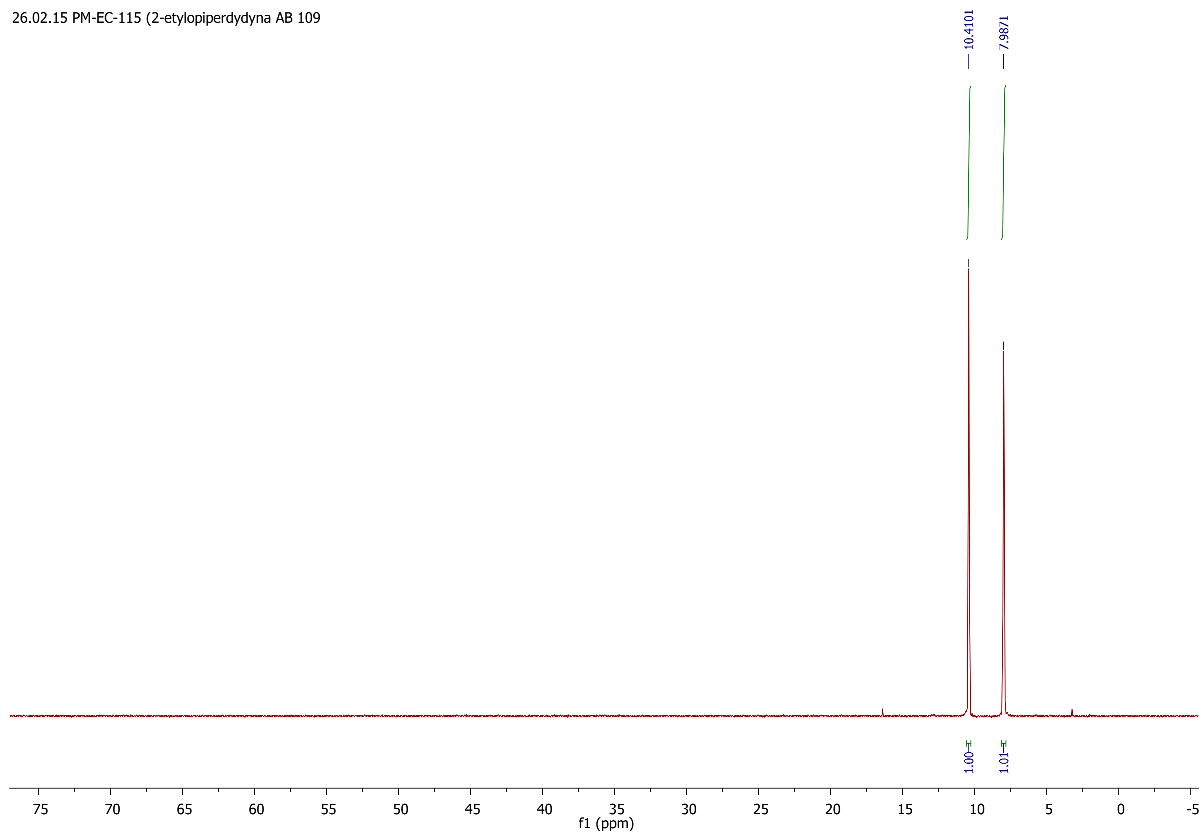

^{31}P NMR spectrum of compound **33**

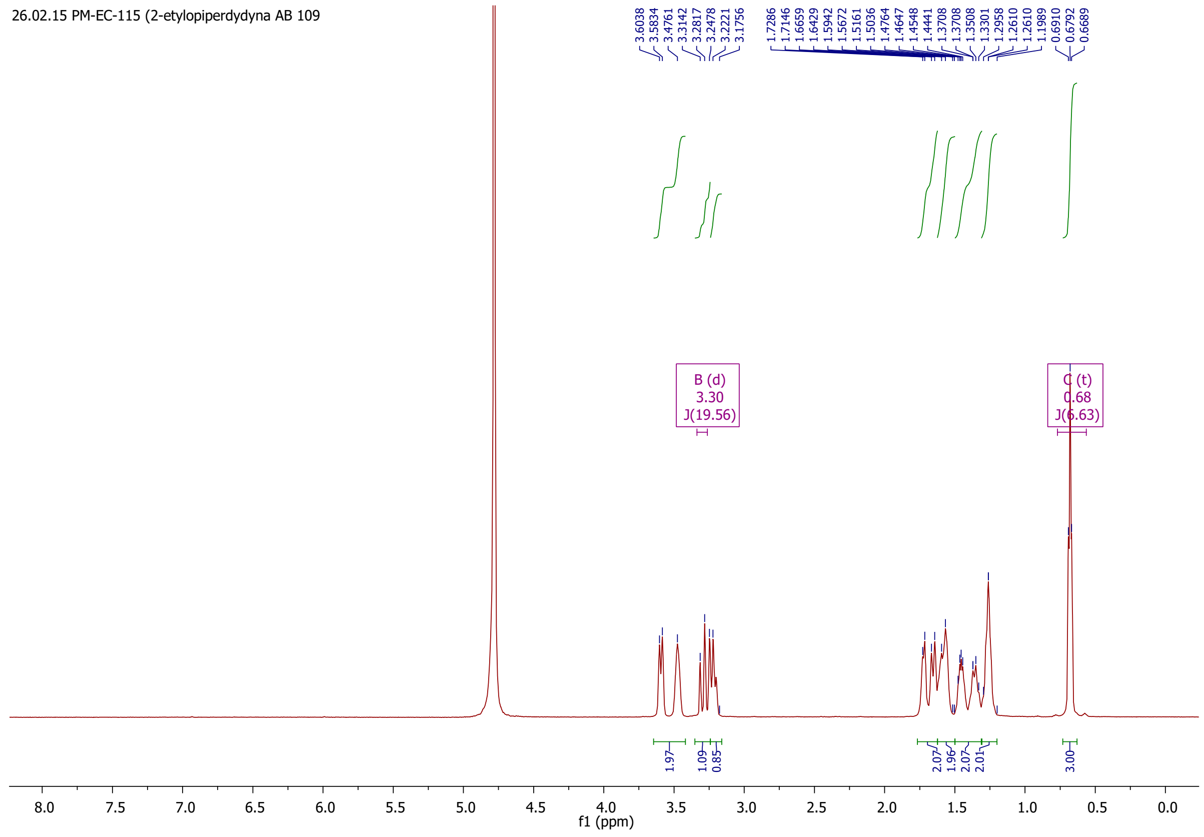

^{1}H NMR spectrum of compound **33**

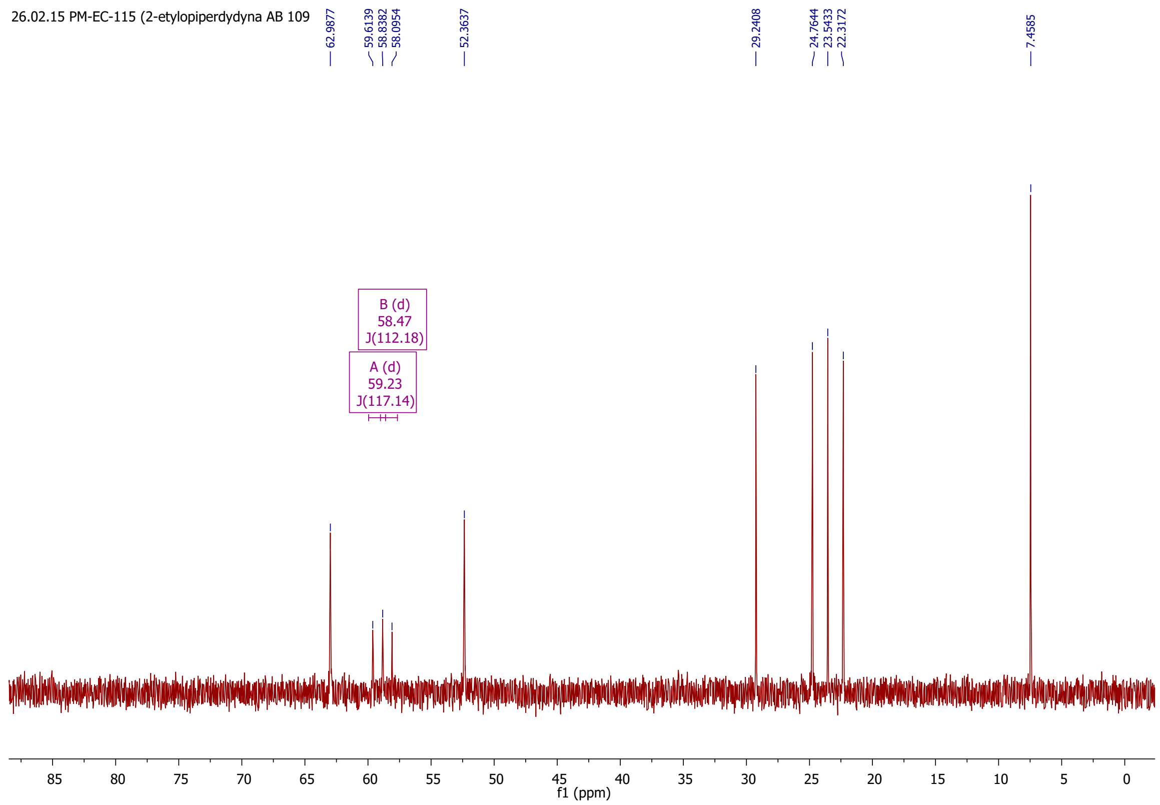

^{13}C NMR spectrum of compound **33**

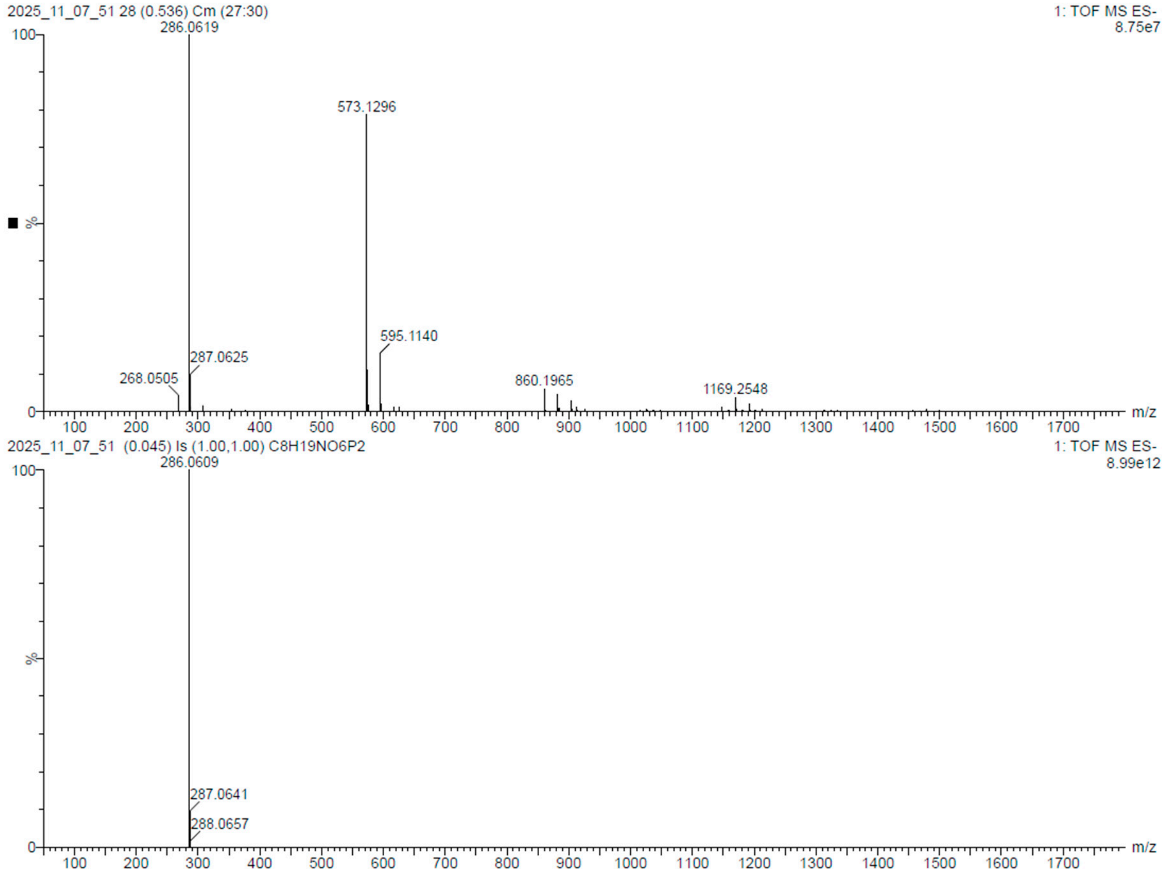

MS spectrum of compound **33**

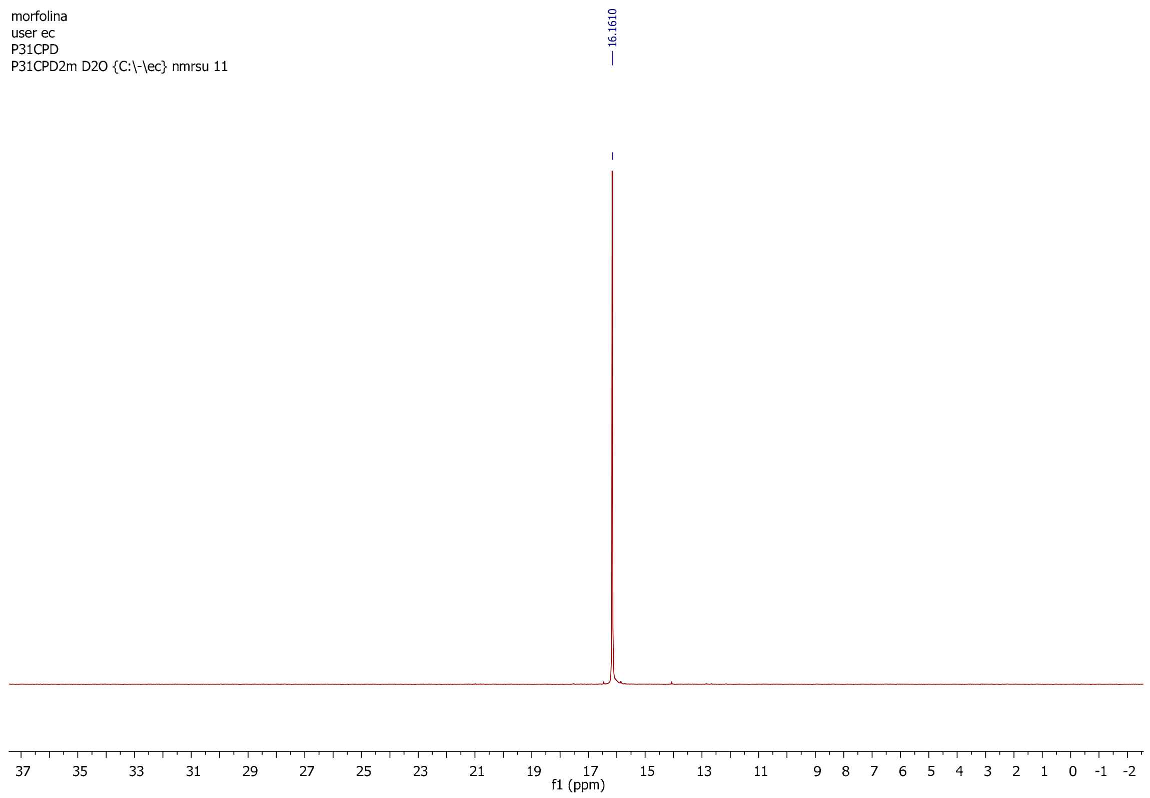

^{31}P NMR spectrum of compound **34**

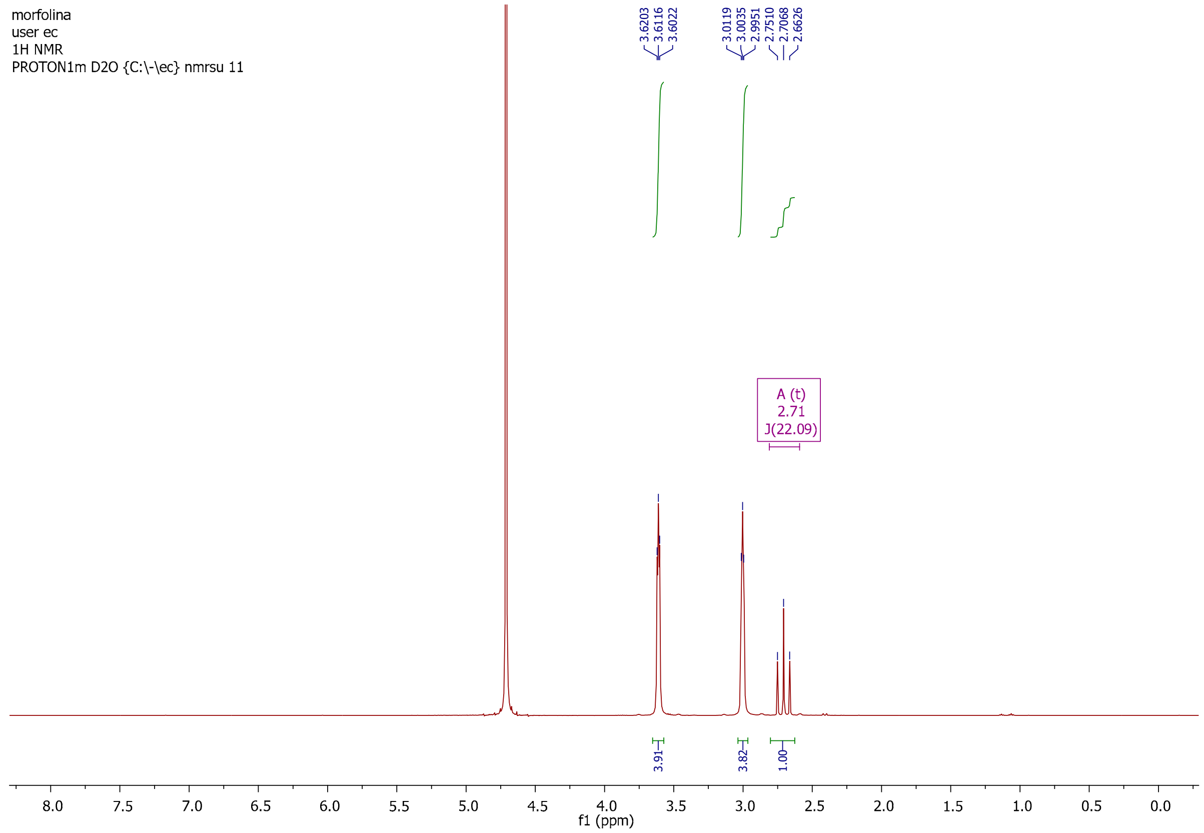

^{1}H NMR spectrum of compound **34**

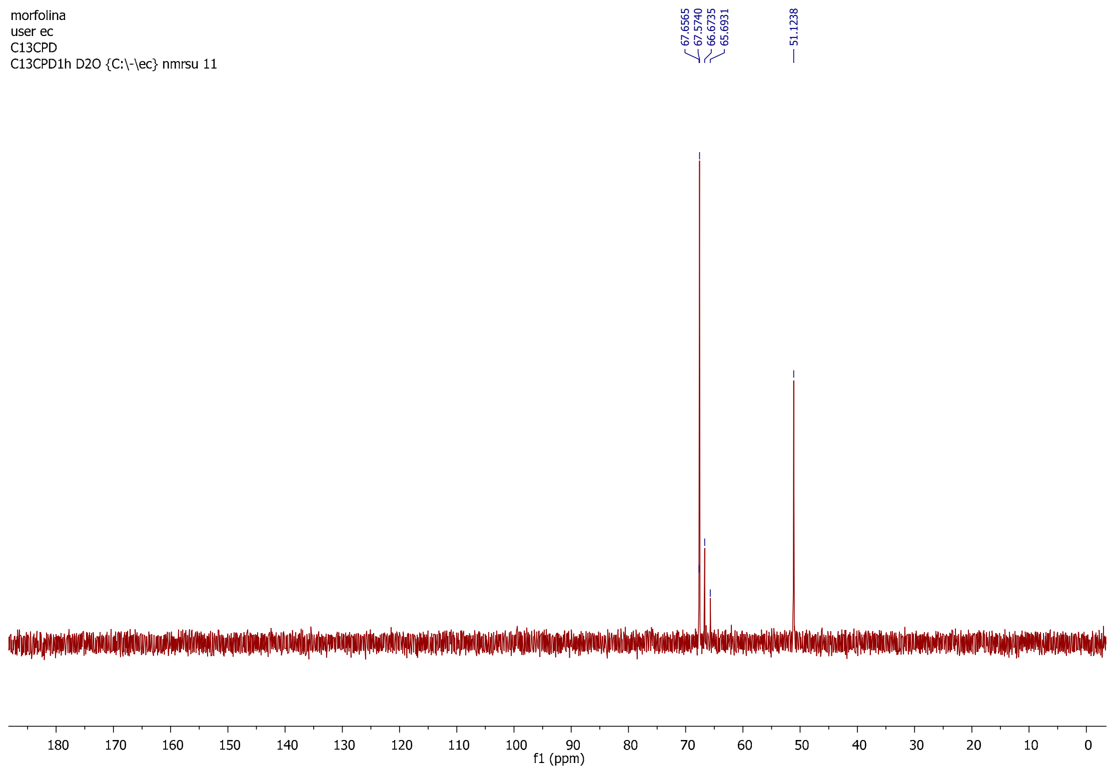

^{13}C NMR spectrum of compound **34**

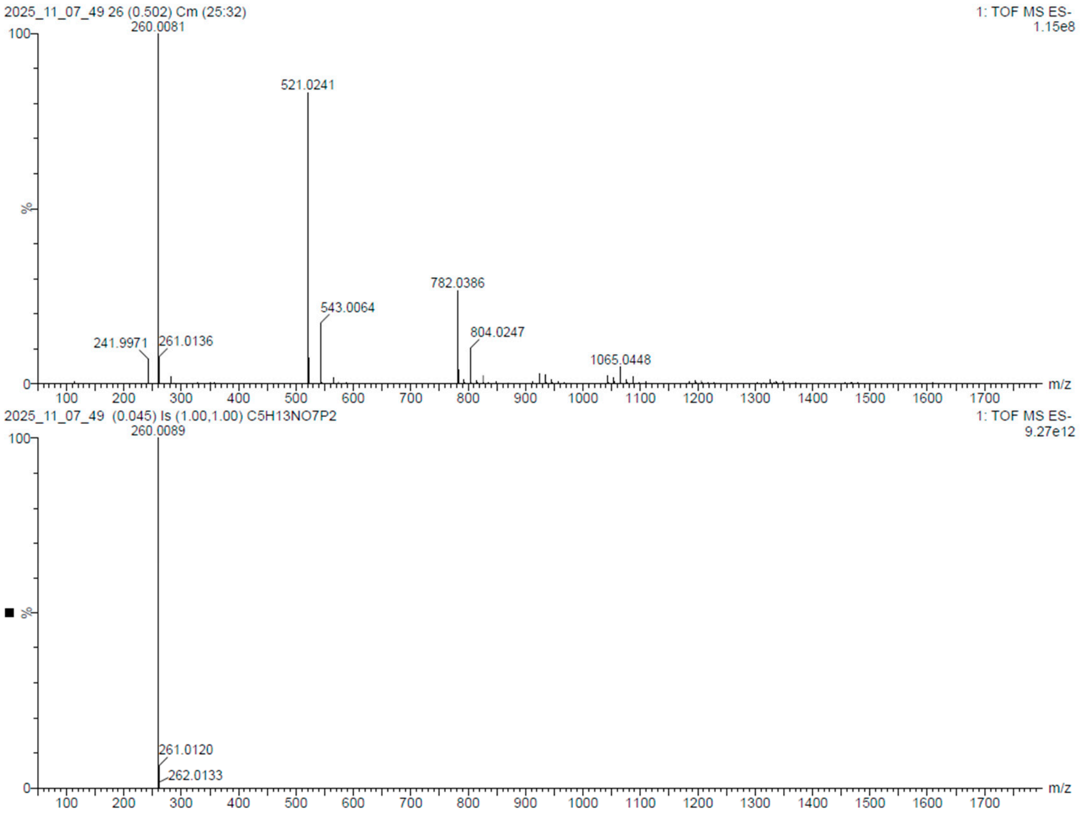

MS spectrum of compound **34**

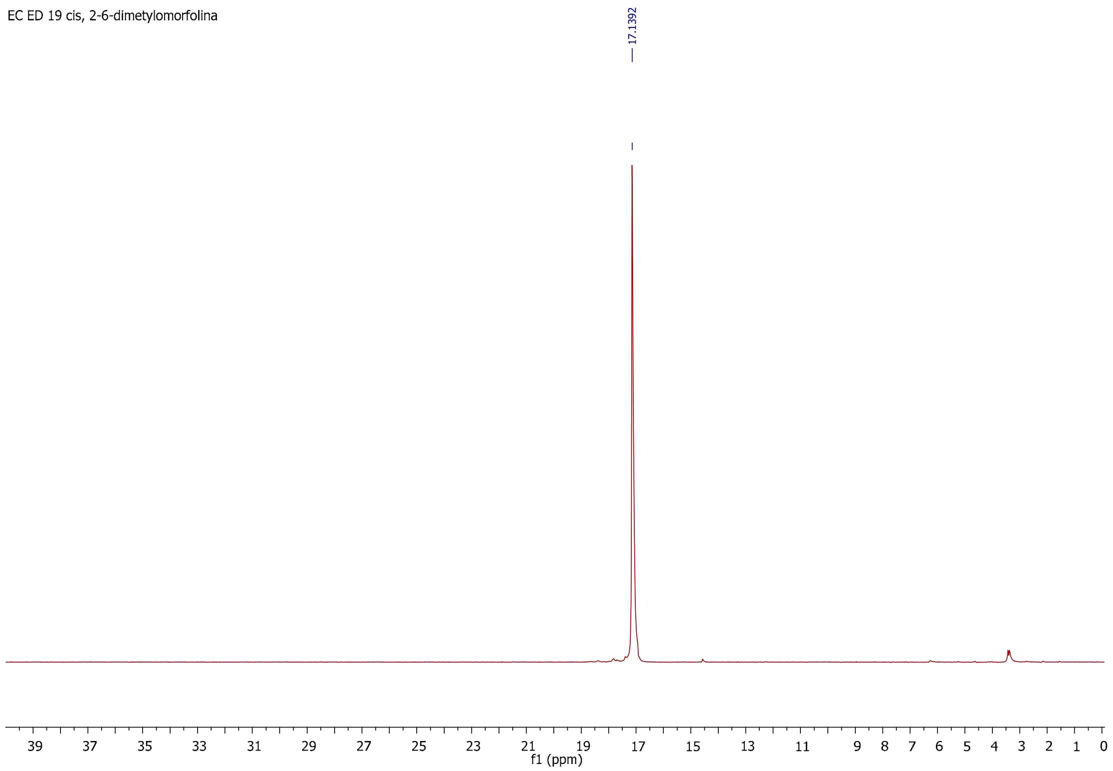

^{31}P NMR spectrum of compound **35**

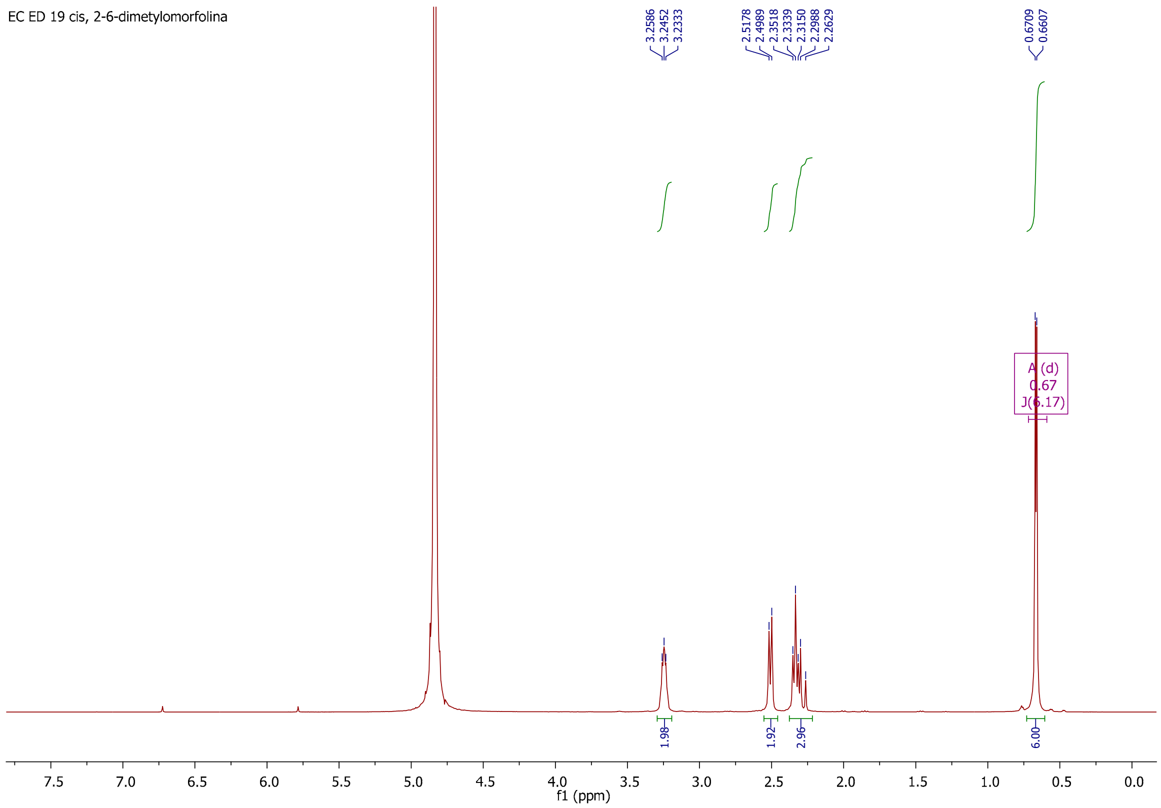

^{1}H NMR spectrum of compound **35**

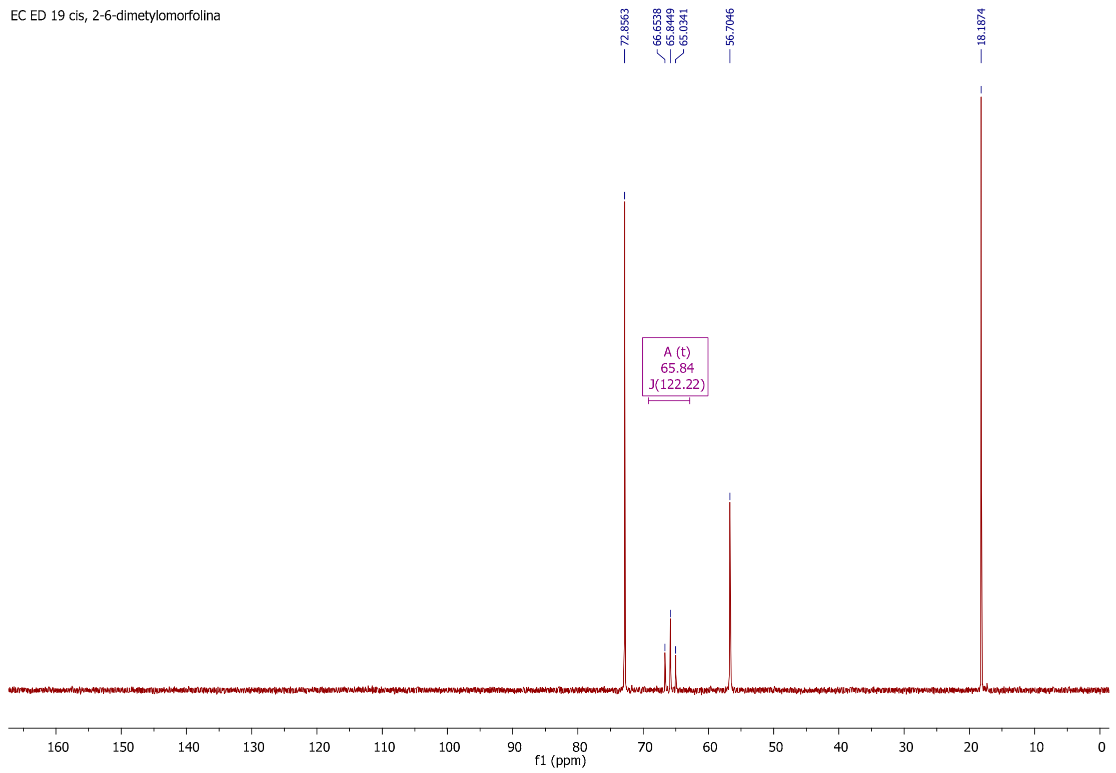

^{13}C NMR spectrum of compound **34**

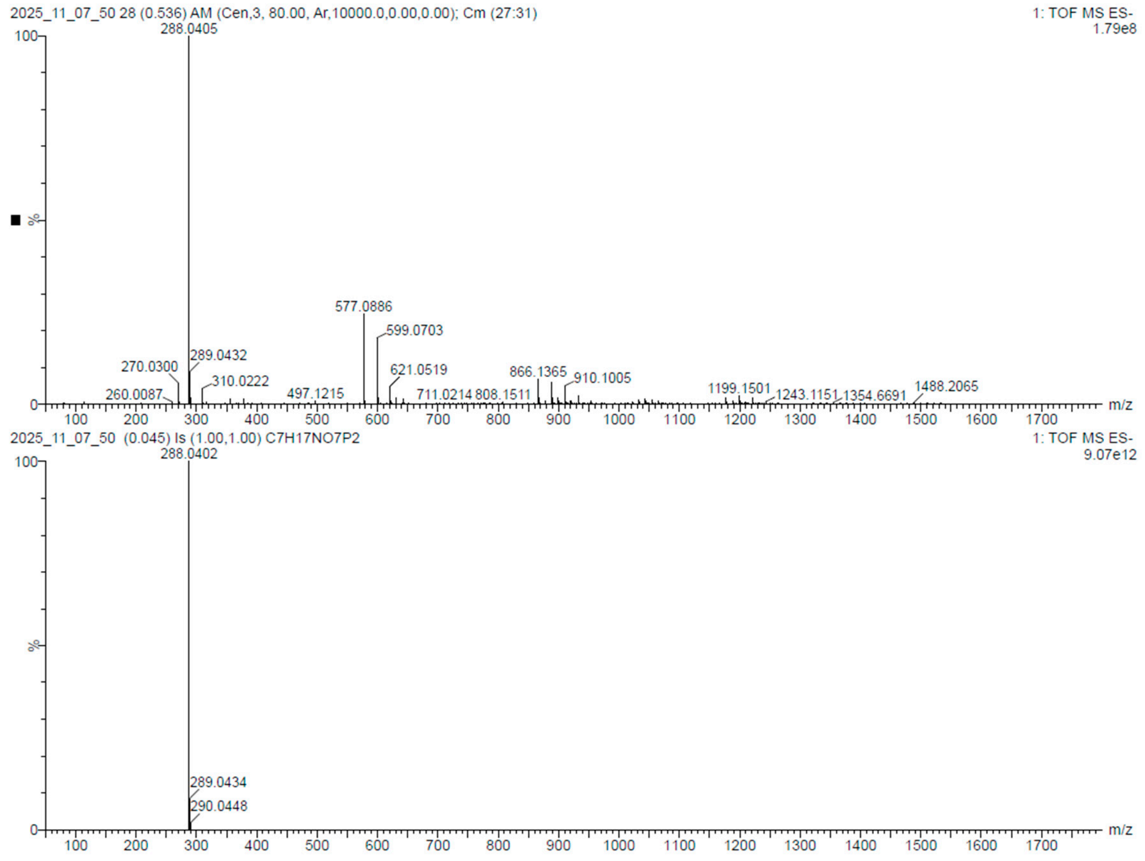

MS spectrum of compound **35**
